# Diagnostic Accuracy of Microbiome‐Derived Biomarkers in Periodontitis: Systematic Review and Meta‐Analysis

**DOI:** 10.1111/jre.13377

**Published:** 2025-01-13

**Authors:** Anbo Dong, Gordon Proctor, Svetislav Zaric

**Affiliations:** ^1^ Centre for Host‐Microbiome Interactions, Faculty of Dentistry, Oral & Craniofacial Sciences King's College London London UK

**Keywords:** diagnostic accuracy, meta‐analysis, microbiome‐derived biomarkers, periodontitis, sensitivity, specificity, systematic review

## Abstract

**Aim:**

To evaluate the diagnostic accuracy of microbiome‐derived biomarkers for periodontitis in oral fluids (saliva and subgingival samples).

**Methods:**

This systematic review followed PRISMA guidelines. Electronic searches were performed across multiple databases from December 2022 to November 2024. Subgroup analyses, divided into saliva and subgingival samples, were performed using the Random Effects Model (REM), while individual biomarker sensitivity and specificity were evaluated through the Bivariate Random‐Effects Model (BREM).

**Results:**

Ten studies were included, stratified by sample type. In the saliva group, *Porphyromonas gingivalis, Tannerella forsythia* and *Prevotella intermedia* demonstrated the highest diagnostic accuracy, with sensitivities reaching 89.2%, 89.2% and 86.5%, and specificities of 94.6%, 86.5% and 83.8%, respectively, achieving AUC values above 0.80. *Porphyromonas gingivalis* was further analysed using BREM, with the Summary Receiver Operating Characteristic (SROC) curve indicating a combined sensitivity and specificity of 84.2% and 85.4%, with an AUC of 0.864. In the subgingival group, biomarkers such as endotoxin activity and combined bacterial biomarkers (5 bacterial species) displayed the highest diagnostic performance, with sensitivities of 90.6% and 85.1% and specificities of 87.9% and 100%, respectively, and AUC values of 0.93 and 0.88.

**Conclusion:**

Microbiome‐derived biomarkers show good clinical utility for improving diagnoses of periodontitis, offering high specificity and sensitivity. Future research should focus on standardising methodologies, increasing sample sizes, and including diverse populations to validate these findings, thereby improving diagnostic precision and facilitating the screening methods for the onset of periodontitis and dysbiotic activity.

## Graphical abstract

Microbiome‐derived biomarkers demonstrate high diagnostic accuracy for periodontitis, with subgingival samples outperforming saliva in clinical utility. This systematic review and meta‐analysis analysed 13 biomarkers from 10 studies, highlighting their diagnostic potential across oral fluids.

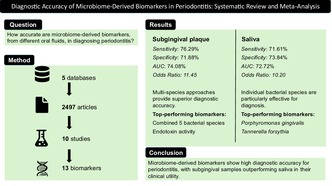

## 1 Introduction

Periodontitis is one of the most prevalent oral diseases globally, affecting over 1 billion people in 2021 alone, with severe cases projected to rise by over 44% by 2050 [1]. Despite advancements in oral health services, the prevalence of periodontitis remains high, underscoring the need for innovative approaches to prevention and early diagnosis [2]. Current oral care heavily relies on restorative treatments, which fail to address the growing burden of preventable diseases. A shift towards point‐of‐care, personalised prevention and novel diagnostic tools is urgently needed to tackle this global challenge.

Conventional methods for diagnosing periodontitis, such as pocket probing depth and radiographic assessments, primarily reflect accumulated damage and lack the sensitivity to detect active microbial or inflammatory challenges [3]. Additionally, these subjective assessments often fail to account for the variability in clinical presentations, highlighting the need for more objective and reliable diagnostic alternatives [4].

Biomarkers have emerged as promising tools in enhancing the diagnosis of periodontitis, by providing molecular‐level insights into microbial dysbiosis and inflammatory dynamics, addressing the diagnostic limitations of traditional methods [5]. Unlike host‐derived biomarkers, which primarily reflect the immunological response to infection [6, 7, 8], microbiome‐derived biomarkers offer direct evidence of the microbial contributions to disease processes. Originating from the dysbiotic oral microbiome, these markers provide a more targeted understanding of the infectious components of periodontal diseases, thereby supporting the development of point‐of‐care diagnostic tools [9, 10].

Whole‐scale changes to the overall microbial population structure and function of the subgingival biofilms and bacterial load are invariably associated with destructive periodontal disease [11]. Microbiome‐derived biomarkers, encompassing both the compositional characteristics of the oral microbiome and specific microbial products [12], are gaining attention in the realm of periodontal diagnostics. The microbial products or so‐called microbe‐associated molecular patterns (MAMPs) could be innate morphological features of the members of the oral microbiome, or they could be expressed as a result of their pathogenic activity. While these biomarkers hold promise, their diagnostic performance, particularly in terms of sensitivity and specificity, remains to be thoroughly explored and updated.

An important consideration in this exploration is the source of the sample, as biomarkers derived from different biological samples—such as saliva, subgingival plaque or gingival crevicular fluid—may offer distinct insights into the periodontal disease status. Specifically, saliva provides a comprehensive overview of oral microbial activity, while subgingival plaque captures site‐specific dysbiosis, and gingival crevicular fluid reflects localised inflammatory responses [13]. The diagnostic performance of these biomarkers can vary significantly based on their origin, reflecting the diverse microbial landscapes and inflammatory profiles within different oral niches [5, 14].

Recognising the transformative potential of microbiome‐derived biomarkers for diagnosing periodontitis and guiding novel diagnostic approaches, the aim of this systematic review was to evaluate the clinical utility and diagnostic accuracy of microbiome‐derived biomarkers by assessing their sensitivity and specificity in periodontitis diagnosis and to explore and to compare the diagnostic performance of these biomarkers from different oral sources (saliva and subgingival biofilm).

## 2 Methods

This systematic review adhered to the guidelines provided by the Cochrane Review Handbook for Systematic Reviews of Diagnostic Tests. It was reported in line with the Preferred Reporting Items for Systematic Reviews and Meta‐Analyses (PRISMA) checklist (Table S1) [15]. The review protocol, encompassing the aim, search strategy, eligibility criteria, data screening, extraction and analyses, was registered with the International Prospective Register of Systematic Reviews (PROSPERO‐CRD42022320040).

### 2.1 PICO Question

For patients diagnosed with periodontitis, how do the diagnostic specificity and sensitivity of microbiome‐derived biomarkers compare to traditional clinical parameters?

### 2.2 Criteria for Considering Studies for This Review

#### 2.2.1 Types of Study

We included studies on single or combinations of microbiome‐derived biomarkers demonstrating sensitivity and specificity based on clinically diagnosed periodontitis. Exclusions were applied to studies lacking binary classification contingency tables or explicit sensitivity and specificity values.

#### 2.2.2 Participants

This review focused on patients with clinical periodontal diagnoses. Studies on animals or in vitro models were excluded.

#### 2.2.3 Index Tests

Considered index tests were any single or combination of biomarkers detected in oral samples analysed for sensitivity and specificity.

#### 2.2.4 Target and Control Conditions

Based on classifications by Armitage [16] and Tonetti, Greenwell, and Kornman [3], target conditions included chronic and aggressive periodontitis and generalised, localised or molar‐incisor pattern periodontitis in addition to disease extent and severity, respectively. Control conditions were patients diagnosed with periodontal health or gingivitis.

#### 2.2.5 Reference Standard

The reference standard was based on clinical (probing pocket depth, bleeding on probing) or radiographic parameters. Any definitions of periodontitis and periodontal health, based on the authors' reported criteria, were accepted. Studies without a detailed reference standard were ineligible.

### 2.3 Search Methods for Identification of Studies

#### 2.3.1 Electronic Searches

The search was conducted from December 2022 to November 2024. We systematically searched electronic databases for studies published between 1981 and November 2024. Our search encompassed PubMed, Nature, Cochrane and OVID, which includes Embase, MEDLINE [R] and PsycINFO. For OVID, we combined MESH terms with text words (Table S2). Additionally, we explored the grey literature through Open Grey, Google and Google Scholar. The literature search was independently executed by the first author (AD), and the results were subsequently consolidated with other authors.

#### 2.3.2 Search Term

Main search terms included: *bacteria, bacteria derived, bacterially, pathogen, periodontitis, periodontal disease, gingivitis, gingival disease, gum disease, detection, detect, diagnosis, biomarker, marker, point‐of‐care, saliva, salivary, GCF, gingival crevicular fluid, plaque, dental biofilm*.

### 2.4 Study Eligibility Assessment

Titles and abstracts were screened by the first author (AD) using the set criteria. Full reports were assessed by two authors (AD and SZ), with discrepancies resolved through a third reviewer.

#### 2.4.1 Inclusion Criteria

- Studies involving human subjects.
- Oral fluid samples: saliva, subgingival plaque and gingival crevicular fluid.
- Defined cases of periodontal disease and periodontitis.
- Bacterial‐derived biomarkers directly related to periodontitis, including microbial counts or products.
- Clinical examinations detailing both pocket probing depth (PPD) and bleeding on probing (BOP) measurements are in line with AAP and EFP guidelines.
- Research methodologies can be quantitative, qualitative or mixed methods, encompassing randomised control trials, observational studies, case–control studies, cross‐sectional and cohort studies.
- Primary research articles.
- Publications in English.

#### 2.4.2 Exclusion Criteria

- Studies without sensitivity and specificity data or without sufficient data to create appropriate matrices for analysis.
- Reviews, systematic reviews, books, case reports and other documents.
- Inappropriate sample sources, such as in vitro samples, cellular models, animal studies, blood samples or non‐oral sources.
- Inappropriate outcomes such as studies focusing on host‐derived biomarkers or lacking quantifiable indicators.
- Studies with aims unrelated to biomarker identification or diagnostic evaluation.
- Studies primarily focused on systemic diseases (e.g. diabetes, cardiovascular diseases) rather than periodontitis or lacking essential clinical records.
- Inappropriate study design, such as longitudinal or long‐term studies and technique comparisons.
- Studies that do not report appropriate sensitivity or specificity data, or lack sufficient matrix data for calculation.
- Unavailability of the full text or publications in languages other than English.

### 2.5 Assessment of Methodological Quality

The methodological quality of selected studies was rigorously assessed using the updated QUADAS‐2 tool as outlined by Whiting et al. [17]. This critical evaluation focused on the domains of patient selection, index test, reference standard and flow and timing to ensure the reliability and validity of the included studies. To enhance the objectivity of this quality assessment, it was independently conducted by the first author, AD, with cross‐validation by a secondary reviewer to resolve discrepancies through consensus. Detailed criteria and scoring rationale are provided in Table S3a.

### 2.6 Subgroup Analyses

The subgroup analyses focused on oral biomarkers derived from different sample sources (saliva or subgingival plaque), enabling a focused comparison of sensitivity and specificity across different sample types, helping to inform optimal biomarker selection for clinical practice.

To further assess the diagnostic accuracy of each biomarker, the Area Under the Curve (AUC) was calculated.

### 2.7 Meta‐Analyses

Meta‐analyses were performed to evaluate pooled sensitivity and specificity, AUC and odds ratios (ORs) for all biomarkers in each subgroup, using random‐effects models under the Restricted Maximum Likelihood (REML) method. For biomarkers evaluated in three or more studies, sensitivity and specificity were systematically aggregated using a Bivariate Random‐Effects Model (BREM), which accounted for potential sources of diagnostic variability, including detection methods, target bacteria, biomarker types, clinical diagnostic criteria and the ratio of cases to controls. Summary Receiver Operating Characteristic (SROC) curves were generated to provide a comprehensive evaluation of their diagnostic performance.

Study homogeneity was assessed using the Q statistic and the *I* ^2^ statistic, calculated with the ‘metafor’ package in R. Based on the *I* ^2^ values, either a Fixed Effects Model (FEM) was chosen for low heterogeneity (*I* ^2^ < 50%) or a Random Effects Model (REM) for significant heterogeneity (*I* ^2^ ≥ 50%) [18].

## 3 Results

### 3.1 Study Selection

**FIGURE 1:**
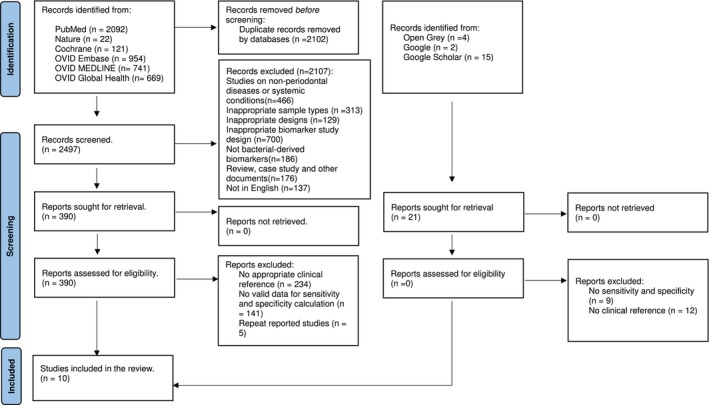
PRISMA 2020. The flowchart summarises the database search and details the sources used, number of hits per source as well as the reasons for exclusion to clarify the process of inclusion of the final 10 studies used in the systematic review.

The search across four databases found 4599 articles, and after removing duplicates, the 2497 abstracts were screened using the Rayyan software. This step led to a review of 390 full‐text articles for eligibility, from which 10 studies met the inclusion criteria for detailed analysis. (Figure 1; Table S4).

### 3.2 Study Characteristics

The ten included studies consisted of six cross‐sectional, three case–controlled and one prospective studies. The sample size ranged from 40 to 217 participants and from 126 to 702 sites. Two of the studies were conducted in the United Kingdom and the United States and one each in Germany, Japan, Slovenia, Turkey, Australia and Finland. The main characteristics of the studies are summarised in Table 1.

**TABLE 1:** Summary of the included studies with number of participants, target conditions and diagnostic criteria.

Author (year)	Country	Type of study	No. of control condition	Control condition	No. of target condition	Target condition	Standard reference	Diagnostic criteria for control condition	Diagnostic criteria for target condition
Arweiler et al. (2020)	Germany	Observational case–control	25 patients	H	100 patients	P	PPD, BOP No calibration	H: PPD 1–3 mm and no BOP	P: at least two pockets with probing depths ≥ 5 mm and BOP
Grundner et al. (2022)	Slovenia	Observational case–control	20 patients	H	20 patients	P	PPD, RBL Calibrated	H: good oral hygiene and no gingival inflammation or systemic disease	P: PPD ≥ 5 mm in at least 4 teeth in four different quadrants and at least 20 teeth (excluding third molars). The diagnosis was supported by radiographic bone loss extending to at least the middle third of the root
Hemmings, Griffiths, and Bulman (1997)	UK	Observational Cross‐sectional	36 sites	H	90 sites	P	PPD, BOP, RBL Calibrated	H: PPD < 3 mm, bleeding score 0, no radiographic bone loss	P: PPD > 5 mm; a colour and bleeding score > 1; radiographic bone loss
Hyva rinen et al. (2009)	Finland	Observational Case–control	81 patients	H	85 patients	P	PPD No calibration	H: PPD < 4 mm	P: at least 14 teeth with probing pocket depth of ≥ 4 mm
Loesche et al. (1990)	USA	Observational Cross‐sectional	252 sites	H	450 sites	P	BOP, PPD, calibrated	H: sites with PPD ≤ 4 mm, no gingival recession and no BOP	P: PPD ≥ 5 mm, BOP
Ma et al. (2021)	Japan	Observational Cross‐sectional	25 patients	H	42 patients	P	PPD No calibration	H: patients with no pocket > 4 mm	P: PPD ≥ 4 mm (in more than 15 sites)
O'Brien‐Simpson et al. (2017)	Australia	Observational cross‐sectional	50 patients	H	50 patients	CP	GI, PD, CAL No calibration	H: no PPD > 3 mm, FMBS < 5%, no radiographic evidence of bone loss	CP: at least 1 pocket ≥ 5 mm, FMBS > 5%, clear radiographic evidence of bone loss
Ramseier et al. (2009)	USA	Observational cross‐sectional study	50 patients	H and G	49 patients	P	RBL, PD, CAL, BOP Calibrated	Healthy and Gingivitis: No radiographic alveolar bone loss, no PPD > 4 mm, CAL < 3 mm.	Periodontitis: at least 4 sites with evidence of radiographic bone loss, CAL > 3 mm and PD > 4 mm
Saygun et al. (2011)	Turkey	Observational cross‐sectional	68 (37 Healthy patients 31 Gingivitis patients)	H and G	82 (46 CP patients 36 (AP patients)	CP and AP	PD, CAL, BOP, No calibration	H: No PPD > 3 mm and no teeth with attachment loss or BOP G: showed several teeth with bleeding on probing but no PPD > 3 mm and no CAL	CP: patients had at least nine posterior teeth with PPD 5–7 mm and three teeth with CAL > 6 mm AP: CAL > 5 mm on more than 14 teeth, with at least three teeth other than incisors or first molars
Zaric et al. (2022)	UK	Prospective cohort study	33 patients	H	32 patients	P	BOP, PPD, RBL, No calibration	H: PPD ≤ 3 mm, no more than 10% BOP and no signs of radiographic bone loss	P: radiographic alveolar bone loss > 15% affecting more than 30% of teeth with PPD ≥ 5 mm and BOP

Abbreviations: AP, Aggressive periodontitis; BOP, Bleeding on probing; CAL, Clinical attachment loss; CP, Chronic periodontitis; G, Gingivitis patients or sites with gingivitis; GI, Gingival index; H, Healthy control subjects or periodontal sites; P, Periodontitis patients or sites with periodontitis; PCP, Progressive chronic periodontitis; PPD, Probing pocket depth; RBL, Radiographic bone loss; SCP, Stable chronic periodontitis.

Six studies focused on the detection of individual bacterial species or their combinations, while four studies utilised bacterial enzymes, products or components as periodontal biomarkers ceramide phosphoethanolamine (CPE), BANA hydrolase (PerioScan) or Endotoxin activity (rFC assays) sourced from subgingival plaque or saliva. The sample types and main findings from these studies are summarised in Table 2.

**TABLE 2:** Summary of the included studies with age of participants, main findings and conclusions.

Author (year)	Age	Sample type (collection methods)	Objective	Key finding	Author's conclusion
Arweiler et al. (2020)	Mean (H): 31.3 ± 11.2 year. Mean (P): 57.4 ± 13.7 year	Subgingival plaque sample (paper point)	To evaluate the diagnostic sensitivity and specificity of a newly developed chair‐side test (CST) for periodontal pathogens against quantitative PCR (qPCR) as the reference method	CST demonstrated “good” sensitivity but lower compared to qPCR and exhibited a high specificity (100%) similar to that of qPCR. This indicates CST's potential utility in identifying key periodontal pathogens, including *Treponema denticola, Tannerella forsythia, Porphyromonas gingivalis, Prevotella intermedia and Aggregatibacter actinomycetemcomitans*	CST is a rapid and effective method for detecting periodontal pathogens, supporting personalised therapy decisions. Despite its qualitative nature and the potential challenge in visual result interpretation, CST's high specificity and good sensitivity make it a valuable tool for diagnosing periodontal disease, particularly relevant for reviews focusing on bacteria‐derived biomarkers
Grundner et al. (2022)	Range (H): 21–25 year Range (P): 25–70 year	Subgingival plaque (by sterile curette)	To investigate the potential of ceramide phosphoethanolamine (CPE) as a diagnostic biomarker for periodontal disease, leveraging the unique presence of CPE in periodontopathogen bacteria	The study discovered that CPE, a lipid not commonly found in vertebrates but prevalent in specific periodontal pathogens, could be specifically detected using aegerolysin‐based assays in clinical samples. This finding indicates the potential of CPE as a biomarker for periodontal disease, providing a novel approach to early diagnosis	The detection of bacterial CPE in periodontal disease patients suggests its utility as a biomarker for early diagnosis and monitoring disease progression. The research highlights the need for further validation and development of CPE detection methods to enhance diagnostic accuracy and facilitate timely treatment interventions
Hemmings, Griffiths, and Bulman (1997)	Range (total): 30–65 year	Subgingival plaque (by sterile curette)	Evaluate the ability of PerioCheck and PerioScan diagnostic tests to detect periodontal disease and monitor response to initial therapy	At baseline, PerioCheck showed a sensitivity of 88% and specificity of 61%, while PerioScan showed a sensitivity of 99% and specificity of 55%. Post‐treatment, the agreement of tests with clinical outcomes was 50.4% for Periocheck and 52% for Perioscan, indicating both tests' moderate alignment with clinical judgements	Despite demonstrating good sensitivity, both diagnostic tests did not reliably reflect the clinical assessment of periodontal disease status or treatment outcomes in this study. The findings underscore the need for further refinement and validation of these tests as adjunctive tools in periodontal diagnosis and management
Hyvärinen et al. (2009)	Mean (H): 47.9 ± 5.7 year Mean (P): 49.6 ± 5.2 year	Whole saliva (the paraffin‐stimulated)	To develop a quantitative real‐time PCR (qPCR) assay based on single copy genes for accurate detection and quantification of five major periodontal pathogens (*Aggregatibacter actinomycetemcomitans, Porphyromonas gingivalis, Prevotella intermedia, Treponema denticola and Tannerella forsythia*)	The developed qPCR assay was sensitive and specific, demonstrating the capability to accurately quantify the presence of the five pathogens in saliva samples. The assay effectively distinguished between subjects with and without periodontitis, showcasing a significant diagnostic potential with high sensitivity and specificity across a wide concentration range of bacterial DNA	The qPCR assay represents a highly sensitive and specific method for detecting major periodontal pathogens, suitable for large‐scale population studies and supporting the diagnosis and treatment of periodontitis. This assay holds significant promise for enhancing periodontal disease management through improved detection and quantification of pathogenic bacteria
Loesche et al. (1990)	Range (total): 23–80 year	Subgingival plaque (by sterile curette)	To assess a chairside diagnostic method's effectiveness, particularly focusing on the BANA hydrolysis test (PerioScan), in identifying specific periodontopathic bacteria such as *Bacteroides gingivalis* and *Treponema denticola* in periodontal disease. This method utilises a solid‐state assay to detect bacterial enzyme activity that hydrolyses BANA, forming a colour reaction	The study demonstrated high sensitivity (92% on average across different centres) and moderate specificity (70% average) in detecting *B. gingivalis* and *T. denticola* in plaque samples using the BANA hydrolysis test. The method was effective in distinguishing between healthy and diseased sites, indicating its potential utility in detecting periodontal pathogens	The BANA hydrolysis test offers a practical, rapid and cost‐effective tool for the detection of key periodontal pathogens, aiding in the diagnosis and management of periodontal disease. It highlights the test's utility in chairside diagnosis due to its simplicity and quick turnaround time, providing valuable information for the detection of periodontal pathogens and supporting treatment decisions
Ma et al. (2021)	Mean (total): 55.4 ± 16.2 year	Whole saliva (the paraffin‐stimulated)	To assess the utility of subgingival plaque‐specific bacteria (SUBP bacteria) in saliva for periodontitis detection, using 16 s ribosomal RNA gene sequencing on saliva samples from 125 subjects to determine the relative abundance of 11 identified SUBP bacteria	SUBP bacteria accounted for 0%–15.4% of salivary microbiota, distinguishing periodontitis patients with a sensitivity of 90% and specificity of 70%. The study suggests examining SUBP bacteria in saliva can be useful for mass screening of periodontitis	The findings underscore the potential of using salivary SUBP bacteria as biomarkers for periodontitis detection, providing a non‐invasive, practical approach for early diagnosis and monitoring, particularly suitable for large‐scale screening efforts
O'Brien‐Simpson et al. (2017)	Mean (H): 52.4 ± 12.1 year (Range: 26–71 year) Mean (CP): 51.2 ± 12.0 year (Range: 28–70 year)	Whole saliva (the paraffin‐stimulated)	Develop a rapid chair‐side saliva‐based detection test for *Porphyromonas gingivalis* using monoclonal antibodies against the A1‐adhesin domain of the *P. gingivalis* RgpA‐Kgp proteinase‐adhesin complex	The immunochromatographic device showed high sensitivity (95.0%), specificity (93.3%), positive predictive value (90.5%) and accuracy (94.0%) in detecting *P. gingivalis* in saliva, correlating strongly with its levels in subgingival plaque and clinical parameters of disease	The developed chair‐side test provides a highly sensitive and specific method for detecting *P. gingivalis* , supporting its use as a practical tool for early intervention and management of periodontal disease
Ramseier et al. (2009)	Mean (H) = 45 year Mean (G) = 42 year Mean (CP) = 53 year	Subgingival plaque (by sterile curette)	Explore host‐response salivary biomarkers and microbial biofilm DNA to identify different stages of periodontal disease, employing qPCR and sensitive immunoassays for validation	Elevated levels of MMP‐8 and −9, and the presence of specific anaerobic pathogens like *Porphyromonas gingivalis* and *Treponema denticola* , were significantly associated with periodontal disease, offering robust predictive values for disease identification	The combination of specific host‐response markers and pathogen levels provides a promising basis for developing rapid point‐of‐care diagnostics for periodontal and potentially other systemic diseases, highlighting the importance of multi‐marker approaches for accurate disease status assessment
Saygun et al. (2011)	Mean (H): 33.1 ± 6.7 year Mean (G): 30.9 ± 8.4 year Mean (CP): 42.7 ± 8.2 year Mean (AP): 34.5 ± 7.3 year	Whole saliva (unstimulated)	Examine if salivary counts of periodontopathic bacteria correlate with periodontal pocket counts and distinguish between periodontal health and disease	Higher salivary counts of specific bacteria, including *Campylobacter rectus, Fusobacterium nucleatum, Porphyromonas gingivalis, Prevotella intermedia and Tannerella forsythia*, were significantly associated with periodontal disease. The study identified salivary thresholds for these bacteria that could predict periodontitis with high sensitivity and specificity	Salivary detection of key periodontopathic bacteria has potential for periodontitis diagnosis, indicating that salivary diagnostics can aid in identifying periodontal disease status and support the need for further research on their predictive value for disease progression
Zaric et al. (2022)	Mean (H): 31 ± 9 year Mean (P): 47 ± 8 year	Whole saliva (unstimulated) and subgingival plaque samples (paper point)	Explore the diagnostic and prognostic utility of oral lipopolysaccharides (LPS) as bacterially derived periodontal biomarker, focusing on the association between clinical periodontal parameters and subgingival/salivary endotoxin activities	Subgingival endotoxin activity displayed high specificity and sensitivity (0.91 and 0.85 respectively) for identifying periodontal health and disease. Salivary endotoxin activity, while positively associated with periodontal diagnosis, had lower sensitivity and specificity (0.69 and 0.61) compared to subgingival endotoxin activity	Subgingival endotoxin activity is a valuable site‐specific periodontal biomarker with significant diagnostic and prognostic capabilities, unaffected by patient demographics. Salivary endotoxin activity, although it shows good correlation with disease extent and severity, has lower diagnostic and prognostic value

### 3.3 Quality Assessment of Selected Studies

**FIGURE 2:**
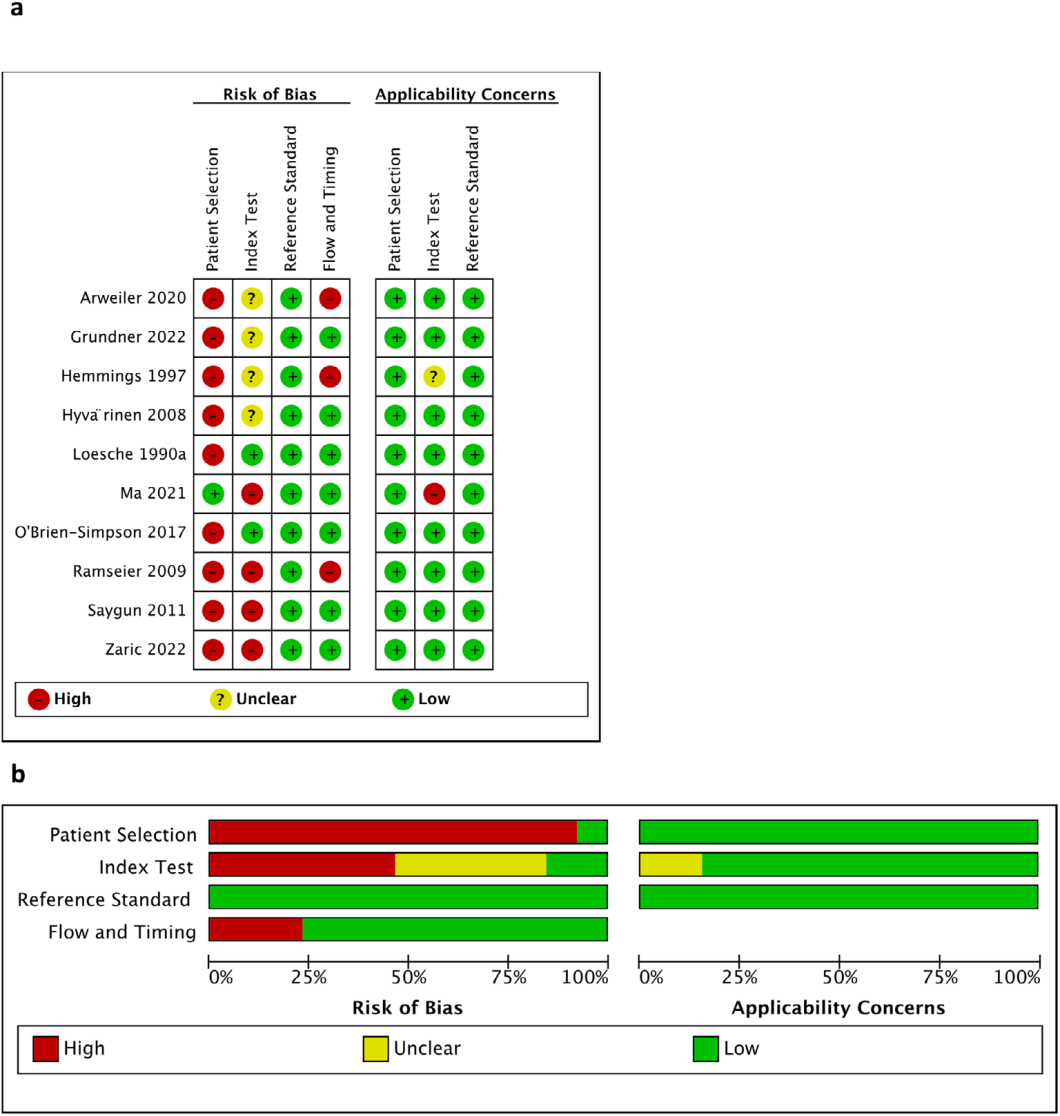
(a) Risk of bias and applicability concerns evaluation for the reviewed studies (PRISMA Framework). (b) Summary of risk of bias and applicability issues (PRISMA).

The quality of the selected studies was assessed using the modified QUADAS‐2 tool [17] (Figure 2a,b).

Except for one study [19], nine studies utilised non‐random, convenience sampling methods for recruiting participants. Consequently, the risk of patient selection bias was recorded as “high” for these studies. In terms of the index tests, the risk of bias was often marked as “unclear” because the reports did not mention the blinding protocols or the interpretation or measurement protocols for the reference tests were not explained. [20, 21, 22, 23]. This absence of detailed methodological transparency potentially compromises the reliability of the index test outcomes across these studies. Furthermore, the risk for most studies was considered “high” because examinations/assessments were conducted before the collection of samples, introducing potential pre‐analytical variability that could influence the test results.(Table S3b).

All studies exhibited a low risk of bias with regard to the reference standard, applying clear and correct clinical diagnostic criteria consistently across the board. This uniformity ensures the reliability of the diagnostic outcomes. However, in terms of flow and timing, Hemmings, Griffiths, and Bulman [22], Ramseier et al. [24] and Arweiler et al. [20] studies demonstrated higher risks due to not including all participants in the final analyses, which could potentially compromise the generalizability and accuracy of their findings. This highlights the importance of maintaining consistent participant tracking and analyses to uphold the integrity of study results.

### 3.4 Subgroup Analyses

#### 3.4.1 Salivary Biomarkers

##### 3.4.1.1 Diagnostic Accuracy Analyses of Salivary Biomarkers

**FIGURE 3:**
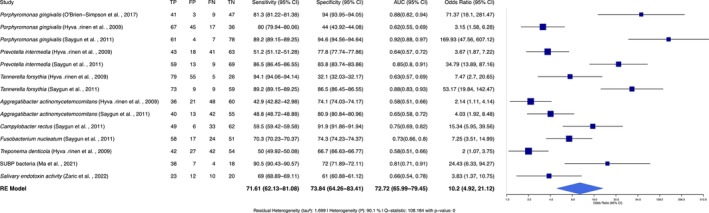
Forest plot showing diagnostic accuracy of salivary biomarkers for periodontitis. Diagnostic accuracy metrics (sensitivity, specificity, AUC and odds ratio) of salivary biomarkers for periodontitis. The odds ratio (OR) indicates the strength of association between biomarkers and periodontitis. A random‐effects model was applied to account for study heterogeneity. FN = false negatives, FP = false positives, TN = true negatives, TP = true positives.

In the evaluation of salivary biomarkers for the diagnostic accuracy for periodontitis, nine biomarkers were identified, with sensitivity across the studies ranging from 43% to 94%, while specificity varied from 32% to 95% (Figure 3).

*Porphyromonas gingivalis* demonstrated the highest diagnostic performance, with Saygun et al. [25] reporting a sensitivity and specificity of 89.2% and 94.6%, respectively, and an AUC of 0.92, with an OR of 169.93 (95% CI: 41.56–607.12). *Prevotella intermedia* achieved the sensitivity and specificity of up to 86.5% and 83.8% [25], with an AUC of 0.85. The calculated OR of *Prevotella intermedia* was 34.79 (95% CI: 13.89–87.16). For *Tannerella forsythia* , Saygun et al. [25] reported a sensitivity of 89.2% and specificity of 86.5% (AUC = 0.88), with an estimated OR of 53.17 (95% CI: 19.84–142.47). *Aggregatibacter actinomycetemcomitans* reported sensitivity was 48.8%, and specificity of 80.9%, with an AUC of 0.65 and an OR of 4.03 (95% CI: 1.92–8.48) [25]. The detailed metrics are provided in Table S5a.

Zaric et al. [26] evaluated salivary endotoxin activity as a biomarker for periodontitis, reporting a sensitivity of 69% and a specificity of 61%, with a moderate AUC of 0.66, and an OR of 3.83 (95% CI: 1.37–10.75). The combined detection of multiple pathogens by Ma et al. [19] on SUBP bacteria yielded a sensitivity of 90.5% and specificity of 72%, with an AUC of 0.91, with an OR of 24.43 (95% CI: 6.33–94.27).

##### 3.4.1.2 Homogeneity and Heterogeneity Assessment

The meta‐analysis of the salivary group included nine biomarkers, with the REML analysis revealing substantial heterogeneity among the included studies (*I* ^2^ = 90.1%, Q (*df* = 15) = 108.18, *p* < 0.0001). This significant variability in diagnostic performance across studies necessitated the use of a random‐effects model (REM) to address the heterogeneity. A detailed summary of the included studies is provided in Table S6a.

The REM analysis revealed an overall odds ratio (OR) of 10.2 (95% CI: 4.92–21.12, *p* < 0.0001), with a pooled sensitivity of 71.61%, pooled specificity of 73.84%, and a pooled AUC of 72.72%, indicating a strong association between these salivary biomarkers and periodontitis. The estimated residual heterogeneity (*τ* ^2^) was 1.699, underscoring the diversity in effect sizes across studies.

##### 3.4.1.3 Summary Receiver Operating Characteristics Curve Analyses for Selected Salivary Biomarkers

**FIGURE 4:**
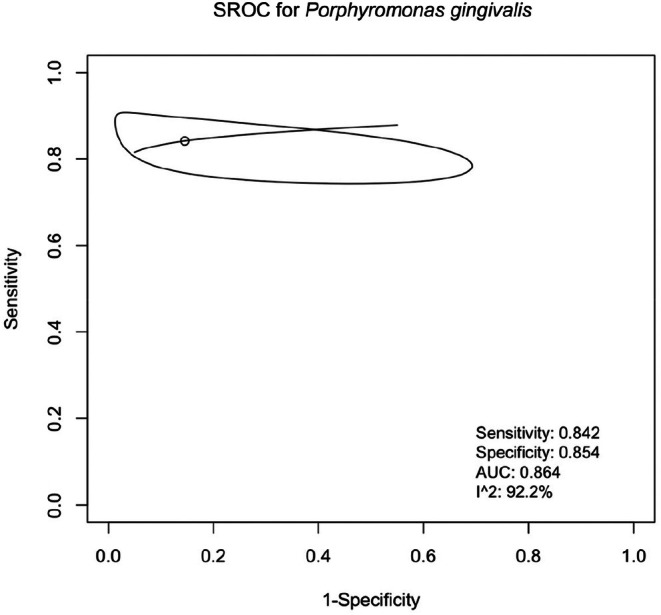
Bivariate random‐effects meta‐analysis of SROC curves for *Porphyromonas gingivalis* in the diagnosis of periodontitis.

Only one salivary biomarker *(P. gingivalis)* had enough data for the SROC analysis (Figure 4). For *Porphyromonas gingivalis* , the SROC curve revealed an estimated sensitivity of 84.2% and specificity of 85.4%, with an area under the curve (AUC) of 0.864. However, the *I* ^2^ value of 92.2% reflects high heterogeneity.

#### 3.4.2 Subgingival Biomarkers

##### 3.4.2.1 Diagnostic Accuracy Analyses of Subgingival Biomarkers

**FIGURE 5:**
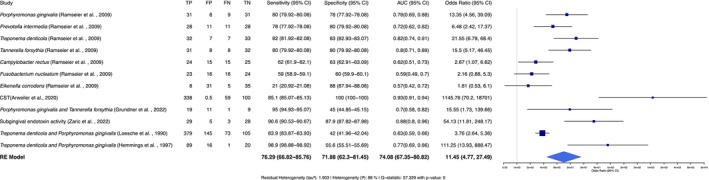
Forest plot showing diagnostic accuracy of subgingival biomarkers for periodontitis. Diagnostic accuracy metrics (sensitivity, specificity, AUC and odds ratio) of salivary biomarkers for periodontitis. The odds ratio (OR) indicates the strength of association between biomarkers and periodontitis. A random‐effects model was applied to account for the studies heterogeneity. FN = false negatives, FP = false positives, TN = true negatives, TP = true positives.

In the evaluation of subgingival biomarkers for diagnosis of periodontitis, the analysis revealed that sensitivity across the studies ranged from 21% to 98%, while specificity varied from 41.9% to 100% (Figure 5). For *Porphyromonas gingivalis* , Ramseier et al. [24] reported a sensitivity of 80% and specificity of 78%, with an AUC of 0.78 and an OR of 9.21 (95% CI: 3.45–24.63). *Prevotella intermedia* detection showed sensitivity and specificity up to 72.0% and 80.0% [24], with an AUC of 0.70 and an OR of 6.75 (95% CI: 2.48–18.36). Detection of *Treponema denticola* showed good diagnostic performance, with Ramseier et al. [24] reporting sensitivity and specificity up to 82.0% and 83.0%, along with an AUC of 0.82. Its OR was estimated at 8.34 (95% CI: 3.12–22.23).

The combined detection of multiple pathogens by Arweiler et al. [20] using a chair‐side device (CST) yielded the highest diagnostic performance, achieving a sensitivity of 85% and specificity of 100%, with an AUC of 0.93, and an OR of 1145.76 (95% CI: 702.0–18 701), highlighting its potential as a rapid diagnostic tool. Zaric et al. [26] evaluated subgingival endotoxin activity (using rFC assays) as a periodontal biomarker and reported sensitivity and specificity of 90.6% and 87.9%, respectively, with an AUC of 0.88 and an OR of 54.13 (95% CI: 11.81–248.17). The trypsin‐like proteolytic activity in subgingival samples, assessed by Loesche et al. [27] and Hemmings, Griffiths, and Bulman [22] through BANA assays, showed sensitivities of 84% and 98.9%, and specificities of 42% and 55.6%, respectively, with AUCs of 0.63 to 0.77. ORs for these studies were calculated as 2.45 (95% CI: 0.87–6.92) and 3.12 (95% CI: 1.02–9.63), suggesting limited diagnostic utility. The detailed performance metrics are summarised in the Table S5b.

##### 3.4.2.2 Homogeneity and Heterogeneity Assessment

The meta‐analysis examined 11 subgingival biomarkers, with the REML analysis indicating considerable heterogeneity among the studies (*I* ^2^ = 88%, Q (*df* = 11) = 57.329, *p* < 0.0001). Due to this substantial variability in diagnostic performance across studies, a random‐effects model (REM) was employed to accommodate for the heterogeneity. The comprehensive summary of the included studies is detailed in Table S6b.

The REM analysis highlighted a significant total heterogeneity (*τ* ^2^ = 1.903), suggesting that the differences between studies were pronounced. The overall effect size from the REM yielded an odds ratio (OR) of 11.45 (95% CI: 4.77–27.49, *p* < 0.0001), indicating a strong association between the subgingival biomarkers and periodontitis. Pooled diagnostic metrics showed a sensitivity of 76.29%, a specificity of 71.88%, and an AUC of 74.08%.

None of the biomarkers met the criteria for further analysis using a Bivariate Random‐Effects Model (BREM) due to a lack of significant ORs across more than three studies, which is necessary to ensure statistical power and accuracy in subgroup analyses.

## 4 Discussion

This systematic review explores the diagnostic potential of salivary and subgingival biomarkers for periodontitis, identifying a total of 13 bacterially derived biomarkers across 10 studies. This is the first review to assess the diagnostic accuracy of both salivary and subgingival biomarkers with a focus on sensitivity and specificity through subgroup analyses by sample source. This approach allows for a more intricate understanding of biomarker utility across sample types, which could support the development of more targeted diagnostic strategies for periodontitis. By addressing this previously unexplored area, our work aims to fill a significant gap in the current literature, highlighting the critical importance of microbiome‐derived biomarkers in advancing periodontitis diagnostics.

A recent systematic review on diagnostic sensitivity and specificity of host‐derived biomarkers [7], assessing only saliva as a sampling source, concluded that certain salivary biomarkers (IL‐6, MMP‐8, IL1‐beta) can potentially be useful alone or in combination, for the diagnosis of periodontitis. However, further methodically robust research was suggested in order to validate these biomarkers. Similarly, [28] reported that in saliva, the dual combinations of IL‐1β, IL‐6 and MMP‐8 have an excellent ability to detect periodontitis and a good capacity to detect non‐periodontitis, but a meta‐analysis of gingival crevicular fluid biomarkers was not possible. Chew et al. [29] also highlighted the role of microbial biomarkers as potential predictors in periodontal treatment response, specifically for active and supportive periodontal therapy outcomes, though their study noted variability in the prognostic accuracy across different microbial species and treatment contexts.

### 4.1 Biomarker Evidence

In this systematic review, the diagnostic potential of several microbiome‐derived biomarkers for periodontitis was highlighted, primarily focusing on red‐complex pathogens such as *Porphyromonas gingivalis* , *Prevotella intermedia* and *Tannerella forsythia* . These species exhibited high sensitivity and specificity across both saliva and subgingival samples, though the analysis is limited to targeted approaches that may not fully capture the breadth of potential biomarkers [30]. Notably, *Porphyromonas gingivalis* performed consistently well as a versatile biomarker differentiating between healthy and periodontitis, particularly in studies employing targeted detection methods such as monoclonal antibody‐based diagnostics and qPCR assays. While these targeted approaches offer high diagnostic accuracy, future research utilising untargeted sequencing methods could uncover additional microbial biomarkers.

In subgingival samples, multi‐pathogen approaches like the Chairside Test (CST) showed the highest diagnostic accuracy, effectively leveraging the complex microbial environment [20]. Bacterial products, endotoxin activity and BANA assays, also performed well, likely because they capture broader pathogenic activity rather than specific bacteria [22, 26]. However, single pathogens [24] showed comparatively lower diagnostic performance, highlighting the limitations of single species in diverse subgingival communities. These results underscore the value of combined profiles and bacterial products over single bacteria for accurate periodontal diagnosis. In salivary samples, monoclonal antibodies targeting *Porphyromonas gingivalis* used in an immunochromatographic device represent a significant advancement in diagnostics, demonstrating notable diagnostic performance [31]. Unlike in subgingival samples, where multi‐pathogen and product‐based markers perform well, salivary diagnostics are more effective with specificity to individual pathogens [19]. *P. gingivalis* , *P. intermedia* and *T. forsythia* exhibit strong diagnostic performance in saliva, supporting their use as reliable indicators for periodontal disease. In contrast, salivary endotoxin measurements have shown lower diagnostic value, likely due to reduced microbial complexity in saliva compared to subgingival samples [26].

### 4.2 Strength of Evidence and Study Design Considerations

Our initial broad search strategy led to the retrieval of a large number of articles, many of which were subsequently excluded due to insufficient data for evaluating sensitivity and specificity. This comprehensive approach, while thorough, introduced challenges related to variability among the studies. Furthermore, our lenient inclusion criteria regarding publication dates resulted in the inclusion of studies using different diagnostic criteria for periodontitis, reflecting changes in standards over time, such as those from 1999 versus 2017 [3, 16]. To address these issues, rigorous methods were employed to assess and manage heterogeneity. The REML method was applied to aggregate variables and control for inter‐study differences. Subgroup analyses and random‐effects meta‐analyses further facilitated the examination of both within‐group and between‐group homogeneity and heterogeneity, enabling a more specific exploration of the diagnostic potential of microbiome‐derived biomarkers [18, 32].

The majority of the studies included in this review, were case–control designs. This design is common in biomarker and diagnostic studies but comes with higher risks of bias compared to randomised controlled trials (RCTs). Case–control studies are invaluable for exploring associations and outcomes where RCTs may not be feasible due to ethical constraints. However, they are prone to selection bias, information bias and confounding, which can distort the association between exposure and outcome. Prospective cohort studies, on the other hand, offer a more robust design for studying risk factors and outcomes over time, providing valuable insights into disease progression and the impact of interventions. Despite their advantages, these studies require meticulous planning and adequate sample sizes, particularly for rare diseases or long‐term outcomes [33].

### 4.3 Index Test Variation and Reference Test Variation

The variability in index tests significantly increases the heterogeneity between studies, impacting the final analyses. In the selected studies, different detection techniques were used, including both qPCR and 16S rRNA sequencing for assessing biomarkers [19, 24]. Variations in primer selection and amplification protocols led to inconsistent thresholds, which in turn impacted the assessment of sensitivity and specificity. Although an attempt was made to analyse these varying thresholds, the limited data available precluded a meaningful exploration of their effects.

Unlike other reviews that primarily focus on saliva, our review includes studies using saliva and subgingival samples. This cross‐sample analysis introduces additional risks when comparing studies, including variations in sample collection, handling and storage processes. In saliva collection, Zaric et al. [26] and Saygun et al. [25] used unstimulated whole saliva samples, while other studies employed paraffin‐stimulated methods. These methodological differences add to the variability in our analysis. Similarly, in subgingival sampling, Arweiler et al. [20] and Zaric et al. [26] used paper points, whereas the other studies utilised curettes.

Regarding reference test variation, the heterogeneity in case definitions also contributed to variability. While probing depth (PD) and bleeding on probing (BOP) are common diagnostic criteria for healthy and periodontal disease states, the PD thresholds varied. O'Brien‐Simpson et al. [31] and Saygun et al. [25] used a PD ≤ 3 mm to define the healthy group, while other studies used 4 mm as the threshold between periodontitis and control.

### 4.4 Effects of Confounders

The influence of smoking, a known confounder in periodontal research, was variably reported. Hyvärinen et al. [23] noted smoking habits in both control and target groups, while O'Brien‐Simpson et al. [31] exclusively studied non‐smokers. The masking effect of smoking on gingival inflammation and bleeding can potentially skew results if not adequately controlled [34, 35].

The age of the participants, another potential confounder, varied significantly across studies. For example, the age range in Hemmings, Griffiths, and Bulman [22] was 30–65 years, while in Ma et al. [19], it spanned from 22 to 91 years.

Similarly, the collection methods, storage and analyses varied across studies too. For instance, while some studies like Loesche et al. [27] stored samples at −10°C, others like Ma et al. [19] and Zaric et al. [26] stored them at −80°C. These variations can introduce discrepancies in biomarker analysis.

In conclusion, while these studies provide valuable insights into diagnostic biomarkers for periodontitis, variability in confounding factors like smoking, age and sample handling methods necessitates cautious interpretation. Future research should prioritise standardised methodologies and rigorous confounder control to enhance reliability and comparability across studies.

### 4.5 Limitations

This review has some limitations that must be acknowledged. A primary limitation lies in the variability among the included studies in terms of study design and sample handling protocols. Variability in sample types, collection methods and processing protocols likely contributed to inconsistencies in diagnostic accuracy, complicating the synthesis and interpretation of pooled estimates. Furthermore, while this review accounted for confounders such as smoking and age, other systemic conditions, including diabetes, were not consistently controlled across studies. To address the potential impact of confounders, a random‐effects model (REM) was employed in the analysis to account for variability across studies and accommodate overall heterogeneity. However, the limitation remains that no subgroup analyses were performed to specifically evaluate these confounders, such as stratifications by age, smoking status or diabetes.

Another limitation is the variability in detection methods and thresholds across studies. Techniques such as qPCR, monoclonal antibody assays, and sequencing often use differing thresholds and units, affecting biomarker performance metrics. Although these thresholds and units were incorporated into subgroup analyses (Tables S5a and S5b), the lack of standardisation introduces bias and limits comparability. Future research should adopt consistent diagnostic protocols to enhance reproducibility.

The reliance on targeted strategies in most studies, focusing on well‐known periodontal pathobionts, may overlook less known microbial markers or novel pathways with diagnostic and prognostic potential. Future research should employ untargeted, high‐throughput approaches, such as metagenomics and metabolomics, to identify novel biomarkers and better characterise microbial dysbiosis in periodontitis.

In conclusion, there is a need for larger, well‐designed studies with standardised protocols, comprehensive data collection and advanced statistical methods to enhance the reliability and validity of future research in periodontal disease diagnostics. Collaborative efforts and data sharing among research institutions will also facilitate more extensive and cost‐effective studies, ultimately advancing the field.

### 4.6 Clinical Significance

The clinical significance of microbiome‐derived biomarkers in periodontitis diagnostics is highlighted by their potential to offer more specific, personalised and early detection compared to traditional diagnostic techniques. Unlike host‐derived markers, which reflect the body's general inflammatory response and can be influenced by a variety of conditions, microbiome‐derived biomarkers such as *Porphyromonas gingivalis* , *Prevotella intermedia* and *Tannerella forsythia* directly target the pathobionts involved in the pathogenesis of periodontitis. Microbiome‐derived biomarkers allow for the detection of periodontal pathogens before significant tissue damage and clinical symptoms occur, facilitating earlier intervention and potentially halting disease progression [36]. This capability is particularly advantageous over host‐derived biomarkers, which may only indicate an inflammatory response once significant tissue damage has already taken place [37]. By enabling earlier and more precise detection, microbiome‐derived biomarkers support the development of personalised treatment strategies [38, 39]. Clinicians can tailor interventions to the specific bacterial profiles identified, enhancing treatment efficacy and improving patient outcomes [40]. This personalised approach is a significant advancement over traditional methods, which often rely on broader and less specific indicators of periodontal health.

## 5 Conclusion

### 5.1 Implications for Clinical Practice

This systematic review highlights the diagnostic accuracy of microbiome‐derived biomarkers, such as *Porphyromonas gingivalis* , *Prevotella intermedia* and *Tannerella forsythia* , in distinguishing periodontitis from health and gingivitis, with high sensitivity and specificity across sample types like saliva and subgingival plaque. Single biomarkers, such as *P. gingivalis* detected by qPCR or endotoxin activity as a general dysbiosis marker, have shown promise as effective diagnostic tools, while point‐of‐care tests like the Chairside Test (CST) that combines multiple bacterial profiles provide a practical approach for periodontitis detection and risk stratification in clinical settings [20, 23].

These biomarkers could enhance periodontitis screening by offering clinicians more precise tools to monitor microbial activity and stratify patients based on their risk profiles. While the focus of this review is on diagnostic accuracy, the ability of certain biomarkers to reflect disease progression highlights their potential role in dynamic monitoring, particularly in tracking shifts from gingivitis to periodontitis.

Further exploration of novel biomarkers and multi‐omic approaches could expand the diagnostic capabilities of microbiome‐derived tools, enabling a more comprehensive understanding of microbial dysbiosis in periodontitis.

### 5.2 Implications for Research

Future research should prioritise the standardisation of detection methodologies for microbiome‐derived biomarkers and focus on validating these findings in larger, more diverse cohorts. Whilst the targeted detection of specific bacterial species has shown promising diagnostic accuracy, untargeted metagenomic approaches may offer broader insights into microbial communities and their roles in the disease development. Additionally, further investigation of microbial products, such as endotoxins and other virulence factors, is necessary to fully elucidate their contributions to periodontal pathogenesis. Given the diagnostic potential seen in both saliva and subgingival samples, future studies could refine biomarker selection based on the strengths of each sample type. Such research could lead to the development of novel diagnostic markers and therapeutic targets, enhancing the precision and effectiveness of periodontitis management.

## Conflicts of Interest

The authors declare no conflicts of interest.

## Supporting information

Data S1.

Table S2.

Table S3a.

Table S3b.

Table S4.

Table S5a.

Table S5b.

Table S6a.

Table S6b.

## Data Availability

The data that supports the findings of this study are available in the supporting information of this article.
