# Supplementary material for: Diagnostic Accuracy of Microbiome‐Derived Biomarkers in Periodontitis: Systematic Review and Meta‐Analysis
Source: J Periodontal Res. 2025 Jan 13;60(8):748–61. doi: 10.1111/jre.13377 (PMC12476084; doi:10.1111/jre.13377)
Supplement: Supplementary file 2 — Table S2. [file JRE-60-748-s001.docx]

Table S2. Search strategy, which was applied in the different databases.

| - **Terms for target condition** |
| --- |
| 1. periodontitis 2. periodontal disease 3. gingivitis 4. gingival disease 5. gum disease |
| - **Terms for the type of oral sample analysed** |
| 1. Saliva 2. Salivary 3. GCF 4. gingival crevicular fluid 5. plaque 6. dental biofilm |
| - **Terms for the index tests** |
| 1. Bacteria 2. bacteria derived 3. bacterially derived 4. pathogen 5. detection 6. detect 7. diagnosis 8. biomarker 9. marker 10. point-of-care |
| (1 or 2 or 3 or 4 or 5) and (6 or 7 or 8 or 9 or 10 or 11) and (12 or 13 or 14 or 15) and (16 or 17 or 18 or 19 or 20 or 21) |
