# Supplementary material for: Diagnostic Accuracy of Microbiome‐Derived Biomarkers in Periodontitis: Systematic Review and Meta‐Analysis
Source: J Periodontal Res. 2025 Jan 13;60(8):748–61. doi: 10.1111/jre.13377 (PMC12476084; doi:10.1111/jre.13377)
Supplement: Supplementary file 3 — Table S3a. [file JRE-60-748-s004.docx]

***Table S3a. QUADAS-2 tool modified according to the characteristics of the included diagnostic accuracy studies.***

| **QUADAS2: METHODOLOGICAL QUALITY OF THE DIAGNOSTIC ACCURACY STUDIES** | | | |
| --- | --- | --- | --- |
| **Item** | **Authors’ judgement**  (yes, no,  or unclear) | **Risk of bias**  (high,  low, or unclear) | **Applicability concerns**  (high, low, or  unclear) |
| **DOMAIN 1. PATIENT SELECTION** | | | |
| 1.1 Was a consecutive or random sample of  patients enrolled? |  |  |  |
| 1.2 Was a case–control design avoided? |  |  |  |
| 1.3 Did the study avoid inappropriate exclusions? |  |  |  |
| **1.4 Could the selection of patients have introduced bias?** |  |  |  |
| **1.5 Are there concerns that the included patients do not match the review question?** |  |  |  |
| **DOMAIN 2. INDEX TEST** | | | |
| 2.1 Were the index test results interpreted without knowledge of the results of the reference standard? |  |  |  |
| 2.2 If a threshold was used, was it prespecified? |  |  |  |
| **2.3 Could the conduct or interpretation of the index test have introduced bias?** |  |  |  |
| **2.4 Are there concerns that the index test, its conduct, or interpretation differ from the review question?** |  |  |  |
| **DOMAIN 3. REFERENCE STANDARD** | | | |
| 3.1 Is the reference standard likely to correctly  classify the target condition? |  |  |  |
| 3.2 Were the reference standard results  interpreted without knowledge of the results  of the index test? |  |  |  |
| **3.3 Could the reference standard, its conduct, or**  **its interpretation have introduced bias?** |  |  |  |
| **3.4 Are there concerns that the target condition as defined by the reference standard does not match the review question?** |  |  |  |
| **DOMAIN 4. FLOW AND TIMING** | | | |
| 4.1 Was there an appropriate interval between index tests and reference standard? |  |  |  |
| 4.2 Did all patients receive the same reference standard? |  |  |  |
| 4.3 Were all patients or subgingival sites included in the analysis? |  |  |  |
| **4.4 Could the patient flow have introduced bias?** |  |  |  |
