# Supplementary material for: Diagnostic Accuracy of Microbiome‐Derived Biomarkers in Periodontitis: Systematic Review and Meta‐Analysis
Source: J Periodontal Res. 2025 Jan 13;60(8):748–61. doi: 10.1111/jre.13377 (PMC12476084; doi:10.1111/jre.13377)
Supplement: Supplementary file 4 — Table S3b. [file JRE-60-748-s006.docx]

Table S3b. Results obtained at the item level in the 10 articles included applying the modified QUADAS-2 tool.

|  |  | **Items** | | | | | | | | | | | | | | | |
| --- | --- | --- | --- | --- | --- | --- | --- | --- | --- | --- | --- | --- | --- | --- | --- | --- | --- |
|  | **Domain 1** | | | | | **Domain 2** | | | | **Domain 3** | | | | **Domain 4** | | | |
|  | 1.1 | 1.2 | 1.3 | **1.4** | **1.5** | 2.1 | **2.2** | **2.3** | **2.4** | 3.1 | 3.2 | **3.3** | **3.4** | 4.1 | 4.2 | 4.3 | **4.4** |
| Arweiler 2020 | Y | N | Y | **H** | **L** | U | **Y** | **U** | **L** | Y | Y | **L** | **L** | Y | Y | N | **H** |
| Grundner 2022 | N | N | Y | **H** | **L** | U | **Y** | **U** | **L** | Y | Y | **L** | **L** | Y | Y | Y | **L** |
| Hemmings 1997 | Y | N | Y | **H** | **L** | U | **U** | **U** | **U** | Y | Y | **L** | **L** | Y | Y | N | **H** |
| Hyva ̈rinen 2008 | Y | N | U | **H** | **L** | U | **Y** | **U** | **L** | Y | Y | **L** | **L** | Y | Y | Y | **L** |
| Loesche 1990 | Y | N | Y | **H** | **L** | Y | **Y** | **L** | **L** | Y | Y | **L** | **L** | Y | Y | Y | **L** |
| Ma 2021 | Y | N | Y | **H** | **L** | U | **N** | **H** | **L** | Y | Y | **L** | **L** | Y | Y | Y | **L** |
| O'Brien-Simpson 2017 | Y | N | Y | **H** | **L** | Y | **Y** | **L** | **L** | Y | Y | **L** | **L** | Y | Y | Y | **L** |
| Ramseier et al. 2009 | Y | N | Y | **H** | **L** | U | **N** | **H** | **L** | Y | Y | **L** | **L** | Y | Y | N | **H** |
| Saygun 2011 | Y | N | Y | **H** | **L** | U | **N** | **H** | **L** | Y | Y | **L** | **L** | Y | Y | Y | **L** |
| Zaric 2022 | Y | N | Y | **H** | **L** | U | **N** | **H** | **L** | Y | Y | **L** | **L** | Y | Y | Y | **L** |

Yes (Y), no (N), unclear (U). Risk: LOW (L) /HIGH (H) /UNCLEAR (U).
