# Supplementary material for: Diagnostic Accuracy of Microbiome‐Derived Biomarkers in Periodontitis: Systematic Review and Meta‐Analysis
Source: J Periodontal Res. 2025 Jan 13;60(8):748–61. doi: 10.1111/jre.13377 (PMC12476084; doi:10.1111/jre.13377)
Supplement: Supplementary file 5 — Table S4. [file JRE-60-748-s005.docx]

**Table S4. Excluded articles and reasons for exclusion**

1. Reviews, systematic reviews, books, case reports, and other documents (n=176)
2. Inappropriate sample types, such as in vitro models, cellular studies, animal models, blood samples, or sources outside oral fluids (n=313)
3. Irrelevant outcomes, including studies focused on non-bacterial-derived biomarkers, such as host-derived biomarkers (n=186)
4. Inappropriate target or reference groups, including studies focused on other diseases or systemic conditions (n=466)
5. Inappropriate study design, including longitudinal studies, long-term analyses, or technique comparisons (n=129)
6. Studies with objectives unrelated to biomarker identification or diagnostic evaluation, such as those focused on treatment approaches or other non-diagnostic aims (n=700)
7. Publications in languages other than English (n=137)
8. Studies that do not report appropriate sensitivity or specificity data, or lack sufficient matrix data for calculation (n=141)
9. Studies without a focus on distinguishing healthy from periodontal disease groups (n=234)
10. Duplicate reports of the same study (n=5)

| Title | Authors | Notes |
| --- | --- | --- |
| Risk factors involved in coronary heart disease in relation with oral hygiene | Abijeth B. and Priya J. | 1 |
| Presence of Helicobacter pylori in subgingival plaque of periodontitis patients with and without dyspepsia, detected by polymerase chain reaction and culture. | Agarwal, Sangita and Jithendra, K D | 1 |
| Role of Antimicrobial Peptides in Periodontitis | Al-Daragi, F. Z. and Al-Ghurabi, B. H. | 1 |
| The benefit of culture-independent methods to detect bacteria and fungi in re-infected root filled teeth: a pilot study | Al-Sakati H. and Kowollik S. and Gabris S. and Balasiu A. and Ommerborn M. and Pfeffer K. and Henrich B. and Raab W.H.-M. AO - Al-Sakati H.; ORCID: https://orcid.org/0000-0002-8471-1488 | 1 |
| Potential Impact of Prosthetic Biomaterials on the Periodontium: A Comprehensive Review | Alarcon-Sanchez M.A. and Heboyan A. and Fernandes G.V.O. and Castro-Alarcon N. and Romero-Castro N.S. AO - Alarcon-Sanchez, Mario Alberto; ORCID: https://orcid.org/0000-0001-6... | 1 |
| Application of 16s RRNA in identifying oral microflora - a review of literature. | Amrita Geevarghese, Amrita Geevarghese and Baskaradoss, J. K. and Al-Dosari, A. A. | 1 |
| Antibiotic resistance in periodontitis patients: A systematic scoping review of randomized clinical trials | Ardila C.-M. and Bedoya-Garcia J.-A. and Arrubla-Escobar D.-E. AO - Ardila, Carlos-M.; ORCID: https://orcid.org/0000-0002-3663-1416 | 1 |
| Peri-implantitis: Associated microbiota and treatment | Ata-Ali J. and Candel-Marti M.E. and Flichy-Fernandez A.J. and Penarrocha-Oltra D. and Balaguer-Martinez J.F. and Diago M.P. | 1 |
| Effects of hydrogen rich water and pure water on periodontal inflammatory factor level, oxidative stress level and oral flora: a systematic review and meta-analysis | Bai Y. and Wang C. and Jiang H. and Wang L. and Li N. and Zhang W. and Liu H. | 1 |
| Acceleration of purine degradation by periodontal diseases | Barnes V.M. and Teles R. and Trivedi H.M. and Devizio W. and Xu T. and Mitchell M.W. and Milburn M.V. and Guo L. | 1 |
| The Role of Periodontopathogens and Oral Microbiome in the Progression of Oral Cancer. A Review | Beltran J.F. and Viafara-Garcia S.M. and Labrador A.P. and Basterrechea J. | 1 |
| Innate cellular responses to the periodontal biofilm | Benakanakere M. and Kinane D.F. | 1 |
| Generalized aggressive periodontitis in a prepubertal patient: a case report | Bodur A. and Bodur H. and Bal B. and Balos K. | 1 |
| Oral profiles of bulimic women: Diagnosis and management. What is the evidence?. | Bretz, Walter A | 1 |
| Effects of baking-soda-containing dentifrices on oral malodor | Brunette D.M. | 1 |
| Tobacco-induced suppression of the vascular response to dental plaque | Buduneli N. and Scott D.A. AO - Scott D.A.; ORCID: https://orcid.org/0000-0003-1007-2756 | 1 |
| The role of extracellular vesicles in periodontitis: pathogenesis, diagnosis, and therapy | Cai R. and Wang L. and Zhang W. and Liu B. and Wu Y. and Pang J. and Ma C. | 1 |
| A Review of Probiotic Therapy in Preventive Dental Practice | Cannon M.L. | 1 |
| Microbial biomarkers as a predictor of periodontal treatment response: A systematic review | Chew R.J.J. and Goh C.E. and Sriram G. and Preshaw P.M. and Tan K.S. AO - Chew, Ren Jie Jacob; ORCID: https://orcid.org/0000-0003-4407-0134 AO - Go... | 1 |
| Periodontitis and angular alveolar lesions: A critical distinction | Clarke N.G. and Hirsch R.S. | 1 |
| Periodontal and oral health status of people with Cystic Fibrosis: a systematic review | Coffey N. and O' Leary F. and Burke F. and Roberts A. and Hayes M. | 1 |
| Oral microbiota features in subjects with down syndrome and periodontal diseases: A systematic review | Contaldo M. and Lucchese A. and Romano A. and Vella F.D. and Di Stasio D. and Serpico R. and Petruzzi M. | 1 |
| Dysbiosis From a Microbial and Host Perspective Relative to Oral Health and Disease | Cugini C. and Ramasubbu N. and Tsiagbe V.K. and Fine D.H. | 1 |
| Periodontal status of patients with dentin dysplasia type I: Report of three cases within a family | Da Ros Goncxalves L. and Oliveira C.A.G.R. and Holanda R. and Silva-Boghossian C.M. and Colombo A.P.V. and Maia L.C. and Feres-Filho E.J. | 1 |
| Recurrent lung infection due to chronic peri-odontitis | Dev Y.P. and Goyal O.P. | 1 |
| Impact of Oral Microbiome in Periodontal Health and Periodontitis: A Critical Review on Prevention and Treatment | Di Stefano M. and Polizzi A. and Santonocito S. and Romano A. and Lombardi T. and Isola G. | 1 |
| Necrotizing periodontal disease in a patient with COVID-19 history: A case report | Duman I. and Dogan B. | 1 |
| THE CONSISTENT DETECTION OF ORAL BACTERIA IN ATHEROSCLEROTIC PLAQUE DOES NOT QUALIFY FOR DENTAL TREATMENT TO REDUCE CARDIOVASCULAR RISK | Eberhard J. | 1 |
| Advances in periodontal diagnosis. 4. Potential microbiological markers | Eley B.M. and Cox S.W. | 1 |
| Effects of azithromycin versus metronidazole-amoxicillin combination as an adjunct to nonsurgical periodontal therapy of generalized aggressive periodontitis | Ercan E. and Uzun B.C. and Ustaoglu G. | 1 |
| Papillon Lefevre syndrome: treatment of two cases with a clinical microbiological and histopathological investigation | Eronat N. and Ucar F. and Kilinc G. | 1 |
| Halitosis: a review. | Feller, L and Blignaut, E | 1 |
| A Qualitative Analysis of Periodontal Pathogens in Chronic Periodontitis Patients after Nonsurgical Periodontal Therapy with and without Diode Laser Disinfection Using Benzoyl-DL Arginine-2-Naphthylamide Test: A Randomized Clinical Trial. | Fenol, Angel and Boban, Nidhi Chinnu and Jayachandran, P and Shereef, Mohammed and Balakrishnan, Biju and Lakshmi, P | 1 |
| Current concepts in the microbial etiology and treatment of chronic periodontitis. | Feres, Magda and Figueiredo, Luciene Cristina | 1 |
| How we got attached to Actinobacillus actinomycetemcomitans: A model for infectious diseases | Fine D.H. and Kaplan J.B. and Kachlany S.C. and Schreiner H.C. | 1 |
| Microbiota in Periodontitis: Advances in the Omic Era. | Fong, Shao Bing and Boyer, Emile and Bonnaure-Mallet, Martine and Meuric, Vincent | 1 |
| Dental calculus - oral health, forensic studies and archaeology: a review | Forshaw, R. | 1 |
| Unfolded p53 in non-neuronal cells supports bacterial etiology of Alzheimer's disease | French P.W. AO - French, Peter W.; ORCID: https://orcid.org/0000-0002-9290-1687 | 1 |
| Porphyromonas gingivalis in the development of periodontitis: impact on dysbiosis and inflammation. | Gasmi Benahmed, A. and Kumar Mujawdiya, P. and Noor, S. and Gasmi, A. | 1 |
| Salivary diagnostics for periodontal diseases | Giannobile W.V. | 1 |
| Saliva as a diagnostic tool for periodontal disease: current state and future directions. | Giannobile, W. V. and Beikler, T. and Kinney, J. S. and Ramseier, C. A. and Morelli, T. and Wong, D. T. | 1 |
| Antibacterial dentifrices. Clinical data and relevance with emphasis on zinc/triclosan. | Gjermo, P and Saxton, C A | 1 |
| Progress in oral personalized medicine: Contribution of 'omics' | Glurich I. and Acharya A. and Brilliant M.H. and Shukla S.K. | 1 |
| Microscopic monitoring of pathogens associated with periodontal diseases. A review | Greenstein G. and Polson A. | 1 |
| The role of bleeding upon probing in the diagnosis of periodontal disease. A literature review. | Greenstein, G | 1 |
| Efficacy of subantimicrobial dosing with doxycycline. Point/counterpoint. | Greenstein, G and Lamster, I | 1 |
| Pathogenic Mechanisms of Fusobacterium nucleatum on Oral Epithelial Cells. | Groeger, Sabine and Zhou, Yuxi and Ruf, Sabine and Meyle, Joerg | 1 |
| Gingival crevicular fluid as a periodontal diagnostic indicator- II: Inflammatory mediators, host-response modifiers and chair side diagnostic aids | Gupta G. | 1 |
| Editorial: Use of saliva in diagnosis of periodontitis: Cumulative use of bacterial and host-derived biomarkers | Gursoy U.K. and Kononen E. | 1 |
| Proteomics for the discovery of biomarkers and diagnosis of periodontitis: A critical review | Guzman Y.A. and Sakellari D. and Arsenakis M. and Floudas C.A. | 1 |
| Determination of periodontal disease activity. | Hancock, E B | 1 |
| Periodontal Microbiology | Harvey J.D. | 1 |
| Putative periodontal pathogens in the subgingival plaque of Sudanese subjects with aggressive periodontitis. | Hashim, N. T. and Linden, G. J. and Winning, L. and Ibrahim, M. E. and Gismalla, B. G. and Lundy, F. T. and El-Karim, I. A. | 1 |
| Comparative biology of chronic and aggressive periodontitis vs. peri-implantitis. | Heitz-Mayfield, Lisa J A and Lang, Niklaus P | 1 |
| Levels of common salivary protein 1 in healthy subjects and periodontal patients. | Heo, Seok-Mo and Lee, Sol and Wang, HongTao and Jeong, Jeong Hyeok and Oh, Sang Wook | 1 |
| Acute periodontal lesions | Herrera D. and Alonso B. and de Arriba L. and Santa Cruz I. and Serrano C. and Sanz M. | 1 |
| Image-based quantification of alveolar bone | Hildebolt C.F. and Bartlett T.W. and Shrout M.K. and Yokoyama-Crothers N. and Rupich R.C. | 1 |
| Bacteriological study of pigmented bacteria and molecular study based for 16s rRNA gene | Hindi N.K.K. | 1 |
| Dental diagnostics: molecular analysis of oral biofilms | Hiyari S. and Bennett K.M. | 1 |
| Candida species in periodontitis: A new villain or a new target? | Hu, Y. and Ren, B. and Cheng, L. and Deng, S. and Chen, Q. | 1 |
| Bacterial interactions in dental biofilm | Huang, R. and Li, M. and Gregory, R. L. | 1 |
| Furcation and its management - Review article | Hussain S.T. and Pavithra P. | 1 |
| Salivary metabolomics for diagnosis and monitoring diseases: Challenges and possibilities | Hyvarinen E. and Savolainen M. and Mikkonen J.J.W. and Kullaa A.M. | 1 |
| Diagnosis and treatment of HIV-associated manifestations in otolaryngology | Iacovou E. and Vlastarakos P.V. and Papacharalampous G. and Kampessis G. and Nikolopoulos T.P. | 1 |
| Prevalence of tetM, tetQ, nim and blaTEM genes in the oral cavities of Greek subjects: A pilot study | Ioannidis I. and Sakellari D. and Spala A. and Arsenakis M. and Konstantinidis A. | 1 |
| Adjunct Antimicrobial Therapy and Periodontal Surgery to Treat Generalized Aggressive Periodontitis: A Case Report | Irokawa D. and Makino-Oi A. and Fujita T. and Yamamoto S. and Tomita S. and Saito A. | 1 |
| The Roles of Neutrophils Linking Periodontitis and Atherosclerotic Cardiovascular Diseases | Irwandi R.A. and Chiesa S.T. and Hajishengallis G. and Papayannopoulos V. and Deanfield J.E. and D'Aiuto F. | 1 |
| Periodic health examination, 1993 update: 3. Periodontal diseases: Classification, diagnosis, risk factors and prevention | Ismail A.I. and Lewis D.W. and Battista R.N. and Anderson G. and Beaulieu M.-D. and Elford R.W. and Feightner J.W. and Feldman W. and Logan A.G. and Morrison B. and Offord D. and Patterson C. and Spitzer W.O. and Wang E. and Mickelson P. and Dingle J. and Beagan B. | 1 |
| Saliva biotechnology as a diagnostic tool for periodontal diseases: new challenges for clinical practice | Isola G. | 1 |
| New frontiers on adjuvants drug strategies and treatments in periodontitis | Isola G. and Polizzi A. and Santonocito S. and Dalessandri D. and Migliorati M. and Indelicato F. | 1 |
| Femoral osteomyelitis caused by oral anaerobic bacteria with mixed bacteremia of Campylobacter rectus and Parvimonas micra in a chronic periodontitis patient: a case report | Itoh N. and Akazawa N. and Ishibana Y. and Hamada S. and Hagiwara S. and Murakami H. AO - Itoh, Naoya; ORCID: https://orcid.org/0000-0001-6269-7884 | 1 |
| Current concepts in periodontal disease testing | Jeffcoat M.K. | 1 |
| Subgingival microbiome dynamic alteration associated with necrotizing periodontal disease: A case report | Jia J. and Zhou Y. and Wang X. and Liu Y. AO - Wang, Xinwen; ORCID: https://orcid.org/0000-0001-6880-8098 | 1 |
| Comparison of Red-Complex Bacteria Between Saliva and Subgingival Plaque of Periodontitis Patients: A Systematic Review and Meta-Analysis | Jiang Y. and Song B. and Brandt B.W. and Cheng L. and Zhou X. and Exterkate R.A.M. and Crielaard W. and Deng D.M. | 1 |
| Detection of periodontal microorganisms in coronary atheromatous plaque specimens of myocardial infarction patients: A systematic review and meta-analysis. | Joshi, Chaitanya and Bapat, Ranjeet and Anderson, William and Dawson, Dana and Hijazi, Karolin and Cherukara, George | 1 |
| Antimicrobial effect of Anacardium occidentale leaf extract against pathogens causing periodontal disease. | Jothi Varghese, Jothi Varghese and Tumkur, V. K. and Vasudev Ballal, Vasudev Ballal and Bhat, G. S. | 1 |
| Molecular mechanisms of periodontal disease. | Kajiya, Mikihito and Kurihara, Hidemi | 1 |
| Oral microbiota and oral cancer: Review | Kakabadze M.Z. and Paresishvili T. and Karalashvili L. and Chakhunashvili D. and Kakabadze Z. | 1 |
| Unilateral gingival enlargement - a case report. | Kalburge, J. V. and Metgud Rashmi, Metgud Rashmi | 1 |
| Effect of local drug delivery in chronic periodontitis patients: A meta-analysis. | Kalsi, Rupali and Vandana, K L and Prakash, Shobha | 1 |
| Chlorhexidine--pharmaco-biological activity and application. | Karpinski, T M and Szkaradkiewicz, A K | 1 |
| Characterization of Salivary Microbiota in Patients with Atherosclerotic Cardiovascular Disease: A Case-Control Study | Kato-Kogoe N. and Sakaguchi S. and Kamiya K. and Omori M. and Gu Y.-H. and Ito Y. and Nakamura S. and Nakano T. and Tamaki J. and Ueno T. and Hoshiga M. | 1 |
| Analysis of saliva for periodontal diagnosis--a review | Kaufman E. and Lamster I.B. | 1 |
| Xerostomia, hyposalivation, and oral microbiota in type 2 diabetic patients: a preliminary study. | Khovidhunkit, Siribang-on Piboonniyom and Suwantuntula, Tongchat and Thaweboon, Soisiri and Mitrirattanakul, Somsak and Chomkhakhai, Umawadee and Khovidhunkit, Weerapan | 1 |
| Molecular diagnostics of periodontitis | Korona-Glowniak I. and Siwiec R. and Berger M. and Malm A. and Szymanska J. | 1 |
| The role of Actinomyces spp. and related organisms in cervicofacial infections: pathomechanism, diagnosis and therapeutic aspects. | Kover, Zsanett and Nordskag, Vidar Johansen and Ban, Agnes and Gajdacs, Mario and Urban, Edit | 1 |
| Periodontopathogens: Bacteriology of periodontal disease: Mini review | Kulshrestha R. and Srinivasa T.S. and Biswas J. | 1 |
| Treponema denticola as a prognostic biomarker for periodontitis in dogs | Kwon D. and Bae K. and Kim H. and Kim S.-H. and Lee D. and Lee J.-H. | 1 |
| Oral Microbiome Is Associated With Incident Hypertension Among Postmenopausal Women | Lamonte M.J. and Gordon J.H. and Diaz-Moreno P. and Andrews C.A. and Shimbo D. and Hovey K.M. and Buck M.J. and Wactawski-Wende J. | 1 |
| Current concepts and future trends for periodontal disease and periodontal therapy, Part 2: Classification, diagnosis, and nonsurgical and surgical therapy. | Lamster, I B | 1 |
| Infantile agranulocytosis with survival into adolescence: periodontal manifestations and laboratory findings. A case report. | Lamster, I B and Oshrain, R L and Harper, D S | 1 |
| Randomized clinical trial on the effects of full mouth disinfection versus conventional quadrant therapy in the control of chronic periodontitis. | Latronico, M. and Russo, R. and Garbarino, F. and Rolandi, C. and Mascolo, A. and Blasi, G. | 1 |
| The loop-mediated isothermal amplification technique in periodontal diagnostics: A systematic review | Lenkowski M. and Nijakowski K. and Kaczmarek M. and Surdacka A. | 1 |
| Microbial identification in the management of periodontal diseases. A systematic review | Listgarten M.A. and Loomer P.M. | 1 |
| Effect of probiotics on gingival inflammation and oral microbiota: a meta-analysis. | Liu, Jia-xuan and Liu, Zhen-min and Huang, Jia-qi and Tao, Ren-chuan | 1 |
| Overview of Candida albicans and Human Papillomavirus (HPV) Infection Agents and their Biomolecular Mechanisms in Promoting Oral Cancer in Pediatric Patients | Lo Muzio L. and Ballini A. and Cantore S. and Bottalico L. and Charitos I.A. and Ambrosino M. and Nocini R. and Malcangi A. and Dioguardi M. and Cazzolla A.P. and Brauner E. and Santacroce L. and Di Cosola M. | 1 |
| The antimicrobial treatment of periodontal disease: Changing the treatment paradigm | Loesche W.J. | 1 |
| Quantitative real-time PCR using TaqMan and SYBR Green for Actinobacillus actinomycetemcomitans, Porphyromonas gingivalis, Prevotella intermedia, tetQ gene and total bacteria | Maeda H. and Fujimoto C. and Haruki Y. and Maeda T. and Kokeguchi S. and Petelin M. and Arai H. and Tanimoto I. and Nishimura F. and Takashiba S. | 1 |
| Editorial Commentary: The role of periodontal microorganisms in the pathogenesis of myocardial infarction. From PCR techniques to microbiome sequencing | Magan-Fernandez A. and Castellino G. and Cappello F. and Mesa F. AO - Magan-Fernandez, Antonio; ORCID: https://orcid.org/0000-0001-6430-2276 | 1 |
| Periodontal pathogens and risk of incident cancer in postmenopausal females: the Buffalo OsteoPerio Study. | Mai XiaoDan, Mai XiaoDan and Genco, R. J. and LaMonte, M. J. and Hovey, K. M. and Freudenheim, J. L. and Andrews, C. A. and Wactawski-Wende, J. | 1 |
| Wound biofilms: lessons learned from oral biofilms. | Mancl, Kimberly A and Kirsner, Robert S and Ajdic, Dragana | 1 |
| An increased prevalence of periodontal disease, porphyromonas gingivalis and aggregatibacter actinomycetemcomitans in anti-CCP positive individuals at-risk of inflammatory arthritis | Mankia K. and Cheng Z. and Do T. and Kang J. and Hunt L. and Meade J. and Clerehugh V. and Speirs A. and Tugnait A. and Hensor E. and Devine D. and Emery P. | 1 |
| Clinical Literature Evaluation Supports Correlation Between Oral Health And Systemic Diseases | Mark K. and Rusinol J. | 1 |
| Salivary Microbiota Composition in Patients with Oral Squamous Cell Carcinoma: A Systematic Review | Mauceri R. and Coppini M. and Vacca D. and Bertolazzi G. and Panzarella V. and Di Fede O. and Tripodo C. and Campisi G. AO - Mauceri, Rodolfo; ORCID: https://orcid.org/0000-0002-4008-6502 AO - Cop... | 1 |
| The association between Aggregatibacter actinomycetemcomitans JP2 clone and periodontitis: A systematic review and meta-analysis | Mehta, Jaimini and Eaton, Carolyn and AlAmri, Meaad and Lin, Guo-Hao and Nibali, Luigi | 1 |
| Oral pathogens, immunity, and periodontal diseases | Mesa F. and Liebana J. and Galindo-Moreno P. and O'Valle F.J. | 1 |
| Case Report of a 4-Year-Old Girl with Stage IV Grade C Localized Periodontitis (Pre-Puberal Localized Aggressive Periodontitis) Affected by Misrecognition and Late Diagnosis | Moga, Radu-Andrei and Olteanu, Cristian Doru and Delean, Ada Gabriela | 1 |
| Inflammatory mediators in saliva and gingival fluid of children with congenital heart defect | Mohamed Ali H. and Mustafa M. and Suliman S. and Elshazali O.H. and Ali R.W. and Berggreen E. AO - Mohamed Ali, Hiba; ORCID: https://orcid.org/0000-0003-2552-572X | 1 |
| The characteristics of biofilms in peri-implant disease | Mombelli A. and Decaillet F. | 1 |
| Association between oral malodor and adult periodontitis: a review | Morita M. and Wang H.L. | 1 |
| Oral microbial biofilms: an update | Mosaddad S.A. and Tahmasebi E. and Yazdanian A. and Rezvani M.B. and Seifalian A. and Yazdanian M. and Tebyanian H. AO - Tebyanian, Hamid; ORCID: https://orcid.org/0000-0002-5647-637X | 1 |
| STRONG ORAL MALODOUR IN A PATIENT WITH PERIODONTITIS: ANALYSIS OF POSSIBLE CAUSATIVE FACTORS | Music L. and Badovinac A. and Zabarovic M. | 1 |
| Elevated MicroRNA-128 in Periodontitis Mitigates Tumor Necrosis Factor-alpha Response via p38 Signaling Pathway in Macrophages | Na H.S. and Park M.H. and Song Y.R. and Kim S. and Kim H.-J. and Lee J.Y. and Choi J.-I. and Chung J. | 1 |
| The effects of oral rinses on halitosis. | Nachnani, S | 1 |
| Periodontal disease and risk of atherosclerotic coronary heart disease | Nakajima T. and Yamazaki K. | 1 |
| COVID-19 and oral diseases: Assessing manifestations of a new pathogen in oral infections | Naqvi A.R. and Schwartz J. and Brandini D.A. and Schaller S. and Hussein H. and Valverde A. and Naqvi R.A. and Shukla D. AO - Naqvi, Afsar R.; ORCID: https://orcid.org/0000-0001-7436-3056 | 1 |
| Plaque-host imbalance in severe periodontitis. A discussion based on two cases | Newman H.N. and Rule D.C. | 1 |
| Microbial sampling process can change results of microbiological analysis in periodontitis diagnosis. A minireview | Nguyen-Hieu T. | 1 |
| Methanogenic archaea in subgingival sites: a review. | Nguyen-Hieu, Tung and Khelaifia, Saber and Aboudharam, Gerard and Drancourt, Michel | 1 |
| Aggressive Periodontitis: Microbes and host response, who to blame? | Nibali L. | 1 |
| Empirical or microbiologically guided systemic antimicrobials as adjuncts to non-surgical periodontal therapy? A systematic review | Nibali L. and Koidou V.P. and Hamborg T. and Donos N. AO - Nibali, Luigi; ORCID: https://orcid.org/0000-0002-7750-5010 | 1 |
| Periodontal infectogenomics: systematic review of associations between host genetic variants and subgingival microbial detection. | Nibali, L. and Iorio, A. di and Onabolu, O. and Lin GuoHao, Lin GuoHao | 1 |
| Unusual gingival enlargement with rapidly progressive periodontitis. Report of a case | Nitta H. and Kameyama Y. and Ishikawa I. | 1 |
| Bacteriological diagnosis of periodontal disease | Okuda K. | 1 |
| Cultivated and not-yet-cultivated bacteria in oral biofilms. | Olsen, I. and Preza, D. and Aas, J. A. and Paster, B. J. | 1 |
| Relationship between oral microbiota and periodontal disease: A systematic review | Patini R. and Staderini E. and Lajolo C. and Lopetuso L. and Mohammed H. and Rimondini L. and Rocchetti V. and Franceschi F. and Cordaro M. and Gallenzi P. | 1 |
| Inflammation in Periodontal Disease: Possible Link to Vascular Disease | Paul O. and Arora P. and Mayer M. and Chatterjee S. | 1 |
| Periodontal infection in adult-onset Still's disease patient: Clinical and haematological considerations | Pessoa L. and Galvao V. and Ferreira C. and Neto L.S. | 1 |
| Microbiota and Oral Cancer as A Complex and Dynamic Microenvironment: A Narrative Review from Etiology to Prognosis | Pignatelli P. and Romei F.M. and Bondi D. and Giuliani M. and Piattelli A. and Curia M.C. AO - Pignatelli, Pamela; ORCID: https://orcid.org/0000-0002-3023-7143 AO - C... | 1 |
| Late-onset Papillon-Lefevre syndrome without alteration of the cathepsin C gene | Pilger U. and Hennies H.C. and Truschnegg A. and Aberer E. | 1 |
| Increased levels of Malondialdehyde and Cathepsin C by Aggregatibacter actinomycetemcomitans in saliva as aggressive periodontitis biomarkers: a review. | Ramadhani, Nastiti Faradilla and Nugraha, Alexander Patera and Gofur, Nanda Rachmad Putra and Permatasari, Ryza Indah and Ridwan, Rini Devijanti | 1 |
| Peri-implantitis Update: Risk Indicators, Diagnosis, and Treatment. | Rokaya, Dinesh and Srimaneepong, Viritpon and Wisitrasameewon, Wichaya and Humagain, Manoj and Thunyakitpisal, Pasutha | 1 |
| Historical and contemporary hypotheses on the development of oral diseases: Are we there yet? | Rosier B.T. and De Jager M. and Zaura E. and Krom B.P. | 1 |
| A novel method for the detection of TNF-alpha in gingival crevicular fluid | Rossomando E.F. and White L. | 1 |
| [Diagnostic etiology of periodontal disease]. | Sabelli, C and Piovano, S | 1 |
| Can Periodontitis Influence the Progression of Abdominal Aortic Aneurysm? A Systematic Review | Salhi L. and Rompen E. and Sakalihasan N. and Laleman I. and Teughels W. and Michel J.-B. and Lambert F. AO - Salhi, Leila; ORCID: https://orcid.org/0000-0003-3529-8452 | 1 |
| Methods of detection of Actinobacillus actinomycetemcomitans, Porphyromonas gingivalis and Tannerella forsythensis in periodontal microbiology, with special emphasis on advanced molecular techniques: a review. | Sanz, Mariano and Lau, Laura and Herrera, David and Morillo, Juan Manuel and Silva, Augusto | 1 |
| Matrix metalloproteinases and periodontal diseases | Sapna G. and Gokul S. and Bagri-Manjrekar K. | 1 |
| Periodontal therapy in siblings with Papillon-Lefevre syndrome and tinea capitis: a report of two cases. | Schacher, B. and Baron, F. and Ludwig, B. and Valesky, E. and Noack, B. and Eickholz, P. | 1 |
| Effect of genetic variability on the inflammatory response to periodontal infection. | Shapira, Lior and Wilensky, Asaf and Kinane, Denis F | 1 |
| The role of Toll-like receptors in periodontitis. | Song, B and Zhang, Y L and Chen, L J and Zhou, T and Huang, W K and Zhou, X and Shao, L Q | 1 |
| Markers of periodontal disease susceptibility and activity--a review | Stark D.E. and Hoover J.N. | 1 |
| Drug-induced gingival enlargement | Straka M. and Varga I. and Erdelsky I. and Straka-Trapezanlidis M. and Krnoulova J. | 1 |
| Gingival crevicular fluid: An overview | Subbarao K. and Nattuthurai G. and Sundararajan S. and Sujith I. and Joseph J. and Syedshah Y. | 1 |
| Nexus between COVID-19 and periodontal disease | Sukumar K. and Tadepalli A. AO - Sukumar, Kanchana; ORCID: https://orcid.org/0000-0003-4029-9150 | 1 |
| An insight into the link between oral health and neurological diseases. | Surajit Pathak, Surajit Pathak and Prasad, S. V. and Sushmitha Sriramulu, Sushmitha Sriramulu and Ganesan Jothimani, Ganesan Jothimani and Ramachandran Murugesan, Ramachandran Murugesan and Marotta, F. and Antara Banerjee, Antara Banerjee and Matsumoto, T. and Yamaoka, Y. | 1 |
| Diagnostic biomarkers for oral and periodontal diseases. | Taba, Mario Jr and Kinney, Janet and Kim, Amy S and Giannobile, William V | 1 |
| Extra-oral halitosis: An overview | Tangerman A. and Winkel E.G. | 1 |
| Protein biomarkers of periodontitis in saliva. | Taylor, John J | 1 |
| Role of salivary interleukin 1 in chronic periodontitis: A review | Thirumalaisamy V. and Gajendran P. | 1 |
| Periodontitis among non-Hispanic African Americans versus other populations | Tong L. and Bolan N. | 1 |
| Langerhans' cell histiocytosis in a 5-year-old girl: Evidence of pPeriodontal pathogens | Torrungruang K. and Sittisomwong S. and Rojanasomsith K. and Asvanit P. and Korkongwisarut D. and Vipismakul V. | 1 |
| What exactly distinguishes aggressive from chronic periodontitis: is it mainly a difference in the degree of bacterial invasiveness? | Van der Velden U. | 1 |
| The effect of amoxicillin on destructive periodontitis. A case report. | van Oosten, M A and Hug, H U and Mikx, F H and Renggli, H H | 1 |
| Microbiology in diagnosis and treatment planning in periodontics | van Winkelhoff A.J. | 1 |
| A case report of chronic neutropenia: Clinical and ultrastructural findings | Vaughan A.G. and Vrahopoulos T.P. and Joachim F. and Sati K. and Barber P. and Newman H.N. | 1 |
| [Diagnosis of periodontitis]. | Vogel, G | 1 |
| Empyema caused by Anaeroglobus geminates, a case report with literature review | Wang C.-H. and Kan L.-P. and Sun J.-R. and Yu C.-M. and Yin T. and Huang T.-W. and Tsai W.-C. and Yang Y.-S. | 1 |
| Roles of oral bacteria in cardiovascular diseases - From molecular mechanisms to clinical cases: Treatment of periodontal disease regarded as biofilm infection: Systemic administration of azithromycin | Wang P.-L. | 1 |
| Chronic Oral Inflammation and the Progression of Periodontal Pathology in the Third Molar Region | White Jr. R.P. and Offenbacher S. and Blakey G.H. and Haug R.H. and Jacks M.T. and Nance P.E. and Phillips C. | 1 |
| [Juvenile periodontitis (JP)--a review of the literature 1979-1984]. | Widmer, B R | 1 |
| Detection of high-risk groups and individuals for periodontal diseases: laboratory markers from analysis of saliva. | Wilton, J M and Curtis, M A and Gillett, I R and Griffiths, G S and Maiden, M F and Sterne, J A and Wilson, D T and Johnson, N W | 1 |
| Bacteria as risk markers for periodontitis | Wolff L. and Dahlen G. and Aeppli D. | 1 |
| Salt and peroxide compared with conventional oral hygiene. II. Microbial results. | Wolff, L F and Pihlstrom, B L and Bakdash, M B and Schaffer, E M and Jensen, J R and Aeppli, D M and Bandt, C L | 1 |
| Saliva testing for Helicobacter pylori infection in children and adolescents - a literature review | Zakrzewska M. and Zakrzewski M. and Gladka A. and Czarniecka-Barglowska K. and Maciorkowska E. AO - Zakrzewska, Magdalena; ORCID: https://orcid.org/0000-0002-2756-6659 | 1 |
| Elizabethkingia miricola as an opportunistic oral pathogen associated with superinfectious complications in humoral immunodeficiency: a case report. | Zdziarski, Przemyslaw and Pasciak, Mariola and Rogala, Klaudia and Korzeniowska-Kowal, Agnieszka and Gamian, Andrzej | 1 |
| Diagnostic potential and future directions of matrix metalloproteinases as biomarkers in gingival crevicular fluid of oral and systemic diseases | Zhang F. and Liu E. and Radaic A. and Yu X. and Yang S. and Yu C. and Xiao S. and Ye C. | 1 |
| Advances in the prevention and treatment of Alzheimer's disease based on oral bacteria | Zhang M. and Mi N. and Ying Z. and Lin X. and Jin Y. | 1 |
| Raman spectroscopy: a potential diagnostic tool for oral diseases | Zhang, Yu-wei and Ren, Li-ang and Wang, Qi and Wen, Zhi-ning and Liu, Cheng-cheng and Ding, Yi | 1 |
| Idiopathic gingival fibromatosis and primary analysis of dominant bacteria in subgingival biofilm: a case report | Zhu, J. and Fan, X. and Ding, L. and Song, T. | 1 |
| Periodontal infectogenomics: a systematic review update of associations between host genetic variants and subgingival microbial detection. | Zoheir, Noha and Kurushima, Yuko and Lin, Guo-Hao and Nibali, Luigi | 1 |
| A physiologically relevant culture platform for long-term studies of in vitro gingival tissue | Adelfio M. and Bonzanni M. and Callen G.E. and Paster B.J. and Hasturk H. and Ghezzi C.E. AO - Adelfio M.; ORCID: https://orcid.org/0000-0001-8293-7355 AO - Callen G... | 2 |
| Change in diet and oral hygiene over an 8-week period: Effects on oral health and oral biofilm | Al-Ahmad A. and Roth D. and Wolkewitz M. and Wiedmann-Al-Ahmad M. and Follo M. and Ratka-Kruger P. and Deimling D. and Hellwig E. and Hannig C. | 2 |
| Quantitative analysis of classical and new putative periodontal pathogens in subgingival biofilm: a case-control study | Al-hebshi N.N. and Al-Alimi A. and Taiyeb-Ali T. and Jaafar N. | 2 |
| Osteogenic potential of periodontal ligament stem cells are unaffected after exposure to lipopolysaccharides | Albiero M.L. and Amorim B.R. and Casati M.Z. and Sallum E.A. and Nociti F.H. and Silverio K.G. | 2 |
| Microbial associations of 4 putative periodontal pathogens in Sudanese adult periodontitis patients determined by DNA probe analysis | Ali R.W. and Skaug N. and Nilsen R. and Bakken V. | 2 |
| Improved, low-cost selective culture medium for actinobacillus actinomycetemcomitans | Alsina M. and Olle E. and Frias J. | 2 |
| Inhibitory test of andaliman (Zanthoxylum achantopodium DC) extract mouthwash against dental plaque bacteria. | Amalia, Martina and Yosuana, Priscillia Sekar and Mohammad, Iqlima Salsabila and Nababan, Filya Suri Risky and Zulkarnain, and Wulandari, Pitu and Nasution, Aini Hariyani and Syahputra, Armia | 2 |
| Validation of a quantitative real-time PCR assay and comparison with fluorescence microscopy and selective agar plate counting for species-specific quantification of an in vitro subgingival biofilm model | Ammann T.W. and Bostanci N. and Belibasakis G.N. and Thurnheer T. | 2 |
| The clinical evaluation of Vi-one chlorhexidine mouthwash on plaque-induced gingivitis: A double-blind randomized clinical trial. | Amoian, Babak and Omidbakhsh, Marzyeh and Khafri, Soraya | 2 |
| The Oral Microbiome across Oral Sites in Cats with Chronic Gingivostomatitis, Periodontal Disease, and Tooth Resorption Compared with Healthy Cats | Anderson J.G. and Rojas C.A. and Scarsella E. and Entrolezo Z. and Jospin G. and Hoffman S.L. and Force J. and MacLellan R.H. and Peak M. and Shope B.H. and Tsugawa A.J. and Ganz H.H. AO - Rojas, Connie A.; ORCID: https://orcid.org/0000-0002-6097-8970 AO - Scar... | 2 |
| Isolation and molecular detection of methylotrophic bacteria occurring in the human mouth | Anesti V. and McDonald I.R. and Ramaswamy M. and Wade W.G. and Kelly D.P. and Wood A.P. | 2 |
| A bacterial sensitivity test to determine the effectiveness of minocycline HCI (Minocin), erythromycin (Erythrocin) and ampicillin (Ampicin) on the predominant microorganisms present in a diseased periodontium | Angeles B.L. and Book D.R. and Go K.G. and Lim D.J. and Uy H.G. | 2 |
| Prophylaxis and treatment of adverse oral conditions with biologically active peptides | Anonymous. | 2 |
| A comparative evaluation of the antibacterial efficacy of honey in vitro and antiplaque efficacy in a 4-day plaque regrowth model in vivo: preliminary results. | Aparna, S. and Srirangarajan, S. and Veena Malgi, Veena Malgi and Setlur, K. P. and Shashidhar, R. and Swati Setty, Swati Setty and Srinath Thakur, Srinath Thakur | 2 |
| The diagnosis of periodontal disease in private practice | Apsey D.J. and Kaciroti N. and Loesche W.J. | 2 |
| Antimicrobial susceptibility of moxifloxacin against gram-negative enteric rods from Colombian patients with chronic periodontitis. | Ardila, C. M. and Fernandez, N. and Guzman, I. C. | 2 |
| Bacteria and bacterial DNA in atherosclerotic plaque and aneurysmal wall biopsies from patients with and without periodontitis. | Armingohar, Zahra and Jorgensen, Jorgen J and Kristoffersen, Anne Karin and Abesha-Belay, Emnet and Olsen, Ingar | 2 |
| An In Vitro Evaluation of the Antibacterial Efficacy of Solanum xanthocarpum Extracts on Bacteria From Dental Plaque Biofilm. | Arumuganainar, Deepavalli and Subramaniam, Gopinath and Kurumathur Vasudevan, Arun and Subbusamy Kanakasabapathy, Balaji | 2 |
| Absence of Helicobacter pylori in subgingival samples determined by polymerase chain reaction. | Asikainen, S and Chen, C and Slots, J | 2 |
| Inducible expression of A Disintegrin and Metalloproteinase 8 in chronic periodontitis and gingival epithelial cells. | Aung, W. P. P. and Chotjumlong, P. and Pata, S. and Montreekachon, P. and Supanchart, C. and Khongkhunthian, S. and Sastraruji, T. and Krisanaprakornkit, S. | 2 |
| Effects of ultrasonic and sonic scalers on dental plaque microflora in vitro and in vivo | Baehni, P. and Thilo, B. and Chapuis, B. and Pernet, D. | 2 |
| Potential Application of Non-Invasive Optical Imaging Methods in Orthodontic Diagnosis | Baek, J. H. | 2 |
| Probiotic curd as antibacterial agent against pathogens causing oral deformities - in vitro microbiological study | Balaganesh S. and Kumar P. and Girija A.S.S. and Rathinavelu P.K. | 2 |
| Bilophila wadsworthia: A unique gram-negative anaerobic rod | Baron E.J. | 2 |
| In vitro antioxidant activities of antioxidant-enriched toothpastes | Battino M. and Ferreiro M.S. and Armeni T. and Politi A. and Bompadre S. and Massoli A. and Bullon P. | 2 |
| Validation of antibiotic efficacy on in vitro subgingival biofilms | Belibasakis G.N. and Thurnheer T. | 2 |
| Susceptibility to various oral antiseptics of Porphyromonas gingivalis W83 within a biofilm | Bercy P. and Lasserre J. | 2 |
| Septic pericarditis and pneumopericardium in a dog with an oesophageal foreign body | Botha W.J. and Mukorera V. and Kirberger R.M. | 2 |
| Reduced human beta defensin 3 in individuals with periodontal disease | Brancatisano F.L. and Maisetta G. and Barsotti F. and Esin S. and Miceli M. and Gabriele M. and Giuca M.R. and Campa M. and Batoni G. | 2 |
| Efficacy of Listerine, Meridol and chlorhexidine mouthrinses on plaque, gingivitis and plaque bacteria vitality | Brecx M. and Netuschil L. and Reichert B. and Schreil G. | 2 |
| Differentiation of oral bacteria in in vitro cultures and human saliva by secondary electrospray ionization - mass spectrometry. | Bregy, Lukas and Muggler, Annick R and Martinez-Lozano Sinues, Pablo and Garcia-Gomez, Diego and Suter, Yannick and Belibasakis, Georgios N and Kohler, Malcolm and Schmidlin, Patrick R and Zenobi, Renato | 2 |
| Defensin-induced adaptive immunity in mice and its potential in preventing periodontal disease | Brogden K.A. and Heidari M. and Sacco R.E. and Palmquist D. and Guthmiller J.M. and Johnson G.K. and Jia H.P. and Tack B.F. and McCray Jr. P.B. | 2 |
| Periodontal infections and pre-term low birth weight: a case-control study. | Buduneli, Nurcan and Baylas, Haluk and Buduneli, Eralp and Turkoglu, Oya and Kose, Timur and Dahlen, Gunnar | 2 |
| Porphyromonas gingivalis differentially modulates cell death profile in Ox-LDL and TNF-alpha pre-treated endothelial cells | Bugueno I.M. and Khelif Y. and Seelam N. and Morand D.-N. and Tenenbaum H. and Davideau J.-L. and Huck O. | 2 |
| Clinical significance of ragA, ragB, and PG0982 genes in Porphyromonas gingivalis isolates from periodontitis patients. | Bunte, Kubra and Kuhn, Christina and Walther, Carolin and Peters, Ulrike and Aarabi, Ghazal and Smeets, Ralf and Beikler, Thomas | 2 |
| The scientific rationale and development of an optimized stannous fluoride dentifrice, Part 1. | Burke, M R and Gambogi, R J and Simone, A J and Williams, M I | 2 |
| C-reactive protein and serum amyloid A in a canine model of chronic apical periodontitis. | Buttke, T. M. and Shipper, G. and Delano, E. O. and Trope, M. | 2 |
| Gingival changes during pregnancy: III. Impact of clinical, microbiological, immunological and socio-demographic factors on gingival inflammation. | Carrillo-de-Albornoz, A. and Figuero, E. and Herrera, D. and Cuesta, P. and Bascones-Martinez, A. | 2 |
| Gingival changes during pregnancy: II. Influence of hormonal variations on the subgingival biofilm. | Carrillo-de-Albornoz, Ana and Figuero, Elena and Herrera, David and Bascones-Martinez, Antonio | 2 |
| Outer Membrane Vesicles From Fusobacterium nucleatum Switch M0-Like Macrophages Toward the M1 Phenotype to Destroy Periodontal Tissues in Mice | Chen G. and Sun Q. and Cai Q. and Zhou H. | 2 |
| The use of monoclonal antibodies to detect Bacteroides gingivalis in biological samples | Chen P. and Bochacki V. and Reynolds H.S. and Beanan J. and Tatakis D.N. and Zambon J.J. and Genco R.J. | 2 |
| NOD2 contributes to Parvimonas micra-induced bone resorption in diabetic rats with experimental periodontitis | Chen, Ying-Yi and Tan, Li and Su, Xiao-Lin and Chen, Ning-Xin and Liu, Qiong and Feng, Yun-Zhi and Guo, Yue | 2 |
| Exogenous monocyte myeloid-derived suppressor cells ameliorate immune imbalance, neuroinflammation and cognitive impairment in 5xFAD mice infected with Porphyromonas gingivalis | Cheng X. and Chi L. and Lin T. and Liang F. and Pei Z. and Sun J. and Teng W. | 2 |
| Relationship between the burden of major periodontal bacteria and serum lipid profile in a cross-sectional Japanese study | Choi, Youn-Hee and Kosaka, Takayuki and Ojima, Miki and Sekine, Shinichi and Kokubo, Yoshihiro and Watanabe, Makoto and Miyamoto, Yoshihiro and Ono, Takahiro and Amano, Atsuo | 2 |
| Phylogenetic analysis of pathogen-related oral spirochetes | Chol B.-K. and Wyss C. and Gobel U.B. | 2 |
| Sequential colonization of periodontal pathogens in induction of periodontal disease and atherosclerosis in LDLRnull mice | Chukkapalli S.S. and Easwaran M. and Rivera-Kweh M.F. and Velsko I.M. and Ambadapadi S. and Dai J. and Larjava H. and Lucas A.R. and Kesavalu L. | 2 |
| Bacterial antibody titers in ligature-induced periodontitis in beagle dogs | Chung C.P. and Nisengard R. and Slots J. and Ciancio S. | 2 |
| Ultrastructural examination of human periodontal pockets following the use of an oral irrigation device in vivo. | Cobb, C M and Rodgers, R L and Killoy, W J | 2 |
| The effects of nicotine and cotinine on Porphyromonas gingivalis colonisation of epithelial cells | Cogo K. and Calvi B.M. and Mariano F.S. and Franco G.C.N. and Goncalves R.B. and Groppo F.C. | 2 |
| Optimized oligonucleotides for the differentiation of Prevotella intermedia and Prevotella nigrescens | Conrads G. and Pelz K. and Hughes B. and Seyfarth I. and Devine D.A. | 2 |
| Characteristics of systemic antibody responses of nonhuman primates to cell envelope and cell wall antigens from periodontal pathogens | Cox S.E. and Holt S.C. and Ebersole J.L. | 2 |
| Additive manufactured titanium for prosthetic application in dentistry: Surface topography characterization and in vitro cellular response of human gingival fibroblasts (HGFs) | Crenn M.J. and Benoit A. and Rohman G. and Guilbert T. and Chaussain C. and Fromentin O. and Attal J.P. and Bardet C. | 2 |
| Er:YAG laser scaling of diseased root surfaces: a histologic study. | Crespi, Roberto and Barone, Antonio and Covani, Ugo | 2 |
| Comparison of periodontal disease in HIV seropositive subjects and controls (II). Microbiology, immunology and predictors of disease progression | Cross D.L. and Smith G.L. | 2 |
| Oral malodor and its relevance to periodontal disease in the dog | Culham N. and Rawlings J.M. | 2 |
| Comparative evaluation of antimicrobial activity of pomegranate-containing mouthwash against oral-biofilm forming organisms: An invitro microbial study | Dabholkar C.S. and Shah M. and Kathariya R. and Bajaj M. and Doshi Y. | 2 |
| Expression of matrix metalloproteinases in healthy and diseased human gingiva. | Dahan, M and Nawrocki, B and Elkaim, R and Soell, M and Bolcato-Bellemin, A L and Birembaut, P and Tenenbaum, H | 2 |
| Predominant bacterial species in subgingival plaque in dogs | Dahlen G. and Charalampakis G. and Abrahamsson I. and Bengtsson L. and Falsen E. | 2 |
| Development of plaque and gingivitis following antibiotic therapy in dogs | Dahlen G. and Hijl L. and Lindhe J. and Moller A. | 2 |
| Examining the Relationship: Porphyromonas gingivalis Presence in Subgingival and Atherosclerotic Plaques of Cadavers | Daneshparvar, H. and Esfahanizadeh, N. and Kalantar Joshaghani, S. and Goudarzi, H. and Hajikhani, B. and Mohammadi, S. and Sadrjamali, M. | 2 |
| Examining the relationship: <ovid:i>Porphyromonas gingivalis</ovid:i> presence in subgingival and atherosclerotic plaques of cadavers | Daneshparvar, Hamidreza and Esfahanizadeh, Nasrin and Joshaghani, Shermineh Kalantar and Goudarzi, Hossein and Hajikhani, Bahareh and Mohammadi, Saba and Sadrjamali, Melika | 2 |
| The Unculturables: Targeted isolation of bacterial species associated with canine periodontal health or disease from dental plaque | Davis I.J. and Bull C. and Horsfall A. and Morley I. and Harris S. | 2 |
| Gene Sequence Analyses of the Healthy Oral Microbiome in Humans and Companion Animals. | Davis, Eric M | 2 |
| Absence of interleukin 22 affects the oral microbiota and the progression of induced periapical lesions in murine teeth | de Oliveira K.M. and da Silva R.A. and De Rossi A. and Fukada S.Y. and Feres M. and Nelson-Filho P. and da Silva L.A. | 2 |
| Study of prevalence and sensitivity pattern of dental plaque bacteria against antibiotics and pomegranate | Devi A. and Singh V. and Bhatt A.B. | 2 |
| Potential Roles of the Free Salivary Microbiome Dysbiosis in Periodontal Diseases | Diao J. and Yuan C. and Tong P. and Ma Z. and Sun X. and Zheng S. | 2 |
| Alzheimer's Disease-Like Pathology Triggered by Porphyromonas gingivalis in Wild Type Rats Is Serotype Dependent | Diaz-Zuniga J. and More J. and Melgar-Rodriguez S. and Jimenez-Union M. and Villalobos-Orchard F. and Munoz-Manriquez C. and Monasterio G. and Valdes J.L. and Vernal R. and Paula-Lima A. | 2 |
| An In Vitro Study of Bactericidal Effect of Gallium Aluminium Arsenide Laser on Anaerobic Photosensitized Periodontopathics | Dodani K. and Khare N. and Bathini C. and Mishra S. and Inamdar M.N. and Nasha A. | 2 |
| Coaggregation among periodontal pathogens, emphasizing Bacteroides gingivalis--Actinomyces viscosus cohesion on a saliva-coated mineral surface. | Ellen, R P and Schwarz-Faulkner, S and Grove, D A | 2 |
| Dental plaque inhibition by a combination of triclosan and polydimethylsiloxane (silicone oil) | Ellingsen J.E. and Rolla G. | 2 |
| Microbially-Induced Exosomes from Dendritic Cells Promote Paracrine Immune Senescence: Novel Mechanism of Bone Degenerative Disease in Mice | Elsayed R. and Elashiry M. and Liu Y. and Morandini A.C. and El-Awady A. and Elashiry M.M. and Hamrick M. and Cutler C.W. | 2 |
| Efficacy of a low-concentration chlorhexidine mouth rinse in non-compliant periodontitis patients attending a supportive periodontal care programme: a randomized clinical trial. | Escribano, M. and Herrera, D. and Morante, S. and Teughels, W. and Quirynen, M. and Sanz, M. | 2 |
| Monocyte differentiation into destructive macrophages on in vitro administration of gingival crevicular fluid from periodontitis patients | Fageeh H.I. and Fageeh H.N. and Patil S. | 2 |
| Comparison of different modes of photo-activated disinfection against Porphyromonas gingivalis: An in vitro study | Fekrazad R. and Khoei F. and Bahador A. and Hakimiha N. | 2 |
| In vitro antimicrobial activity of plant extracts and propolis in saliva samples of healthy and periodontally-involved subjects. | Feres, Magda and Figueiredo, Luciene Cristina and Barreto, Ilizvania Maroly Quindere and Coelho, Mary Hellen M and Araujo, Marcelo Werneck Barata and Cortelli, Sheila Cavalca | 2 |
| Saliva from subjects harboring Actinobacillus actinomycetemcomitans kills Streptococcus mutans in vitro. | Fine, D. H. and Furgang, D. and Goldman, D. | 2 |
| Can salivary activity predict periodontal breakdown in A. actinomycetemcomitans infected adolescents?. | Fine, Daniel H and Furgang, David and McKiernan, Marie and Rubin, Michelle | 2 |
| Detection of Eikenella corrodens and Actinobacillus actinomycetemcomitans by use of the polymerase chain reaction (PCR) in vitro and in subgingival plaque | Furcht C. and Eschrich K. and Merte K. | 2 |
| Occurrence of porphyromonas gingivalis and its antibacterial susceptibility to metronidazole and tetracycline in patients with chronic periodontitis | Gamboa F. and Acosta A. and Garcia D.-A. and Velosa J. and Araya N. and Ledergerber R. | 2 |
| Necrotizing gingivitis: microbial diversity and quantification of protein secretion in necrotizing gingivitis. | Gerhard, Nicolas and Thurnheer, Thomas and Kreutzer, Susanne and Gmur, Rudolf Dominik and Attin, Thomas and Russo, Giancarlo and Karygianni, Lamprini | 2 |
| Is feline foamy virus really apathogenic? | German A.C. and Harbour D.A. and Helps C.R. and Gruffydd-Jones T.J. | 2 |
| Fluorescence in situ hybridization for direct visualization of Gram-negative anaerobes in subgingival plaque samples | Gersdorf H. and Pelz K. and Gobel U.B. | 2 |
| Innate immune recognition of invasive bacteria accelerates atherosclerosis in apolipoprotein E-deficient mice | Gibson III F.C. and Hong C. and Chou H.-H. and Yumoto H. and Chen J. and Lien E. and Wong J. and Genco C.A. | 2 |
| An update on herbal anti-inflammatory agents in periodontal therapy | Giraudi M. and Romano F. and Aimetti M. | 2 |
| Evaluation of sulcular sulphide level monitoring using a portable sensor system. | Gleissner, C and Springborn, I and Willershausen, B | 2 |
| Dominant cross-reactive antibodies generated during the response to a variety of oral bacterial species detect phosphorylcholine | Gmur, R. and Thurnheer, T. and Guggenheim, B. | 2 |
| Immunolocalization of the TGFB1 system in submandibular gland fibrosis after experimental periodontitis in rats. | Gonzalez, Candela R and Amer, Mariano Ar and Vitullo, Alfredo D and Gonzalez-Calvar, Silvia I and Vacas, Maria I | 2 |
| Periodontal disease treatment by local drug delivery. | Goodson, J M and Offenbacher, S and Farr, D H and Hogan, P E | 2 |
| Antimicrobial peptides in periodontal innate defense | Gorr S.U. | 2 |
| Two cases of feline pyothorax: Medical versus surgical treatment and associated challenges | Gorris F. and Faut S. and De Rooster H. and Vandervekens E. and Bosmans T. and Daminet S. and Smets P. and Paepe D. | 2 |
| Comparison of gingival crevicular fluid sampling methods in patients with severe chronic periodontitis. | Guentsch, Arndt and Kramesberger, Martin and Sroka, Aneta and Pfister, Wolfgang and Potempa, Jan and Eick, Sigrun | 2 |
| Comparison of Antimicrobial Effects of Three Different Mouthwashes. | Haerian-Ardakani, Ahmad and Rezaei, Mahsa and Talebi-Ardakani, Mohammadreza and Keshavarz Valian, Nasrin and Amid, Reza and Meimandi, Mansoor and Esmailnejad, Azadeh and Ariankia, Azarnoosh | 2 |
| Interactions of oral pathogens with toll-like receptors: possible role in atherosclerosis | Hajishengallis G. and Sharma A. and Russell M.W. and Genco R.J. | 2 |
| The r40-kDa outer membrane protein human monoclonal antibody protects against porphyromonas gingivalis-induced bone loss in rats | Hamada N. and Watanabe K. and Tahara T. and Nakazawa K. and Ishida I. and Shibata Y. and Kobayashi T. and Yoshie H. and Abiko Y. and Umemoto T. | 2 |
| Evaluation of the antimicrobial activity of dentifrices on human oral bacteria | Haraszthy V.I. and Zambon J. and Sreenivasan P.K. | 2 |
| Sampling of periodontal pathogens by paper points: Evaluation of basic parameters | Hartroth B. and Seyfahrt I. and Conrads G. | 2 |
| Antimicrobial susceptibility of subgingival bacterial flora in dogs with gingivitis | Harvey C.E. and Thornsberry C. and Miller B.R. and Shofer F.S. | 2 |
| Identification of <ovid:i>Streptococcus sanguinis</ovid:i> genes producing biofilm from gingivitis | Hassan, Pakhshan Abdullah and Saeed, Chiman Hameed and Rashid, Sirwan Ahmed and Sorchee, Sawsan Mohammed and Shareef, Suhayla Hamad | 2 |
| Periodontal pathogens are associated with osteoarthritis in a miniature pig model | Haughan J.E. and Indugun R. and Vecherelli B. and Engiles J. and Pitta D. and Schaer T.P. | 2 |
| Human periodontitis-associated salivary microbiome affects the immune response of diabetic mice | He J. and Shen X. and Fu D. and Yang Y. and Xiong K. and Zhao L. and Xie H. and Pelekos G. and Li Y. | 2 |
| Assessing the antimicrobial effect of the essential oil of Myrtus communis on the clinical isolates of Porphyromonas gingivalis: an in vitro study. | Hedayati, A. and Khosropanah, H. and Bazargani, A. and Abed, M. and Emami, A. | 2 |
| Longitudinal analysis at three oral sites links oral microbiota to clinical outcomes in allogeneic hematopoietic stem-cell transplant | Heidrich V. and Knebel F.H. and Bruno J.S. and de Molla V.C. and Miranda-Silva W. and Asprino P.F. and Tucunduva L. and Rocha V. and Novis Y. and Fregnani E.R. and Arrais-Rodrigues C. and Camargo A.A. AO - Knebel, Franciele H.; ORCID: https://orcid.org/0000-0001-8275-9499 AO ... | 2 |
| Microbial Diversity in the Early In Vivo-Formed Dental Biofilm | Heller D. and Helmerhorst E.J. and Gower A.C. and Siqueira W.L. and Paster B.J. and Oppenheim F.G. | 2 |
| Fcgamma-binding bacteria in periodontal lesions | Hillestad M. and Helgeland K. and Tolo K. | 2 |
| In vitro antibacterial activity of plants extracts against Porphyromonas gingivalis, Prevotella intermedia and aggregatibacter actinomycetemcomitans Streptococcus mutanus, isolated from periodontitis patients in Babylon province, Iraq | Hindi, N. K. K. | 2 |
| Protective effect of an egg yolk-derived immunoglobulin (IgY) against Prevotella intermedia-mediated gingivitis. | Hou, Y. Y. and Zhen, Y. H. and Wang, D. and Zhu, J. and Sun, D. X. and Liu, X. T. and Wang, H. X. and Liu, Y. and Long, Y. Y. and Shu, X. H. | 2 |
| Serum antibody responses to periodontal microbiota in chronic and aggressive periodontitis: a postulate revisited. | Hwang, A. M. and Stoupel, J. and Celenti, R. and Demmer, R. T. and Papapanou, P. N. | 2 |
| OmpA-like proteins of Porphyromonas gingivalis contribute to serum resistance and prevent Toll-like receptor 4-mediated host cell activation | Inomata M. and Horie T. and Into T. | 2 |
| Bifidobacterium animalis subsp lactis HN019 presents antimicrobial potential against periodontopathogens and modulates the immunological response of oral mucosa in periodontitis patients. | Invernici, Marcos M. and Furlaneto, Flavia A. C. and Salvador, Sergio L. and Ouwehand, Arthur C. and Salminen, Seppo and Mantziari, Anastasia and Vinderola, Gabriel and Ervolino, Edilson and Santana, Sandro Isaias and Silva, Pedro Henrique Felix and Messora, Michel R. | 2 |
| Subgingival temperature in rats with natural gingivitis | Isogai E. and Isogai H. and Hirose K. and Kimura K. and Fujii N. and Shibahara N. | 2 |
| Volatile Sulfur Compounds Produced by the Anaerobic Bacteria Porphyromonas spp. Isolated from the Oral Cavities of Dogs | Ito N. and Itoh N. and Kameshima S. AO - Itoh, Naoyuki; ORCID: https://orcid.org/0000-0001-6424-3886 | 2 |
| Evaluation of the ability of the trypsin-like peptidase activity assay to detect severe periodontitis | Iwasaki M. and Usui M. and Ariyoshi W. and Nakashima K. and Nagai-Yoshioka Y. and Inoue M. and Kobayashi K. and Nishihara T. | 2 |
| Antibacterial effect of an herbal product persica on porphyromonas gingivalis and aggregatibacter actinomycetemcomitans: an in-vitro study. | Jelvehgaran Esfahani, Zahra and Kadkhoda, Zeinab and Eshraghi, Seyed Saeed and Salehi Surmaghi, Mohammad Hossein | 2 |
| Comparison of curet and paper point sampling of subgingival bacteria as analyzed by real-time polymerase chain reaction. | Jervoe-Storm, Pia-Merete and Alahdab, Hazem and Koltzscher, Max and Fimmers, Rolf and Jepsen, Soren | 2 |
| Inhibitory effects of plant extracts on multi-species dental biofilm formation in-vitro. | John, N. R. and Gala, V. C. and Sawant, C. S. | 2 |
| Treponema denticola stimulates Oncostatin M cytokine release and de novo synthesis in neutrophils and macrophages | Jones M.M. and Vanyo S.T. and Ibraheem W. and Maddi A. and Visser M.B. AO - Jones, Megan M.; ORCID: https://orcid.org/0000-0002-4231-6607 AO - Vis... | 2 |
| Comparative Evaluation on the effect of Herbal mouthwash on Putative Periodontal Pathogens - In vitro study | Joseph R.A. and Sabarish R. and Muthukumar S. and Bhat K. and Balaji S.K. | 2 |
| Detection of Periodontal Pathogens from Dental Plaques of Dogs with and without Periodontal Disease | Kacirova J. and Sondorova M. and Mad'ari A. and Stykova E. and Mucha R. and Nemcova R. and Marecakova N. and Farbakova J. and Mad'ar M. | 2 |
| Clinicomicrobiological evaluation of the efficacy of local delivery of moxifloxacin and ibuprofen gel as an adjunct to scaling and root planning in chronic periodontitis patients. | Kadadasu, Ramyasri and Atchuta, Abhinav and Palaparthy, Rajababu and Reddy, S Harinath and Sisinty, Vidyasagar and Beeravolu, Mounika | 2 |
| <ovid:i>Ex vivo</ovid:i> Detection of Amyloid-beta in Naturally Formed Oral Biofilm | Kanagasingam, Shalini and von Ruhland, Christopher and Welbury, Richard and Singhrao, Sim K. | 2 |
| Microbiological and biochemical findings in relation to clinical periodontal status in active smokers, non-smokers and passive smokers. | Kanmaz, Burcu and Lamont, Gwyneth and Danaci, Gulcan and Gogeneni, Himabindu and Buduneli, Nurcan and Scott, David A | 2 |
| Quantitation of biofilm and planktonic life forms of coexisting periodontal species. | Karched, Maribasappa and Bhardwaj, Radhika G and Inbamani, Anandavalli and Asikainen, Sirkka | 2 |
| Innate inflammatory responses of human decidual cells to periodontopathic bacteria. | Keelan, Jeffrey A and Wong, Pui-Mun and Bird, Philip S and Mitchell, Murray D | 2 |
| The microbiome associated with equine periodontitis and oral health | Kennedy R. and Lappin D.F. and Dixon P.M. and Buijs M.J. and Zaura E. and Crielaard W. and O'Donnell L. and Bennett D. and Brandt B.W. and Riggio M.P. | 2 |
| Fretibacterium sp. human oral taxon 360 is a novel biomarker for periodontitis screening in the Japanese population. | Khemwong, Thatawee and Kobayashi, Hiroaki and Ikeda, Yuichi and Matsuura, Takanori and Sudo, Takeaki and Kano, Chihiro and Mikami, Ryo and Izumi, Yuichi | 2 |
| Induction of calprotectin release by Porphyromonas gingivalis lipopolysaccharide in human neutrophils | Kido J.-I. and Kido R. and Suryono and Kataoka M. and Fagerhol M.K. and Nagata T. | 2 |
| Optimization of alveolar bone loss for ligatureinduced periodontitis between various sizes of silk ligature in mice | Kim K. and Lee J.-M. and Suh J.-Y. and Kim Y.-G. | 2 |
| Specific oral microbiome is closely associated with oral potentially malignant disorders and oral squamous cell carcinoma | Kioi M. and Isono H. and Nakajima S. | 2 |
| Development and pyrosequencing analysis of an in-vitro oral biofilm model | Kistler J.O. and Pesaro M. and Wade W.G. | 2 |
| In vitro antimicrobial activity of propolis and Arnica montana against oral pathogens | Koo H. and Gomes B.P.F.A. and Rosalen P.L. and Ambrosano G.M.B. and Park Y.K. and Cury J.A. | 2 |
| Protease inhibitor levels in periodontal health and disease. | Kretschmar, S. and Yin, L. and Roberts, F. and London, R. and Flemmig, T. T. and Arushanov, D. and Kaiyala, K. and Chung, W. O. | 2 |
| Mapping of a Subgingival Dual-Species Biofilm Model Using Confocal Raman Microscopy | Kriem L.S. and Wright K. and Ccahuana-Vasquez R.A. and Rupp S. | 2 |
| Effect of Ligilactobacillus salivarius and Other Natural Components against Anaerobic Periodontal Bacteria | Kucia M. and Wietrak E. and Szymczak M. and Kowalczyk P. AO - Szymczak, Mateusz; ORCID: https://orcid.org/0000-0003-4056-7792 AO - ... | 2 |
| Combinatorial effects of amoxicillin and metronidazole on selected periodontal bacteria and whole plaque samples | Kulik Kunz E.M. and Lenkeit K. and Waltimo T. and Weiger R. and Walter C. | 2 |
| New bacterial species associated with chronic periodontitis. | Kumar, P S and Griffen, A L and Barton, J A and Paster, B J and Moeschberger, M L and Leys, E J | 2 |
| Oxidative modification of lipoprotein by periodontopathic bacteria | Kurita-Ochiai T. and Jia R. and Hashizume T. and Yamamoto M. | 2 |
| Humoral immune response to heat shock protein 60 of Aggregatibacter actinomycetemcomitans and cross-reactivity with malondialdehyde acetaldehyde-modified LDL | Kyrklund M. and Bildo M. and Akhi R. and Nissinen A.E. and Pussinen P. and Horkko S. and Wang C. | 2 |
| Immunization with gingipain A hemagglutinin domain of Porphyromonas gingivalis induces IgM antibodies binding to malondialdehyde-acetaldehyde modified low-density lipoprotein | Kyrklund M. and Kummu O. and Kankaanpaa J. and Akhi R. and Nissinen A. and Pauliina Turunen S. and Pussinen P. and Wang C. and Horkko S. | 2 |
| Life below the gum line: Pathogenic mechanisms of Porphyromonas gingivalis | Lamont R.J. and Jenkinson H.F. | 2 |
| Antibacterial efficacy of a cetylpyridinium chloride-based mouthrinse against Fusobacterium nucleatum and in vitro plaques | Latimer J. and Munday J.L. and Buzza K.M. and Sreenivasan P.K. and McBain A.J. | 2 |
| Fusobacterium nucleatum GroEL induces risk factors of atherosclerosis in human microvascular endothelial cells and ApoE-/- mice | Lee H.-R. and Jun H.-K. and Kim H.-D. and Lee S.-H. and Choi B.-K. | 2 |
| Evidence of a direct relationship between neutrophil collagenase activity and periodontal tissue destruction in vivo: role of active enzyme in human periodontitis. | Lee, W and Aitken, S and Sodek, J and McCulloch, C A | 2 |
| Use of monoclonal antibodies with neutralizing effects on toxic antigens from human bacterial plaque to detect specific bacteria by colony blotting | Levine M. and Miller F.C. | 2 |
| Detection and strain identification of Actinobacillus actinomycetemcomitans by nested PCR | Leys E.J. and Griffen A.L. and Strong S.J. and Fuerst P.A. | 2 |
| A lingering mouthwash with sustained antibiotic release and biofilm eradication for periodontitis | Li B. and Shi L. and Liu R. and Li Z. and Cao S. and Li J. AO - Li, Jingguo; ORCID: https://orcid.org/0000-0002-0471-4540 | 2 |
| Photobiomodulation (450 nm) alters the infection of periodontitis bacteria via the ROS/MAPK/mTOR signaling pathway | Li H. and Sun T. and Liu C. and Cao Y. and Liu X. | 2 |
| Porphyromonas gingivalis infection accelerates the progression of atherosclerosis in a heterozygous apolipoprotein E-deficient murine model | Li L. and Messas E. and Batista Jr. E.L. and Levine R.A. and Amar S. | 2 |
| Parotid salivary S-IgA antibodies during experimental gingivitis in smokers and non-smokers | Lie M.A. and Myint M.M. and Schenck K. and Timmerman M.F. and van der Velden U. and van der Weijden G.A. and Loos B.G. | 2 |
| Antimicrobial activity of Antrodia camphorata extracts against oral bacteria | Lien H.-M. and Tseng C.-J. and Huang C.-L. and Lin Y.-T. and Chen C.-C. and Lai Y.-Y. | 2 |
| Combining salivary pathogen and serum antibody levels improves their diagnostic ability in detection of periodontitis. | Liljestrand, John M and Gursoy, Ulvi K and Hyvarinen, Kati and Sorsa, Timo and Suominen, Anna L and Kononen, Eija and Pussinen, Pirkko J | 2 |
| Mucoadhesive thin films for the simultaneous delivery of microbicide and anti-inflammatory drugs in the treatment of periodontal diseases | Lim S.Y. and Dafydd M. and Ong J. and Ord-McDermott L.A. and Board-Davies E. and Sands K. and Williams D. and Sloan A.J. and Heard C.M. | 2 |
| Implications of oral infections on systemic diseases in the institutionalized elderly with a special focus on pneumonia | Limeback H. | 2 |
| In vitro anti-microbial and in vivo cytokine modulating effects of different prepared Chinese herbal medicines | Lin S.-J. and Chen C.-S. and Lin S.-S. and Chou M.-Y. and Shih H.-C. and Lee I.-P. and Kao C.-T. and Ho C.-C. and Chen F.-L. and Ho Y.-C. and Hsieh K.-H. and Huang C.-R. and Yang C.-C. | 2 |
| ZIF-8 modified multifunctional injectable photopolymerizable GelMA hydrogel for the treatment of periodontitis | Liu Y. and Li T. and Sun M. and Cheng Z. and Jia W. and Jiao K. and Wang S. and Jiang K. and Yang Y. and Dai Z. and Liu L. and Liu G. and Luo Y. | 2 |
| Clustering of subgingival microbial species in adolescents with periodontitis. | Lopez, Rodrigo and Dahlen, Gunnar and Retamales, Carolina and Baelum, Vibeke | 2 |
| Periodontitis-related salivary microbiota aggravates Alzheimer's disease via gut-brain axis crosstalk | Lu J. and Zhang S. and Huang Y. and Qian J. and Tan B. and Qian X. and Zhuang J. and Zou X. and Li Y. and Yan F. AO - Yan, Fuhua; ORCID: https://orcid.org/0000-0002-6963-3530 | 2 |
| In vitro study of the effect of an essential oil and a delmopinol mouth rinse on dental plaque bacteria | LuIs H.S. and Luis L.S. and Bernardo M. | 2 |
| Detection of feline leukemia virus infection in saliva. | Lutz, H. and Jarrett, O. | 2 |
| Quantitative real-time PCR for Porphyromonas gingivalis and total bacteria | Lyons S.R. and Griffen A.L. and Leys E.J. | 2 |
| Longitudinal study on clinical and microbial analysis of periodontal status in pregnancy. | Machado, Fernanda Campos and Cesar, Dioneia Evangelista and Apolonio, Ana Carolina Morais and Ribeiro, Luiz Claudio and Ribeiro, Rosangela Almeida | 2 |
| Animal models for periodontal disease | Madden T.E. and Caton J.G. | 2 |
| Upregulation of co-stimulatory molecule expression and dendritic cell marker (CD83) on B cells in periodontal disease | Mahanonda R. and Sa-Ard-Iam N. and Yongvanitchit K. and Wisetchang M. and Ishikawa I. and Nagasawa T. and Walsh D.S. and Pichyangkul S. | 2 |
| Antimicrobial efficacy of Tulsi leaf (Ocimum sanctum) extract on periodontal pathogens: An in vitro study. | Mallikarjun, Sajjanshetty and Rao, Ashwini and Rajesh, Gururaghavendran and Shenoy, Ramya and Pai, Mithun | 2 |
| Antibody synthesis specific for nonoral antigens in inflamed gingiva | Mallison III S.M. and Szakal A.K. and Ranney R.R. and Tew J.G. | 2 |
| Eubacterium yurii subsp. schtitka subsp. nov.: Test tube brush bacteria from subgingival dental plaque | Margaret B.S. and Krywolap G.N. | 2 |
| Analysis of the effect of periodontopathogens on the molecular mechanisms of atherosclerosis. Systematic review | Marroquin T.Y. and Guauque-Olarte S. | 2 |
| Periodontal pathogens in atherosclerosis. A metagenomic and in-vitro analysis | Marroquin, T. and Guauque-Olarte, S. and Hernandez, J. C. and Florez, L. | 2 |
| Evaluation of a novel dog animal model for peri-implant disease: clinical, radiographic, microbiological and histological assessment | Martins O. and Ramos J.C. and Mota M. and Dard M. and Viegas C. and Caramelo F. and Nogueira C. and Goncalves T. and Baptista I.P. AO - Martins, Orlando; ORCID: https://orcid.org/0000-0001-6950-0925 | 2 |
| The effect of interleukin-11 on the progression of ligature-induced periodontal disease in the beagle dog | Martuscelli G. and Fiorellini J.P. and Crohin C.C. and Howell T.H. | 2 |
| Correlation of periodontal and microbiological evaluations, with serum levels of estradiol and progesterone, during different trimesters of gestation | Massoni R.S.S. and Aranha A.M.F. and Matos F.Z. and Guedes O.A. and Borges AH. and Miotto M. and Porto A.N. AO - Aranha, Andreza Maria Fabio; ORCID: https://orcid.org/0000-0003-0014-7937 | 2 |
| Effect of gallium-arsenic laser on photosensitized periodontopathic anaerobic organisms: An in vitro study. | Mathur, Setu and Kothiwale, Shaila Veerappa and Nag, Buddhi Prakash and Mathur, Tanu and Bhansali, Ashoka and Khatri, Rohit Kumar | 2 |
| Modulation of colonization by black-pigmented Bacteroides species in squirrel monkeys by immunization with Bacteroides gingivalis | McArthur W.P. and Magnusson I. and Marks R.G. and Clark W.B. | 2 |
| Randomized controlled trial of Doxycycline in prevention of recurrent periodontitis in high-risk patients: Antimicrobial activity and collagenase inhibition | McCulloch C.A.G. and Birek P. and Overall C. and Aitken S. and Lee W. and Kulkarni G. | 2 |
| Dysbiotic human oral microbiota alters systemic metabolism via modulation of gut microbiota in germ-free mice | Miyauchi E. and Kato T. and Sato K. and Suda W. and Tsuzuno T. and Yamada-Hara M. and Sasaki N. and Ohno H. and Yamazaki K. AO - Yamazaki, Kazuhisa; ORCID: https://orcid.org/0000-0002-1893-4202 | 2 |
| Gemella haemolysans inhibits the growth of the periodontal pathogen Porphyromonas gingivalis | Miyoshi, T. and Oge, S. and Nakata, S. and Ueno, Y. and Ukita, H. and Kousaka, R. and Miura, Y. and Yoshinari, N. and Yoshida, A. | 2 |
| Direct detection of Porphyromonas gingivalis in Macaca fascicularis dental plaque samples using an oligonucleotide probe | Moncla B.J. and Braham P.H. and Persson G.R. and Page R.C. and Weinberg A. | 2 |
| Effects of Colocasia antiquorum var. Esculenta Extract In Vitro and In Vivo against Periodontal Disease | Moon S.-H. and Shin S.-J. and Tae H.-J. and Oh S.-H. and Bae J.-M. AO - Oh, Seung-Han; ORCID: https://orcid.org/0000-0002-7250-721X AO - Bae, Ji... | 2 |
| A Photoacoustic-Fluorescent Imaging Probe for Proteolytic Gingipains Expressed by Porphyromonas gingivalis | Moore C. and Cheng Y. and Tjokro N. and Zhang B. and Kerr M. and Hayati M. and Chang K.C.J. and Shah N. and Chen C. and Jokerst J.V. AO - Moore, Colman; ORCID: https://orcid.org/0000-0002-1809-5955 AO - Joke... | 2 |
| A study into the plaque-inhibitory activity of experimental toothpaste formulations containing antimicrobial agents. | Moran, J. and Newcombe, R. G. and Wright, P. and Haywood, J. and Marlow, I. and Addy, M. | 2 |
| Quantitative real-time polymerase chain reaction based on single copy gene sequence for detection of periodontal pathogens | Morillo J.M. and Lau L. and Sanz M. and Herrera D. and Martin C. and Silva A. | 2 |
| Characterization of oral microbiota in 6-8-month-old small breed dogs | Morita, M. and Nambu, T. and Yamasaki, R. and Nagai-Yoshioka, Y. and Inoue, M. and Nishihara, T. and Okinaga, T. and Ariyoshi, W. | 2 |
| Effects of lactoferrin and lactoperoxidase-containing food on the oral hygiene status of older individuals: A randomized, double blinded, placebo-controlled clinical trial. | Morita, Yu and Ishikawa, Kentaro and Nakano, Manabu and Wakabayashi, Hiroyuki and Yamauchi, Koji and Abe, Fumiaki and Ooka, Takafumi and Hironaka, Shouji | 2 |
| E-test: a new technique for antimicrobial susceptibility testing for periodontal microorganisms. | Nachnani, S and Scuteri, A and Newman, M G and Avanessian, A B and Lomeli, S L | 2 |
| Effect of eucalyptus extract chewing gum on periodontal health: A double-masked, randomized trial | Nagata H. and Inagaki Y. and Tanaka M. and Ojima M. and Kataoka K. and Kuboniwa M. and Nishida N. and Shimizu K. and Osawa K. and Shizukuishi S. | 2 |
| Distribution and molecular characterization of Porphyromonas gingivalis carrying a new type of fimA gene | Nakagawa I. and Amano A. and Kimura R.K. and Nakamura T. and Kawabata S. and Hamada S. | 2 |
| Antibodies produced in vitro in the detection of periodontal bacteria by using surface plasmon resonance analysis. | Nakka, Sravya Sowdamini and Lonn, Johanna and Starkhammar Johansson, Carin and Bengtsson, Torbjorn and Nayeri, Fariba | 2 |
| PAI-2/SerpinB2 inhibits proteolytic activity in a P. gingivalis-dominated multispecies bacterial consortium | Neilands, J. and Bikker, F. J. and Kinnby, B. | 2 |
| How to select the right mouthrinses in periodontal prevention and therapy. Part I. Test systems and clinical investigations. | Netuschil, L and Hoffmann, T and Brecx, M | 2 |
| Interrelation of Cardiovascular Diseases with Anaerobic Bacteria of Subgingival Biofilm. | Nikolaeva, Elena Nikolaevna and Tsarev, Viktor Nikolaevich and Tsareva, Tatyana Viktorovna and Ippolitov, Evgenii Valeryevich and Arutyunov, Sergey Darchoevich | 2 |
| Development of a rapid latex agglutination test for periodontal pathogens. | Nisengard, R J and Mikulski, L and McDuffie, D and Bronson, P | 2 |
| Association of Treponema spp. with canine periodontitis | Nordhoff M. and Ruhe B. and Kellermeier C. and Moter A. and Schmitz R. and Brunnberg L. and Wieler L.H. | 2 |
| Autoinducer-2 and QseC control biofilm formation and in vivo virulence of Aggregatibacter actinomycetemcomitans. | Novak, Elizabeth A and Shao, HanJuan and Daep, Carlo Amorin and Demuth, Donald R | 2 |
| Effects of a novel dental chew on oral health outcomes, halitosis, and microbiota of adult dogs | Oba, P. M. and Sieja, K. M. and Schauwecker, A. and Somrak, A. J. and Hristova, T. S. and Keating, S. C. J. and Swanson, K. S. | 2 |
| Effect of Lacticaseibacillus rhamnosus L8020 on the abundance of periodontal pathogens in individuals with intellectual disability: a randomized clinical trial | Oda Y. and Kawano R. and Murakami J. and Kado I. and Okada Y. and Nikawa H. | 2 |
| Antibiotic susceptibility of aggregatibacter actinomycetemcomitans JP2 in a biofilm | Oettinger-Barak O. and Dashper S.G. and Catmull D.V. and Adams G.G. and Sela M.N. and Machtei E.E. and Reynolds E.C. | 2 |
| Associations between subgingival plaque bacterial morphotypes and clinical indices?. | Omar, A A and Newman, H N and Bulman, J and Osborn, J | 2 |
| Proteomics, lipidomics, metabolomics, and 16S DNA sequencing of dental plaque from patients with diabetes and periodontal disease | Overmyer K.A. and Rhoads T.W. and Merrill A.E. and Ye Z. and Westphall M.S. and Acharya A. and Shukla S.K. and Coon J.J. | 2 |
| Molecular detection of feline and canine periodontal pathogens | Ozavci V. and Erbas G. and Parin U. and Yuksel H.T. and Kirkan AO - Erbas, Goksel; ORCID: https://orcid.org/0000-0002-1839-754X | 2 |
| In vitro antibacterial action of Tetraclean, MTAD and five experimental irrigation solutions | Pappen F.G. and Shen Y. and Qian W. and Leonardo M.R. and Giardino L. and Haapasalo M. | 2 |
| Toothpastes with enzymes support gum health and reduce plaque formation | Paque P.N. and Schmidlin P.R. and Wiedemeier D.B. and Wegehaupt F.J. and Burrer P.D. and Korner P. and Deari S. and Sciotti M.-A. and Attin T. | 2 |
| Antimicrobial Study of Chitosan-Based Crosslinked Hydrogel Against Staphylococcus aureus, Porphyromonas gingivalis, Pseudomonas aeruginosa, and Streptococcus mutans | Parmar A.S. and Panwar A.S. | 2 |
| Evaluation of Predation Capability of Periodontopathogens Bacteria by Bdellovibrio Bacteriovorus HD100. An in Vitro Study. | Patini, Romeo and Cattani, Paola and Marchetti, Simona and Isola, Gaetano and Quaranta, Gianluca and Gallenzi, Patrizia | 2 |
| Periodontal disease burden and pathological changes in organs of dogs. | Pavlica, Z. and Petelin, M. and Juntes, P. and Erzen, D. and Crossley, D. A. and Skaleric, U. | 2 |
| Isolation and identification of Porphyromonas spp. and other putative pathogens from cats with periodontal disease | Perez-Salcedo L. and Herrera D. and Esteban-Saltiveri D. and Leon R. and Jeusette I. and Torre C. and O'Connor A. and Gonzalez I. | 2 |
| Comparison of two sampling methods for microbiological evaluation of periodontal disease in cats | Perez-Salcedo L. and Herrera D. and Esteban-Saltiveri D. and Leon R. and Jeusette I. and Torre C. and O'Connor A. and Gonzalez I. and Sanz M. | 2 |
| Molecular identification of black-pigmented bacteria from subgingival samples of cats suffering from periodontal disease | Perez-Salcedo L. and Laguna E. and Sanchez M.C. and Marin M.J. and O'Connor A. and Gonzalez I. and Sanz M. and Herrera D. | 2 |
| Macaca fascicularis as a model in which to assess the safety and efficacy of a vaccine for periodontitis. | Persson, G R and Engel, L D and Whitney, C W and Weinberg, A and Moncla, B J and Darveau, R P and Houston, L and Braham, P and Page, R C | 2 |
| Solvent exchange-induced in situ forming gel comprising ethyl cellulose-antimicrobial drugs | Phaechamud T. and Mahadlek J. AO - Phaechamud, Thawatchai; ORCID: https://orcid.org/0000-0003-2270-5303 | 2 |
| Multiple enzymes can make hydrogen sulfide from cysteine in Treponema denticola | Phillips L. and Chu L. and Kolodrubetz D. | 2 |
| Use of beta-caryophyllene to combat bacterial dental plaque formation in dogs | Pieri F.A. and Souza M.C. and Vermelho L.L. and Vermelho M.L. and Perciano P.G. and Vargas F.S. and Borges A.P. and da Veiga-Junior V.F. and Moreira M.A. | 2 |
| Multidrug-resistant serratia rubidaea strains in the oral microbiota of healthy horses | Pimenta J.C. and Saavedra M.J. and da Silva G.J. and Cotovio M. | 2 |
| In vitro antimicrobial efficiency of a mouthwash containing triclosan/gantrez and sodium bicarbonate | Pires J.R. and Rossa Jr. C. and Pizzolitto A.C. | 2 |
| Assessment of viable periodontal pathogens by reverse transcription quantitative polymerase chain reaction | Polonyi M. and Prenninger N. and Arweiler N.B. and Haririan H. and Winklehner P. and Kierstein S. | 2 |
| Inflammation associated with implants with different surface types. | Pongnarisorn, Nuchjaree J and Gemmell, Erica and Tan, Albert E S and Henry, Patrick J and Marshall, Roderick I and Seymour, Gregory J | 2 |
| Towards microbiome transplant as a therapy for periodontitis: an exploratory study of periodontitis microbial signature contrasted by oral health, caries and edentulism. | Pozhitkov, Alex E and Leroux, Brian G and Randolph, Timothy W and Beikler, Thomas and Flemmig, Thomas F and Noble, Peter A | 2 |
| Mucosal immunization with a flagellin-adjuvanted Hgp44 vaccine enhances protective immune responses in a murine Porphyromonas gingivalis infection model | Puth S. and Hong S.H. and Park M.J. and Lee H.H. and Lee Y.S. and Jeong K. and Kang I.-C. and Koh J.T. and Moon B. and Park S.C. and Rhee J.H. and Lee S.E. AO - Rhee, Joon Haeng; ORCID: https://orcid.org/0000-0003-4018-3203 AO - Lee, ... | 2 |
| Microbial penetration along the implant components of the Branemark system. An in vitro study | Quirynen M. and Bollen C.M. and Eyssen H. and van Steenberghe D. | 2 |
| Examination of periodontal pathogens in stenotic valve specimens and in whole blood samples in patients affected by aortic valve stenosis and chronic periodontitis. | Raffaelli, L and Santangelo, R and Falchetti, P and Galluccio, F and Luciani, N and Anselmi, A and Nowzari, H and Verdugo, F and Fadda, G and D'Addona, A | 2 |
| Sustained antibacterial actions of a new stabilized stannous fluoride dentifrice containing sodium hexametaphosphate | Ramji N. and Baig A. and He T. and Lawless M.A. and Saletta L. and Suszcynsky-Meister E. and Coggan J. | 2 |
| Microbiological aspects of human mandibular subperiosteal dental implants | Rams T.E. and Balkin B.E. and Roberts T.W. and Molzan A.K. | 2 |
| Centipeda periodontii in human periodontitis | Rams T.E. and Hawley C.E. and Whitaker E.J. and Degener J.E. and van Winkelhoff A.J. | 2 |
| Emergence of Antibiotic-Resistant Porphyromonas gingivalis in United States Periodontitis Patients | Rams T.E. and Sautter J.D. and van Winkelhoff A.J. | 2 |
| Prevalence of beta -lactamase-producing bacteria in human periodontitis. | Rams, T. E. and Degener, J. E. and Winkelhoff, A. J. van | 2 |
| Antibiotic resistance in human chronic periodontitis microbiota. | Rams, Thomas E and Degener, John E and van Winkelhoff, Arie J | 2 |
| Comparative in vitro resistance of human periodontal bacterial pathogens to tinidazole and four other antibiotics. | Rams, Thomas E. and Sautter, Jacqueline D. and van Winkelhoff, Arie J. | 2 |
| Clinical effect of locally delivered gel containing green tea extract as an adjunct to non-surgical periodontal treatment | Rattanasuwan K. and Rassameemasmaung S. and Sangalungkarn V. and Komoltri C. | 2 |
| Prospects for treatment of <ovid:i>Porphyromonas gingivalis</ovid:i>-mediated disease - immune-based therapy | Reynolds, E. C. and O'Brien-Simpson, N. and Rowe, T. and Nash, A. and McCluskey, J. and Vingadassalom, D. and Kleanthous, H. | 2 |
| Prospects for treatment of Porphyromonas gingivalis-mediated disease - immune-based therapy. | Reynolds, Eric C and O'Brien-Simpson, Neil and Rowe, Tony and Nash, Andrew and McCluskey, Jackie and Vingadassalom, Didier and Kleanthous, Harold | 2 |
| Molecular identification of bacteria associated with canine periodontal disease | Riggio M.P. and Lennon A. and Taylor D.J. and Bennett D. | 2 |
| Detection of pathogen-related oral spirochetes, Treponema denticola, and Treponema socranskii in dental plaque from dogs | Riviere G.R. and Thompson A.J. and Brannan R.D. and McCoy D.E. and Simonson L.G. | 2 |
| Stress and the periodontal diseases: Effects of catecholamines on the growth of periodontal bacteria in vitro | Roberts A. and Matthews J.B. and Socransky S.S. and Freestone P.P.E. and Williams P.H. and Chapple I.L.C. | 2 |
| Periodontal pathogens in periodontal pockets and in carotid atheromatous plaques. | Romano, F. and Barbui, A. and Atmetti, M. | 2 |
| Adhesion of Streptococcus mutans to various dental materials in a laminar flow chamber system | Rosentritt M. and Hahnel S. and Groger G. and Muhlfriedel B. and Burgers R. and Handel G. | 2 |
| Nitrate reduction capacity of the oral microbiota is impaired in periodontitis: potential implications for systemic nitric oxide availability | Rosier B. and Johnston W. and Carda-Dieguez M. and Simpson A. and Cabello-Yeves E. and Piela K. and Reilly R. and Artacho A. and Easton C. and Burleigh M. and Culshaw S. and Mira A. AO - Rosier, Bob; ORCID: https://orcid.org/0000-0002-3267-6561 AO - Carda-Diegu... | 2 |
| Nitrate as a potential prebiotic for the oral microbiome. | Rosier, B T and Buetas, E and Moya-Gonzalvez, E M and Artacho, A and Mira, Alex | 2 |
| Killing activity of LFchimera on periodontopathic bacteria and multispecies oral biofilm formation in vitro | Ruangcharoen S. and Suwannarong W. and Lachica M.R.C.T. and Bolscher J.G.M. and Nazmi K. and Khunkitti W. and Taweechaisupapong S. AO - Taweechaisupapong, Suwimol; ORCID: https://orcid.org/0000-0001-604... | 2 |
| Effect of feeding a daily oral care chew on the composition of plaque microbiota in dogs | Ruparell A. and Warren M. and Staunton R. and Deusch O. and Dobenecker B. and Wallis C. and O'Flynn C. and McGenity P. and Holcombe L.J. | 2 |
| Comparison of subgingival and gingival margin plaque microbiota from dogs with healthy gingiva and early periodontal disease. | Ruparell, Avika and Wallis, Corrin and Haydock, Richard and Cawthrow, Amy and Holcombe, Lucy J | 2 |
| Optical approach to evaluating the effects of a novel dental gel on oral biofilm | Sabokpey S. and Biren-Fetz J. and Krasieva T.B. and Dadkhah M. and Chung N.E. and Ajdaharian J. and Wink C. and Wilder-Smith P. | 2 |
| Opsonophagocytic effect of antibody against recombinant conserved 40-kDa outer membrane protein of Porphyromonas gingivalis | Saito S. and Hayakawa M. and Takiguchi H. and Abiko Y. | 2 |
| Detection of Treponema socranskii associated with human periodontitis by PCR | Sakamoto M. and Takeuchi Y. and Umeda M. and Ishikawa I. and Benno Y. and Nakase T. | 2 |
| Evaluation and comparison of antibacterial efficacy of herbal extracts in combination with antibiotics on periodontal pathobionts: an in vitro microbiological study. | Saquib, S. A. and AlQahtani, N. A. and Irfan Ahmad, Irfan Ahmad and Kader, M. A. and Al-Shahrani, S. S. and Asiri, E. A. | 2 |
| The efficacy of tinidazole in naturally occurring periodontitis in dogs: Bacteriological and clinical results | Sarkiala E.M. and Asikainen S.E.A. and Kanervo A. and Junttila J. and Jousimies-Somer H.R. | 2 |
| Associations between oral conditions and respiratory disease in a national sample survey population | Scannapieco F.A. and Papandonatos G.D. and Dunford R.G. | 2 |
| In vitro efficacy of an amine fluoride + zinc lactate containing mouthwash | Schaeffer-Korbylo L. and Yang Y. | 2 |
| Filifactor alocis--involvement in periodontal biofilms. | Schlafer, Sebastian and Riep, Birgit and Griffen, Ann L and Petrich, Annett and Hubner, Julia and Berning, Moritz and Friedmann, Anton and Gobel, Ulf B and Moter, Annette | 2 |
| The effectiveness of root debridement in open flap procedures by means of a comparison between hand instruments and diamond burs. A SEM study. | Schwarz, J P and Guggenheim, R and Duggelin, M and Hefti, A F and Rateitschak-Pluss, E M and Rateitschak, K H | 2 |
| A sensitive enzymatic method (SK-013) for detection of Treponema denticola, Porphyromonas gingivals and Bacteroides forsythus in subgingival plaque samples | Seida K. and Saito A. and Yamada S. and Ishihara K. and Naito Y. and Okuda K. | 2 |
| The effect of a mouth rinse containing phenolic compounds on plaque formation and developing gingivitis | Sekino S. and Ramberg P. | 2 |
| Microbiological and clinical effects of a proanthocyanidin-enriched extract from Rumex Acetosa in periodontally healthy carriers of Porphyromonas gingivalis: a randomized controlled pilot study. | Selbach, Sabine and Klocke, Astrid and Peters, Ulrike and Beckert, Sabine and Watt, Rory Munro and Tong, Raymond and Flemmig, Thomas Frank and Hensel, Andreas and Beikler, Thomas | 2 |
| Relationship between periodontal infections and systemic disease | Seymour G.J. and Ford P.J. and Cullinan M.P. and Leishman S. and Yamazaki K. | 2 |
| Investigating the biological properties of carbohydrate derived fulvic acid (CHD-FA) as a potential novel therapy for the management of oral biofilm infections | Sherry L. and Millhouse E. and Lappin D.F. and Murray C. and Culshaw S. and Nile C.J. and Ramage G. | 2 |
| Comparison of Periodontal Bacteria of Edo and Modern Periods Using Novel Diagnostic Approach for Periodontitis With Micro-CT | Shiba T. and Komatsu K. and Sudo T. and Sawafuji R. and Saso A. and Ueda S. and Watanabe T. and Nemoto T. and Kano C. and Nagai T. and Ohsugi Y. and Katagiri S. and Takeuchi Y. and Kobayashi H. and Iwata T. | 2 |
| Lethal photosensitization in microbiological treatment of ligature-induced peri-implantitis: a preliminary study in dogs | Shibli J.A. and Martins M.C. and Theodoro L.H. and Lotufo R.F. and Garcia V.G. and Marcantonio E.J. | 2 |
| Antibody and T Cell Responses to Fusobacterium nucleatum and Treponema denticola in Health and Chronic Periodontitis | Shin J. and Kho S.-A. and Choi Y.S. and Kim Y.C. and Rhyu I.-C. and Choi Y. | 2 |
| The effect of supragingival plaque control on the composition of the subgingival microbial flora in ligature-induced periodontitis in the monkey. | Siegrist, B and Kornman, K S | 2 |
| Efficacy of photodynamic therapy on inflammatory signs and two selected periodontopathogenic species in a beagle dog model | Sigusch B.W. and Pfitzner A. and Albrecht V. and Glockmann E. | 2 |
| Genotypic variations of Porphyromonas gingivalis in chronic periodontitis patients with and without diabetes: an in vitro study using arbitrarily primed-polymerase chain reaction and heteroduplex-polymerase chain reaction. | Siladitya Sen, Siladitya Sen and Anand, K. M. | 2 |
| Characterization of progressive periodontal lesions in chronic periodontitis patients: Levels of chemokines, cytokines, matrix metalloproteinase-13, periodontal pathogens and inflammatory cells | Silva N. and Dutzan N. and Hernandez M. and Dezerega A. and Rivera O. and Aguillon J.C. and Aravena O. and Lastres P. and Pozo P. and Vernal R. and Gamonal J. | 2 |
| Subgingival bacterial microbiota associated with ovine periodontitis1 | Silva N.S. and Borsanelli A.C. and Gaetti-Jardim E. and Schweitzer C.M. and Silveira J.A.S. and Bomjardim H.A. and Dutra I.S. and Barbosa J.D. | 2 |
| Analytical performance of an immunologic-based periodontal bacterial test for simultaneous detection and differentiation of Actinobacillus actinomycetemcomitans, Porphyromonas gingivalis, and Prevotella intermedia | Snyder B. and Ryerson C.C. and Corona H. and Grogan E.A. and Reynolds H.S. and Contestable P.B. and Boyer B.P. and Mayer J. and Mangan T. and Norkus N. and Zambon J.J. and Genco R.J. | 2 |
| Effects of azithromycin, metronidazole, amoxicillin, and metronidazole plus amoxicillin on an in vitro polymicrobial subgingival biofilm model. | Soares, G. M. S. and Teles, F. and Starr, J. R. and Feres, M. and Patel, M. and Martin, L. and Teles, R. | 2 |
| Use of checkerboard DNA-DNA hybridization to study complex microbial ecosystems | Socransky, S. S. and Haffajee, A. D. and Smith, C. and Martin, L. and Haffajee, J. A. and Uzel, N. G. and Goodson, J. M. | 2 |
| Strong antibacterial effect of miswak against oral microorganisms associated with periodontitis and caries. | Sofrata, Abier H and Claesson, Rolf L K and Lingstrom, Peter K and Gustafsson, Anders K | 2 |
| Innate immune responses of exfoliated epithelial cells. (P4165) | Srinivasan M. and Swaminathan V. and Negrini T. and Arthur R. | 2 |
| Activity of pradofloxacin against Porphyromonas and Prevotella spp. implicated in periodontal disease in dogs: Susceptibility test data from a European Multicenter Study | Stephan B. and Greife H.A. and Pridmore A. and Silley P. | 2 |
| Adherence of Porphyromonas (Bacteroides) gingivalis to Streptococcus sanguis in vitro | Stinson M.W. and Safulko K. and Levine M.J. | 2 |
| In-Vitro Inhibitory Efficacy of 3 Types of Probiotics on the Growth of Aggregatibacter actinomycetemcomitans Bacteria | Sulistiowati C.P. and Suhartono M. and Rahmawati D.F. and Ulfah N. and Supandi S.K. and Wijaksana I.K.E. and Abullais S.S. and Dhadse P. | 2 |
| Low levels of caries in aggressive periodontitis: A literature review | Sulugodu Ramachandra S. | 2 |
| A pilot study on antiplaque effects of mastic chewing gum in the oral cavity | Takahashi K. and Fukazawa M. and Motohira H. and Ochiai K. and Nishikawa H. and Miyata T. | 2 |
| Involvement of adhesion molecule in in vitro plaque-like formation of macrophages stimulated with Aggregatibacter actinomycetemcomitans lipopolysaccharide | Takeshi T. and Keisuke N. and Takaaki I. and Makoto Y. and Tatsuji N. | 2 |
| Massively Increased Caries Susceptibility in an Irf6 Cleft Lip/Palate Model | Tamasas B. and Cox T.C. | 2 |
| Subgingival and tongue microbiota during early periodontitis. | Tanner, A C R and Paster, B J and Lu, S C and Kanasi, E and Kent, R Jr and Van Dyke, T and Sonis, S T | 2 |
| Periodontal bacterial DNA suppresses the immune response to mutans streptococcal glucosyltransferase | Taubman M.A. and Han X. and LaRosa K.B. and Socransky S.S. and Smith D.J. | 2 |
| Effects of Streblus asper leaf extract on the biofilm formation of subgingival pathogens. | Taweechaisupapong, S. and Pinsuwan, W. and Suwannarong, W. and Kukhetpitakwong, R. and Luengpailin, S. | 2 |
| Viruses of the oral cavity: Prevalence, pathobiology and association with oral diseases | Thakkar P. and Banks J.M. and Rahat R. and Brandini D.A. and Naqvi A.R. AO - Naqvi, Afsar R.; ORCID: https://orcid.org/0000-0001-7436-3056 | 2 |
| Association of gingivitis with dental calculus thickness or dental calculus coverage and subgingival bacteria in feline leukemia virus-and feline immunodeficiency virus-negative cats | Thengchaisri N. and Steiner J.M. and Suchodolski J.S. and Sattasathuchana P. | 2 |
| EVALUATION AND COMPARISON OF ANTIBACTERIAL EFFICACY OF ALBIZIA LEBBECK (L.) BENTH., BAUHINIA VARIEGATA L. AND CHLORHEXIDINE MOUTHWASHES: IN-VITRO STUDY | Thomas M. and Shettar L. and Agnihotri P. and Bhat K. | 2 |
| Characterization of monoclonal antibodies for rapid identification of Actinomyces naeslundii in clinical samples | Thurnheer T. and Guggenheim B. and Gmur R. | 2 |
| Antibody titer against porphyromonas gingivalis in rats with experimentally induced periodontitis. | Torkzaban, P. and Zamani, A. and Yousefimashouf, R. and Faradmal, J. and Hedayatipanah, M. and Karimitabar, Z. | 2 |
| Improved multiplex PCR using conserved and species-specific 16S rRNA gene primers for simultaneous detection of Actinobacillus actinomycetemcomitans, Bacteroides forsythus, and Porphyromonas gingivalis | Tran S.D. and Rudney J.D. | 2 |
| The neutrophil elastase-upregulated placenta growth factor promotes the pathogenesis and progression of periodontal disease | Tseng H.-Y. and Chen Y.-W. and Lee B.-S. and Chang P.-C. and Wang Y.-P. and Lin C.-P. and Cheng S.-J. and Kuo M.Y.-P. and Hou H.-H. AO - Chen, Yi-Wen; ORCID: https://orcid.org/0000-0002-9008-8346 | 2 |
| Butyrate, a bacterial metabolite, induces apoptosis and autophagic cell death in gingival epithelial cells | Tsuda H. and Ochiai K. and Suzuki N. and Otsuka K. | 2 |
| The distribution of periodontopathic bacteria among Japanese children and their parents | Umeda M. and Miwa Z. and Takeuchi Y. and Ishizuka M. and Huang Y. and Noguchi K. and Tanaka M. and Takagi Y. and Ishikawa I. | 2 |
| Antibacterial effects of some herbal extracts against oral pathogens | Vahabi S. and Najafi E. and Alizadeh A. | 2 |
| In vitro antimicrobial effects of some herbal essences against oral pathogens | Vahabi S. and Najafi E. and Alizadeh S. | 2 |
| Towards defining the outer membrane proteome of Porphyromonas gingivalis | Veith P.D. and Gorasia D.G. and Reynolds E.C. AO - Reynolds, Eric C.; ORCID: https://orcid.org/0000-0002-6618-4856 | 2 |
| Fusobacterium nucleatum alters atherosclerosis risk factors and enhances inflammatory markers with an atheroprotective immune response in ApoEnull mice | Velsko I.M. and Chukkapalli S.S. and Rivera-Kweh M.F. and Chen H. and Zheng D. and Bhattacharyya I. and Gangula P.R. and Lucas A.R. and Kesavalu L. | 2 |
| The apical border plaque in chronic adult periodontitis. An ultrastructural study. I. Morphology, structure, and cell content | Vrahopoulos T.P. and Barber P.M. and Newman H.N. | 2 |
| Simultaneous detection of Actinobacillus actinomycetemcomitans and Porphyromonas gingivalis by a rapid PCR method | Wahlfors J. and Meurman J.H. and Vaisanen P. and Alakuijala P. and Korhonen A. and Torkko H. and Janne J. | 2 |
| Periodontitis, periodontopathic bacteria and lactoferrin. | Wakabayashi, Hiroyuki and Kondo, Ichiro and Kobayashi, Tetsuo and Yamauchi, Koji and Toida, Tomohiro and Iwatsuki, Keiji and Yoshie, Hiromasa | 2 |
| Subgingival microbiota of dogs with healthy gingiva or early periodontal disease from different geographical locations | Wallis C. and Milella L. and Colyer A. and O'Flynn C. and Harris S. and Holcombe L.J. AO - Wallis C.; ORCID: https://orcid.org/0000-0003-2260-7279 | 2 |
| Bacterial composition changes in canine plaque over periodontal disease severity and daily care practices | Watanabe A. and Okada J. and Niwa R. and Inui Y. and Ito K. and Shimokawa Y. and Kihira M. AO - Watanabe, Ayano; ORCID: https://orcid.org/0009-0004-3077-5859 AO - Kihir... | 2 |
| Endogenously citrullinated proteins from porphyromonas gingivalis as a candidate source of priming autoantigens in rheumatoid arthritis | Wegner N. and Eick S. and Sroka A. and Nguyen K.-A. and Potempa J. and Venables P. | 2 |
| IgG subclass antibodies to Porphyromonas gingivalis in patients with destructive periodontal disease. A case: control study | Wilton J.M. and Hurst T.J. and Austin A.K. | 2 |
| The Potential of 24-Propylcholestrol as Antibacterial Oral Bacteria of Enterococcus faecalis ATCC 29212 and Inhibitor Biofilms Formation: in vitro and in silico Study. | Windaryanti, Devi and Gabriel, Christine Sondang and Hidayat, Ika Wiani and Zainuddin, Achmad and Dharsono, Hendra Dian Adhita and Satari, Mieke Hemiawati and Kurnia, Dikdik | 2 |
| Porphyromonas gingivalis HmuY protein as a potential marker of chronic periodontitis | Wojtowicz H. and Radwan-Oczko M. and Chomyszyn-Gajewska M. and Olczak T. | 2 |
| Recovery of periodontopathogenic bacteria from embalmed human cadavers. | Wood, Nelson and Johnson, Roger B | 2 |
| Effect of <ovid:i>Porphyromonas gingivalis</ovid:i> PrtC on cytokine expression in ECV304 endothelial cells and its level in subgingival plaques from patients with chronic periodontitis | Wu YanMin, Wu YanMin and Chen LiLi, Chen LiLi and Yan Jie, Yan Jie and Zhuang ChunYan, Zhuang ChunYan and Gu ZhiYuan, Gu ZhiYuan | 2 |
| Grape products and oral health. | Wu, C. D. and Pezzuto, J. M. and Venkatasubramanian, V. and Hamad, M. and Morris, K. R. | 2 |
| Enhancing production of herpes simplex virus type 1 in oral epithelial cells by co-infection with Aggregatibacter actinomycetemcomitans | Wu, Ching-Yi and Yu, Zhu-Yun and Hsu, Yu-Chun and Hung, Shan-Ling | 2 |
| Effect of Porphyromonas gingivalis PrtC on cytokine expression in ECV304 endothelial cells and its level in subgingival plaques from patients with chronic periodontitis | Wu, Yan-min and Chen, Li-li and Yan, Jie and Zhuang, Chun-yan and Gu, Zhi-yuan | 2 |
| Detection of bacterial diversity in rat's periodontitis model under imitational altitude hypoxia environment | Xiao X. and Li Y. and Zhang G. and Gao Y. and Kong Y. and Liu M. and Tan Y. | 2 |
| Assosiation of inflammasome in periodontal desease and atherosclerosisinduced by porphyromonas gingivalis oral infection | Yamaguchi Y. and Ochiai T. and Hashizume T. and Kobayashi R. and Ando T. | 2 |
| Distribution of periodontopathic bacterial species in dogs and their owners | Yamasaki Y. and Nomura R. and Nakano K. and Naka S. and Matsumoto-Nakano M. and Asai F. and Ooshima T. | 2 |
| Effects of panduratin A isolated from Kaempferia pandurata ROXB. on the expression of matrix metalloproteinase-9 by Porphyromonas gingivalis supernatant-induced KB cells. | Yanti, Yanti and Oh HyunIn, Oh HyunIn and Anggakusuma, Anggakusuma and Hwang JaeKwan, Hwang JaeKwan | 2 |
| Involvement of a periodontal pathogen, Porphyromonas gingivalis on the pathogenesis of non-alcoholic fatty liver disease. | Yoneda, Masato and Naka, Shuhei and Nakano, Kazuhiko and Wada, Koichiro and Endo, Hiroki and Mawatari, Hironori and Imajo, Kento and Nomura, Ryota and Hokamura, Kazuya and Ono, Masafumi and Murata, Shogo and Tohnai, Iwai and Sumida, Yoshio and Shima, Toshihide and Kuboniwa, Masae and Umemura, Kazuo and Kamisaki, Yoshinori and Amano, Atsuo and Okanoue, Takeshi and Ooshima, Takashi and Nakajima, Atsushi | 2 |
| Evidence supporting oral hygiene management by owners through a genetic analysis of dental plaque bacteria in dogs | Yu, Jeong Suk and Kim, Minhee and Cho, Il-Hoon and Sim, Yu-Min and Hwang, Young Sun | 2 |
| Porphyromonas gingivalis infection accelerates intimal thickening in iliac arteries in a balloon-injured rabbit model | Zhang M.-Z. and Li C.-L. and Jiang Y.-T. and Jiang W. and Sun Y. and Shu R. and Liang J.-P. | 2 |
| The virulence factor GroEL promotes gelatinase secretion from cells in the osteoblast lineage: Implication for direct crosstalk between bacteria and adult cells | Zhang, L. and Cui, Y. and Yang, Y. and Wei, J. and Liu, W. and Cai, L. and Wang, L. and Zhang, D. and Xie, J. and Cheng, L. | 2 |
| Transcriptome analysis of Fusobacterium nucleatum reveals differential gene expression patterns in the biofilm versus planktonic cells | Zhao T. and Chen J. and Liu S. and Yang J. and Wu J. and Miao L. and Sun W. AO - Zhao, Tian; ORCID: https://orcid.org/0000-0002-4981-2099 | 2 |
| Periodontal Bacterial DNA and Their Link to Human Cardiac Tissue: Findings of a Pilot Study | Ziebolz D. and Rost C. and Schmidt J. and Waldmann-Beushausen R. and Schondube F.A. and Mausberg R.F. and Danner B.C. | 2 |
| Gum Periobalance(TM) tablets and chewing gum and oral health - scientific substantiation of a health claim related to Gum PeriobalanceTM tablets and chewing gum and oral health pursuant to Article 13(5) of Regulation (EC) No 1924/2006. | | 2 |
| Polymorphism of IL-4 (-590) and IL-6 (-174) is not associated with chronic periodontitis in Babylonian population | Abd F.G. | 3 |
| Modification of cystatin C activity by bacterial proteinases and neutrophil elastase in periodontitis | Abrahamson M. and Wikstrom M. and Potempa J. and Renvert S. and Hall A. | 3 |
| Synergistic antibacterial activity of herbal extracts with antibiotics on bacteria responsible for periodontitis. | Abullais Saquib, Shahabe and Abdullah AlQahtani, Nabeeh and Ahmad, Irfan and Arora, Suraj and Mohammed Asif, Shaik and Ahmed Javali, Mukhatar and Nisar, Nazima | 3 |
| Assessment of oral and overall health parameters using the SillHa Oral Wellness System | Adibi S.S. and Hanson R. and Fray D.F. and Abedi T. and Neil B. and Maher D. and Tribble G. and Warner B.F. and Farach-Carson M.C. | 3 |
| Darkfield microscopy of the flora of subgingival plaque of patients with severe periodontitis and its use in therapeutic assessment. | Africa, C W and Parker, J R and Reddy, J | 3 |
| NOX1/2 activation in human gingival fibroblasts by Fusobacterium nucleatum facilitates attachment of Porphyromonas gingivalis | Ahn S.H. and Song J.-E. and Kim S. and Cho S.-H. and Lim Y.K. and Kook J.-K. and Kook M.-S. and Lee T.-H. | 3 |
| Effect of gingival inflammation on the inflammatory response in patients with idiopathic uveitis. | Akcali, A. and Yilmaz, S. G. and Lappin, D. F. and EgrIlmez, S. and BudunelI, N. | 3 |
| Screening of interleukin 17F gene polymorphisms and eight subgingival pathogens in chronic periodontitis in Libyan patients | Alsherif E. and Alhudiri I. and ElJilani M. and Ramadan A. and Rutland P. and Elzagheid A. and Enattah N. | 3 |
| Immune response to cytolethal distending toxin of Aggregatibacter actinomycetemcomitans in periodontitis patients. | Ando, E. S. and De-Gennaro, L. A. and Faveri, M. and Feres, M. and DiRienzo, J. M. and Mayer, M. P. A. | 3 |
| Increased heart failure prevalence in patients with a high antibody level against periodontal pathogen | Aoyama N. and Kure K. and Minabe M. and Izumi Y. | 3 |
| Clinical associations between IL-17 family cytokines and periodontitis and potential differential roles for IL-17A and IL-17E in periodontal immunity. | Awang, Raja Azman and Lappin, David F and MacPherson, Alexandrea and Riggio, Marcello and Robertson, Douglas and Hodge, Penny and Ramage, Gordon and Culshaw, Shauna and Preshaw, Philip M and Taylor, John and Nile, Christopher | 3 |
| Interleukin-6 production by cultured peripheral blood monocytes before and after stimulation by E. coli lipopolysaccharide in Iranian patients with aggressive periodontitis | Bajestan M.N. and Radvar M. and Afshari J.T. and Naseh M.R. and Arab H.R. | 3 |
| Association of the CD14 -260C/T polymorphism with plaque-induced gingivitis depends on the presence of Porphyromonas gingivalis | Bartosova M. and Borilova Linhartova P. and Musilova K. and Broukal Z. and Kukletova M. and Kukla L. and Izakovicova Holla L. AO - Izakovicova Holla, Lydie; ORCID: https://orcid.org/0000-0002-7610-... | 3 |
| H2S mediates increased interleukin (IL)-1beta and IL-18 production in leukocytes from patients with periodontitis | Basic A. and Serino G. and Leonhardt A. and Dahlen G. AO - Basic, Amina; ORCID: https://orcid.org/0000-0003-2217-3642 | 3 |
| Hydrogen sulfide production from subgingival plaque samples. | Basic, A and Dahlen, G | 3 |
| Cytokine profile changes in gingival crevicular fluid after placement different brackets types | Bergamo A.Z.N. and Nelson-Filho P. and do Nascimento C. and Casarin R.C.V. and Casati M.Z. and Andrucioli M.C.D. and Kuchler EC. and Longo D.L. and da Silva L.A.B. and Matsumoto M.A.N. | 3 |
| The prevalence of human herpes viruses in the saliva of chronic periodontitis patients compared to oral health providers and healthy controls. | Bilder, L. and Elimelech, R. and Szwarcwort-Cohen, M. and Kra-Oz, Z. and Machtei, E. E. | 3 |
| Passive immunization with monoclonal antibodies against Porphyromonas gingivalis in patients with periodontitis | Booth V. and Ashley F.P. and Lehner T. | 3 |
| Application of label-free absolute quantitative proteomics in human gingival crevicular fluid by LC/MSE (Gingival Exudatome) | Bostanci N. and Heywood W. and Mills K. and Parkar M. and Nibali L. and Donos N. | 3 |
| Colonization by Actinobacillus actinomycetemcomitans, Porphyromonas gingivalis and Prevotella intermedia in adult periodontitis patients as detected by the antibody-based Evalusite Test | Boyer B.P. and Ryerson C.C. and Reynolds H.S. and Zambon J.J. and Genco R.J. and Snyder B. | 3 |
| Impact of Periodontitis on the Leakage of Oral Bacteria to the Gut | Buetas, E. and Jordan-Lopez, M. and Lopez-Roldan, A. and Mira, A. and Carda-Dieguez, M. | 3 |
| Aggregatibacter actinomycetemcomitans in African Americans with Localized Aggressive Periodontitis | Burgess D. and Huang H. and Harrison P. and Aukhil I. and Shaddox L. | 3 |
| Immunoassay standardization for the detection of immunoglobulin a (IgA) against Porphyromonas gingivalis antigens in saliva of individuals with and without leprosy. | Calheira, Mariana Costa and Trindade, Soraya Castro and Falcao, Michelle Miranda Lopes and Barbosa, Luciana Sales Conceicao and Carvalho, Gislene Regina Batista and Machado, Paulo Roberto Lima and Gomes Filho, Isaac Suzart and Campos, Elisangela de Jesus and de Carvalho Filho, Paulo Cirino and Xavier, Marcia Tosta and de Farias, Antonio Pedro Froes and Rocha Filho, Jose Tadeu Raynal and Passos-Soares, Johelle de Santana | 3 |
| Comparative Analyses of Subgingival Microbiome in Chronic Periodontitis Patients with and Without IgA Nephropathy by High Throughput 16S rRNA Sequencing. | Cao, Yali and Qiao, Min and Tian, Zhigang and Yu, Yan and Xu, Baohua and Lao, Wansheng and Ma, Xuguo and Li, Wenge | 3 |
| Periodontal Bacteria in the Genital Tract: Are They Related to Adverse Pregnancy Outcome? | Cassini, M. A. and Pilloni, A. and Condo, S. G. and Vitali, L. A. and Pasquantonio, G. and Cerroni, L. | 3 |
| Genetic association with subgingival bacterial colonization in chronic periodontitis | Cavalla F. and Biguetti C.C. and Lima Melchiades J. and Tabanez A.P. and de Campos Soriani Azevedo M. and Favaro Trombone A.P. and Faveri M. and Feres M. and Pompermaier Garlet G. | 3 |
| Chemiluminescent assay of alkaline phosphatase in human gingival crevicular fluid: investigations with an experimental gingivitis model and studies on the source of the enzyme within crevicular fluid. | Chapple, I L and Socransky, S S and Dibart, S and Glenwright, H D and Matthews, J B | 3 |
| Effect of an essential oil-containing dentifrice on dental plaque microbial composition. | Charles, C H and Vincent, J W and Borycheski, L and Amatnieks, Y and Sarina, M and Qaqish, J and Proskin, H M | 3 |
| C1q and C4 detection on bacterial plaque organisms. | Chisikovsky, J and Toto, P D and Gargiulo, A W | 3 |
| Clinical and microbial evaluation of the effects on gingivitis of a mouth rinse containing an Enteromorpha linza extract. | Cho HanBin, Cho HanBin and Lee HeeHyun, Lee HeeHyun and Lee OkHwan, Lee OkHwan and Choi, H. S. and Choi JaeSuk, Choi JaeSuk and Lee BooYong, Lee BooYong | 3 |
| Quantitative detection of Staphylococcus aureus, Enterococcus faecalis and Pseudomonas aeruginosa in human oral epithelial cells from subjects with periodontitis and periodontal health. | Colombo, Andrea V and Barbosa, Graziela M and Higashi, Daniela and di Micheli, Giorgio and Rodrigues, Paulo H and Simionato, Maria Regina L | 3 |
| Effect of salivary urea, pH and ureolytic microflora on dental calculus formation and its correlation with periodontal status. | D'souza, Liberia L and Lawande, Sandeep A and Samuel, James and Wiseman Pinto, Maria Jose | 3 |
| Circulating antibodies against leukotoxin A as marker of periodontitis grades B and C and oral infection with Aggregatibacter actinomycetemcomitans. | Damgaard, Christian and Danielsen, Anne Katrine and Enevold, Christian and Reinholdt, Jesper and Holmstrup, Palle and Nielsen, Claus H. and Massarenti, Laura | 3 |
| Real-time polymerase chain reaction to determine the prevalence and copy number of Epstein-Barr virus and cytomegalovirus DNA in subgingival plaque at individual healthy and periodontal disease sites. | Dawson, D. R., III and Wang, C. M. and Danaher, R. J. and Lin, Y. S. and Kryscio, R. J. and Jacob, R. J. and Miller, C. S. | 3 |
| Salivary AST, ALP and CK levels in patients with periodontitis | Deepika V. and Vishnu Priya V. and Bedre A. and Harsha L. | 3 |
| Levels of pro- and anti-inflammatory cytokines in cystic fibrosis patients with or without gingivitis | Duruel O. and Berker E. and Ozsin-Ozler C. and Gharibzadeh-Hizal M. and Gurpinar O. and Eryilmaz-Polat S. and Ataman-Duruel E.T. and Tan C. and Karabulut E. and Tekcicek M. and Koseoglu-Eser O. and Kiper N. and Tezcan I. | 3 |
| Longitudinal dynamics of infection and serum antibody in A. actinomycetemcomitans periodontitis | Ebersole J.L. and Capelli D. and Steffen M.J. | 3 |
| Human serum antibody responses to oral microorganisms. IV. Correlation with homologous infection | Ebersole J.L. and Taubman M.A. and Smith D.J. and Frey D.E. and Haffajee A.D. and Socransky S.S. | 3 |
| Local antibody responses in periodontal diseases. | Ebersole, J L and Taubman, M A and Smith, D J | 3 |
| 3-Hydroxy fatty acids in saliva as diagnostic markers in chronic periodontitis | Ferrando R. and Szponar B. and Sanchez A. and Larsson L. and Valero-Guillen P.L. | 3 |
| Macrophage inflammatory protein-1alpha shows predictive value as a risk marker for subjects and sites vulnerable to bone loss in a longitudinal model of aggressive periodontitis | Fine D.H. and Markowitz K. and Fairlie K. and Tischio-Bereski D. and Ferrandiz J. and Godboley D. and Furgang D. and Gunsolley J. and Best A. | 3 |
| Macrophage inflammatory protein-1 alpha : a salivary biomarker of bone loss in a longitudinal cohort study of children at risk for aggressive periodontal disease? | Fine, D. H. and Markowitz, K. and Furgang, D. and Fairlie, K. and Ferrandiz, J. and Nasri, C. and McKiernan, M. and Donnelly, R. and Gunsolley, J. | 3 |
| Inflammation, heat shock proteins and periodontal pathogens in atherosclerosis: An immunohistologic study | Ford P.J. and Gemmell E. and Chan A. and Carter C.L. and Walker P.J. and Bird P.S. and West M.J. and Cullinan M.P. and Seymour G.J. | 3 |
| Effects of initial periodontal therapy on heat shock protein 70 levels in gingival crevicular fluid from periodontitis patients | Furuse N. and Takai H. and Ogata Y. | 3 |
| The genus Weissella: Taxonomy, ecology and biotechnological potential | Fusco V. and Quero G.M. and Cho G.-S. and Kabisch J. and Meske D. and Neve H. and Bockelmann W. and Franz C.M.A.P. | 3 |
| Distribution of biotypes and leukotoxic activity of Aggregatibacter actinomycetemcomitans isolated from Brazilian patients with chronic periodontitis. | Gaetti-Jardim Junior, E. and Wahasugui, T. C. and Tomazinho, P. H. and Marques, M. M. and Nakano, V. and Avila-Campos, M. J. | 3 |
| Effect of Trivrit Virechana, Yashtyadi Pratisarana and Panchaksheeri Vriksha Kashaya Gandoosha in Periodontitis - A Case Series | Gangadharan N. and Sivabalaji K. and Ashwini B.N. | 3 |
| Periodontitis diagnostics on the basis of saliva raman spectroscopy | Gonchukov S.A. and Sukhinina A.V. | 3 |
| Effect of herbal, essential oil, and chlorhexidine mouthrinses on the composition of the subgingival microbiota and clinical periodontal parameters. | Haffajee, Anne D and Roberts, Christine and Murray, Lora and Veiga, Nancy and Martin, Lynn and Teles, Ricardo P and Letteri, Marie and Socransky, Sigmund S | 3 |
| Total IgA and Porphyromonas gingivalis-reactive IgA in the saliva of patients with generalised early-onset periodontitis | Hagewald S. and Bernimoulin J.P. and Kottgen E. and Kage A. | 3 |
| Salivary IgA subclasses and bacteria-reactive IgA in patients with aggressive periodontitis | Hagewald S. and Bernimoulin J.P. and Kottgen E. and Kage A. | 3 |
| Assessing the use of Quantitative Light-induced Fluorescence-Digital as a clinical plaque assessment | Han S.-Y. and Kim B.-R. and Ko H.-Y. and Kwon H.-K. and Kim B.-I. | 3 |
| Antibiotic resistance in bacteria isolated from subgingival plaque in a Norwegian population with refractory marginal periodontitis | Handal T. and Caugant D.A. and Olsen I. | 3 |
| Detection and characterization of beta-lactamase genes in subgingival bacteria from patients with refractory periodontitis. | Handal, Trude and Olsen, Ingar and Walker, Clay B and Caugant, Dominique A | 3 |
| Comparative Analysis of Calcium-Binding Myeloid-Related Protein-8/14 in Saliva and Serum of Patients With Periodontitis and Healthy Individuals | Haririan H. and Andrukhov O. and Pablik E. and Neuhofer M. and Moritz A. and Rausch-Fan X. | 3 |
| Associations between systemic status, periodontal status, serum cytokine levels, and delivery outcomes in pregnant women with a diagnosis of threatened premature labor. | Hasegawa, K. and Furuichi, Y. and Shimotsu, A. and Nakamura, M. and Yoshinaga, M. and Kamitomo, M. and Hatae, M. and Maruyama, I. and Izumi, Y. | 3 |
| Enzymatic measurement of short-chain fatty acids and application in periodontal disease diagnosis | Hatanaka K. and Shirahase Y. and Yoshida T. and Kono M. and Toya N. and Sakasegawa S.-I. and Konishi K. and Yamamoto T. and Ochiai K. and Takashiba S. | 3 |
| Cross-sectional analysis of risk factors for subclinical periodontitis; active matrix metalloproteinase-8 as a potential indicator in initial periodontitis in adolescents | Heikkinen A.M. and Raisanen I.T. and Tervahartiala T. and Sorsa T. | 3 |
| Interferon-gamma +874A/T polymorphism in relation to generalized chronic periodontitis and the presence of periodontopathic bacteria | Holla L.I. and Hrdlickova B. and Linhartova P. and Fassmann A. | 3 |
| Association of Toll-like receptor 9 haplotypes with chronic periodontitis in Czech population. | Holla, Lydie Izakovicova and Vokurka, Jan and Hrdlickova, Barbara and Augustin, Peter and Fassmann, Antonin | 3 |
| Salivary collagenase, elastase- and trypsin-like proteases as biochemical markers of periodontal tissue destruction in adult and localized juvenile periodontitis | Ingman T. and Sorsa T. and Konttinen Y.T. and Liede K. and Saari H. and Lindy O. and Suomalainen K. | 3 |
| Salivary leukocyte esterase activity by SillHa is a risk indicator of periodontal disease | Ishii K. and Venkataiah V.S. and Kajiwara T. and Umezawa K. and Suzuki S. and Nakano M. and Sawaguchi M. and Yahata Y. and Saito M. | 3 |
| Salivary levels of azurocidin and soluble azurophilic granules in periodontal disease | Jasim, F. S. and Al-Ghurabi, B. H. and Abdulameer, L. A. | 3 |
| Periodontal Dressing as an Adjunct after Scaling and Root Planing--A Useful Preventive Tool? | Jentsch H.F. and Knofler G.U. and Purschwitz R.E. and Eick S. | 3 |
| Soluble CD14 levels in gingival crevicular fluid of subjects with untreated adult periodontitis | Jin L. and Darveau R.P. | 3 |
| Interleukin-8 and granulocyte elastase in gingival crevicular fluid in relation to periodontopathogens in untreated adult periodontitis | Jin L. and Soder B. and Corbet E.F. | 3 |
| Relationship of changes in interleukin-8 levels and granulocyte elastase activity in gingival crevicular fluid to subgingival periodontopathogens following non-surgical periodontal therapy in subjects with chronic periodontitis | Jin, L. J. and Leung, W. K. and Corbet, E. F. and Soder, B. | 3 |
| BANA-Positive Plaque Samples Are Associated with Oral Hygiene Practices and Not CD4+ T Cell Counts in HIV-Positive Patients. | John, Cathy Nisha and Xavier Graham Stephen, Lawrence and Wilma Joyce Africa, Charlene | 3 |
| Type a behavior pattern and perceived stress are associated with more severe periodontal disease | Jungo S.J. and Consoli S.M. and Guez D.M. | 3 |
| Human monoclonal antibody inhibits porphyromonas gingivalis hemagglutinin activity | Kaizuka K. and Hosogi Y. and Hayakawa M. and Shibata Y. and Abiko Y. | 3 |
| Chemokine Receptor 2 (CXCR2) Gene Variants and Their Association with Periodontal Bacteria in Patients with Chronic Periodontitis. | Kavrikova, Denisa and Borilova Linhartova, Petra and Lucanova, Svetlana and Poskerova, Hana and Fassmann, Antonin and Izakovicova Holla, Lydie | 3 |
| Cytokines and chemokines are differentially expressed in patients with periodontitis: possible role for TGF- beta 1 as a marker for disease progression. | Khalaf, H. and Lonn, J. and Bengtsson, T. | 3 |
| Salivary thiol levels and periodontal parameters assessed with a chromogenic strip | Khocht A. and Seyedain M. and Hardan S. and Gaughan J. and Suzuki J.B. | 3 |
| Quantification of Epstein-Barr virus and human cytomegalovirus in chronic periodontal patients | Khosropanah H. and Karandish M. and Ziaeyan M. and Jamalidoust M. | 3 |
| Activation of toll-like receptors 2 and 4 by gram-negative periodontal bacteria | Kikkert R. and Laine M.L. and Aarden L.A. and Van Winkelhoff A.J. | 3 |
| Salivary IL-1s but not dietary flavonoids are associated with healing after periodontal therapy to prevent tooth loss | Klok S. and Yumol J. and Sparrow T. and Sullivan P. and Ward W. and Fritz P. | 3 |
| The interleukin-1 genotype as a severity factor in adult periodontal disease | Kornman K.S. and Crane A. and Wang H.Y. and di Giovine F.S. and Newman M.G. and Pirk F.W. and Wilson Jr. T.G. and Higginbottom F.L. and Duff G.W. | 3 |
| Application of lactoferrin and alpha 1-antitrypsin in gingival retention fluid to diagnosis of periodontal disease. | Koshi, R. and Kotani, K. and Ohtsu, M. and Yoshinuma, N. and Sugano, N. | 3 |
| Detection of <ovid:i>Tannerella forsythia bspA</ovid:i> and <ovid:i>prtH</ovid:i> genotypes among periodontitis patients and healthy subjects - a case - control study | Krishnan Mahalakshmi, Krishnan Mahalakshmi and Padma Krishnan, Padma Krishnan and Chandrasekaran, S. C. | 3 |
| CD70 Deficiency Associated With Chronic Epstein-Barr Virus Infection, Recurrent Airway Infections and Severe Gingivitis in a 24-Year-Old Woman | Kruger R. and Martin E. and Dmytrus J. and Feiterna-Sperling C. and Meisel C. and Unterwalder N. and Kolsch U. and Wahn V. and Hofmann J. and Korn P. and Latour S. and Boztug K. and von Bernuth H. | 3 |
| Identification of multiple strains of Porphyromonas gingivalis using heteroduplex polymerase chain reaction in varying severity of chronic periodontitis. | Kulkarni, M. K. and Bhat, K. G. and Thomas, B. S. and Bhat, G. S. and Kulkarni, R. D. | 3 |
| Genetic control of strong and weak global innate immunity to gingivitis may predict periodontitis severity | Levine M. and Ruksakiet K. and Foldes A. and Dinya E. and Lohinai Z.M. | 3 |
| Autoinducer-2 produced by oral microbial flora and alveolar bone loss in periodontitis | Li, C. and Zhou, H. and Gou, H. and Fan, Z. and Zhang, Y. and Tang, P. and Huang, J. and Xu, Y. and Li, L. | 3 |
| TLR4 polymorphisms may increase susceptibility to periodontitis in Pg-positive individuals. | Li, Wen-jing and Cao, Xiao-jing and He, Lu and Meng, Huan-xin and Yang, Bing-tao and Liao, Yan-ting | 3 |
| Artesunate Alleviates Kidney Fibrosis in Type 1 Diabetes with Periodontitis Rats via Promoting Autophagy and Suppression of Inflammation | Liang, Chen and Ma, Licheng and Chen, Yi and Li, Jiaquan and Wang, Binge and Ma, Chubin and Yuan, Zhong and Nong, Xiaolin | 3 |
| Radiographic and clinical signs of periodontitis and associated bacterial species in a Swedish adolescent population | Lindholm M. and Claesson R. and Lof H. and Chiang H.-M. and Oscarsson J. and Johansson A. and Aberg C.H. AO - Lindholm, Mark; ORCID: https://orcid.org/0000-0002-5797-6608 AO - Johan... | 3 |
| Haplotype analysis of interleukin-8 gene polymorphisms in chronic and aggressive periodontitis. | Linhartova, P. B. and Vokurka, J. and Poskerova, H. and Fassmann, A. and Holla, L. I. | 3 |
| Mannose-binding lectin gene polymorphism in relation to periodontal infection | Liukkonen A. and He Q. and Gursoy U.K. and Pussinen P.J. and Grondahl-Yli-Hannuksela K. and Liukkonen J. and Sorsa T. and Suominen A.L. and Huumonen S. and Kononen E. | 3 |
| Genetic Control of GCF Exudation: Innate Immunity Genes and Periodontitis Susceptibility | Lohinai Z.M. and Ruksakiet K. and Foldes A. and Dinya E. and Levine M. AO - Lohinai, Zsolt M.; ORCID: https://orcid.org/0000-0003-2695-5166 AO - Ruk... | 3 |
| Alkaline phosphatase as a periodontal disease marker | Malhotra R. and Grover V. and Kapoor A. and Kapur R. | 3 |
| Validation of a multiplex qPCR assay for the identification and quantification of <ovid:i>Aggregatibacter actinomycetemcomitans</ovid:i> and <ovid:i>Porphyromonas gingivalis</ovid:i>: <ovid:i>in vitro</ovid:i> and subgingival plaque samples | Marin, M. J. and Ambrosio, N. and Herrera, D. and Sanz, M. and Figuero, E. | 3 |
| Evaluation of circulating IgG antibodies against Porphyromonas gingivalis or its gingipains as serological markers of periodontitis and carriage of the bacterium | Massarenti, L. and Nielsen, C. H. and Danielsen, A. K. and Jensen, P. O. and Enevold, C. and Damgaard, C. | 3 |
| Pentraxin 3 and its role in periodontitis | Mathew V. and Sankari M. and Jangid K. | 3 |
| Inflammatory cytokine production and specific antibody responses against possible causative bacteria in patients with multilesional periapical periodontitis | Matsushita K. and Tajima T. and Tomita K. and Abeyama K. and Maruyama I. and Takada H. and Nagaoka S. | 3 |
| Inflammatory cytokine production and specific antibody responses to lipopolysaccharide from endodontopathic black-pigmented bacteria in patients with multilesional periapical periodontitis | Matsushita K. and Tajima T. and Tomita K. and Takada H. and Nagaoka S. and Torii M. | 3 |
| Interleukin-4 polymorphisms in early onset periodontitis | Michel J. and Gonzales J.R. and Wunderlich D. and Diete A. and Herrmann J.M. and Meyle J. | 3 |
| Comparative evaluation of the levels of nod-like receptor family pyrin domain-containing protein (NLRP) 3 in saliva of subjects with chronic periodontitis and healthy controls | Mitra D. and Chavan R. and Prithyani S. and Kandawalla S. and Shah R. and Rodrigues S. | 3 |
| Myeloperoxidase isoform activities released by human neutrophils in response to dental and periodontal bacteria | Miyasaki K.T. and Nemirovskiy E. | 3 |
| Presence of SNP of interleukin17F in patients with periodontitis in a Bulgarian population | Mlachkova A. and Pashova-Tasseva Z. and Popova H. and Kicheva M. | 3 |
| Actinobacillus actinomycetemcomitans in Chinese adults. Serotype distribution and analysis of the leukotoxin gene promoter locus | Mombelli A. and Gmur R. and Lang N.P. and Corbert E. and Frey J. | 3 |
| Molecular epidemiology of oral treponemes associated with periodontal disease | Moter A. and Hoenig C. and Choi B.-K. and Riep B. and Gobel U.B. | 3 |
| Identification of Potential Oral Microbial Biomarkers for the Diagnosis of Periodontitis | Na, Hee Sam and Kim, Si Yeong and Han, Hyejung and Kim, Hyun-Joo and Lee, Ju-Youn and Lee, Jae-Hyung and Chung, Jin | 3 |
| Effect of scaling and root planing on level of immunoglobulin E and immunoglobulin G4 in children with gingivitis and house-dust mite allergy: A pilot randomised controlled trial | Nelwan S.C. and Nugraha R.A. and Endaryanto A. and Dewi F. and Nuraini P. and Tedjosasongko U. and Utomo D.H. AO - Nelwan, Sindy Cornelia; ORCID: https://orcid.org/0000-0001-7829-6524 AO ... | 3 |
| Association between interleukin-6 -174 polymorphism and Aggregatibacter actinomycetemcomitans in chronic periodontitis | Nibali L. and Donos N. and Farrell S. and Ready D. and Pratten J. and Tu Y.K. and D'Aiuto F. | 3 |
| IL6 -174 genotype associated with Aggregatibacter actinomycetemcomitans in Indians. | Nibali, L. and Madden, I. and Chillida, F. F. and Heitz-Mayfield, L. J. A. and Brett, P. M. and Donos, N. | 3 |
| Interleukin-6 polymorphisms are associated with pathogenic bacteria in subjects with periodontitis. | Nibali, Luigi and Tonetti, Maurizio S and Ready, Derren and Parkar, Mohamed and Brett, Peter M and Donos, Nikos and D'Aiuto, Francesco | 3 |
| MIP-1alpha and MCP-1 as salivary biomarkers in periodontal disease | Nisha K.J. and Suresh A. and Anilkumar A. and Padmanabhan S. | 3 |
| The bacterial association with oral cavity and intra-abdominal abscess after gastrectomy | Nishikawa, M. and Honda, M. and Kimura, R. and Kobayashi, A. and Yamaguchi, Y. and Hori, S. and Kobayashi, H. and Waragai, M. and Kawamura, H. and Nakayama, Y. and Todate, Y. and Takano, Y. and Yamaguchi, H. and Hamada, K. and Iketani, S. and Seto, I. and Izumi, Y. and Seto, K. | 3 |
| alpha-Amylase is a potential growth inhibitor of Porphyromonas gingivalis, a periodontal pathogenic bacterium | Ochiai A. and Harada K. and Hashimoto K. and Shibata K. and Ishiyama Y. and Mitsui T. and Tanaka T. and Taniguchi M. | 3 |
| The microbial morphotypes associated with periodontal health and adult periodontitis: composition and distribution. | Offenbacher, S and Odle, B and van Dyke, T | 3 |
| Level of neopterin, a marker of immune cell activation in gingival crevicular fluid, saliva, and urine in patients with aggressive periodontitis | Ozmeric N. and Baydar T. and Bodur A. and Engin A.B. and Uraz A. and Eren K. and Sahin G. | 3 |
| Host response tests for diagnosing periodontal diseases | Page R.C. | 3 |
| Gingival crevicular fluid levels of interferon- gamma , but not interleukin-4 or -33 or thymic stromal lymphopoietin, are increased in inflamed sites in patients with periodontal disease. | Papathanasiou, E. and Flavia, T. and Griffin, T. and Arguello, E. and Finkelman, M. and Hanley, J. and Theoharides, T. C. | 3 |
| Sulcular sulfide monitoring: an indicator of early dental plaque-induced gingival disease. | Pavolotskaya, Aleksandra and McCombs, Gayle and Darby, Michele and Marinak, Kenneth and Dayanand, Naik N | 3 |
| C-reactive protein as a systemic marker of inflammation in periodontitis. | Pejcic, A. and Kesic, L. J. and Milasin, J. | 3 |
| The association between detectable plasmatic human immunodeficiency virus (HIV) viral load and different subgingival microorganisms in Brazilian adults with HIV: a multilevel analysis | Pereira V.T. and Pavan P. and Souza R.C. and Souto R. and Vettore M.V. and Torres S.R. and Colombo A.P. and de Uzeda M. and Sansone C. and Goncalves L.S. | 3 |
| Serum antibody titers to Bacteroides forsythus in elderly subjects with gingivitis or periodontitis | Persson G.R. and Schlegel-Bregenzer B. and Chung W.O. and Houston L. and Oswald T. and Roberts M.C. | 3 |
| The Effect of Acute High-Altitude Exposure on Oral Pathogenic Bacteria and Salivary Oxi-Inflammatory Markers | Pignatelli, P. and Mrakic-Sposta, S. and Bondi, D. and D'Antonio, D. L. and Piattelli, A. and Santangelo, C. and Verratti, V. and Curia, M. C. | 3 |
| Hyaluronan (hyaluronic acid) and its regulation in human saliva by hyaluronidase and its inhibitors. | Pogrel, Michael Anthony and Low, Marie Anne and Stern, Robert | 3 |
| Determination of dysbiosis by the method of multiplex real-time polymerase chain reaction in chronic catarrhal gingivitis in children | Polishchuk, T. V. and Sheshukova, O. V. and Mosiienko, A. S. and Trufanova, V. P. and Bauman, S. S. and Kazakova, K. S. | 3 |
| A randomized, single-blind, parallel-group clinical study to evaluate the effect of soluble beta-1,3/1,6-glucan on experimental gingivitis in man. | Preus, Hans R and Aass, Anne M and Hansen, Bjorn F and Moe, Brit and Gjermo, Per | 3 |
| Correlation of salivary immunoglobulin A against lipopolysaccharide of Porphyromonas gingivalis with clinical periodontal parameters. | Pudakalkatti, P. S. and Baheti, A. S. | 3 |
| Frequency of Fimbrial Gene Types I, Ib, and II in Clinical Strains of Porphyromonas gingivalis Characterized From Periodontitis Patients | R, Pradeep V. and Girija, A. S. Smiline and Priyadharsini, J. Vijayashree and Parameshwari, K. Kannika | 3 |
| Localization and density of <ovid:i>Porphyromonas gingivalis</ovid:i> and <ovid:i>Tannerella forsythia</ovid:i> in gingival and subgingival granulation tissues affected by chronic or aggressive periodontitis | Rajakaruna, G. A. and Negi, M. and Uchida, K. and Sekine, M. and Furukawa, A. and Ito, T. and Kobayashi, D. and Suzuki, Y. and Akashi, T. and Umeda, M. and Meinzer, W. and Izumi, Y. and Eishi, Y. | 3 |
| Oral diagnostic methods for the detection of periodontal disease | Ramenzoni L.L. and Lehner M.P. and Kaufmann M.E. and Wiedemeier D. and Attin T. and Schmidlin P.R. | 3 |
| Periodontal bacteria in human carotid atherothrombosis as a potential trigger for neutrophil activation | Range H. and Labreuche J. and Louedec L. and Rondeau P. and Planesse C. and Sebbag U. and Bourdon E. and Michel J.-B. and Bouchard P. and Meilhac O. | 3 |
| Interleukin-2 -330 and 166 gene polymorphisms in relation to aggressive or chronic periodontitis and the presence of periodontopathic bacteria | Reichert S. and MacHulla H.K.G. and Klapproth J. and Zimmermann U. and Reichert Y. and Glaser C. and Schaller H.-G. and Schulz S. | 3 |
| The interleukin-10 promoter haplotype ATA is a putative risk factor for aggressive periodontitis | Reichert S. and MacHulla H.K.G. and Klapproth J. and Zimmermann U. and Reichert Y. and Glaser C.H. and Schaller H.G. and Stein J. and Schulz S. | 3 |
| Are there common human leucocyte antigen associations in juvenile idiopathic arthritis and periodontitis? | Reichert, S. and Stein, J. and Fuchs, C. and John, V. and Schaller, H. G. and Machulla, H. K. G. | 3 |
| Individual composition of human leukocyte antigens and periodontopathogens in the background of periodontitis. | Reichert, Stefan and Altermann, Wolfgang and Stein, Jamal M and Schaller, Hans-Gunter and Machulla, Helmut K G and Schulz, Susanne | 3 |
| Interferon-gamma and interleukin-12 gene polymorphisms and their relation to aggressive and chronic periodontitis and key periodontal pathogens. | Reichert, Stefan and Machulla, Helmut K G and Klapproth, Jana and Zimmermann, Uta and Reichert, Yvonne and Glaser, Christiane and Schaller, Hans-Gunter and Schulz, Susanne | 3 |
| The genetic impact of the Q551R interleukin-4 receptor alpha polymorphism for aggressive or chronic periodontitis and the occurrence of periodontopathic bacteria. | Reichert, Stefan and Stein, Jamal M and Klapproth, Jana and Zimmermann, Uta and Reichert, Yvonne and Glaser, Christiane and Schaller, Hans-Gunter and Schulz, Susanne | 3 |
| Analysis of leukotoxin gene types of Actinobacillus actinomycetemcomitans in Brazilians with aggressive periodontitis. | Rosalem Junior, W. and Souza, R. C. de and Andrade, A. F. B. de and Colombo, A. P. V. | 3 |
| Estimation of serum antibody to subgingival species using checkerboard immunoblotting. | Sakellari, D and Socransky, S S and Dibart, S and Eftimiadi, C and Taubman, M A | 3 |
| Increased expression of HSP70, HSP90 and P53 in tissue of patients with periodontal and gingivitis deseases | Sanchez-Rodriguez, S. and Lopez-Martinez, L. and Hernandez-Rodriguez, A. and Ramirez-Santoyo, R. and Barbosa-Cisneros, O. and Aguilera-Galaviz, L. and Lopez-Luna, M. | 3 |
| Relationship between interleukin 1 (IL-1) genetic polymorphism and periimplantitis: systematic literature review and meta-analysis | Santostasi, N. and Gerardi, D. and Rinaldi, F. and Bernardi, S. and Bianchi, I. and Pinchi, V. and Piattelli, M. and Varvara, G. | 3 |
| Polymerase chain reaction for the detection of flaA-1 genes of oral spirochaetes in human advanced periodontal pockets. | Sato, T and Kuramitsu, H K | 3 |
| Antiphosphorylcholine antibody levels are elevated in humans with periodontal diseases | Schenkein H.A. and Gunsolley J.C. and Best A.M. and Harrison M.T. and Hahn C.-L. and Wu J. and Tew J.G. | 3 |
| The role of SNPs in TGF-b1 at codon 10 and 25 and the occurrence of severe periodontitis | Schulz S. and Altermann W. and Klapproth J. and Zimmermann U. and Glaser C. and Stein J.M. and Schaller H.G. and Reichert S. | 3 |
| The del/del genotype of the nuclear factor-kappaB -94ATTG polymorphism and its relation to aggressive periodontitis | Schulz S. and Hierse L. and Altermann W. and Klapproth J. and Zimmermann U. and Reichert Y. and Glaser C. and Kluttig A. and Stein J.M. and Schaller H.-G. and Reichert S. | 3 |
| Impact of genetic variants of CD14 and TLR4 on subgingival periodontopathogens | Schulz S. and Zissler N. and Altermann W. and Klapproth J. and Zimmermann U. and Glaser C. and Schaller H.-G. and Reichert S. | 3 |
| Single nucleotide polymorphisms in interleukin-1gene cluster and subgingival colonization with Aggregatibacter actinomycetemcomitans in patients with aggressive periodontitis. | Schulz, S. and Stein, J. M. and Altermann, W. and Klapproth, J. and Zimmermann, U. and Reichert, Y. and Glaser, C. and Schaller, H. G. and Reichert, S. | 3 |
| Lancefield group of <ovid:i>Streptococci</ovid:i> isolated from dental plaque biofilm | Shailja Singh, Shailja Singh and Khushbu Verma, Khushbu Verma | 3 |
| IL-17 and IL-11 GCF levels in aggressive and chronic periodontitis patients: relation to PCR bacterial detection. | Shaker, Olfat G and Ghallab, Noha A | 3 |
| Improvement of periodontal condition by probiotics with Lactobacillus salivarius WB21: a randomized, double-blind, placebo-controlled study. | Shimauchi, H. and Mayanagi, G. and Nakaya, S. and Minamibuchi, M. and Ito, Y. and Yamaki, K. and Hirata, H. | 3 |
| Antibody and T cell responses to <ovid:i>Fusobacterium nucleatum</ovid:i> and <ovid:i>Treponema denticola</ovid:i> in health and chronic periodontitis | Shin Jieun, Shin Jieun and Kho SangA, Kho SangA and Choi, Y. S. and Kim, Y. C. and Rhyu InChul, Rhyu InChul and Choi YoungNim, Choi YoungNim | 3 |
| Leukotoxicity of Aggregatibacter actinomycetemcomitans in generalized aggressive periodontitis in Brazilians and their family members | Silveira V.R.S. and Nogueira M.V.B. and Nogueira N.A.P. and Lima V. and Furlaneto F.A.C. and Rego R.O. | 3 |
| Immunoglobulin G response of periodontitis patients to Porphyromonas gingivalis capsular carbohydrate and lipopolysaccharide antigens | Sims T.J. and Schifferle R.E. and Ali R.W. and Skaug N. and Page R.C. | 3 |
| Oxidative Stress and IgG Antibody Modify Periodontitis-CRP Association | Singer R.E. and Moss K. and Kim S.J. and Beck J.D. and Offenbacher S. | 3 |
| Levels of matrix metalloproteinases-8 and -9 with simultaneous presence of periodontal pathogens in gingival crevicular fluid as well as matrix metalloproteinase-9 and cholesterol in blood | Soder B. and Airila Mansson S. and Soder P.-O. and Kari K. and Meurman J. | 3 |
| Extended-spectrum beta-lactamase-producing bacteria are not detected in supragingival plaque samples from human fecal carriers of ESBL-producing enterobacteriaceae | Soraas A. and Olsen I. and Sundsfjord A. and Handal T. and Bjorang O. and Jenum P.A. | 3 |
| Porphyromonas gingivalis in dental plaque and serum C-reactive protein levels in pregnancy. | Souccar, N. M. and Chakhtoura, M. and Ghafari, J. G. and Abdelnoor, A. M. | 3 |
| Increased periodontal inflammation in women with preterm premature rupture of membranes | Stadelmann P.F. and Eick S. and Salvi G.E. and Surbek D. and Mohr S. and Burgin W. and Ramseier C.A. and Sculean A. | 3 |
| Control of gingivitis and calculus by a dentifrice containing a zinc salt and triclosan. | Stephen, K W and Saxton, C A and Jones, C L and Ritchie, J A and Morrison, T | 3 |
| Identification of Periopathogenes from Dental Plaque in Periodontal Patients with PCR Technique and Their Association with Composite Interleukin-1 Genotype | Stojanovska A.A. and Todoroska S. and Popovska M. and Muratovska I. and Bedzeti L.Z. | 3 |
| Serotypes of Aggregatibacter actinomycetemcomitans in relation to periodontal status and assessment of leukotoxin in periodontal disease: a clinico-microbiological study. | Suprith, S. S. and Swati Setty, Swati Setty and Kishore Bhat, Kishore Bhat and Srinath Thakur, Srinath Thakur | 3 |
| Correlates of periodontal decline and biologic markers in older adults | Swoboda J.R. and Kiyak H.A. and Darveau R. and Persson G.R. | 3 |
| CE-LIF determination of salivary cadaverine and lysine concentration ratio as an indicator of lysine decarboxylase enzyme activity | Tabi T. and Lohinai Z. and Palfi M. and Levine M. and Szoko E. | 3 |
| Impact of non-surgical periodontal therapy on serum resistin and periodontal pathogen in periodontitis patients with obesity | Tahir, Khairunnisa Md and Ainul Haliza, Ab Malek and Vaithilingam, Rathna Devi and Saub, Roslan and Safii, Syarida Hasnur and Rahman, Mohammad Tariqur and Razak, Fathilah Abdul and Alabsi, Aied M. and Baharuddin, Nor Adinar | 3 |
| Serum IgG reactivity to subgingival bacteria in initial periodontitis, gingivitis and healthy subjects | Tanner A.C. and Kent Jr. R.L. and Maiden M.F. and Macuch P.J. and Taubman M.A. | 3 |
| Clinical and microbiological effects of subgingival irrigation with Streblus asper leaf extract in chronic periodontitis | Taweechaisupapong S. and Intaranongpai K. and Suwannarong W. and Pitiphat W. and Chatrchaiwiwatana S. and Wara-Aswapati N. | 3 |
| Relationships among gingival crevicular fluid biomarkers, clinical parameters of periodontal disease, and the subgingival microbiota. | Teles, Ricardo and Sakellari, Dimitra and Teles, Flavia and Konstantinidis, Antonis and Kent, Ralph and Socransky, Sigmund and Haffajee, Anne | 3 |
| The diagnostic potential of salivary protease activities in periodontal health and disease | Thomadaki K. and Bosch J.A. and Oppenheim F.G. and Helmerhorst E.J. | 3 |
| Streptococcus mitis and Prevotella melaninogenica Influence Gene Expression Changes in Oral Mucosal Lesions in Periodontitis Patients | Tomic, U. and Nikolic, N. and Carkic, J. and Mihailovic, D. and Jelovac, D. and Milasin, J. and Pucar, A. | 3 |
| Current status of proteomic technologies for discovering and identifying gingival crevicular fluid biomarkers for periodontal disease | Tsuchida S. and Satoh M. and Takiwaki M. and Nomura F. | 3 |
| Detection of periodontopathogenic bacteria in pregnant women by traditional anaerobic culture method and by a commercial molecular genetic method. | Urban, Edit and Terhes, Gabriella and Radnai, Marta and Gorzo, Istvan and Nagy, Elisabeth | 3 |
| Antigenically diverse reference strains and autologous strains of Actinobacillus actinomycetemcomitans are equally efficient antigens in enzyme-linked immunosorbent assay analysis. | Vilkuna-Rautiainen, Tiina and Pussinen, Pirkko J and Mattila, Kimmo and Vesanen, Marja and Ahman, Heidi and Dogan, Basak and Asikainen, Sirkka | 3 |
| Neutrophil gelatinase associated lipocalin a biomarker for bacterial-induced pharyngeal infection-A pilot study. | Walvik, Lena and Kirchmann, Malene and Jensen, Claus Antonio Juel and Kristiansen, Soren and Hansen, Lennart Friis and Howitz, Michael Frantz | 3 |
| Relationship between expression of human gingival beta-defensins and levels of periodontopathogens in subgingival plaque | Wang P. and Duan D. and Zhou X. and Li X. and Yang J. and Deng M. and Xu Y. | 3 |
| [A preliminary study on the effect of histatin 5 inhibiting <ovid:i>Porphyromonas gingivalis</ovid:i> and <ovid:i>Fusobacterium nucleatum</ovid:i> co-aggregation] | Wang, H. Y. and Liu, J. W. and Li, Q. and Tan, L. S. and Lin, L. and Pan, Y. P. | 3 |
| P. gingivalis in oral-prostate axis exacerbates benign prostatic hyperplasia via IL-6/IL-6R pathway | Wang, S. Y. and Cai, Y. and Hu, X. and Li, F. and Qian, X. H. and Xia, L. Y. and Gao, B. and Wu, L. and Xie, W. Z. and Gu, J. M. and Deng, T. and Zhu, C. and Jia, H. C. and Peng, W. Q. and Huang, J. and Fang, C. and Zeng, X. T. | 3 |
| CD11b mRNA expression in neutrophils isolated from peripheral blood and gingival crevicular fluid | Watanabe, K. and Blew, B. and Scherer, M. and Burke, J. and Koh, G. and Block, C. and Ramakrishnan, V. and Frommel, T. O. | 3 |
| Role of Calprotectin as a Biomarker in Periodontal Disease | Wei, L. and Liu, M. and Xiong, H. and Pagliari, C. | 3 |
| Specific antibody responses to subgingival plaque bacteria as aids to the diagnosis and prognosis of destructive periodontitis | Wilton J.M. and Johnson N.W. and Curtis M.A. and Gillett I.R. and Carman R.J. and Bampton J.L. and Griffiths G.S. and Sterne J.A. | 3 |
| The association between subgingival periodontal pathogens and systemic inflammation | Winning L. and Patterson C.C. and Cullen K.M. and Stevenson K.A. and Lundy F.T. and Kee F. and Linden G.J. | 3 |
| Treponema denticola associates with increased levels of MMP-8 and MMP-9 in gingival crevicular fluid. | Yakob, M. and Meurman, J. H. and Sorsa, T. and Soder, B. | 3 |
| Distribution of Archaea in Japanese patients with periodontitis and humoral immune response to the components | Yamabe K. and Maeda H. and Kokeguchi S. and Tanimoto I. and Sonoi N. and Asakawa S. and Takashiba S. | 3 |
| D-mannose alleviated alveolar bone loss in mice with experimental periodontitis via regulating the anti-inflammatory effect of amino acids. | Yang, Hua and Han, Nan-nan and Luo, Zhen-hua and Xu, Jun-ji and Guo, Li-jia and Liu, Yi | 3 |
| Streptococci and Actinomyces induce antibodies which cross react with epithelial antigens in periodontitis | Ye P. and Harty D.W.S. and Chapple C.C. and Nadkarni M.A. and Carlo A.A.D.E. and Hunter N. | 3 |
| Analysis of the activity to induce Toll-like Receptor (TLR)2- And TLR4-mediated stimulation of supragingival plaque | Yoshioka H. and Yoshimura A. and Kaneko T. and Golenbock D.T. and Hara Y. | 3 |
| Estimating the salivary levels of IL-35 in smokers with periodontitis: A cross sectional study | Yuvashri, P. and Renuka Devi, R. and Esther Nalini, H. and Arun Kumar Prasad, P. | 3 |
| Salivary thiols and enzyme markers of cell damage in periodontal disease | Zappacosta B. and Manni A. and Persichilli S. and Boari A. and Scribano D. and Minucci A. and Raffaelli L. and Giardina B. and De Sole P. | 3 |
| A Systems Biology Approach to Reveal Putative Host-Derived Biomarkers of Periodontitis by Network Topology Characterization of MMP-REDOX/NO and Apoptosis Integrated Pathways. | Zeidan-Chulia, Fares and Gursoy, Mervi and Neves de Oliveira, Ben-Hur and Ozdemir, Vural and Kononen, Eija and Gursoy, Ulvi K | 3 |
| Macrophage activating factor: A potential biomarker of periodontal health status | Zhang P. and Fan Y. and Li Q. and Chen J. and Zhou W. and Luo Y. and Zhang J. and Su L. and Xue X. and Zhou X. and Feng Y. | 3 |
| Total antioxidant capacity and total oxidant status in saliva of periodontitis patients in relation to bacterial load | Zhang T. and Andrukhov O. and Haririan H. and Muller-Kern M. and Liu S. and Liu Z. and Rausch-Fan X. | 3 |
| Relationship between vaginal and oral microbiome in patients of human papillomavirus (HPV) infection and cervical cancer | Zhang, W. and Yin, Y. and Jiang, Y. and Yang, Y. and Wang, W. and Wang, X. and Ge, Y. and Liu, B. and Yao, L. | 3 |
| Subgingival plaque microbiota in HIV positive patients | Aas J.A. and Barbuto S.M. and Alpagot T. and Olsen I. and Dewhirst F.E. and Paster B.J. | 4 |
| Does pregnancy have an impact on the subgingival microbiota?. | Adriaens, Laurence M and Alessandri, Regina and Sporri, Stefan and Lang, Niklaus P and Persson, G Rutger | 4 |
| Quantification of key periodontal pathogens in insulin-dependent type 2 diabetic and non-diabetic patients with generalized chronic periodontitis. | Aemaimanan, P. and Amimanan, P. and Taweechaisupapong, S. | 4 |
| Interrelationship between periodontitis and chronic obstructive pulmonary disease | Agarwal S.K. and Agarwal K. and Kumar N. | 4 |
| Biologically Defined or Biologically Informed Traits Are More Heritable Than Clinically Defined Ones: The Case of Oral and Dental Phenotypes | Agler C.S. and Moss K. and Philips K.H. and Marchesan J.T. and Simancas-Pallares M. and Beck J.D. and Divaris K. | 4 |
| Study of oral microbial flora in institutionalized mentally retarded pediatrics of SARI -2011 | Ahanjan M. and Akhavan A. and Abedian F. and Mirabi A.M. and Hagshenas M.R. | 4 |
| Simple and Sensitive Detection of Bacterial Hydrogen Sulfide Production Using a Paper-Based Colorimetric Assay | Ahn, Byung-Ki and Ahn, Yong-Jin and Lee, Young-Ju and Lee, Yeon-Hee and Lee, Gi-Ja | 4 |
| Microbiologic analysis of periodontal pockets and carotid atheromatous plaques in advanced chronic periodontitis patients. | Aimetti, Mario and Romano, Federica and Nessi, Franco | 4 |
| Is the presence of Helicobacter pylori in dental plaque of patients with chronic periodontitis a risk factor for gastric infection? | Al Asqah, M. and Al Hamoudi, N. and Anil, S. and Al Jebreen, A. and Al-Hamoudi, W. K. | 4 |
| The effect of the extract of the miswak (chewing sticks) used in Jordan and the Middle East on oral bacteria | Al lafi T. and Ababneh H. | 4 |
| Can Helicobacter pylori reside in the human oral cavity? | Al-Ahmad A. and Kurschner A. and Wittmer A. and Hellwig E. and Kist M. and Waidner B. | 4 |
| Is the presence of <ovid:i>Helicobacter pylori</ovid:i> in dental plaque of patients with chronic periodontitis a risk factor for gastric infection? | Al-Asqah, M. and Al-Hamoudi, N. and Anil, S. and Al-Jebreen, A. and Al-Hamoudi, W. | 4 |
| Genotypic and phenotypic detection of some virulence factors among Porphyromonas gingivalis related with period ontitis in Al-Najaf Al-Ashraf city, Iraq. | Al-Bdery, A. S. J. and Al-Yasseen, A. K. | 4 |
| Isolation and antibiotic susceptibility of anaerobic bacteria of periodontitis in diabetic and non-diabetic patients in Basrah Province, South of Iraq | Al-Farhan S.R. and Al-Mussawi A.A. and Al-Abdulla A.A. | 4 |
| Indocyanine-mediated antimicrobial photodynamic therapy promotes superior clinical effects in stage III and grade C chronic periodontitis among controlled and uncontrolled diabetes mellitus: A randomized controlled clinical trial | Al-Momani M.M. | 4 |
| The Relation between Periodontopathogenic Bacterial Levels and Resistin in the Saliva of Obese Type 2 Diabetic Patients | Al-Rawi N. and Al-Marzooq F. AO - Al-Rawi, Natheer; ORCID: https://orcid.org/0000-0002-7483-6594 AO - ... | 4 |
| Diagnosis and detection of VicK gene in Streptococcus mutans isolated from the saliva of patients with diabetic type 2 with tooth decay in the Iraqi population. | Al-Sudani, Susan F. Khadhem and Hamad, Laheeb R. and Ali, Fattma A. | 4 |
| Dental plaque microbial profiles of children from Khartoum, Sudan, with congenital heart defects | Ali H.M. and Berggreen E. and Nguyen D. and Ali R.W. and Van Dyke T.E. and Hasturk H. and Mustafa M. | 4 |
| The comparison between dental plaque score before and after gargling with tongra original honey 5% solution (Study of Student in Dentistry of Syiah Kuala University) | Alibasyah Z.M. and Sunnati and Saputri D. and Alviana V. | 4 |
| Modulation of the Oral Microbiome to Improve Oral Health and Reduce BSI from Oral Flora in Pediatric Hematopoietic Stem Cell Transplant Recipients: A Randomized Controlled Trial | Alonso P.B. and Andersen H. and Haslam D. and Nelson A.S. and El-Bietar J. and Pate A.R. and Golkari S. and Teusink-Cross A. and Flesch L. and Bedel A. and Hickey V. and Kramer K. and Davies S.M. and Thikkurissy S. and Dandoy C.E. | 4 |
| Risk factors for periodontitis in HIV patients | Alpagot T. and Duzgunes N. and Wolff L.F. and Lee A. | 4 |
| Risk factors for periodontitis in HIV<ovid:sup>+</ovid:sup> patients | Alpagot, T. and Duzgunes, N. and Wolff, L. F. and Lee, A. | 4 |
| Identification of microorganisms in biofluids of individuals with periodontitis and chronic kidney disease using matrix-assisted laser desorption/ionization time-of-flight mass spectrometry | Alves L.A. and Souza R.C. and da Silva T.M. and Watanabe A. and Dias M. and Mendes M.A. and Ciamponi A.L. | 4 |
| Oral care in pediatric intensive care unit | Alwahsh, S. and Heard, C. and Twiss, J. and Alawadhi, Y. and Joshi, P. and Sciandra, D. and Faden, H. | 4 |
| Relationship of periodontopathic bacteria with early-onset periodontitis in Down's syndrome | Amano A. and Kishima T. and Akiyama S. and Nakagawa I. and Hamada S. and Morisaki I. | 4 |
| Relationship between halitosis and periodontal disease - associated oral bacteria in tongue coatings | Amou T. and Hinode D. and Yoshioka M. and Grenier D. | 4 |
| Microbial profile on metallic and ceramic bracket materials. | Anhoury, Patrick and Nathanson, Dan and Hughes, Christopher V and Socransky, Sigmund and Feres, Magda and Chou, Laisheng Lee | 4 |
| Oral and Gut Microbial Diversity and Immune Regulation in Patients with HIV on Antiretroviral Therapy | Annavajhala, Medini K. and Khan, Sabrina D. and Sullivan, Sean B. and Shah, Jayesh and Pass, Lauren and Kister, Karolina and Kunen, Heather and Chiang, Victor and Monnot, Gwennaelle C. and Ricupero, Christopher L. and Mazur, Rebecca A. and Gordon, Peter and de Jong, Annemieke and Wadhwa, Sunil and Yin, Michael T. and Demmer, Ryan T. and Uhlemann, Anne-Catrin | 4 |
| Association of oral Helicobacter pylori with gastric complications | Ansari S.A. and Iqbal M.U.N. and Khan T.A. and Kazmi S.U. | 4 |
| Specific periodontopathic bacterial infection affects hypertension in male cardiovascular disease patients | Aoyama N. and Suzuki J.-I. and Kumagai H. and Ikeda Y. and Akazawa H. and Komuro I. and Minabe M. and Izumi Y. and Isobe M. AO - Aoyama, Norio; ORCID: https://orcid.org/0000-0002-8498-4494 | 4 |
| Increased Oral Porphyromonas gingivalis Prevalence in Cardiovascular Patients with Uncontrolled Diabetes Mellitus. | Aoyama, Norio and Suzuki, Jun-Ichi and Kobayashi, Naho and Hanatani, Tomoya and Ashigaki, Norihiko and Yoshida, Asuka and Shiheido, Yuka and Sato, Hiroki and Izumi, Yuichi and Isobe, Mitsuaki | 4 |
| Flow cytometry as a new method to quantify the cellular content of human saliva and its relation to gingivitis. | Aps, Johan K M and Van den Maagdenberg, Karijn and Delanghe, Joris R and Martens, Luc C | 4 |
| Detection of putative periodontopathic bacteria in type 1 diabetic and healthy children: a comparative study | Arangannal P. and Krishnan P. and Nichani M.H. and Krishnan M. and Chamarthi V. | 4 |
| Relationship between gingivitis and autism in children: a matched case-control study. | Archana Singh Sikarwar, Archana Singh Sikarwar and Abhishek Parolia, Abhishek Parolia and Fransazellea Anak, R. R. and Ankur Barua, Ankur Barua | 4 |
| Association between periodontal pathogens and coronary artery disease: A case-control study | Ardakani, M. T. and Sobouti, F. and Ghavidel, A. A. and Dehaki, M. G. and Shariati, M. and Kazemi, B. | 4 |
| Oral cleaning habits and the copy number of periodontal bacteria in pregnant women and its correlation with birth outcomes: an epidemiological study in Mibilizi, Rwanda | Arima, H. and Calliope, A. S. and Fukuda, H. and Nzaramba, T. and Mukakarake, M. G. and Wada, T. and Yorifuji, T. and Mutesa, L. and Yamamoto, T. | 4 |
| Inhibition activity of water hyacinth leaf extract (Eichornia crassipes) to the growth of subgingival plaque bacteria colony | Arismawati A. and Ulfah N. and Bargowo L. | 4 |
| Elevated subgingival levels of periodontal pathogens in rheumatoid arthritis patients, particularly leptotrichia species in new-onset disease | Arvikar S. and Hasturk H. and Nguyen D. and Strle K. and Bolster M.B. and Collier D. and Steere A.C. and Kantarci A. | 4 |
| Detection and quantification of oral treponemes in subgingival plaque by real-time PCR. | Asai, Y. and Jinno, T. and Igarashi, H. and Ohyama, Y. and Ogawa, T. | 4 |
| Chronic liver disease impairs bacterial clearance in a human model of induced bacteremia | Ashare A. and Stanford C. and Hancock P. and Stark D. and Lilli K. and Birrer E. and Nymon A. and Doerschug K.C. and Hunninghake G.W. | 4 |
| Lactic acid bacteria in the oral cavity - assessment of the probiotic potential | Atanasov N.N. and Evstatieva Y.Y. and Nikolova D.P. | 4 |
| The relationship between the presence of periodontopathogenic bacteria in saliva and halitosis | Awano S. and Gohara K. and Kurihara E. and Ansai T. and Takehara T. | 4 |
| Filifactor alocis and Dialister pneumosintes in a Mexican population affected by periodontitis and rheumatoid arthritis: An exploratory study | Ayala Herrera J.L. and Apreza Patron L. and Martinez Martinez R.E. and Dominguez Perez R.A. and Abud Mendoza C. and Hernandez Castro B. AO - Ayala Herrera, Jose Luis; ORCID: https://orcid.org/0000-0001-7732-7... | 4 |
| Assessing the levels of immunoglobulins in the saliva of diabetic individuals with periodontitis using checkerboard immunodetection. | Bachrach, G. and Muster, Z. and Raz, I. and Chaushu, G. and Stabholz, A. and Nussbaum, G. and Gutner, M. and Chaushu, S. | 4 |
| Impact of xylitol on oral microbiome and blood stream infections in HSCT recipients | Badia P. and Andersen H. and Haslam D. and Nelson A.S. and El-Bietar J. and Pate A.R. and Golkari S. and Teusink-Cross A. and Flesch L. and Bedel A. and Hickey V. and Kramer K. and Davies S.M. and Thikkurissy S. and Dandoy C.E. | 4 |
| Dysregulation of miR-146a by periodontal pathogens: A risk for acute coronary syndrome | Bagavad Gita J. and George A.V. and Pavithra N. and Chandrasekaran S.C. and Latchumanadhas K. and Gnanamani A. | 4 |
| Antibodies to periodontogenic bacteria are associated with higher disease activity in lupus patients | Bagavant H. and Dunkleberger M.L. and Wolska N. and Sroka M. and Rasmussen A. and Adrianto I. and Montgomery C. and Sivils K. and Guthridge J.M. and James J.A. and Merrill J.T. and Deshmukh U.S. | 4 |
| Salivary levels of tumor necrosis factor-alpha in periodontitis. | Balwant Rai, Balwant Rai | 4 |
| Association between Periodontal Disease and Oral Benign, Potentially Malignant, Malignant, and Chronic Immune-Mediated Disorders: A Clinical Study | Barbarisi, Antonio and Cremonini, Francesca and Lauritano, Dorina and Visconti, Valeria and Caccianiga, Gianluigi and Ceraulo, Saverio | 4 |
| Lipid peroxidation is associated with the severity of periodontal disease and local inflammatory markers in patients with type 2 diabetes | Bastos A.S. and Graves D.T. and Loureiro A.P.D.M. and Junior C.R. and Abdalla D.S.P. and Faulin T.D.E.S. and Camara N.O. and Andriankaja O.M. and Orrico S.R.P. | 4 |
| Classifying dementia progression using microbial profiling of saliva | Bathini P. and Foucras S. and Dupanloup I. and Imeri H. and Perna A. and Berruex J.-L. and Doucey M.-A. and Annoni J.-M. and Auber Alberi L. | 4 |
| Periodontal disease in individuals with a genetic risk of developing arthritis and early rheumatoid arthritis: a cross-sectional study. | Bello-Gualtero, J. M. and Lafaurie, G. I. and Hoyos, L. X. and Castillo, D. M. and De-Avila, J. and Munevar, J. C. and Unriza, S. and Londono, J. and Valle-Onate, R. and Romero-Sanchez, C. | 4 |
| Comparative analysis of bacterial profiles in unstimulated and stimulated saliva samples | Belstrom D. and Holmstrup P. and Bardow A. and Kokaras A. and Fiehn N.-E. and Paster B.J. | 4 |
| Metaproteomics of saliva identifies human protein markers specific for individuals with periodontitis and dental caries compared to orally healthy controls | Belstrom D. and Jersie-Christensen R.R. and Lyon D. and Damgaard C. and Jensen L.J. and Holmstrup P. and Olsen J.V. | 4 |
| Altered bacterial profiles in saliva from adults with caries lesions: a case-cohort study. | Belstrom, D and Fiehn, N-E and Nielsen, C H and Holmstrup, P and Kirkby, N and Klepac-Ceraj, V and Paster, B J and Twetman, S | 4 |
| Metagenomic and metatranscriptomic analysis of saliva reveals disease-associated microbiota in patients with periodontitis and dental caries. | Belstrom, Daniel and Constancias, Florentin and Liu, Yang and Yang, Liang and Drautz-Moses, Daniela I and Schuster, Stephan C and Kohli, Gurjeet Singh and Jakobsen, Tim Holm and Holmstrup, Palle and Givskov, Michael | 4 |
| Periodontal Disease, Inflammation and Atherosclerosis Progression in Patients with Acute Coronary Syndromes - The ATHERODENT Study | Benedek T. and Rodean I. and Ratiu M. and Rat N. and Eremie L.Y. and Birie C. and Lazr L. and Pcurar M. and Benedek I. | 4 |
| Effect of a stannous fluoride dentifrice on the sulcular microbiota: a prospective cohort study in subjects with various levels of periodontal inflammation | Benjasupattananan S. and Lai C.S. and Persson G.R. and Pjetursson B.E. and Lang N.P. | 4 |
| Detection of Helicobacter pylori DNA in the oral cavity and gastroduodenal system of a Venezuelan population. | Berroteran, A. and Perrone, M. and Correnti, M. and Cavazza, M. E. and Tombazzi, C. and Goncalvez, R. and Lecuna, V. | 4 |
| Periodontal disease and influence of periodontal treatment on disease activity in patients with rheumatoid arthritis and spondyloarthritis. | Bialowas, Katarzyna and Radwan-Oczko, Malgorzata and Dus-Ilnicka, Irena and Korman, Lucyna and Swierkot, Jerzy | 4 |
| The relationship between Porphyromonas gingivalis infection and local and systemic factors in children | Bimstein E. and Sapir S. and Houri-Haddad Y. and Dibart S. and Van Dyke T.E. and Shapira L. | 4 |
| Growth and development considerations in the diagnosis of gingivitis and periodontitis in children. | Bimstein, E and Matsson, L | 4 |
| The influence of abutment surface roughness on plaque accumulation and peri-implant mucositis | Bollen, C. M. and Papaioanno, W. and Van Eldere, J. and Schepers, E. and Quirynen, M. and van Steenberghe, D. | 4 |
| Smoking and subgingival microflora in periodontal disease | Bostrom L. and Bergstrom J. and Dahlen G. and Linder L.E. | 4 |
| Frequency of detection of periodontopathic and superinfecting bacteria in HIV-positive patients with periodontitis | Botero J.E. and Arce R.M. and Escudero M. and Betancourth M. and Jaramillo A. and Contreras A. | 4 |
| The effect of antimicrobial photodynamic therapy on periodontal disease and glycemic control in patients with type 2 diabetes mellitus | Brinar S. and Skvarca A. and Gaspirc B. and Schara R. | 4 |
| Evaluation of the relationship between smoking during pregnancy and subgingival microbiota. | Buduneli, Nurcan and Baylas, Haluk and Buduneli, Eralp and Turkoglu, Oya and Dahlen, Gunnar | 4 |
| Microbial composition of atherosclerotic plaques. | Calandrini, C A and Ribeiro, A C and Gonnelli, A C and Ota-Tsuzuki, C and Rangel, L P and Saba-Chujfi, E and Mayer, M P A | 4 |
| Subgingival microbiota in health compared to periodontitis and the influence of smoking. | Camelo-Castillo, A. J. and Mira, A. and Pico, A. and Nibali, L. and Henderson, B. and Donos, N. and Tomas, I. | 4 |
| Detection of Mogibacterium timidum in subgingival biofilm of aggressive and non-diabetic and diabetic chronic periodontitis patients. | Casarin, R. C. V. and Saito, D. and Santos, V. R. and Pimentel, S. P. and Duarte, P. M. and Casati, M. Z. and Goncalves, R. B. | 4 |
| Occurrence of periodontal pathogens in patients treated with fixed orthodontic appliances | Cernochova P. and Augustin P. and Fassmann A. and Izakovicova-Holla L. | 4 |
| Periodontopathic bacteria and herpesviruses in chronic periodontitis | Chalabi M. and Rezaie F. and Moghim S. and Mogharehabed A. and Rezaei M. and Mehraban B. | 4 |
| Diode Laser - A Novel Therapeutic Approach in the Treatment of Chronic Periodontitis in Type 2 Diabetes Mellitus Patients: A Prospective Randomized Controlled Clinical Trial. | Chandra, Sourav and Shashikumar, Pratibha | 4 |
| Adiponectin could be a mediator of the presence of p gingivalis in patients with early rheumatoid arthritis | Chaparro J.A. and Bello-Gualtero J.M. and Valle-Onate R. and Bautista-Molano W. and Chila L. and Castillo D.M. and Lafaurie G. and ChalemCh P. and Romero-Sanchez C. | 4 |
| Can metagenomics unravel the impact of oral bacteriome in human diseases? | Chattopadhyay I. and Lu W. and Manikam R. and Malarvili M.B. and Ambati R.R. and Gundamaraju R. | 4 |
| Occurrence of Aggregatibacter actinomycetemcomitans serotypes in subgingival plaque from United States subjects | Chen C. and Wang T. and Chen W. | 4 |
| Quantifying periodontitis-associated oral dysbiosis in tongue and saliva microbiomes-An integrated data analysis | Chew, R. J. J. and Tan, K. S. and Chen, T. and Al-Hebshi, N. N. and Goh, C. E. | 4 |
| Healing response to non-surgical periodontal therapy in patients with diabetes mellitus: clinical, microbiological, and immunologic results | Christgau M. and Palitzsch K.D. and Schmalz G. and Kreiner U. and Frenzel S. | 4 |
| Digital oral health biomarkers for early detection of cognitive decline | Chung P.-C. and Chan T.-C. | 4 |
| Maternal obesity, periodontitis and preterm delivery | Clivio V. and Mazzocco M. and Lissoni A. and Castellarin P. and Grossi E. and Calabrese S. and Mando C. and Cardellicchio M. and Novielli C. and Anelli G. and Cetin I. and Abate S. | 4 |
| Salivary matrix metalloproteinase (MMP-8) levels and gelatinase (MMP-9) activities in patients with type 2 diabetes mellitus | Collin H.L. and Sorsa T. and Meurman J.H. and Niskanen L. and Salo T. and Ronka H. and Konttinen Y.T. and Koivisto A.M. and Uusitupa M. | 4 |
| Parvimonas micra can translocate from the subgingival sulcus of the human oral cavity to colorectal adenocarcinoma | Conde-Perez, K. and Buetas, E. and Aja-Macaya, P. and Martin-De Arribas, E. and Iglesias-Corras, I. and Trigo-Tasende, N. and Nasser-Ali, M. and Estevez, L. S. and Rumbo-Feal, S. and Otero-Alen, B. and Noguera, J. F. and Concha, A. and Pardinas-Lopez, S. and Carda-Dieguez, M. and Gomez-Randulfe, I. and Martinez-Lago, N. and Ladra, S. and Aparicio, L. A. and Bou, G. and Mira, A. and Vallejo, J. A. and Poza, M. | 4 |
| The multispecies microbial cluster of Fusobacterium, Parvimonas, Bacteroides and Faecalibacterium as a precision biomarker for colorectal cancer diagnosis | Conde-Perez, Kelly and Aja-Macaya, Pablo and Buetas, Elena and Trigo-Tasende, Noelia and Nasser-Ali, Mohammed and Rumbo-Feal, Soraya and Nion, Paula and Arribas, Elsa Martin-De and Estevez, Lara S. and Otero-Alen, Begona and Noguera, Jose F. and Concha, Angel and Pardinas-Lopez, Simon and Carda-Dieguez, Miguel and Gomez-Randulfe, Igor and Martinez-Lago, Nieves and Ladra, Susana and Aparicio, Luis M. A. and Bou, German and Mira, Alex and Vallejo, Juan A. and Poza, Margarita | 4 |
| Flow cytometry-based analysis by Sysmex-UF1000i is an alternative method in the assessment of periodontal inflammation | Coopman R. and Speeckaert M.M. and Aps J.K. and Delanghe J.R. | 4 |
| Clinical status and detection of periodontopathogens and Streptococcus mutans in children with high levels of supragingival biofilm | Cortelli S.C. and Cortelli J.R. and Aquino D.R. and Holzhausen M. and Franco G.C.N. and Costa F.d.O. and Fine D. | 4 |
| Salivary and serum adma levels as biomarkers of endothelial dysfunction in patients with periodontal and cardiovascular disease | Curro M. and Ferlazzo N. and Isola G. and Rizzo V. and Caccamo D. and Ientile R. | 4 |
| Helicobacter pylori in the oral cavity and its implications in gastric infection, periodontal health, immunology and dyspepsia | Czesnikiewicz-Guzik M. and Bielanski W. and Guzik T.J. and Loster B. and Konturek S.J. | 4 |
| Role of suspected periodontopathogens in microbiological monitoring of periodontitis | Dahlen G. | 4 |
| Early detection of oral bacteria causing gum infections and dental caries in children | Darabi, M. and Bakhtiari, R. and Jafari, A. and Mehran, M. and Eshraghi, S. S. and Barati, A. | 4 |
| Salivary microbiota levels in relation to periodontal status, experience of caries and miswak use in Sudanese adults | Darout I.A. and Albandar J.M. and Skaug N. and Ali R.W. | 4 |
| Periodontal pathogens and gestational diabetes mellitus. | Dasanayake, A. P. and Chhun, N. and Tanner, A. C. R. and Craig, R. G. and Lee, M. J. and Moore, A. F. and Norman, R. G. | 4 |
| The subgingival plaque microbiome, systemic antibodies against bacteria and citrullinated proteins following periodontal therapy | Davison E. and Johnston W. and Piela K. and Rosier B.T. and Paterson M. and Mira A. and Culshaw S. | 4 |
| Prevalence of periodontitis is high in rheumatoid arthritis patients and correlated to disease activity | De Smit M.J. and Westra J. and Vissink A. and Der Meer B.D.-V. and Roelofs P.A. and Brouwer E. and Van Winkelhoff A.J. | 4 |
| Periodontitis in established rheumatoid arthritis patients: a cross-sectional clinical, microbiological and serological study. | de Smit, Menke and Westra, Johanna and Vissink, Arjan and Doornbos-van der Meer, Berber and Brouwer, Elisabeth and van Winkelhoff, Arie Jan | 4 |
| Disorders of the oral cavity. | DeBowes, L. J. and Hall, J. E. and Simpson, J. W. and Williams, D. A. | 4 |
| Features of severe periodontal disease in a teenager with Chediak-Higashi syndrome | Delcourt-Debruyne E.M. and Boutigny H.R. and Hildebrand H.F. | 4 |
| Pathogens in the inflammatory process of periodontal disease and juvenile idiopathic arthritis | Delnay N. and McNinch N. and Toth M. | 4 |
| Inadequate salivary flow and poor oral mucosal status in intubated intensive care unit patients. | Dennesen, Paul and van der Ven, Andre and Vlasveld, Mariel and Lokker, Linka and Ramsay, Graham and Kessels, Alphons and van den Keijbus, Petra and van Nieuw Amerongen, Arie and Veerman, Enno | 4 |
| Periodontal microbiota and carotid intima-media thickness: The Oral Infections and Vascular Disease Epidemiology Study (INVEST) | Desvarieux M. and Demmer R.T. and Rundek T. and Boden-Albala B. and Jacobs Jr. D.R. and Sacco R.L. and Papapanou P.N. | 4 |
| Changes in clinical and microbiological periodontal profiles relate to progression of carotid intima-media thickness: the Oral Infections and Vascular Disease Epidemiology study. | Desvarieux, Moise and Demmer, Ryan T and Jacobs, David R and Papapanou, Panos N and Sacco, Ralph L and Rundek, Tatjana | 4 |
| Haemophilus parasuis (Glaesserella parasuis) as a Potential Driver of Molecular Mimicry and Inflammation in Rheumatoid Arthritis | Di Sante G. and Gremese E. and Tolusso B. and Cattani P. and Di Mario C. and Marchetti S. and Alivernini S. and Tredicine M. and Petricca L. and Palucci I. and Camponeschi C. and Aragon V. and Gambotto A. and Ria F. and Ferraccioli G. AO - Di Sante, Gabriele; ORCID: https://orcid.org/0000-0001-6608-3388 AO ... | 4 |
| Prevalence of gingival candidiasis by cytopatholgy | Dias E.P. and Miranda A.M.O. and De Miranda Ferrari T. and Gregorio A.M. and Vielh P. and Solomon D. and Cochand-Priollet B. and Schmitt F. | 4 |
| Investigation of six selected bacterial species in endo-periodontal lesions | Didilescu A.C. and Rusu D. and Anghel A. and Nica L. and Iliescu A. and Greabu M. and Bancescu G. and Stratul S.I. | 4 |
| Decision tree approach to the impact of parents' oral health on dental caries experience in children: A cross-sectional study | Dima S. and Wang K.-J. and Chen K.-H. and Huang Y.-K. and Chang W.-J. and Lee S.-Y. and Teng N.-C. | 4 |
| Identification and characterization of genetic cluster groups of Actinobacillus actinomycetemcomitans isolated from the human oral cavity | DiRienzo J.M. and McKay T.L. | 4 |
| Molecular identification of Dialister pneumosintes in subgingival plaque of humans | Doan N. and Contreras A. and Flynn J. and Slots J. and Chen C. | 4 |
| Characteristics of periodontal microflora in acute myocardial infarction | Dogan B. and Buduneli E. and Emingil G. and Atilla G. and Akilli A. and Antinheimo J. and Lakio L. and Asikainen S. | 4 |
| Periodontitis, a marker of risk in pregnancy for preterm birth. | Dortbudak, Orhun and Eberhardt, Rita and Ulm, Martin and Persson, G Rutger | 4 |
| Preventing Aspiration in the Nursing Home: The Role of Biofilm and Data from the ICU | Drinka P. | 4 |
| Patients with type 2 diabetes and severe periodontitis harbor a less pathogenic subgingival biofilm than normoglycemic individuals with severe periodontitis | Duarte P.M. and Felix E. and Santos V.R. and Figueiredo L.C. and da Silva H.D.P. and Mendes J.A.V. and Feres M. and Miranda T.S. AO - Duarte, Poliana M.; ORCID: https://orcid.org/0000-0001-8872-5943 AO -... | 4 |
| Microbiological, lipid and immunological profiles in children with gingivitis and type 1 diabetes mellitus | Duque C. and Joao M.F. and Camargo G.A. and Teixeira G.S. and Machado T.S. and Azevedo R.S. and Mariano F.S. and Colombo N.H. and Vizoto N.L. and Mattos-Graner R.O. | 4 |
| Experimental gingivitis induces systemic inflammatory markers in young healthy individuals: a single-subject interventional study. | Eberhard, J. and Grote, K. and Luchtefeld, M. and Heuer, W. and Schuett, H. and Divchev, D. and Scherer, R. and Schmitz-Streit, R. and Langfeldt, D. and Stumpp, N. and Staufenbiel, I. and Schieffer, B. and Stiesch, M. | 4 |
| Salivary microbiome and biomarker characteristics of diabetics with periodontitis | Ebersole, J. L. and Kirakodu, S. S. and Zhang, X. and Dawson, D. and Miller, C. S. | 4 |
| A comparative clinical, microbiological and glycemic analysis of photodynamic therapy and Lactobacillus reuteri in the treatment of chronic periodontitis in type-2 diabetes mellitus patients | Elsadek M.F. and Ahmed B.M. and Alkhawtani D.M. and Zia Siddiqui A. | 4 |
| Periodontopathogen profile of healthy and oral lichen planus patients with gingivitis or periodontitis. | Ertugrul, Abdullah Seckin and Arslan, Ugur and Dursun, Recep and Hakki, Sema Sezgin | 4 |
| Oral microbiota identifies patients with early rheumatoid arthritis | Esberg A. and Johansson L. and Johansson I. and Rantapaa Dahlqvist S. | 4 |
| Heritability of Oral Microbiota and Immune Responses to Oral Bacteria. | Esberg, Anders and Haworth, Simon and Kuja-Halkola, Ralf and Magnusson, Patrik K E and Johansson, Ingegerd | 4 |
| Prevalence and antimicrobial susceptibility of Gram-negative bacilli in subgingival biofilm associated with periodontal diseases. | Espindola, Lais Christina Pontes and Picao, Renata Cristina and Mancano, Stella Maria Casas Novas and Martins do Souto, Renata and Colombo, Ana Paula Vieira | 4 |
| Possible link between periodontal disease and chronic prostatitis | Estemalik, J. and Bissada, N. and Joshi, N. and Demko, C. and Shankar, E. and Bodner, D. and Gupta, S. | 4 |
| Simultaneous Detection of Oral Pathogens in Subgingival Plaque and Prostatic Fluid of Men With Periodontal and Prostatic Diseases. | Estemalik, John and Demko, Catherine and Bissada, Nabil F and Joshi, Nishant and Bodner, Donald and Shankar, Eswar and Gupta, Sanjay | 4 |
| Oral microbiota in patients with atherosclerosis. | Fak, Frida and Tremaroli, Valentina and Bergstrom, Goran and Backhed, Fredrik | 4 |
| Are anti-citrullinated protein antibody levels associated with periodontal disease in rheumatoid arthritis? | Febles J.G. and Sanchez-Alonso F. and Rodriguez J.L.G. and Alonso M.S. and Diaz-Gonzalez F. and Lozano B.R. | 4 |
| Periodontal disease: an overview for physicians | Fenesy K.E. | 4 |
| Molecular analysis of oral bacteria in dental biofilm and atherosclerotic plaques of patients with vascular disease | Fernandes C.P. and Oliveira F.A.F. and Silva P.G.D.B. and Alves A.P.N.N. and Mota M.R.L. and Montenegro R.C. and Burbano R.M.R. and Seabra A.D. and Lobo Filho J.G. and Lima D.L.F. and Soares Filho A.W.E. and Sousa F.B. | 4 |
| Investigation and quantification of key periodontal pathogens in patients with type 2 diabetes | Field, C. A. and Gidley, M. D. and Preshaw, P. M. and Jakubovics, N. | 4 |
| The relationship of oral malodor in patients with or without periodontal disease | Figueiredo L.C. and Rosetti E.P. and Marcantonio Jr. E. and Marcantonio R.A.C. and Salvador S.L. | 4 |
| Quantification of periodontal pathogens in vascular, blood, and subgingival samples from patients with peripheral arterial disease or abdominal aortic aneurysms. | Figuero, Elena and Lindahl, Christeel and Marin, Maria Jose and Renvert, Stefan and Herrera, David and Ohlsson, Ola and Wetterling, Thomas and Sanz, Mariano | 4 |
| Detection of periodontal bacteria in atheromatous plaque by nested polymerase chain reaction. | Figuero, Elena and Sanchez-Beltran, Maria and Cuesta-Frechoso, Susana and Tejerina, Jose Maria and del Castro, Jose Antonio and Gutierrez, Jose Maria and Herrera, David and Sanz, Mariano | 4 |
| Xerostomy, dental caries and periodontal disease in HIV+ patients | Filho J.C.C. and Giovani E.M. | 4 |
| Xerostomy, dental caries and periodontal disease in HIV<ovid:sup>+</ovid:sup> patients | Filho, J. C. C. and Giovani, E. M. | 4 |
| Analysis of predisposing factors for rapid dental calculus formation | Fons-Badal C. and Fons-Font A. and Labaig-Rueda C. and Sola-Ruiz M.F. and Selva-Otaolaurruchi E. and Agustin-Panadero R. | 4 |
| Identification of caries risk factors in toddlers | Fontana M. and Jackson R. and Eckert G. and Swigonski N. and Chin J. and Ferreira Zandona A. and Ando M. and Stookey G.K. and Downs S. and Zero D.T. | 4 |
| Characterization of heat shock protein-specific T cells in atherosclerosis | Ford P. and Gemmell E. and Walker P. and West M. and Cullinan M. and Seymour G. | 4 |
| Exploring the connection between porphyromonas gingivalis and neurodegenerative diseases: A pilot quantitative study on the bacterium abundance in oral cavity and the amount of antibodies in serum | Franciotti R. and Pignatelli P. and Carrarini C. and Romei F.M. and Mastrippolito M. and Gentile A. and Mancinelli R. and Fulle S. and Piattelli A. and Onofrj M. and Curia M.C. | 4 |
| Oral microbiome and serological analyses on association of Alzheimer's disease and periodontitis | Fu K.-L. and Chiu M.-J. and Wara-aswapati N. and Yang C.-N. and Chang L.-C. and Guo Y.L. and Ni Y.-H. and Chen Y.-W. AO - Chen, Yi-Wen; ORCID: https://orcid.org/0000-0002-9008-8346 | 4 |
| Clonal Diversity and Stability of Subgingival Eikenella corrodens | Fujise O. and Chen W. and Rich S. and Chen C. | 4 |
| The presence of <ovid:i>Enterococcus faecalis</ovid:i> in saliva as a risk factor for endodontic infection | Gaeta, Carlo and Marruganti, Crystal and Ali, Islam A. A. and Fabbro, Andrea and Pinzauti, David and Santoro, Francesco and Neelakantan, Prasanna and Pozzi, Gianni and Grandini, Simone | 4 |
| Quantitative detection of periodontopathic bacteria in atherosclerotic plaques from coronary arteries | Gaetti-Jardim Jr. E. and Marcelino S.L. and Feitosa A.C.R. and Romito G.A. and Avila-Campos M.J. | 4 |
| Quantitative analysis of key periodontopathic bacteria in gestational diabetic and non-diabetic women | Ganiger K. and Sridharan S. and Rahul A. and Satyanarayana A. | 4 |
| Oral microbiome of deep and shallow dental pockets in chronic periodontitis. | Ge, Xiuchun and Rodriguez, Rafael and Trinh, My and Gunsolley, John and Xu, Ping | 4 |
| Checkerboard DNA-DNA hybridization technology using digoxigenin detection | Gellen L.S. and Wall-Manning G.M. and Sissons C.H. | 4 |
| Gingival crevice microbiota from Chinese patients with gingivitis or necrotizing ulcerative gingivitis | Gmur R. and Wyss C. and Xue Y. and Thurnheer T. and Guggenheim B. | 4 |
| The effect of a supragingival plaque-control regimen on the subgingival microbiota in smokers and never-smokers: Evaluation by real-time polymerase chain reaction | Gomes, S. C. and Nonnenmacher, C. and Susin, C. and Oppermann, R. V. and Mutters, R. and Marcantonio, R. A. C. | 4 |
| Detection of Helicobacter pylori, Enterococcus faecalis, and Pseudomonas aeruginosa in the subgingival biofilm of HIV-infected subjects undergoing HAART with chronic periodontitis. | Goncalves, L. de S. and Souto, R. and Colombo, A. P. V. | 4 |
| Identification of Actinobacillus actinomycetemcomitans: polymerase chain reaction amplification of lktA-specific sequences | Goncharoff, P. and Figurski, D. H. and Stevens, R. H. and Fine, D. H. | 4 |
| Antimicrobial systems of human whole saliva in relation to dental caries, cariogenic bacteria, and gingival inflammation in young adults. | Grahn, E and Tenovuo, J and Lehtonen, O P and Eerola, E and Vilja, P | 4 |
| Relationship between herpesviruses and periodontopathogens in patients with HIV and periodontitis. | Grande, Sabrina R and Imbronito, Ana V and Okuda, Osmar S and Pannuti, Claudio M and Nunes, Fabio D and Lima, Luiz A | 4 |
| Oral health and plaque microbial profile in juvenile idiopathic arthritis | Grevich S. and Lee P. and Leroux B. and Ringold S. and Darveau R. and Henstorf G. and Berg J. and Kim A. and Velan E. and Kelly J. and Baltuck C. and Reeves A. and Leahey H. and Hager K. and Brittnacher M. and Hayden H. and Miller S. and McLean J. and Stevens A. AO - Grevich, Sriharsha; ORCID: https://orcid.org/0000-0001-8193-7580 | 4 |
| Oral microbial profile in juvenile idiopathic arthritis | Grevich S. and Lee P. and McLean J. and Leroux B. and Ringold S. and Hager K. and Brittnacher M. and Hayden H. and Miller S. and Stevens A. | 4 |
| Oral health and anti-citrullinated peptide antibodies (ACPA) in juvenile idiopathic arthritis | Grevich S. and Lee P. and Ringold S. and Leroux B. and Leahey H. and Yuasa M. and Foster J. and Sokolove J. and Lahey L. and Robinson W. and Newsom J. and Stevens A. | 4 |
| Detection and measurement of oral malodor in chronic periodontitis patients and its correlation with levels of select oral anaerobes in subgingival plaque. | Grover, H S and Blaggana, Anshu and Jain, Yashika and Saini, Neha | 4 |
| Detection and quantification of periodontal pathogens in smokers and never-smokers with chronic periodontitis by real-time polymerase chain reaction. | Guglielmetti, Mariana R and Rosa, Ecinele F and Lourencao, Daniele S and Inoue, Gislene and Gomes, Elaine F and De Micheli, Giorgio and Mendes, Fausto Medeiros and Hirata, Rosario D C and Hirata, Mario H and Pannuti, Claudio M | 4 |
| Profiling the oral microbiomes in patients with Alzheimer's disease | Guo H. and Li B. and Yao H. and Liu D. and Chen R. and Zhou S. and Ji Y. and Zeng L. and Du M. AO - Guo, Haiying; ORCID: https://orcid.org/0000-0002-6853-6149 AO - Du, Minquan;... | 4 |
| Salivary IgA and IgG Antibody Responses against Periodontitis-Associated Bacteria in Crohn's Disease | Gursoy M. and Rautava J. and Pussinen P. and Kristoffersen A.K. and Enersen M. and Loimaranta V. and Gursoy U.K. AO - Gursoy, Mervi; ORCID: https://orcid.org/0000-0001-8545-6821 AO - Loima... | 4 |
| Relationship of cigarette smoking to the subgingival microbiota. | Haffajee, A D and Socransky, S S | 4 |
| Differences in the subgingival microbiota of Swedish and USA subjects who were periodontally healthy or exhibited minimal periodontal disease. | Haffajee, A. D. and Japlit, M. and Bogren, A. and Kent, R. L., Jr. and Goodson, J. M. and Socransky, S. S. | 4 |
| Association of Eubacterium nodatum and Treponema denticola with human periodontitis lesions. | Haffajee, A. D. and Teles, R. P. and Socransky, S. S. | 4 |
| Dysregulation of Porphyromonas gingivalis Agmatine Deiminase Expression in Alzheimer's Disease | Hamdi, A. and Baroudi, S. and Gharbi, A. and Babay, W. and Laaribi, A. B. and Kacem, I. and Mrabet, S. and Zidi, I. and Klibi, N. and Gouider, R. and Ouzari, H. I. | 4 |
| Dysregulation of <ovid:i>Porphyromonas gingivalis</ovid:i> Agmatine Deiminase Expression in Alzheimer's Disease | Hamdi, Asma and Baroudi, Sana and Gharbi, Alya and Babay, Wafa and Laaribi, Ahmed Baligh and Kacem, Imene and Mrabet, Saloua and Zidi, Ines and Klibi, Naouel and Gouider, Riadh and Ouzari, Hadda-Imene | 4 |
| Tannerella forsythensis prtH genotype and association with periodontal status. | Hamlet, Stephen M and Taiyeb-Ali, Tara B and Cullinan, Mary P and Westerman, Bill and Palmer, Janet E and Seymour, Gregory J | 4 |
| beta-lactamase production and antimicrobial susceptibility of subgingival bacteria from refractory periodontitis | Handal T. and Olsen I. and Walker C.B. and Caugant D.A. | 4 |
| Periodontitis and Porphyromonas gingivalis in preclinical stage of arthritis patients. | Hashimoto, M. and Yamazaki, T. and Hamaguchi, M. and Morimoto, T. and Yamori, M. and Asai, K. and Isobe, Y. and Furu, M. and Ito, H. and Fujii, T. and Terao, C. and Mori, M. and Matsuo, T. and Yoshitomi, H. and Yamamoto, K. and Yamamoto, W. and Bessho, K. and Mimori, T. | 4 |
| Subgingival distribution of Campylobacter rectus and Tannerella forsythensis in healthy children with primary dentition | Hayashi F. and Okada M. and Soda Y. and Miura K. and Kozai K. | 4 |
| Detection of sulphate-reducing bacteria in human saliva | Heggendorn F.L. and Goncalves L.S. and Dias E.P. and Silva Junior A. and Galvao M.M. and Lutterbach M.T. | 4 |
| Screening for <ovid:i>Selenomonas noxia</ovid:i> in a pediatric and adolescent patient population reveals differential oral prevalence across age groups | Hendricks, Katelyn and Hatch, Tyler and Kingsley, Karl and Howard, Katherine M. | 4 |
| Relationship between periodontal disease with oxidative stress markers in saliva in older adults | Hernandez-Monjaraz B. and Ruiz-Ramos M. and Mendoza-Nunez V.M. | 4 |
| Greater number of teeth in COPD patients with poor dental health correlates with worse daily respiratory symptoms | Heyman B. and Gaeckle N. and Criner A. and Criner G.J. | 4 |
| Effectiveness of the Sonicare sonic toothbrush on reduction of plaque, gingivitis, probing pocket depth and subgingival bacteria in adolescent orthodontic patients | Ho H.P. and Niederman R. | 4 |
| Clinical and Microbiological Efficacy of Pyrophosphate Containing Toothpaste: A Double-Blinded Placebo-Controlled Randomized Clinical Trial. | Hong, Inpyo and Lee, Hyun Gee and Keum, Hye Lim and Kim, Myong Ji and Jung, Ui-Won and Kim, KiJung and Kim, Su Yeon and Park, Taehun and Kim, Hye-Jin and Kim, Jin Ju and Sul, Woo Jun and An, Susun and Cha, Jae-Kook | 4 |
| T-RFLP-based differences in oral microbial communities as risk factor for development of oral diseases under stress. | Horz, H. P. and Haaf, A. ten and Kessler, O. and Yekta, S. S. and Seyfarth, I. and Hettlich, M. and Lampert, F. and Kupper, T. and Conrads, G. | 4 |
| A NEARLY FATAL DOG BITE DUE TO RARE CAPNOCYTOPHAGA OCHRACEA-INDUCED SEPTICEMIA IN AN ASPLENIC PATIENT | Hossain, S. and Khair, T. and Sami, M. and Gulati, U. D. A. Y. and Bowden, F. and Colaianni, L. E. E. and Singh, N. and Anwar, F. and Cole, R. | 4 |
| Effect of Full-mouth Disinfection Protocol on Glycaemic Control and Subgingival Microbiota in Patients with Type 1 and Type 2 Diabetes | Hropot Plesko N. and Skaleric E. and Seme K. and Janez A. and Skaleric U. and Gaspirc B. | 4 |
| The effects of a zinc citrate dentifrice on bacteria found on oral surfaces | Hu D. and Sreenivasan P.K. and Zhang Y.P. and De Vizio W. | 4 |
| Effect of Helicobacter pylori infection on chronic periodontitis by the change of microecology and inflammation | Hu Z. and Zhang Y. and Li Z. and Yu Y. and Kang W. and Han Y. and Geng X. and Ge S. and Sun Y. | 4 |
| Polybacterial challenge enhances HIV reactivation in latently infected macrophages and dendritic cells | Huang C.B. and Alimova Y.V. and Strange S. and Ebersole J.L. | 4 |
| The first study to detect co-infection of Entamoeba gingivalis and periodontitis-associated bacteria in dental patients in Taiwan | Huang J.M. and Ting C.C. and Chen Y.C. and Yuan K. and Lin W.C. | 4 |
| Chair-side quantitative oral-microflora screening for assessing familial correlation of periodontal status and caries prevalence | Huang Y.-K. and Lee W.-F. and Wang M.-J. and Chang Y.-H.S. and Tchaou W.-S. and Chang W.-J. and Lee S.-Y. and Sheu J.-R. and Teng N.-C. | 4 |
| Molecular identification of Capnocytophaga species from the oral cavity of patients with chronic periodontitis and healthy individuals. | Idate, Ulka and Bhat, Kishore and Kotrashetti, Vijayalakshmi and Kugaji, Manohar and Kumbar, Vijay | 4 |
| Detection and comparison of prevalence of Porphyromonas gingivalis through culture and Real Time-polymerase chain reaction in subgingival plaque samples of chronic periodontitis and healthy individuals. | Ingalagi, Preeti and Bhat, Kishore G and Kulkarni, R D and Kotrashetti, Vijayalakshmi S and Kumbar, Vijay and Kugaji, Manohar | 4 |
| Evaluation of salivary aspartate aminotransferase enzyme level in smoker patients with peptic ulcer in relation to periodontal condition | Irhayyim N.S. and Ahmed M.A.A. and Mahmood H.J. | 4 |
| Investigation of pediatric specifications for the Salivary Multi Test saliva test system | Irie Y. and Tatsukawa N. and Iwamoto Y. and Nakano M. and Ogasawara T. and Sakurai K. and Mitsuhata C. and Kozai K. | 4 |
| Porphyromonas gingivalis infection in the oral cavity is associated with elevated galactose-deficient IgA1 and increased nephritis severity in IgA nephropathy | Ito, S. and Misaki, T. and Nagasawa, Y. and Nomura, R. and Naka, S. and Fukunaga, A. and Matsuoka, D. and Matayoshi, S. and Matsumoto-Nakano, M. and Nakano, K. | 4 |
| Oral bacteria in the occluded arteries of patients with Buerger disease | Iwai T. and Inoue Y. and Umeda M. and Huang Y. and Kurihara N. and Koike M. and Ishikawa I. | 4 |
| Markers of periodontal infection and preterm birth | Jarjoura K. and Devine P.C. and Perez-Delboy A. and Herrera-Abreu M. and D'Alton M. and Papapanou P.N. | 4 |
| FimA gene polymorphism of Porphyromonas gingivalis in root canals and clinical characteristics of patients with chronic apical periodontitis. | Jia, Xi-Jin and Li, Hai-Feng | 4 |
| Nitrate-rich diet alters the composition of the oral microbiota in periodontal recall patients. | Jockel-Schneider, Yvonne and Schlagenhauf, Ulrich and Stolzel, Peggy and Gossner, Sophia and Carle, Reinhold and Ehmke, Benjamin and Prior, Karola and Hagenfeld, Daniel | 4 |
| Detection and measurement of oral malodour in periodontitis patients | John M. and Vandana K.L. | 4 |
| Periodontal disease and its association to endothelial dysfunction and clinical changes in limited systemic sclerosis: A case-control study | Jud P. and Wimmer G. and Meinitzer A. and Strohmaier H. and Schwantzer G. and Moazedi-Furst F. and Schweiger L. and Brodmann M. and Hafner F. and Arefnia B. AO - Arefnia, Behrouz; ORCID: https://orcid.org/0000-0002-5685-3796 AO - Sch... | 4 |
| Oral health conditions in children with idiopathic nephrotic syndrome: a cross-sectional study | Kaczmarek U. and Wrzyszcz-Kowalczyk A. and Jankowska K. and Prosciak K. and Mysiak-Debska M. and Przywitowska I. and Makulska I. AO - Kaczmarek, Urszula; ORCID: https://orcid.org/0000-0002-9692-283X AO - ... | 4 |
| Periopathogenic bacteria in dental plaque of Congolese patients with periodontitis: A pilot study. | Kalala-Kazadi, Em and Sekele-Issouradi, Jean-Paul and Bolenge-Ileboso, Jaques and Lasserre, Jerome F and Mantshumba-Milolo, Augustin and Ntumba-Mulumba, Hubert and Brecx, Michel C | 4 |
| Detection of odoriferous subgingival and tongue microbiota in diabetic and nondiabetic patients with oral malodor using polymerase chain reaction | Kamaraj D.R. and Bhushan K.S. and Laxman V.K. and Mathew J. | 4 |
| Oral microbial dysbiosis and amyloid pathology in cognitively normal subjects | Kamer A.R. and Gulivindala D. and Pushalkar S. and Li Q. and Glodzik L. and Butler T. and Pirraglia E. and Li Y. and Annam K. and Corby P. and Zetterberg H. and Blennow K. and Saxena D. and De leon M.J. | 4 |
| Periodontal dysbiosis associates with reduced csf abeta42 in cognitively normal elderly | Kamer A.R. and Pushalkar S. and Gulivindala D. and Butler T. and Li Y. and Annam K.R.C. and Glodzik L. and Ballman K.V. and Corby P.M. and Blennow K. and Zetterberg H. and Saxena D. and de Leon M.J. | 4 |
| Ex vivo Detection of Amyloid-beta in Naturally Formed Oral Biofilm | Kanagasingam S. and Von Ruhland C. and Welbury R. and Singhrao S.K. | 4 |
| Assessment of Oral Health Knowledge, Attitudes, and Behaviours among University Students in the Asir Region-Saudi Arabia: A Cross-Sectional Study. | Kandasamy, Geetha and Almeleebia, Tahani | 4 |
| Alveolar bone loss associated with age-related macular degeneration in males | Karesvuo P. and Gursoy U.K. and Pussinen P.J. and Suominen A.L. and Huumonen S. and Vesti E. and Kononen E. | 4 |
| Quantitative detection of volatile sulfur compound-producing microorganisms in oral specimens using real-time PCR | Kato H. and Yoshida A. and Awano S. and Ansai T. and Takehara T. | 4 |
| Oral infectious bacteria in dental plaque and saliva as risk factors in patients with esophageal cancer | Kawasaki M. and Ikeda Y. and Ikeda E. and Takahashi M. and Tanaka D. and Nakajima Y. and Arakawa S. and Izumi Y. and Miyake S. AO - Kawasaki, Machiko; ORCID: https://orcid.org/0000-0002-0082-5075 AO - Tak... | 4 |
| Usage of commonly used mouthwash on alterations in salivary pH | Kesava Priya S. and Vishnu Priya V. and Gayathri R. | 4 |
| Prevalence of Human Papillomavirus (HPV)-16 in Different Dental Infections in the Lebanese Population | Khalil, Wael and Alaa El Din, Ferdos and Jaffal, Marwa and Kanj, Abd El Hadi and Nabbouh, Ali and Kurban, Mazen and Rahal, Elias A. and Matar, Ghassan M. | 4 |
| Cross-sectional comparisons of subgingival microbiome and gingival fluid inflammatory cytokines in periodontally healthy vegetarians versus non-vegetarians | Khocht A. and Orlich M. and Paster B. and Bellinger D. and Lenoir L. and Irani C. and Fraser G. AO - Khocht, Ahmed; ORCID: https://orcid.org/0000-0003-3226-453X | 4 |
| Periodontitis associated with Chediak-Higashi syndrome in a young African American male | Khocht A. and Viera-Negron Y.E. and Ameri A. and Abdelsayed R. | 4 |
| Subgingival microbiota in adult Down syndrome periodontitis | Khocht A. and Yaskell T. and Janal M. and Turner B.F. and Rams T.E. and Haffajee A.D. and Socransky S.S. | 4 |
| Comparison of the preventive effects of slightly acidic HOCL mouthwash and CHX mouthwash for oral diseases | Kim Y.-R. and Nam S.-H. | 4 |
| Active matrix metalloproteinase-8 and periodontal bacteria depending on periodontal status in patients with rheumatoid arthritis | Kirchner A. and Jager J. and Krohn-Grimberghe B. and Patschan S. and Kottmann T. and Schmalz G. and Mausberg R.F. and Haak R. and Ziebolz D. AO - Ziebolz D.; ORCID: https://orcid.org/0000-0002-9810-2368 | 4 |
| Prediction of periodontopathic bacteria in dental plaque of periodontal healthy subjects by measurement of volatile sulfur compounds in mouth air | Kishi M. and Ohara-Nemoto Y. and Takahashi M. and Kishi K. and Kimura S. and Aizawa F. and Yonemitsu M. | 4 |
| Increase in detectable opportunistic bacteria in the oral cavity of orthodontic patients | Kitada, K. and de Toledo, A. and Oho, T. | 4 |
| Colonization pattern of periodontal bacteria in Japanese children and their mothers | Kobayashi N. and Ishihara K. and Sugihara N. and Kusumoto M. and Yakushiji M. and Okuda K. | 4 |
| Aggregatibacter actinomycetemcomitans-induced hypercitrullination links periodontal infection to autoimmunity in rheumatoid arthritis | Konig, M. F. and Abusleme, L. and Reinholdt, J. and Palmer, R. J. and Sampson, K. and Teles, R. P. and Nigrovic, P. A. and Rosen, A. and Sokolove, J. and Giles, J. T. and Moutsopoulos, N. M. and Andrade, F. | 4 |
| Detection of <ovid:i>Tannerella forsythia</ovid:i> and/or <ovid:i>Prevotella intermedia</ovid:i> might be useful for microbial predictive markers for the outcome of initial periodontal treatment in Koreans | Kook JoongKi, Kook JoongKi and Sakamoto, T. and Nishi, K. and Kim MiKwang, Kim MiKwang and Seong JinHyo, Seong JinHyo and Son YoungNam, Son YoungNam and Kim DongKie, Kim DongKie | 4 |
| Partial expression of Papillon-Lefevre Syndrome | Kothiwale S. and Mathur S. | 4 |
| Prevalence of Staphylococcus aureus and methicillin resistant Staphylococcus aureus (MRSA) in the oral cavity. | Koukos, G. and Sakellari, D. and Arsenakis, M. and Tsalikis, L. and Slini, T. and Konstantinidis, A. | 4 |
| Effect of smoking on subgingival microflora of patients with periodontitis in Japan. | Kubota, Michiya and Tanno-Nakanishi, Mariko and Yamada, Satoru and Okuda, Katsuji and Ishihara, Kazuyuki | 4 |
| Detection of <ovid:i>Porphyromonas gingivalis</ovid:i> and <ovid:i>Treponema denticola</ovid:i> in chronic and aggressive periodontitis patients: A comparative polymerase chain reaction study | Kumawat, Ramniwas M. and Ganvir, Sindhu M. and Hazarey, Vinay K. and Qureshi, Asifa and Purohit, Hemant J. | 4 |
| Real-Time PCR Method as Diagnostic Tool for Detection of Periodontal Pathogens in Patients with Periodontitis | Kuret, S. and Kalajzic, N. and Ruzdjak, M. and Grahovac, B. and Jezina Buselic, M. A. and Sardelic, S. and Delic, A. and Susak, L. and Sutlovic, D. | 4 |
| Differential analysis of culturable and unculturable subgingival target microorganisms according to the stages of periodontitis | Lafaurie G.I. and Castillo D.M. and Iniesta M. and Sanz M. and Gomez L.A. and Castillo Y. and Pianeta R. and Delgadillo N.A. and Neuta Y. and Diaz-Baez D. and Herrera D. | 4 |
| qPCR assay optimisation for a clinical study comparing oral health risk in Rett syndrome | Lai, Y. Y. L. and Downs, J. and Leishman, S. and Leonard, H. M. and Walsh, L. J. and Zafar, S. | 4 |
| Periodontal infection profiles in type 1 diabetes | Lalla E. and Kaplan S. and Chang S.-M.J. and Roth G.A. and Celenti R. and Hinckley K. and Greenberg E. and Papapanou P.N. | 4 |
| Oral, intestinal, and skin bacteria in ventral hernia mesh implants | Langbach, O. and Kristoffersen, A. K. and Abesha-Belay, E. and Enersen, M. and Rokke, O. and Olsen, I. | 4 |
| Periodontal disease and coronary heart disease: an epidemiological and microbiological study. | Latronico, Matteo and Segantini, Alessandro and Cavallini, Fabrizio and Mascolo, Andrea and Garbarino, Federico and Bondanza, Sara and Debbia, Eugenio A and Blasi, Giorgio | 4 |
| Periodontal Pathogens and Associated Intrathecal Antibodies in Early Stages of Alzheimer's Disease | Laugisch O. and Johnen A. and Maldonado A. and Ehmke B. and Burgin W. and Olsen I. and Potempa J. and Sculean A. and Duning T. and Eick S. | 4 |
| Periodontopathogens in rheumatoid arthritis and periodontal disease | Laugisch O. and Moeller B. and Kantyka T. and Vernables P.J. and Villiger P.M. and Sculean A. and Potempa J. and Eick S. | 4 |
| Knock-on effect of periodontitis to the pathogenesis of Alzheimer's disease? | Leblhuber F. and Huemer J. and Steiner K. and Gostner J.M. and Fuchs D. | 4 |
| Lactobacillus plantarum Lipoteichoic Acids Possess Strain-Specific Regulatory Effects on the Biofilm Formation of Dental Pathogenic Bacteria | Lee D. and Im J. and Park D.H. and Jeong S. and Park M. and Yoon S. and Park J. and Han S.H. | 4 |
| Detection of Porphyromonas gingivalis in the amniotic fluid in pregnant women with a diagnosis of threatened premature labor. | Leon, Ruben and Silva, Nora and Ovalle, Alfredo and Chaparro, Alejandra and Ahumada, Alexis and Gajardo, Marta and Martinez, Maria and Gamonal, Jorge | 4 |
| Subgingival microflora in chronic obstructive pulmonary disease | Leuckfeld I. and Olsen I. and Geiran O. and Bjortuft O. and Paster B.J. | 4 |
| Geographic Variation Did Not Affect the Predictive Power of Salivary Microbiota for Caries in Children With Mixed Dentition | Li S. and Huang S. and Guo Y. and Zhang Y. and Zhang L. and Li F. and Tan K. and Lu J. and Chen Z. and Guo Q. and Tang Y. and Teng F. and Yang F. | 4 |
| The oral microbiome of pregnant women facilitates gestational diabetes discrimination | Li X. and Ma X. and Zhang B. and Zhang J. and Wang W. and Sun C. and Wang Y. and Zheng J. and Chen H. and Tao J. and Wang H. and Zhang F. and Wang J. and Zhang H. | 4 |
| Oral diseases: from detection to diagnostics. | Ligtenberg, Antoon J M and de Soet, Johannes J and Veerman, Enno C I and Amerongen, Arie V Nieuw | 4 |
| Lipopolysaccharide, a possible molecular mediator between periodontitis and coronary artery disease | Liljestrand J.M. and Paju S. and Buhlin K. and Persson G.R. and Sarna S. and Nieminen M.S. and Sinisalo J. and Mantyla P. and Pussinen P.J. AO - Liljestrand, John M.; ORCID: https://orcid.org/0000-0001-6562-2458 | 4 |
| Immunologic burden links periodontitis to acute coronary syndrome | Liljestrand J.M. and Paju S. and Pietiainen M. and Buhlin K. and Persson G.R. and Nieminen M.S. and Sinisalo J. and Mantyla P. and Pussinen P.J. AO - Sinisalo, Juha; ORCID: https://orcid.org/0000-0002-0169-5137 | 4 |
| Differences in interleukin-8 plasma levels between diabetic patients and healthy individuals independently on their periodontal status | Linhartova P.B. and Kavrikova D. and Tomandlova M. and Poskerova H. and Rehka V. and Dusek L. and Holla L.I. | 4 |
| Rapid specific detection of oral bacteria using Cas13-based SHERLOCK | Liu J. and Carmichael C. and Hasturk H. and Shi W. and Bor B. AO - Bor, Batbileg; ORCID: https://orcid.org/0000-0002-1797-1730 | 4 |
| "Magnet" Based on Activated Silver Nanoparticles Adsorbed Bacteria to Predict Refractory Apical Periodontitis Via Surface-Enhanced Raman Scattering | Liu, X. and Jiang, S. and Zhang, T. and Xu, Z. and Liu, L. and Zhang, Z. and Pan, S. and Li, Y. | 4 |
| Epidemiological and etiological aspects of dental caries development | Loban G.A. and Faustova M.O. and Chereda V.V. and Ananieva M.M. | 4 |
| Rapid multiplex real-time PCR method for the detection and quantification of selected cariogenic and periodontal bacteria | Lochman J. and Zapletalova M. and Poskerova H. and Holla L.I. and Linhartova P.B. | 4 |
| Dental Caries and Periodontitis: Contrasting Two Infections That Have Medical Implications | Loesche W. | 4 |
| Effects of metronidazole plus amoxicillin as the only therapy on the microbiological and clinical parameters of untreated chronic periodontitis. | Lopez, Nestor J and Socransky, Sigmund S and Da Silva, Isabel and Japlit, Michele R and Haffajee, Anne D | 4 |
| Putative periodontopathic bacteria and herpesviruses in pregnant women: a case-control study | Lu H. and Zhu C. and Li F. and Xu W. and Tao D. and Feng X. | 4 |
| Salivary detection of periodontopathic bacteria in Fanconi's anemia patients. | Lyko, Karine and Bonfim, Carmem and Benelli, Elaine Machado and Torres-Pereira, Cassius Carvalho and Amenabar, Jose Miguel | 4 |
| Effect of lemon essential oil on halitosis | Ma L. and Pang C. and Yan C. and Chen J. and Wang X. and Hui J. and Zhou L. and Zhang X. AO - Zhang, Xiangyu; ORCID: https://orcid.org/0000-0001-5350-5879 | 4 |
| Detection and enumeration of periodontopathogenic bacteria in subgingival biofilm of pregnant women | Machado F.C. and Cesar D.E. and Assis A.V.D.A. and Ribeiro R.A. and Diniz C.G. | 4 |
| Frequency of putative periodontal pathogens among type 1 diabetes mellitus: a case-control study | Mahalakshmi K. and Arangannal P. and Santoshkumari AO - Mahalakshmi, Krishnan; ORCID: https://orcid.org/0000-0003-2753-9092 | 4 |
| Prevelance of periodontopathogenic bacteria in subgingival biofilm and atherosclerotic plaques of patients undergoing coronary revascularization surgery. | Mahendra, Jaideep and Mahendra, Little and Felix, John and Romanos, Georgios | 4 |
| Role of Periodontal Bacteria, Viruses, and Placental <ovid:i>mir155</ovid:i> in Chronic Periodontitis and Preeclampsia-A Genetic Microbiological Study | Mahendra, Jaideep and Mahendra, Little and Mugri, Maryam H. and Sayed, Mohammed E. and Bhandi, Shilpa and Alshahrani, Rahaf Turki and Balaji, Thodur Madapusi and Varadarajan, Saranya and Tanneeru, Swetha and P, Abirami Nayaki Rao and Srinivasan, Sruthi and Reda, Rodolfo and Testarelli, Luca and Patil, Shankargouda | 4 |
| Prevalence of Periodontal Disease and Periodontopathic Bacteria in Anti-Cyclic Citrullinated Protein Antibody-Positive At-Risk Adults Without Arthritis | Mankia K. and Cheng Z. and Do T. and Hunt L. and Meade J. and Kang J. and Clerehugh V. and Speirs A. and Tugnait A. and Hensor E.M.A. and Nam J.L. and Devine D.A. and Emery P. | 4 |
| A combined immunofluorescence and flow cytometry assay for the detection of dental plaque microorganisms | Manti A. and Baffone W. and Ciandrini E. and Campana R. and Dominici S. and Papa S. | 4 |
| Presence of periodontopathic bacteria in coronary arteries from patients with chronic periodontitis | Marcelino S.L. and Gaetti-Jardim E. and Nakano V. and Canonico L.A.D. and Nunes F.D. and Lotufo R.F.M. and Pustiglioni F.E. and Romito G.A. and Avila-Campos M.J. | 4 |
| Detection of periodontal bacterial DNA in serum and synovial fluid in refractory rheumatoid arthritis patients | Martinez-Martinez R.E. and Abud-Mendoza C. and Patino-Marin N. and Rizo-Rodriguez J.C. and Little J.W. and Loyola-Rodriguez J.P. | 4 |
| Characterization of periodontal biofilm in down syndrome patients: A comparative study | Martinez-Martinez R.E. and Loyola-Rodriguez J.P. and Bonilla-Garro S.E. and Patino-Marin N. and Haubek D. and Amano A. and Poulsen K. | 4 |
| [The physicochemical and microbiological characteristics of saliva during and after pregnancy]. | Martinez-Pabon, Maria C and Martinez Delgado, Cecilia M and Lopez-Palacio, Ana M and Patino-Gomez, Lina M and Arango-Perez, Eduin A | 4 |
| Effects of mouthwash on periodontal pathogens and glycemic control in patients with type 2 diabetes mellitus | Matayoshi, Saaya and Tojo, Fumikazu and Suehiro, Yuto and Okuda, Makoto and Takagi, Misato and Ochiai, Marin and Kadono, Maika and Mikasa, Yusuke and Okawa, Rena and Nomura, Ryota and Itoh, Yoshito and Itoh, Naoto and Nakano, Kazuhiko | 4 |
| Dental health in advanced age and Alzheimer's Disease: A possible link with bacterial toxins entering the brain?. | Maurer, Konrad and Rahming, Sven and Prvulovic, David | 4 |
| Periodontitis and Porphyromonas gingivalis in patients with rheumatoid arthritis. | Mikuls, T. R. and Payne, J. B. and Yu, F. and Thiele, G. M. and Reynolds, R. J. and Cannon, G. W. and Markt, J. and McGowan, D. and Kerr, G. S. and Redman, R. S. and Reimold, A. and Griffiths, G. and Beatty, M. and Gonzalez, S. M. and Bergman, D. A. and Hamilton, B. C., III and Erickson, A. R. and Sokolove, J. and Robinson, W. H. and Walker, C. and Chandad, F. and O'Dell, J. R. | 4 |
| 'Omics' approaches to study the oral microbiome | Mira A. | 4 |
| Increased interleukin-18 in patients with juvenile idiopathic arthritis and early attachment loss | Miranda L.A. and Fischer R.G. and Sztajnbok F.R. and Johansson A. and Figueredo C.M.S. and Gustafsson A. | 4 |
| Influence of glycemic control on the levels of subgingival periodontal pathogens in patients with generalized chronic periodontitis and type 2 diabetes. | Miranda, Tamires Szeremeske and Feres, Magda and Retamal-Valdes, Belen and Perez-Chaparro, Paula Juliana and Maciel, Suellen Silva and Duarte, Poliana Mendes | 4 |
| Quantitative analysis of streptococcus mutans, streptococcus sobrinus and streptococcus sanguinis and their association with early childhood caries | Mitrakul K. and Akarapipatkul B. and Thammachat P. | 4 |
| Detection of heat shock proteins but not superantigen by isolated oral bacteria from patients with Behcet's disease | Miura T. and Ishihara K. and Kato T. and Kimizuka R. and Miyabe H. and Ando T. and Uchiyama T. and Okuda K. | 4 |
| Differences in oral microbiota as risk factors for periodontal disease in diabetic subjects | Mizushiri S. and Daimon M. and Murakami H. and Murabayashi M. and Matsuhashi Y. and Kamba A. | 4 |
| One Year Follow-Up of a 4-Year-Old Caucasian Girl Diagnosed with Stage IV Grade C Localized Periodontitis | Moga, Radu-Andrei and Olteanu, Cristian Doru | 4 |
| Influence of Type 2 Diabetes on Prevalence of Key Periodontal Pathogens, Salivary Matrix Metalloproteinases, and Bone Remodeling Markers in Sudanese Adults with and without Chronic Periodontitis. | Mohamed, Hasaan Gassim and Idris, Shaza Bushra and Mustafa, Manal and Ahmed, Mutaz Faisal and Astrom, Anne Nordrehaug and Mustafa, Kamal and Ibrahim, Salah Osman | 4 |
| Evaluation of periodontal status and detection of <ovid:i>Dialister pneumosintes</ovid:i> in cerebral palsy individuals: A Case-Control study | Mohammed, S. Riyaz and Anand, Nithya and Chandrasekaran, S. C. and Mahalakshmi, Krishnan and Padmavathy, Kesavaram | 4 |
| Systemic Inflammation in Pregnant Women With Periodontitis and Preterm Prelabor Rupture of Membranes: A Prospective Case-Control Study | Mohr S. and Amylidi-Mohr S.K. and Stadelmann P. and Sculean A. and Persson R. and Eick S. and Surbek D.V. | 4 |
| Bacteria and oral cavity: A new story to tell | Mucchi D. | 4 |
| High incidence of Aggregatibacter actinomycetemcomitans infection in patients with cerebral infarction and diabetic renal failure: a cross-sectional study. | Murakami, M. and Suzuki, J. and Yamazaki, S. and Ikezoe, M. and Matsushima, R. and Ashigaki, N. and Aoyama, N. and Kobayashi, N. and Wakayama, K. and Akazawa, H. and Komuro, I. and Izumi, Y. and Isobe, M. | 4 |
| Red complex periodontal pathogens are risk factors for liver cirrhosis | Nagao Y. and Tanigawa T. | 4 |
| Oral microbiota, dental caries and periodontal status in smokeless tobacco chewers in Karnataka, India: a case-control study | Nagarajappa S. and Prasad K.V. | 4 |
| Presence of campylobacter rectus and cnmpositive streptococcus mutans strains in oral cavity was associated with urinary protein levels in igan patients | Nagasawa Y. and Misaki T. and Naka S. and Wato K. and Mizusaki K. and Ito S. and Inaba H. and Nomura R. and Hasuike Y. and Kuragano T. and Nakanishi T. and Matsumoto-Nakano M. and Nakano K. | 4 |
| Distribution of periodontopathic bacterial species in Japanese children with developmental disabilities. | Naka, S. and Yamana, A. and Nakano, K. and Okawa, R. and Fujita, K. and Kojima, A. and Nemoto, H. and Nomura, R. and Matsumoto, M. and Ooshima, T. | 4 |
| Detection and serotype distribution of Actinobacillus actinomycetemcomitans in cardiovascular specimens from Japanese patients | Nakano K. and Inaba H. and Nomura R. and Nemoto H. and Tamura K. and Miyamoto E. and Yoshioka H. and Taniguchi K. and Amano A. and Ooshima T. | 4 |
| Detection of oral bacteria in cardiovascular specimens | Nakano K. and Nemoto H. and Nomura R. and Inaba H. and Yoshioka H. and Taniguchi K. and Amano A. and Ooshima T. | 4 |
| Detection of cariogenic Streptococcus mutans in extirpated heart valve and atheromatous plaque specimens. | Nakano, Kazuhiko and Inaba, Hiroaki and Nomura, Ryota and Nemoto, Hirotoshi and Takeda, Munehiro and Yoshioka, Hideo and Matsue, Hajime and Takahashi, Toshiki and Taniguchi, Kazuhiro and Amano, Atsuo and Ooshima, Takashi | 4 |
| Clinical usefulness of novel immunochromatographic detection device for <ovid:i>Porphyromonas gingivalis</ovid:i> in evaluating effects of scaling and root planing and local antimicrobial therapy | Nakayama, Y. and Ogata, Y. and Hiromatsu, Y. and Imamura, K. and Suzuki, E. and Saito, A. and Shirakawa, S. and Nagano, T. and Gomi, K. and Morozumi, T. and Watanabe, K. and Akiishi, K. and Yoshie, H. | 4 |
| Porphyromonas gingivalis Outer Membrane Vesicles as the Major Driver of and Explanation for Neuropathogenesis, the Cholinergic Hypothesis, Iron Dyshomeostasis, and Salivary Lactoferrin in Alzheimer's Disease | Nara P.L. and Sindelar D. and Penn M.S. and Potempa J. and Griffin W.S.T. | 4 |
| Tobacco smoking and periodontal health in a Saudi Arabian population. | Natto, Suzan Bakur | 4 |
| Polymicrobial infection alter inflammatory microRNA in rat salivary glands during periodontal disease | Nayar G. and Gauna A. and Chukkapalli S. and Velsko I. and Kesavalu L. and Cha S. AO - Chukkapalli, Sasanka; ORCID: https://orcid.org/0000-0001-9063-6907 | 4 |
| The Comparison of Antimicrobial Effect of Nigella sativa Nanoparticle and Chlorhexidine Emulsion on the Most Common Dental Cariogenicic Bacteria. | Nazemi Salman, Bahareh and Sallah, Shilan and Abdi, Fatemeh and Salahi, Sarvenaz and Rostamizadeh, Kobra and Basir Shabestari, Samira | 4 |
| Comparative Molecular Analysis of Gram-Negative Bacteria in Primary Teeth with Irreversible Pulpitis or Periapical Pathology | Nelson-Filho P. and Ruviere D.B. and de Queiroz A.M. and de Paula-Silva F.W.G. and Silva R.A.B.D. and Lucisano M.P. and da Silva L.A.B. | 4 |
| Subgingival Periopathogens Assessment and Clinical Periodontal Evaluation of Gastric Cancer Patients-A Cross Sectional Pilot Study | Nicolae F.M. and Didilescu A.C. and Surlin P. and Ungureanu B.S. and Surlin V.M. and Patrascu S. and Ramboiu S. and Jelihovschi I. and Iancu L.S. and Ghilusi M. and Cucu M. and Gheonea D.I. | 4 |
| Characteristics of Microbial Distribution in Different Oral Niches of Oral Squamous Cell Carcinoma | Nie F. and Wang L. and Huang Y. and Yang P. and Gong P. and Feng Q. and Yang C. | 4 |
| Chewing gum containing citric acid reduces the burden of periodontal pathogens. | Nikawa, H. and Igarashi, S. and Takasu, O. and Tataka, H. and Harano, F. and Shinohara, S. and Makihira, S. and Takemoto, T. and Murayama, T. and Satoda, T. and Amano, H. and Kurihara, H. | 4 |
| Periodontal disease progression in type II non-insulin-dependent diabetes mellitus patients (NIDDM). Part II--Microbiological analysis using the BANA test. | Novaes, A B Jr and Gonzalez Gutierrez, F and Grisi, M F and Novaes, A B | 4 |
| Detection of periodontal bacteria in thrombi of patients with acute myocardial infarction by polymerase chain reaction. | Ohki, Takahiro and Itabashi, Yuji and Kohno, Takashi and Yoshizawa, Akihiro and Nishikubo, Shuichi and Watanabe, Shinya and Yamane, Genyuki and Ishihara, Kazuyuki | 4 |
| Clinical and immunological assessment of periodontal disease in japanese leprosy patients | Ohyama H. and Hongyo H. and Shimizu N. and Shimizu Y. and Nishimura F. and Nakagawa M. and Arai H. and Kato-Kogoe N. and Terada N. and Nagai A. and Takashiba S. and Kurihara H. and Nomura Y. and Murayama Y. | 4 |
| Characterization of specimens obtained by different sampling methods for evaluation of periodontal bacteria | Okada A. and Sogabe K. and Takeuchi H. and Okamoto M. and Nomura Y. and Hanada N. | 4 |
| High prevalence of colonization of oral cavity by respiratory pathogens in dysphagic patients | Ortega O. and Sakwinska O. and Mukherjee R. and Combremont S. and Jankovic I. and Parra C. and Zarzero S. and Nart J. and Clave P. | 4 |
| Oral microbiota in children with type 1 diabetes mellitus | Pachonski M. and Koczor-Rozmus A. and Mocny-Pachonska K. and Lanowy P. and Mertas A. and Jarosz-Chobot P. | 4 |
| Quantification of Porphyromonas gingivalis in chronic periodontitis patients associated with diabetes mellitus using real-time polymerase chain reaction. | Padmalatha, G. V. and Bavle, R. M. and Satyakiran, G. V. V. and Paremala, K. and Sudhakara, M. and Soumya Makarla, Soumya Makarla | 4 |
| Risk assessment for periodontal diseases | Page R.C. and Beck J.D. | 4 |
| The link between total antioxidant status, total oxidant status, arylesterase activity, and subgingival microbiota in psoriasis patients | Paksoy T. and Ustaoglu G. and Yaman D. and Arioz O. and Demirci M. and Unlu O. and Avci E. and Polat M. AO - Paksoy, Tugce; ORCID: https://orcid.org/0000-0001-6204-7304 AO - Demirci,... | 4 |
| Biomarkers of periodontitis and inflammation in ischemic stroke: A case-control study | Palm F. and Lahdentausta L. and Sorsa T. and Tervahartiala T. and Gokel P. and Buggle F. and Safer A. and Becher H. and Grau A.J. and Pussinen P. | 4 |
| Hydrogel-based radio frequency H<ovid:inf>2</ovid:inf>S sensor for in situ periodontitis monitoring and antibacterial treatment | Pan, J. and Li, X. and Sun, R. and Xu, Y. and Shi, Z. and Dai, C. and Wen, H. and Han, R. P. S. and Ye, Q. and Zhang, F. and Liu, Q. | 4 |
| Detection of selected periodontal bacteria in preschool children affected by early childhood caries | Pantuckova P. and Bartosova M. and Broukal Z. and Kukletova M. and Holla L.I. AO - Broukal, Zdenek; ORCID: https://orcid.org/0000-0002-5421-8625 | 4 |
| Contamination of interdental brushes by periodontopathogens | Papaioannou W. and Panis V. and Nakou M. and Mantzavinos Z. | 4 |
| Contribution of host genotype to the composition of health-associated supragingival and subgingival microbiomes | Papapostolou A. and Kroffke B. and Tatakis D.N. and Nagaraja H.N. and Kumar P.S. | 4 |
| Microbial analysis of saliva to identify oral diseases using a point-of-care compatible qpcr assay | Paque P.N. and Herz C. and Jenzer J.S. and Wiedemeier D.B. and Attin T. and Bostanci N. and Belibasakis G.N. and Bao K. and Korner P. and Fritz T. and Prinz J. and Schmidlin P.R. and Thurnheer T. and Wegehaupt F.J. and Mitsakakis K. and Peham J.R. | 4 |
| Salivary biomarkers for dental caries detection and personalized monitoring | Paque P.N. and Herz C. and Wiedemeier D.B. and Mitsakakis K. and Attin T. and Bao K. and Belibasakis G.N. and Hays J.P. and Jenzer J.S. and Kaman W.E. and Karpisek M. and Korner P. and Peham J.R. and Schmidlin P.R. and Thurnheer T. and Wegehaupt F.J. and Bostanci N. | 4 |
| The concept of "risk" and the emerging discipline of periodontal medicine | Paquette D.W. and Madianos P. and Offenbacher S. and Beck J.D. and Williams R.C. | 4 |
| Detection of periodontal pathogens in oral samples and cardiac specimens in patients undergoing aortic valve replacement: A pilot study | Pardo A. and Signoriello A. and Signoretto C. and Messina E. and Carelli M. and Tessari M. and De Manna N.D. and Rossetti C. and Albanese M. and Lombardo G. and Luciani G.B. | 4 |
| Kuwanon G: an antibacterial agent from the root bark of Morus alba against oral pathogens. | Park, K. M. and You, J. S. and Lee, H. Y. and Baek, N. I. and Hwang, J. K. | 4 |
| Microbiological and host factors are involved in promoting the periodontal failure of metaloceramic crowns | Passariello C. and Puttini M. and Virga A. and Gigola P. | 4 |
| Placental TLR recognition of salivary and subgingival microbiota is associated with pregnancy complications | Pax, K. and Buduneli, N. and Alan, M. and Meric, P. and Gurlek, O. and Dabdoub, S. M. and Kumar, P. S. | 4 |
| Effect of granulocytopenia on oral microbial relationships in patients with acute leukemia | Peterson D.E. and Minah G.E. and Reynolds M.A. and Weikel D.S. and Overholser C.D. and DePaola L.G. and Wade J.C. and Suzuki J.B. | 4 |
| The oral microbiota and periodontal health in orthodontic patients | Peterson, Brandon W. and Tjakkes, Geerten-Has and Renkema, Anne-Marie and Manton, David J. and Ren, Yijin | 4 |
| Relationship of a turbidity of an oral rinse with oral health and malodor in Vietnamese patients | Pham T.A. | 4 |
| Factors affecting oral malodor in periodontitis and gingivitis patients | Pham T.A. and Ueno M. and Shinada K. and Kawaguchi Y. | 4 |
| Clinical aspects and microbiology of HIV-associated periodontal lesions. | Piluso, S and Ficarra, G and Orsi, A and Gaglioti, D and Pierotti, P and Orlando, S | 4 |
| Presence of bacterial DNA in thrombotic material of patients with myocardial infarction | Pinon-Esteban P. and Nunez L. and Moure R. and Marron-Linares G.M. and Flores-Rios X. and Aldama-Lopez G. and Salgado-Fernandez J. and Calvino-Santos R. and Rebollal-Leal F. and Pan-Lizcano R. and Vazquez-Gonzalez N. and Bou G. and Tomas M. and Hermida-Prieto M. and Vazquez-Rodriguez J.M. AO - Pinon-Esteban P.; ORCID: https://orcid.org/0000-0001-6078-081X | 4 |
| Does Fusobacterium in Colorectal Cancer Sites Originate From the Oral Cavity? A Pilot Study | Plomp, Niels and Bertl, Kristina and Lydrup, Marie-Louise and Sjoberg, Klas and Harmsen, Hermie J. M. and Stavropoulos, Andreas | 4 |
| 4-Hydroxy-2-nonenal, an oxidative stress marker in crevicular fluid and serum in type 2 diabetes with chronic periodontitis. | Pradeep, A R and Agarwal, Esha and Bajaj, Pavan and Rao, Nishanth S | 4 |
| Sequencing of Porphyromonas gingivalis from saliva in patients with periodontitis and type 2 diabetes mellitus. | Preethi Radhakrishnan, Preethi Radhakrishnan and Rubini Anbalagan, Rubini Anbalagan and Ramya Barani, Ramya Barani and Monika Mani, Monika Mani and Seshadri, K. G. and Padma Srikanth, Padma Srikanth | 4 |
| Prevention of transmission of resistant bacteria between periodontal sites during subgingival application of antibiotics | Preus H.R. and Lassen J. and Aass A.M. and Christersson L.A. | 4 |
| Diversity and site-specificity of the oral microflora in the elderly. | Preza, D and Olsen, I and Willumsen, T and Grinde, B and Paster, B J | 4 |
| PCR detection of Streptococcus mutans and Aggregatibacter actinomycetemcomitans in dental plaque samples from Haitian adolescents | Psoter W.J. and Ge Y. and Russell S.L. and Chen Z. and Katz R.V. and Jean-Charles G. and Li Y. | 4 |
| The influence of smoking and race on adult periodontitis and serum IgG2 levels | Quinn, S. M. and Zhang, J. B. and Gunsolley, J. C. and Schenkein, H. A. and Tew, J. G. | 4 |
| Anti-phosphoryl Choline and hs - CRP in Serum of Atherosclerosis Cardiovascular Patient with Chronic Periodontitis | Radhi R.A. and Almosawi A.O.A. | 4 |
| No obvious role for suspicious oral pathogens in arthritis development | Rahajoe P.S. and de Smit M.J. and Raveling-Eelsing E. and Espina M.D.T. and Stobernack T. and Lisotto P. and Harmsen H.J.M. and van Dijl J.M. and Kertia N. and Vissink A. and Westra J. | 4 |
| Dysbiosis of the Subgingival Microbiome and Relation to Periodontal Disease in Association with Obesity and Overweight | Rahman B. and Al-Marzooq F. and Saad H. and Benzina D. and Al Kawas S. AO - Al-Marzooq, Farah; ORCID: https://orcid.org/0000-0001-5711-6264 | 4 |
| Periodontal status, salivary immunoglobulin, and microbial counts after short exposure to an isolated environment | Rai B. and Kaur J. | 4 |
| Estimation of salivary superoxide dismutase levels in chronic periodontitis patients with or without coronary heart disease | Ram A.J. and Vishnupriya V. and Ponnulakshmi R. and Gayathri R. and Madhan K. and Shyamaladevi B. and Selvaraj J. | 4 |
| Maternal Oral Health Influences Infant Salivary Microbiome | Ramadugu, K. and Bhaumik, D. and Luo, T. and Gicquelais, R. E. and Lee, K. H. and Stafford, E. B. and Marrs, C. F. and Neiswanger, K. and McNeil, D. W. and Marazita, M. L. and Foxman, B. | 4 |
| Role of stoll-like receptors 2 and 4 in stage 2 periodontitis patients with and without type 2 diabetes: A randomized clinical control trial | Ramesh A. and Varma S.R. and Ramamurthy S. and Al Shayeb M. and Shahwan M. and Atia Elkaseh A.M. and Khair A.M.B. and Mageet A. and Arif M. and Thomas B. and Jaganathan P. and Shetty S. and Sharmila K.P. | 4 |
| Biomarkers of cardiovascular disease are increased in untreated chronic periodontitis: A case control study | Ramirez J.H. and Parra B. and Gutierrez S. and Arce R.M. and Jaramillo A. and Ariza Y. and Contreras A. | 4 |
| Identification of helicobacter pylori and its connection with oral cancer in the oral cavity. A cross sectional study | Ramya S. and Preethi M. and Sivapathasundharam B. | 4 |
| Molecular analysis shows the presence of periodontal bacterial DNA in atherosclerotic plaques from patients with coronary artery disease | Rao A. and D'Souza C. and Subramanyam K. and Rai P. and Thomas B. and Gopalakrishnan M. and Karunasagar I. and Kumar B.K. AO - Rao, Amita; ORCID: https://orcid.org/0000-0001-7064-6841 AO - Thomas, B... | 4 |
| Can the Correlation of Periodontopathies with Gastrointestinal Diseases Be Used as Indicators in Severe Colorectal Diseases?. | Rat, Lavinia Alina and Moldovan, Andrada Florina and Trifan, Daniela Florina and Matis, Loredana and Murvai, Gelu Florin and Maris, Lavinia and Ghitea, Timea Claudia and Maghiar, Marius Adrian | 4 |
| Association between periodontal disease and cardiovascular disease | Rehman M.M. and Salama R.I. | 4 |
| Detection of oral bacterial DNA in synovial fluid | Reichert S. and Haffner M. and Keysser G. and Schafer C. and Stein J.M. and Schaller H.-G. and Wienke A. and Strauss H. and Heide S. and Schulz S. | 4 |
| Is periodontitis a prognostic factor in order to indicate antibodies against citrullinated peptides in patients with rheumatoid arthritis? | Reichert S. and Jurianz E. and Putz N. and Schlumberger W. and Dahnrich C. and Johannsen N. and Altermann W. and Schlaf G. and Keysser G. and Schaefer C. and Schaller H.-G. and Schulz S. | 4 |
| Use of floss/interdental brushes is associated with lower risk for new cardiovascular events among patients with coronary heart disease | Reichert S. and Schlitt A. and Beschow V. and Lutze A. and Lischewski S. and Seifert T. and Dudakliewa T. and Gawe R. and Werdan K. and Hofmann B. and Schaller H.-G. and Schulz S. | 4 |
| Association of levels of antibodies against citrullinated cyclic peptides and citrullinated aalpha-enolase in chronic and aggressive periodontitis as a risk factor of Rheumatoid arthritis: A case control study | Reichert S. and Schlumberger W. and Dahnrich C. and Hornig N. and Altermann W. and Schaller H.-G. and Schulz S. | 4 |
| Cardiovascular Disease and Periodontal Disease in Type 1 Diabetes | Remmers L.M. and Snell-Bergeon J. | 4 |
| Periodontitis: a future risk of acute coronary syndrome? A follow-up study over 3 years. | Renvert, Stefan and Ohlsson, Ola and Pettersson, Thomas and Persson, G Rutger | 4 |
| Absence of a specific subgingival microflora in adults with Down's syndrome | Reuland-Bosma W. and van der Reijden W.A. and van Winkelhoff A.J. | 4 |
| Detection of Scardovia wiggsiae and Bifidobacterium dentium in caries lesions - A pilot study | Rheinberg A. and Henne K. and Meyer-Lueckel H. and Conrads G. | 4 |
| Clinical and microbiological efficacy of an antimicrobial mouth rinse containing 0.05% cetylpyridinium chloride in patients with gingivitis. | Rioboo, M and Garcia, V and Serrano, J and O'Connor, A and Herrera, D and Sanz, M | 4 |
| A microbiological study of Papillon-Lefevre syndrome in two patients | Robertson K.L. and Drucker D.B. and James J. and Blinkhorn A.S. and Hamlet S. and Bird P.S. | 4 |
| Evaluation and association of periodontal status with levels of <ovid:i>Porphyromonas gingivalis</ovid:i> in chronic periodontitis with and without type 2 diabetes mellitus following nonsurgical periodontal therapy using quantitative polymerase chain reaction: an interventional study | Rode, Pranita Avinash and Kolte, Rajashri Abhay and Kolte, Abhay Pandurang and Purohit, Hemant Jyotiswarup and Swami, Renuka Kashi | 4 |
| Subgingival microbiota of mexicans with type 2 diabetes with different periodontal and metabolic conditions | Rodriguez-Hernandez A.-P. and Marquez-Corona M.L. and Pontigo-Loyola A.P. and Medina-Solis C.E. and Ximenez-Fyvie L.-A. | 4 |
| Factors Associated to Clinical and Radiographic Disease Progression in Patients with Early RA and First-degree Relatives: A 1-year Follow-up | Romero-Sanchez C. and Bello-Gualtero J.M. and De Avila J. and Lafaurie G. and Choueka P.C. and Tena C.P. and Giraldo-Q S. and Chaparro-Sanabria J.A. and Ramos-Casallas A. and Chila-M L. and Bautista-Molano W. | 4 |
| Tracking the functional meaning of the human oral-microbiome protein-protein interactions. | Rosa, Nuno and Campos, Bruno and Esteves, Ana Cristina and Duarte, Ana Sofia and Correia, Maria Jose and Silva, Raquel M and Barros, Marlene | 4 |
| Oral cavity is not a reservoir for Helicobacter pylori in infected patients with functional dyspepsia. | Rossi-Aguiar, V. P. S. and Navarro-Rodriguez, T. and Mattar, R. and Peres, M. P. S. de M. and Barbuti, R. C. and Silva, F. M. and Carrilho, F. J. and Eisig, J. N. | 4 |
| Microbial profile and endotoxin levels in primary periodontal lesions with secondary endodontic involvement. | Rovai, E. da S. and Matos, F. de S. and Kerbauy, W. D. and Cardoso, F. G. da R. and Martinho, F. C. and Oliveira, L. D. de and Valera, M. C. and Carvalho, C. A. T. | 4 |
| Simultaneous measurement of the viability, aggregation, and live and dead adherence of Streptococcus crista, Streptococcus mutans and Actinobacillus actinomycetemcomitans in human saliva in relation to indices of caries, dental plaque and periodontal disease | Rudney J.D. and Staikov R.K. | 4 |
| [Comparative study by pH-tests of the efficacy of antimicrobial agents in oral cavity]. | Rumiantsev, V A and Iusufov, M V and Khiutti, N V and Moskaleva, I V and Slobodina, E V | 4 |
| Association of leptin in periodontitis and acute myocardial infarction. | Rupasree Gundala, Rupasree Gundala and Chava, V. K. and Ramalingam, K. | 4 |
| Influence of maternal oral microbiome on newborn oral microbiome in healthy pregnancies | Russo M. and Calevo M.G. and D'Alessandro G. and Tantari M. and Migliorati M. and Piccardo I. and Perucchin P.P. and Arioni C. AO - Russo, Monica; ORCID: https://orcid.org/0000-0002-7589-426X | 4 |
| Retrospective assessment of clinical and microbiological factors affecting periimplant tissue conditions | Rutar A. and Lang N.P. and Buser D. and Burgin W. and Mombelli A. | 4 |
| Microbiological and immunological characteristics of young Moroccan patients with aggressive periodontitis with and without detectable Aggregatibacter actinomycetemcomitans JP2 infection | Rylev M. and Bek-Thomsen M. and Reinholdt J. and Ennibi O.-K. and Kilian M. | 4 |
| Relationship of clinical and microbiological variables in patients with type 1 diabetes mellitus and periodontitis | Sakalauskiene, J. and Kubilius, R. and Gleiznys, A. and Vitkauskiene, A. and Ivanauskiene, E. and Saferis, V. | 4 |
| A comparison in prevalence of Helicobacter pylori in the gingival crevicular fluid from subjects with periodontitis and healthy individuals using polymerase chain reaction. | Salehi, M. R. and Aboei, M. S. and Naghsh, N. and Hajisadeghi, S. and Ajami, E. | 4 |
| Quantitative PCR analysis of salivary pathogen burden in periodontitis | Salminen A. and Elisa Kopra K.A. and Hyvarinen K. and Paju S. and Mantyla P. and Buhlin K. and Nieminen M.S. and Sinisalo J. and Pussinen P.J. | 4 |
| Pro-inflammatory biomarkers during experimental gingivitis in patients with type 1 diabetes mellitus: a proof-of-concept study. | Salvi, Giovanni E and Franco, Lea M and Braun, Thomas M and Lee, Angie and Rutger Persson, Gosta and Lang, Niklaus P and Giannobile, William V | 4 |
| Association between <ovid:i>Aggregatibacter actinomycetemcomitans</ovid:i> and <ovid:i>Porphyromonas gingivalis</ovid:i> in subgingival plaque and clinical parameters, in Argentine patients with aggressive periodontitis | Sanchez, G. A. and Acquier, A. B. and Couto, A. de and Busch, L. and Mendez, C. F. | 4 |
| Evaluation of loop-mediated isothermal amplification method for efficient detection of the periodontopathic bacteria Porphyromonas gingivalis | Sangolli, M. N. and Kugaji, M. S. and Ray, S. K. and Bhat, K. G. | 4 |
| Relationship of cariogenic bacteria levels with periodontal status and root surface caries in elderly Japanese | Saotome Y. and Tada A. and Hanada N. and Yoshihara A. and Uematsu H. and Miyazaki H. and Senpuku H. | 4 |
| Periodontal condition and microbiology of healthy and diseased periodontal pockets in type 1 diabetes mellitus patients. | Sastrowijoto, S H and Hillemans, P and van Steenbergen, T J and Abraham-Inpijn, L and de Graaff, J | 4 |
| Helicobacter pylori in periodontal pockets of chronic periodontitis patients with and without type II diabetes mellitus: a randomized controlled trial. | Savita Sambashivaiah, Savita Sambashivaiah and Shivaprasad Bilichodmath, Shivaprasad Bilichodmath and Nanjammanni Nanjaiah, Nanjammanni Nanjaiah and Rithesh Kulal, Rithesh Kulal | 4 |
| Detection of <ovid:i>Porphyromonas gingivalis</ovid:i> in umbilical cord blood of new-born and in subgingival plaque of pregnant participants with periodontal disease and its association with pregnancy outcomes: An observational study | Savitha, J. N. and Bhavya, B. and Yadalam, Umesh and Khan, Safiya Fatima | 4 |
| Herpesviral-bacterial interrelationships in aggressive periodontitis | Saygun I. and Kubar A. and Ozdemir A. and Yapar M. and Slots J. | 4 |
| Quantitative analysis of association between herpesviruses and bacterial pathogens in periodontitis | Saygun I. and Kubar A. and Sahin S. and Sener K. and Slots J. | 4 |
| A split-mouth study on microbiological profile in clinical healthy teeth and implants related to key inflammatory mediators | Schierano, G. and Pejrone, G. and Roana, J. and Scalas, D. and Allizond, V. and Martinasso, G. and Pagano, M. and Canuto, R. A. and Cuffini, A. M. | 4 |
| Regular consumption of Lactobacillus reuteri-containing lozenges reduces pregnancy gingivitis: an RCT. | Schlagenhauf, U. and Jakob, L. and Eigenthaler, M. and Segerer, S. and Jockel-Schneider, Y. and Rehn, M. | 4 |
| MMP-8 and TIMP-1 are associated to periodontal inflammation in patients with rheumatoid arthritis under methotrexate immunosuppression - First results of a cross-sectional study | Schmalz G. and Davarpanah I. and Jager J. and Mausberg R.F. and Krohn-Grimberghe B. and Schmidt J. and Haak R. and Sack U. and Ziebolz D. AO - Ziebolz, Dirk; ORCID: https://orcid.org/0000-0002-9810-2368 | 4 |
| Associations of chairside salivary aMMP-8 findings with periodontal parameters, potentially periodontal pathogenic bacteria and selected blood parameters in systemically healthy adults | Schmalz G. and Hubscher A.E. and Angermann H. and Schmidt J. and Schmickler J. and Legler T.J. and Ziebolz D. | 4 |
| Dental and periodontal health, and microbiological and salivary conditions in patients with or without diabetes undergoing haemodialysis. | Schmalz, Gerhard and Schiffers, Nora and Schwabe, Sandra and Vasko, Radovan and Muller, Gerhard A and Haak, Rainer and Mausberg, Rainer F and Ziebolz, Dirk | 4 |
| Cross-Sectional Evaluation of Periodontal Status and Microbiologic and Rheumatoid Parameters in a Large Cohort of Patients With Rheumatoid Arthritis | Schmickler J. and Rupprecht A. and Patschan S. and Patschan D. and Muller G.A. and Haak R. and Mausberg R.F. and Schmalz G. and Kottmann T. and Ziebolz D. | 4 |
| Shared microbiome in gums and the lung in an outpatient population | Schmidlin P.R. and Fachinger P. and Tini G. and Graber S. and Seifert B. and Dombrowa S. and Irani S. | 4 |
| Periodontal pathogens and their role in cardiovascular outcome. | Schulz, Susanne and Schlitt, Axel and Hofmann, Britt and Schaller, Hans-Gunter and Reichert, Stefan | 4 |
| Association of Distinct Fine Specificities of Anti-Citrullinated Peptide Antibodies With Elevated Immune Responses to Prevotella intermedia in a Subgroup of Patients With Rheumatoid Arthritis and Periodontitis | Schwenzer A. and Quirke A.-M. and Marzeda A.M. and Wong A. and Montgomery A.B. and Sayles H.R. and Eick S. and Gawron K. and Chomyszyn-Gajewska M. and Lazarz-Bartyzel K. and Davis S. and Potempa J. and Kessler B.M. and Fischer R. and Venables P.J. and Payne J.B. and Mikuls T.R. and Midwood K.S. | 4 |
| Infrared Thermal Imaging for Diabetes Detection and Measurement | Selvarani A. and Suresh G.R. AO - Selvarani A.; ORCID: https://orcid.org/0000-0003-0195-2508 | 4 |
| Influence of a triclosan toothpaste on periodontopathic bacteria and periodontitis progression in cardiovascular patients: a randomized controlled trial | Seymour G.J. and Palmer J.E. and Leishman S.J. and Do H.L. and Westerman B. and Carle A.D. and Faddy M.J. and West M.J. and Cullinan M.P. | 4 |
| Connecting the dots: NETosis and the periodontitis-rheumatoid arthritis nexus | Shahbaz, M. and Al-Maleki, A. R. and Cheah, C. W. and Aziz, J. and Bartold, P. M. and Vaithilingam, R. D. | 4 |
| Evaluation of the antibacterial potential of polyphenols against periodontal pathogens | Shahzad M. and Ramage G. and Edwards C.A. and Combet E. | 4 |
| Salivary bacteria linked to oral cancers | Sharma D.C. | 4 |
| Association between respiratory disease in hospitalized patients and periodontal disease: A cross-sectional study | Sharma N. and Shamsuddin H. | 4 |
| Antimicrobial efficacy of the combinations of Acacia nilotica, Murraya koenigii L. sprengel, Eucalyptus hybrid and Psidium guajava on primary plaque colonizers. | Shekar, B. R. C. and Ramesh Nagarajappa, Ramesh Nagarajappa and Rupal Singh, Rupal Singh and Roopesh Thaku, Roopesh Thaku | 4 |
| Prevalence of oral pathogen Slackia exigua among clinical orthodontic and non-orthodontic saliva samples. | Shen, Ching and Simpson, Justin and Clawson, James Brigham and Lam, Steven and Kingsley, Karl | 4 |
| The subgingival microbiome associated with periodontitis in type 2 diabetes mellitus. | Shi, Baochen and Lux, Renate and Klokkevold, Perry and Chang, Michaela and Barnard, Emma and Haake, Susan and Li, Huiying | 4 |
| The prevalence of pathogenic periodontal microflora in healthy young adult smokers | Shiloah J. and Patters M.R. and Waring M.B. | 4 |
| Prevalence of cariogenic and periodontopathic bacteria in japanese children in the primary and mixed dentitions | Shimomura-Kuroki J. and Yamashita-Matsuda K. and Miyagawa Y. and Shimooka S. | 4 |
| Comparison of oral disease manifestation in patients with pulmonary disease and healthy group | Shirazian S. and Manifar S. and Gharabaghi M.A. and Keshvari Z. and Bahrami N. | 4 |
| Presence of Helicobacter pylori in supragingival dental plaque of individuals with periodontal disease and upper gastric diseases. | Silva, D. G. and Stevens, R. H. and Macedo, J. M. B. and Albano, R. M. and Falabella, M. E. V. and Fischer, R. G. and Veerman, E. C. and Tinoco, E. M. B. | 4 |
| A 16S rDNA-based nested PCR protocol to detect Campylobacter gracilis in oral infections | Siqueira Jr. J.F. and Rocas I.N. | 4 |
| Periodontopathic bacteria in young healthy subjects of different ethnic backgrounds in Los Angeles | Sirinian G. and Shimizu T. and Sugar C. and Slots J. and Chen C. | 4 |
| A clinical and microbiological study on the enantiomers of delmopinol | Sjodin T. and Nilner K. and Sparre B. and Bernet C. and Astrom M. | 4 |
| Recent concepts regarding gingival crevicular fluid | Sneha V. and Bhuvaneshwarri J. | 4 |
| To compare the effectiveness of chlorhexidine Extended Protection on patients with and without gingivitis - A clinical trial | Sripradha S. and Ramamurthy J. and Bhagyalakshmi T. | 4 |
| Development and validation of an instrument to assess oral health literacy in Norwegian adult dental patients. | Stein, L. and Pettersen, K. S. and Bergdahl, M. and Bergdahl, J. | 4 |
| Association between cigarette smoking, bacterial pathogens, and periodontal status | Stoltenberg J.L. and Osborn J.B. and Pihlstrom B.L. and Herzberg M.C. and Aeppli D.M. and Wolff L.F. and Fischer G.E. | 4 |
| Oral care and health in rheumatoid arthritis patients based on a self-assessment questionnaire investigation | Strandbygaard L.L. and Radwan-Oczko M. and Dus I. and Thomsen A.M. and Schmokel K. and Rasmussen C. | 4 |
| Members of subgingival plaque bacterial complexes are present in blood of patients with coronary artery disease | Suarez-Molina A. and Fong C. and Cifuentes-C L. and Guauque-Olarte S. | 4 |
| Adrenomedullin, periodontitis, diabetes-unraveling the equivocal relationship: A clinicobiochemical cross-sectional study. | Suchetha, A and Garg, Akanksha and Lakshmi, P and Bhat, Divya and Sapna, N and Apoorva, S M | 4 |
| Occurrence, population structure, and antimicrobial resistance of enterococci in marginal and apical periodontitis. | Sun JingLu, Sun JingLu and Song XiaoBo, Song XiaoBo and Kristiansen, B. E. and Kjaereng, A. and Willems, R. J. L. and Eriksen, H. M. and Sundsfjord, A. and Sollid, J. E. | 4 |
| Periodontal bacterial species in hopeless dentitions with severe periodontitis: comparison of levels before extraction and 90 days after immediate implant placement | Susin D. and Aquino D.R. and Cortelli J.R. and Costa F.O. and Duarte P.M. and Rovai E.S. and Cortelli S.C. | 4 |
| Correlations between the properties of saliva and metabolic syndrome A prospective observational study | Suzuki D. and Yamada S.-I. and Sakurai A. and Karasawa I. and Kondo E. and Sakai H. and Tanaka H. and Shimane T. and Kurita H. | 4 |
| The effects of cigarette smoking on the salivary and tongue microbiome. | Suzuki, Nao and Nakano, Yoshio and Yoneda, Masahiro and Hirofuji, Takao and Hanioka, Takashi | 4 |
| Detection of Helicobacter pylori DNA in the saliva of patients complaining of halitosis. | Suzuki, Nao and Yoneda, Masahiro and Naito, Toru and Iwamoto, Tomoyuki and Masuo, Yousuke and Yamada, Kazuhiko and Hisama, Kazuhiro and Okada, Ichizo and Hirofuji, Takao | 4 |
| High-resolution taxonomic profiling of the subgingival microbiome for biomarker discovery and periodontitis diagnosis. | Szafranski, Szymon P and Wos-Oxley, Melissa L and Vilchez-Vargas, Ramiro and Jauregui, Ruy and Plumeier, Iris and Klawonn, Frank and Tomasch, Jurgen and Meisinger, Christa and Kuhnisch, Jan and Sztajer, Helena and Pieper, Dietmar H and Wagner-Dobler, Irene | 4 |
| Oral lactobacilli in the pathogenesis of dental caries and periodontitis | Szkaradkiewicz A. | 4 |
| Effect of exopolysaccharides from cariogenic bacteria on human gingival fibroblasts | Szkaradkiewicz-Karpinska A.K. and Szkaradkiewicz A. | 4 |
| The Red and Orange Complex Subgingival Microbiome of Cognitive Impairment and Cognitively Normal Elderly with Periodontitis. | Tadjoedin, Fatimah Maria and Masulili, Sri Lelyati C and Rizal, Muhammad Ihsan and Kusdhany, Lindawati S and Turana, Yuda and Ismail, Raden Irawati and Bachtiar, Boy M | 4 |
| The subgingival cultivable bacteria of Albanian subjects with different periodontal status compared to a similar population of Spanish subjects: a case control study | Tafaj G. and Iniesta M. and Sanz M. and Herrera D. | 4 |
| The Association of Periodontopathic Bacteria Levels in Saliva and Tongue Coating with Oral Malodor in Periodontitis Patients | Takeuchi H. and Machigashira M. and Takeuchi N. and Nakamura T. and Noguchi K. | 4 |
| The relationship between turbidity of mouth-rinsed water and oral health status | Takeuchi S. and Ueno M. and Takehara S. and Pham T.A. and Hakuta C. and Morishima S. and Shinada K. and Kawaguchi Y. | 4 |
| Study of the oral microbial flora in patients with renal disease | Takeuchi Y. and Ishikawa H. and Inada M. and Shinozuka O. and Umeda M. and Yamazaki T. | 4 |
| Amoxicillin plus metronidazole therapy for patients with periodontitis and type 2 diabetes - a 2-year randomized controlled trial. | Tamashiro, N. S. and Duarte, P. M. and Miranda, T. S. and Maciel, S. S. and Figueiredo, L. C. and Faveri, M. and Feres, M. | 4 |
| The detection of Porphyromonas gingivalis, Prevotella intermedia, and Actinobacillus actinomycetemcomitans in the supragingival plaque of children with and without caries | Tanaka S. and Murakami Y. and Seto K. and Takamori K. and Yosida M. and Ochiai K. and Watanabe S. and Fujisawa S. | 4 |
| Effect of a social cognitive intervention on oral health status, behavior reports, and cognitions | Tedesco L.A. and Keffer M.A. and Davis E.L. and Christersson L.A. | 4 |
| Prevalence of Clinical Periodontitis and Putative Periodontal Pathogens among South Indian Pregnant Women. | Tellapragada, Chaitanya and Eshwara, Vandana Kalwaje and Acharya, Shashidhar and Bhat, Parvati and Kamath, Asha and Vishwanath, Shashidhar and Mukhopadhyay, Chiranjay | 4 |
| Patients with obstructive sleep apnea can favor the predisposing factors of periodontitis by the presence of <ovid:i>P. melaninogenica</ovid:i> and <ovid:i>C. albicans</ovid:i>, increasing the severity of the periodontal disease | Tellez-Corral, Mayra A. and Herrera-Daza, Eddy and Cuervo-Jimenez, Hayde K. and Arango-Jimenez, Natalia and Morales-Vera, Darena Z. and Velosa-Porras, Juliana and Latorre-Uriza, Catalina and Escobar-Arregoces, Francina M. and Hidalgo-Martinez, Patricia and Cortes, Maria E. and Roa-Molina, Nelly S. and Otero, Liliana and Parra-Giraldo, Claudia M. | 4 |
| Prevalence of six periodontal pathogens detected by DNA probe method in HIV vs non-HIV periodontitis | Tenenbaum, H. and Elkaim, R. and Cuisinier, F. and Dahan, M. and Zamanian, P. and Lang, J. M. | 4 |
| Deteriorated clinical outcome in coronary artery disease patients with a high prevalence of Porphyromonas gingivalis infection | Tezuka D. and Suzuki J.-I. and Kosuge H. and Aoyama N. and Izumi Y. and Yoshikawa S. and Maejima Y. and Ashikaga T. and Hirao K. and Isobe M. | 4 |
| Rheumatoid factor from periodontitis patients cross-reacts with epitopes on oral bacteria | The J. and Ebersole J.L. | 4 |
| PCR-dipstick DNA chromatography for profiling of a subgroup of caries-associated bacterial species in plaque from healthy coronal surfaces and periodontal pockets. | Tian, Lingyang and Sato, Takuichi and Niwa, Kousuke and Kawase, Mitsuo and Mayanagi, Gen and Washio, Jumpei and Takahashi, Nobuhiro | 4 |
| Clinical periodontal status in pregnant women with rheumatic valvar disease | Timerman L. and Romito G.A. and Marcelino S.L. and Pannuti C.M. and Conrado V.C. and Andrade A.C.P. and Angelis G.A.D. and Neves I.L. and Avila W.S. | 4 |
| Herpesvirus in localized juvenile periodontitis | Ting, M. and Contreras, A. and Slots, J. | 4 |
| Oral and dental complications in dieting disorders | Touyz S.W. and Liew V.P. and Tseng P. and Frisken K. and Williams H. and Beumont P.J.V. | 4 |
| Effects of doxycycline on clinical, microbiological and immunological parameters in well-controlled diabetes type-2 patients with periodontal disease: a randomized, controlled clinical trial. | Tsalikis, L. and Sakellari, D. and Dagalis, P. and Boura, P. and Konstantinidis, A. | 4 |
| Predominant cultivable subgingival microbiota of healthy and HIV-infected ethnic Chinese | Tsang C.S.P. and Samaranayake L.P. | 4 |
| Oral health and biochemical risk factors for bisphosphonateassociated jaw osteonecrosis | Tsao C. and Borromeo G. and Darby I. and Walsh K. and O'Brien-Simpson N. and Reynolds E. and Ebeling P. | 4 |
| High prevalence of Helicobacter pylori detected by PCR in the oral cavities of periodontitis patients | Umeda M. and Kobayashi H. and Takeuchi Y. and Hayashi J. and Morotome-Hayashi Y. and Yano K. and Aoki A. and Ohkusa T. and Ishikawa I. | 4 |
| Periodontal conditions during the pregnancy associated with periodontal pathogens | Usin M.M. and Tabares S.M. and Parodi R.J. and Sembaj A. | 4 |
| Signs of Periodontal Pathogens in Acute Arterial Lower Limb Thrombotic Events | Vakhitov D. and Tuomisto S. and Korhonen J. and Salenius J.-P. and Suominen V. and Pekka K. and Oksala N. | 4 |
| Is the oral microbiome involved in the pathogenesis of sjogren's syndrome? | Van Der Meulen T.A. and Kroese F.G.M. and Liefers S.C. and Vila A.V. and Harmsen H.J.M. and Bootsma H. and Spijkervet F.K.L. and Vissink A. | 4 |
| The role of Solobacterium moorei in oral malodour. | Vancauwenberghe, Frederique and Dadamio, Jesica and Laleman, Isabelle and Van Tornout, Marie and Teughels, Wim and Coucke, Wim and Quirynen, Marc | 4 |
| Microbiological features of Papillon-Lefevre syndrome periodontitis | Velazco C.H. and Coelho C. and Salazar F. and Contreras A. and Slots J. and Pacheco J.J. | 4 |
| Periodontal-disease-associated biofilm: A reservoir for pathogens of medical importance. | Vieira Colombo, Ana Paula and Magalhaes, Clarissa Bichara and Hartenbach, Fatima Aparecida Rocha Resende and Martins do Souto, Renata and Maciel da Silva-Boghossian, Carina | 4 |
| DenTiUS Plaque, a Web-Based Application for the Quantification of Bacterial Plaque: Development and Usability Study. | Vila-Blanco, Nicolas and Freire, Vicente and Balsa-Castro, Carlos and Tomas, Inmaculada and Carreira, Maria J | 4 |
| Effect of metalloporphyrins on red autofluorescence from oral bacteria | Volgenant C.M.C. and van der Veen M.H. and de Soet J.J. and ten Cate J.M. | 4 |
| Roles of oral bacteria in cardiovascular diseases--from molecular mechanisms to clinical cases: Involvement of Porphyromonas gingivalis in the development of human aortic aneurysm. | Wada, Koichiro and Kamisaki, Yoshinori | 4 |
| On the transformation of sulfur-containing amino acids and peptides to volatile sulfur compounds (VSC) in the human mouth | Waler S.M. | 4 |
| Alterations and correlations in dental plaque microbial communities and metabolome characteristics in patients with caries, periodontitis, and comorbid diseases | Wang, Ying and Yang, Fei and Wang, Yuan and Deng, Shuli and Zhu, Rui | 4 |
| Protein citrullination by Porphyromonas gingivalis: A promising piece in the citrulline puzzle | Wegner N. and Eick S. and Sro K.A. and Nguyen K.-A. and Potempa J. and Venables P. | 4 |
| Culture-independent evaluation of bacterial signatures of developing in situ dental biofilms | Werra U. and Rupf S. and Heinzel E. and Kerth N. and Hannig M. and Herrmann M. and Von Muller L. | 4 |
| Clinical and microbiologic study of periodontitis associated with kindler syndrome | Wiebe C.B. and Penagos H. and Luong N. and Slots J. and Epstein Jr. E. and Siegel D. and Hakkinen L. and Putnins E.E. and Larjava H.S. | 4 |
| Application of lemon peel as an unused resource in the oral care field | Winning S. and Iida T. and Fukumoto S. and Sasagawa S. and Araki S. and Inoue T. and Kunishige M. and Kawabata N. and Takamiya N. and Hiramitsu M. and Harada T. | 4 |
| Effects of different immunosuppressive drugs on the periodontal status and changes in periodontal pathogenic bacterial flora in rheumatoid arthritis patients | Wu Z. and Yang X. and Li N. | 4 |
| Precision Medicine for Apical Lesions and Peri-Endo Combined Lesions Based on Transfer Learning Using Periapical Radiographs | Wu, Pei-Yi and Mao, Yi-Cheng and Lin, Yuan-Jin and Li, Xin-Hua and Ku, Li-Tzu and Li, Kuo-Chen and Chen, Chiung-An and Chen, Tsung-Yi and Chen, Shih-Lun and Tu, Wei-Chen and Abu, Patricia Angela R. | 4 |
| Alterations in the Salivary Microbiome and Metabolism in Patients With Carotid Atherosclerosis from Rural Northeast China | Wu, Yahong and Xing, Liying and Lu, Lijie and Liu, Shuang and Zhao, Dan and Lin, Li and Wang, Songlin and Li, Chen and Pan, Yaping | 4 |
| Cigarette Smoking and Opium Use in Relation to the Oral Microbiota in Iran. | Wu, Zeni and Han, Yongli and Caporaso, J Gregory and Bokulich, Nicholas and Mohamadkhani, Ashraf and Moayyedkazemi, Alireza and Hua, Xing and Kamangar, Farin and Wan, Yunhu and Suman, Shalabh and Zhu, Bin and Hutchinson, Amy and Dagnall, Casey and Jones, Kristine and Hicks, Belynda and Shi, Jianxin and Malekzadeh, Reza and Abnet, Christian C and Pourshams, Akram and Vogtmann, Emily | 4 |
| Analysis of matrix metalloproteinase (MMP-8 and MMP-2) activity in gingival crevicular fluid from children with Down's syndrome | Yamazaki-Kubota T. and Miyamoto M. and Sano Y. and Kusumoto M. and Yonezu T. and Sugita K. and Okuda K. and Yakushiji M. and Ishihara K. | 4 |
| Characterizing the Subgingival Microbiome of Pregnant African American Women | Yang I. and Knight A.K. and Dunlop A.L. and Corwin E.J. | 4 |
| Gestational diabetes mellitus increases the detection rate and the number of oral bacteria in pregnant women | Yao H. and Xu D. and Zhu Z. and Wang G. | 4 |
| Oral colonization by Entamoeba gingivalis and Trichomonas tenax: a PCR-based study in health, gingivitis, and periodontitis. | Yaseen, Alaa and Mahafzah, Azmi and Dababseh, Deema and Taim, Duaa and Hamdan, Ahmad A. and Al-Fraihat, Esraa and Hassona, Yazan and Sahin, Gulsen Ozkaya and Santi-Rocca, Julien and Sallam, Malik | 4 |
| The antimicrobial activity of compounds from the leaf and stem of Vitis amurensis against two oral pathogens | Yim N. and Ha D.T. and Trung T.N. and Kim J.P. and Lee S. and Na M. and Jung H. and Kim H.S. and Kim Y.H. and Bae K. | 4 |
| Short-chain fatty acids from periodontal pathogens suppress histone deacetylases, EZH2, and SUV39H1 to promote Kaposi's sarcoma-associated herpesvirus replication | Yu, Xiaolan and Shahir, Abdel-Malek and Sha, Jingfeng and Feng, Zhimin and Eapen, Betty and Nithianantham, Stanley and Das, Biswajit and Karn, Jonathan and Weinberg, Aaron and Bissada, Nabil F. and Ye, Fengchun | 4 |
| Rapid diagnosis of periodontal infections: findings in AIDS patients. | Zambon, J. J. | 4 |
| Frequent detection of Streptococcus tigurinus in the human oral microbial flora by a specific 16S rRNA gene real-time TaqMan PCR | Zbinden A. and Aras F. and Zbinden R. and Mouttet F. and Schmidlin P.R. and Bloemberg G.V. and Bostanci N. | 4 |
| Frequent detection of <ovid:i>Streptococcus tigurinus</ovid:i> in the human oral microbial flora by a specific 16S rRNA gene real-time TaqMan PCR | Zbinden, A. and Aras, F. and Zbinden, R. and Mouttet, F. and Schmidlin, P. R. and Bloemberg, G. V. and Bostanci, N. | 4 |
| A case control study on oral microbiota and local inflammatory biomarkers in Parkinson's disease | Zekeridou A. and Fleury V. and Lazarevic V. and Gaia N. and Giannopoulou C. and Genton L. and Cancela J.A. and Girard M. and Goldstein R. and Bally J. and Mombelli A. and Schrenzel J. and Burkhard P. | 4 |
| Diversity of treponema denticola and other oral treponeme lineages in subjects with periodontitis and gingivitis | Zeng H. and Chan Y. and Gao W. and Leung W.K. and Watt R.M. | 4 |
| Difference in oral microbial composition between chronic periodontitis patients with and without diabetic nephropathy | Zhang D. and Liu W. and Peng L. and Wang H. and Lin M. and Li Y. and Wang Z. | 4 |
| Relationship between microorganisms in coronary atheromatous plaques and periodontal pathogenic bacteria. | Zhang YuanMing, Zhang YuanMing and Zhong LiangJun, Zhong LiangJun and Liang Ping, Liang Ping and Liu Hua, Liu Hua and Mu LaTi, Mu LaTi and Ai SiKaer, Ai SiKaer | 4 |
| Gender Variations in the Oral Microbiomes of Elderly Patients with Initial Periodontitis | Zhao, Jie and Zhou, Ying-Hui and Zhao, Ya-Qiong and Feng, Yao and Yan, Fei and Gao, Zheng-Rong and Ye, Qin and Chen, Yun and Liu, Qiong and Tan, Li and Zhang, Shao-Hui and Hu, Jing and Dusenge, Marie Aimee and Feng, Yun-Zhi and Guo, Yue | 4 |
| Relationship between oral problems and Helicobacter pylori infection. | Zheng YanSong, Zheng YanSong and Liu MinYan, Liu MinYan and Shu Hua, Shu Hua and Chen ZhiLai, Chen ZhiLai and Liu GuiXia, Liu GuiXia and Zhang YeYing, Zhang YeYing | 4 |
| Periodontal and Other Oral Bacteria and Risk of Lung Cancer in the Atherosclerosis Risk in Communities (ARIC) Study | Zhou B. and Lu J. and Beck J.D. and Moss K.L. and Prizment A.E. and Demmer R.T. and Porosnicu Rodriguez K.A. and Joshu C.E. and Michaud D.S. and Platz E.A. | 4 |
| Periodontal Status and Microbiologic Pathogens in Patients with Chronic Obstructive Pulmonary Disease and Periodontitis: A Case-Control Study. | Zhou, Xuan and Wang, Jitian and Liu, Wenyan and Huang, Xuan and Song, Yiqing and Wang, Zuomin and Jia, Xingyuan | 4 |
| Periodontal pathogenic bacteria and aMMP-8 findings depending on periodontal conditions of patients before and after liver transplantation | Ziebolz D. and Schmalz G. and Kauffels A. and Widmer F. and Widmer K. and Slotta J.E. and Mausberg R.F. and Kollmar O. | 4 |
| Gingivitis, plaque accumulation and plaque composition under long-term use of Meridol. | Zimmermann, A and Flores-de-Jacoby, L and Pan, P and Pan, P | 4 |
| Marginal and subgingival plaque--a natural habitat of Tropheryma whipplei?. | Zinkernagel, A S and Gmur, R and Fenner, L and Schaffner, A and Schoedon, G and Schneemann, M | 4 |
| Marginal and subgingival plaque - a natural habitat of <ovid:i>Tropheryma whipplei</ovid:i>? | Zinkernagel, A. S. and Gmur, R. and Fenner, L. and Schaffner, A. and Schoedon, G. and Schneemann, M. | 4 |
| Staphylococcus aureus and other bacteria in untreated periodontitis | Zinsli Fritschi B. and Albert-Kiszely A. and Persson G.R. | 4 |
| Prevalence of potential bacterial respiratory pathogens in the oral cavity of hospitalised individuals | Zuanazzi D. and Souto R. and Mattos M.B.A. and Zuanazzi M.R. and Tura B.R. and Sansone C. and Colombo A.P.V. | 4 |
| Adult atopic dermatitis is associated with impaired oral health and oral dysbiosis - a case-control study | | 4 |
| Microbiologic tests in epidemiologic studies: are they reproducible? | Aass A.M. and Preus H.R. and Zambon J.J. and Gjermo P. | 5 |
| Crevicular fluid level of beta-glucuronidase in relation to clinical periodontal parameters and putative periodontal pathogens in early-onset periodontitis. | Albandar, J M and Kingman, A and Lamster, I B | 5 |
| Pleckstrin Levels Are Increased in Patients with Chronic Periodontitis and Regulated <ovid:i>via</ovid:i> the MAP Kinase-p38alpha Signaling Pathway in Gingival Fibroblasts | Alim, M. Abdul and Njenda, Duncan and Lundmark, Anna and Kaminska, Marta and Jansson, Leif and Eriksson, Kaja and Kats, Anna and Johannsen, Gunnar and Arvidsson, Catalin Koro and Mydel, Piotr M. and Yucel-Lindberg, Tulay | 5 |
| Distribution of Porphyromonas gingivalis strains with fimA genotypes in periodontitis patients | Amano A. and Nakagawa I. and Kataoka K. and Morisaki I. and Hamada S. | 5 |
| Salvadora Persica extract chewing gum and gingival health: Improvement of gingival and probe-bleeding index | Amoian B. and Moghadamnia A.A. and Barzi S. and Sheykholeslami S. and Rangiani A. | 5 |
| The Association between Salivary Metabolites and Gingival Bleeding Score in Healthy Subjects: A Pilot Study | Antonelli, R. and Ferrari, E. and Gallo, M. and Ciociola, T. and Calciolari, E. and Spisni, A. and Meleti, M. and Pertinhez, T. A. | 5 |
| Molecular survey of atheromatous plaques for the presence of DNA from periodontal bacterial pathogens, archaea and fungi | Aquino, A. R. L. and Lima, K. C. and Paiva, M. S. and Rocas, I. N. and Siqueira, J. F., Jr. | 5 |
| Comparative 16S rRNA gene sequencing study of subgingival microbiota of healthy subjects and patients with periodontitis from four different countries. | Arredondo, A and Alvarez, G and Isabal, S and Teughels, W and Laleman, I and Contreras, M J and Isbej, L and Huapaya, E and Mendoza, G and Mor, C and Nart, J and Blanc, V and Leon, R | 5 |
| The long-term effect of a plaque control program on tooth mortality, caries and periodontal disease in adults: results after 30 years of maintenance. | Axelsson, P. and Nystrom, B. and Lindhe, J. | 5 |
| Laboratory studies of a family manifesting premature exfoliation of deciduous teeth. | Baab, D A and Page, R C and Ebersole, J L and Williams, B L and Scott, C R | 5 |
| ACE2 gene expression and inflammatory conditions in periodontal microenvironment of COVID-19 patients with and without diabetes evaluated by qPCR | Bachtiar B.M. and Bachtiar E.W. and Sunarto H. and Soeroso Y. and Sulijaya B. and Theodorea C.F. and Pratomo I.P. and Yudhistira and Kusumaningrum A. and Efendi D. and Apriyanti E. and Utami N.R. and Deviana A. and Andriyani A.D. AO - Bachtiar, Boy M.; ORCID: https://orcid.org/0000-0002-4027-778X AO -... | 5 |
| Atomic force spectroscopy evidence of non-specific adhesion of Aggregatibacter actinomycetemcomitans | Bank T.L. and Dosen A. and Giese R.F. and Haase E.M. and Sojar H.T. | 5 |
| COMPARISON OF PERIODONTAL PATHOGEN BIOFILM FORMATION ON TWO D-PTFE MEMBRANES | Begic G. and Gobin I. and Badovinac I.J. and Peloza O.C. and Prpic J. and Kuis D. | 5 |
| Microbial profile comparisons of saliva, pooled and site-specific subgingival samples in periodontitis patients. | Belstrom, D. and Sembler-Moller, M. L. and Grande, M. A. and Kirkby, N. and Cotton, S. L. and Paster, B. J. and Holmstrup, P. | 5 |
| The effect of supragingival plaque control on the composition of the subgingival microflora in human periodontitis. | Beltrami, M and Bickel, M and Baehni, P C | 5 |
| Inhibition of bacterial growth by tetracycline-impregnated enamel and dentin. | Bjorvatn, K and Skaug, N and Selvig, K A | 5 |
| Locally delivered doxycycline during supportive periodontal therapy: a 3-year study. | Bogren, Anna and Teles, Ricardo P and Torresyap, Gay and Haffajee, Anne D and Socransky, Sigmund S and Wennstrom, Jan L | 5 |
| Periodontal pathogens: a quantitative comparison of anaerobic culture and real-time PCR. | Boutaga, Khalil and van Winkelhoff, Arie Jan and Vandenbroucke-Grauls, Christina M J E and Savelkoul, Paul H M | 5 |
| Tissue localization of Actinobacillus actinomycetemcomitans in human periodontitis. II. Correlation between immunofluorescence and culture techniques | Christersson L.A. and Wikesjo U.M. and Albini B. and Zambon J.J. and Genco R.J. | 5 |
| Assessment of five culture media for the growth and isolation of Capnocytophaga spp. | Ciantar, M. and Spratt, D. A. and Newman, H. N. and Wilson, M. | 5 |
| Non-radioactively labelled DNA probes for the detection of periodontopathogenic Prevotella and Porphyromonas species | Conrads G. and Brauner A. | 5 |
| Simultaneous detection of Bacteroides forsythus and Prevotella intermedia by 16S rRNA gene-directed multiplex PCR. | Conrads, G. and Flemmig, T. F. and Seyfarth, I. and Lampert, F. and Lutticken, R. | 5 |
| Dipeptidyl peptidase II- and IV-like activities in gingival tissue and crevicular fluid from human periodontitis lesions | Cox S.W. and Gazi M.I. and Eley B.M. | 5 |
| A. actinomycetemcomitans profile and red complex bacterial species of an Afro-Brazilian community: A comparative study | de Araujo Neris M. and Cortelli S.C. and Aquino D.R. and de Miranda T.B. and Costa Fd.e O. and Cortelli J.R. | 5 |
| Microbiology of subgingival plaque from children with localized prepubertal periodontitis | Delaney J.E. and Kornman K.S. | 5 |
| High-Throughput Combined Analysis of Saliva Microbiota and Metabolomic Profile in Chinese Periodontitis Patients: A Pilot Study | Ding, J. and Li, J. and Zhang, C. and Tan, L. and Zhao, C. and Gao, L. | 5 |
| Enumeration of Porphyromonas gingivalis, Prevotella intermedia and Actinobacillus actinomycetemcomitans in subgingival plaque samples by a quantitative-competitive PCR method. | Doungudomdacha, S and Rawlinson, A and Douglas, C W | 5 |
| Molecular epidemiology and spatial distribution of Selenomonas spp. in subgingival biofilms. | Drescher, J. and Schlafer, S. and Schaudinn, C. and Riep, B. and Neumann, K. and Friedmann, A. and Petrich, A. and Gobel, U. B. and Moter, A. | 5 |
| Periodontal and microbiological data in patients with mucous membrane pemphigoid in a French population in 2021-2022: A pilot cross-sectional study | Ejeil, A. L. and Gaultier, F. and Catherine, B. and Chaubron, F. and Lupi, L. and Dridi, S. M. | 5 |
| Detection of selected bacterial species in intraoral sites of patients with chronic periodontitis using multiplex polymerase chain reaction | Estrela C.R.A. and Pimenta F.C. and de Alencar A.H.G. and Ruiz L.F.N. and Estrela C. | 5 |
| Microbiological effects of amoxicillin plus metronidazole in the treatment of young patients with Stages III and IV periodontitis: a secondary analysis from a 1-year double-blinded placebo-controlled randomized clinical trial. | Faveri, Marcelo and Retamal-Valdes, Belen and Mestnik, Maria Josefa and de Figueiredo, Luciene Cristina and Barao, Valentim Adelino Ricardo and Souza, Joao Gabriel Silva and Duarte, Poliana Mendes and Feres, Magda | 5 |
| [Longitudinal study in 8 human subjects having various gingival inflammation indices]. | Fourel, J | 5 |
| Comparison of laboratory methods for detecting beta -lactamase-positive strains in the species Prevotella intermedia sensu lato isolated from periodontal pockets. | Gatignol, J. P. and Poulet, P. P. and Desse, T. and Duffaut, D. | 5 |
| Effects of mouth rinses with xylitol and fluoride on dental plaque and saliva. | Giertsen, E and Emberland, H and Scheie, A A | 5 |
| The Campylobacter rectus lipopolysaccharide core is essential for maximum prostaglandin E2 elicitation in mouse macrophages | Gillespie M.J. | 5 |
| A combined immunofluorescence and fluorescent in situ hybridization assay for single cell analyses of dental plaque microorganisms. | Gmur, Rudolf and Luthi-Schaller, Helga | 5 |
| Whole mouth microbiota effects following subgingival delivery of sanguinarium | Godowski, K. C. and Wolff, E. D. and Thompson, D. M. and Housley, C. J. and Polson, A. M. and Dunn, R. L. and Duke, S. P. and Stoller, N. H. and Southard, G. L. | 5 |
| Enumeration of subgingival species on primary isolation plates using colony lifts. | Gunaratnam, M and Smith, G L and Socransky, S S and Smith, C M and Haffajee, A D | 5 |
| Changes in subgingival microbiota during puberty. A 4-year longitudinal study. | Gusberti, F A and Mombelli, A and Lang, N P and Minder, C E | 5 |
| Factors affecting human supragingival biofilm composition. II. Tooth position | Haffajee A.D. and Teles R.P. and Patel M.R. and Song X. and Yaskell T. and Socransky S.S. | 5 |
| Comparison between polymerase chain reaction-based and checkerboard DNA hybridization techniques for microbial assessment of subgingival plaque samples. | Haffajee, Anne D and Yaskell, Tina and Torresyap, Gay and Teles, Ricardo and Socransky, Sigmund S | 5 |
| Simple and rapid detection of porphyromonas gingivalis and aggregatibacter actinomycetemcomitans by loop-mediated isothermal amplification assay | Hamzan N.I. and Fauzi F.H. and Taib H. and Mohamad S. | 5 |
| Saliva Diagnosis Using Small Extracellular Vesicles and Salivaomics | Han P. and Li X. and Wei W. and Ivanovski S. | 5 |
| Community-level assessment of dental plaque bacteria susceptibility to triclosan over 19 years. | Haraszthy, Violet I and Sreenivasan, Prem K and Zambon, Joseph J | 5 |
| Risk of aggressive periodontitis in adolescent carriers of the JP2 clone of <ovid:i>Aggregatibacter</ovid:i> (<ovid:i>Actinobacillus</ovid:i>) <ovid:i>actinomycetemcomitans</ovid:i> in Morocco: a prospective longitudinal cohort study | Haubek, D. and Ennibi, O. K. and Poulsen, K. and Vaeth, M. and Poulsen, S. and Kilian, M. | 5 |
| Subgingival microbial profiles in chronic periodontitis patients from Chile, Colombia and Spain. | Herrera, D. and Contreras, A. and Gamonal, J. and Oteo, A. and Jaramillo, A. and Silva, N. and Sanz, M. and Botero, J. E. and Leon, R. | 5 |
| Characteristics and sites of infection of Eubacterium nodatum, Eubacterium timidum, Eubacterium brachy, and other asaccharolytic eubacteria | Hill G.B. and Ayers O.M. and Kohan A.P. | 5 |
| Metronidazole Potentiation by Panax Ginseng and <ovid:i>Symphytum officinale</ovid:i>: A New Strategy for <ovid:i>P. gingivalis</ovid:i> Infection Control | Ibrahim, Salah M. and Al-Mizraqchi, Abbas S. and Haider, Julfikar | 5 |
| Detection of Campylobacter rectus in periodontitis sites by monoclonal antibodies | Ihara H. and Miura T. and Kato T. and Ishihara K. and Nakagawa T. and Yamada S. and Okuda K. | 5 |
| Evaluation of TLR2 and 4 in chronic periodontitis | Ilango P. and Mahalingam A. and Parthasarathy H. and Reddy V.K. and Reddy V.S. | 5 |
| Determining the Relationship of Bacteria Associated with Dental Disease and Infective Endocarditis: A Cross-sectional Study | Indermun, S. and Baswaraj, D. and Regunath, H. and Kumar, S. | 5 |
| A sensitive enzymatic method (SK-013) for detection and quantification of specific periodontopathogens | Ishihara K. and Naito Y. and Kato T. and Takazoe I. and Okuda K. and Eguchi T. and Nakashima K. and Matsuda N. and Yamasaki K. and Hasegawa K. | 5 |
| Polymerase chain reaction in the identification of periodontopathogens - a reliable and satisfactory method? | Jakoba, N. N. and Vojnovic, S. and Pavic, A. and Jankovic, S. and Lekovic, V. and Vasiljevic, B. | 5 |
| Autoinducer 2 of Fusobacterium nucleatum as a target molecule to inhibit biofilm formation of periodontopathogens | Jang Y.-J. and Choi Y.-J. and Lee S.-H. and Jun H.-K. and Choi B.-K. | 5 |
| Effect of preparation method and storage period on the stability of saliva DNA | Karched M. and Bhardwaj R.G. and Pauline E.M. and George S. and Asikainen S. | 5 |
| Proportional distribution of the red complex and its individual pathogens after sample storage using the checkerboard DNA-DNA hybridization technique. | Katsoulis, J. and Lang, N. P. and Persson, G. R. | 5 |
| Saliva/pathogen biomarker signatures and periodontal disease progression | Kinney J.S. and Morelli T. and Braun T. and Ramseier C.A. and Herr A.E. and Sugai J.V. and Shelburne C.E. and Rayburn L.A. and Singh A.K. and Giannobile W.V. | 5 |
| Structure of the fimbrial protein Mfa4 from Porphyromonas gingivalis in its precursor form: implications for a donor-strand complementation mechanism. | Kloppsteck, Patrik and Hall, Michael and Hasegawa, Yoshiaki and Persson, Karina | 5 |
| Clinical and microbiological effects of a sanguinaria-containing mouthrinse and dentifrice with and without fluoride during 6 months of use. | Kopczyk, R A and Abrams, H and Brown, A T and Matheny, J L and Kaplan, A L | 5 |
| Detection of bacterial DNA in atheromatous plaques by quantitative PCR | Kozarov E. and Sweier D. and Shelburne C. and Progulske-Fox A. and Lopatin D. | 5 |
| Changes in periodontal health status are associated with bacterial community shifts as assessed by quantitative 16S cloning and sequencing. | Kumar, Purnima S and Leys, Eugene J and Bryk, Jennifer M and Martinez, Francisco J and Moeschberger, Melvin L and Griffen, Ann L | 5 |
| Differences in the subgingival microbiome according to stage of periodontitis: A comparison of two geographic regions | Lafaurie G.I. and Neuta Y. and Rios R. and Pacheco-Montealegre M. and Pianeta R. and Castillo D.M. and Herrera D. and Reyes J. and Diaz L. and Castillo Y. and Sanz M. and Iniesta M. | 5 |
| Actinobacillus actinomycetemcomitans proportion of subgingival bacterial flora in relation to its clonal type | Lakio L. and Kuula H. and Dogan B. and Asikainen S. | 5 |
| Quantitative real-time polymerase chain reaction versus culture: a comparison between two methods for the detection and quantification of Actinobacillus actinomycetemcomitans, Porphyromonas gingivalis and Tannerella forsythensis in subgingival plaque samples. | Lau, L. and Sanz, M. and Herrera, D. and Morillo, J. M. and Martin, C. and Silva, A. | 5 |
| Molecular analysis of the subgingival microbiota in health and disease. | Ledder, R. G. and Gilbert, P. and Huws, S. A. and Aarons, L. and Ashley, M. P. and Hull, P. S. and McBain, A. J. | 5 |
| Identification of Porphyromonas gingivalis strains by heteroduplex analysis and detection of multiple strains | Leys E.J. and Smith J.H. and Lyons S.R. and Griffen A.L. | 5 |
| Rapid and specific detection of the leukotoxin sequences of Actinobacillus actinomycetemcomitans from periodontal pockets by the polymerase chain reaction | Lin C.Y. and Wong M.Y. and Jeng J.H. and Chang W.K. and Kuo M.Y. | 5 |
| Differential dark field microscopy of subgingival bacteria as an aid in selecting recall intervals: Results after 18 months | Listgarten M.A. and Schifter C. | 5 |
| Failure of a microbial assay to reliably predict disease recurrence in a treated periodontitis population receiving regularly scheduled prophylaxes | Listgarten M.A. and Schifter C.C. and Sullivan P. and George C. and Rosenberg E.S. | 5 |
| 3-year longitudinal study of the periodontal status of an adult population with gingivitis. | Listgarten, M A and Schifter, C C and Laster, L | 5 |
| Comparative longitudinal study of 2 methods of scheduling maintenance visits: 4-year data. | Listgarten, M A and Sullivan, P and George, C and Nitkin, L and Rosenberg, E S and Chilton, N W and Kramer, A A | 5 |
| Periodontal health and disease in young people: screening for priority care | Loe H. and Morrison E. | 5 |
| Comparison of various detection methods for periodontopathic bacteria: Can culture be considered the primary reference standard? | Loesche W.J. and Lopatin D.E. and Stoll J. and Van Poperin N. and Hujoel P.P. | 5 |
| Probiotics do not alter the long-term stability of the supragingival microbiota in healthy subjects: a randomized controlled trial. | Lundtorp-Olsen, Christine and Enevold, Christian and Twetman, Svante and Belstrom, Daniel | 5 |
| Lipoxin A4 levels predict site-specific clinical improvements post scaling and root planing and correlate negatively with periodontal pathogens in severe periodontitis | Ma, Rui and Liu, Yi-ying and Xu, Yi and Duan, Ding-yu | 5 |
| Alternative methods for screening periodontal disease in adults. | Machtei, E E and Christersson, L A and Zambon, J J and Hausmann, E and Grossi, S G and Dunford, R and Genco, R J | 5 |
| Detection of periodontal pathogen Porphyromonas gingivalis by loop-mediated isothermal amplification method | Maeda H. and Kokeguchi S. and Fujimoto C. and Tanimoto I. and Yoshizumi W. and Nishimura F. and Takashiba S. | 5 |
| Supragingival cleaning 3 times a week. The microbiological effects in moderately deep pockets. | McNabb, H and Mombelli, A and Lang, N P | 5 |
| Novel strategy to detect and locate periodontal pathogens: The PNA-FISH technique. | Mendes, Luzia and Rocha, Rui and Azevedo, Andreia Sofia and Ferreira, Catarina and Henriques, Mariana and Pinto, Miguel Goncalves and Azevedo, Nuno Filipe | 5 |
| Prevalence of Helicobacter pylorivacA genotypes and cagA gene in dental plaque of asymptomatic Mexican children. | Mendoza-Cantu, A. and Urrutia-Baca, V. H. and Urbina-Rios, C. S. and Garza-Ramos, M. A. de la and Garcia-Martinez, M. E. and Torre-Martinez, H. H. H. | 5 |
| Rapid and simple detection of eight major periodontal pathogens by the loop-mediated isothermal amplification method | Miyagawa J. and Maeda H. and Murauchi T. and Kokeguchi S. and Yamabe K. and Tanimoto I. and Nishimura F. and Fukui K. and Takashiba S. | 5 |
| Microbial changes associated with the development of puberty gingivitis. | Mombelli, A and Lang, N P and Burgin, W B and Gusberti, F A | 5 |
| Use of synthetic oligonucleotide DNA probes for identification and direct detection of Bacteroides forsythus in plaque samples | Moncla B.J. and Motley S.T. and Braham P. and Ewing L. and Adams T.H. and Vermeulen N.M.J. | 5 |
| Porphyromonas gingivalis Fim-A genotype distribution among Colombians. | Moreno, Sandra and Jaramillo, Adriana and Parra, Beatriz and Botero, Javier Enrique and Contreras, Adolfo | 5 |
| Haemophilus pittmaniae and Leptotrichia spp. constitute a multi-marker signature in a cohort of human papillomavirus-positive head and neck cancer patients. | Mougeot, Jean-Luc C. and Beckman, Micaela F. and Langdon, Holden C. and Lalla, Rajesh V. and Brennan, Michael T. and Mougeot, Farah K. Bahrani | 5 |
| Effect of gingival fluid collection on subgingival plaque sampling | Mullally B. and Wolff L. and Hardie N. and Aeppli D. and Pihlstrom B. | 5 |
| Biologic modelling of periodontal disease progression | Nagarajan R. and Miller C.S. and Dawson D. and Ebersole J.L. AO - Ebersole, Jeffrey L.; ORCID: https://orcid.org/0000-0002-9743-6585 | 5 |
| Distribution of 10 periodontal bacterial species in children and adolescents over a 7-year period. | Nakano, K. and Miyamoto, E. and Tamura, K. and Nemoto, H. and Fujita, K. and Nomura, R. and Ooshima, T. | 5 |
| Comparison of Two Different Sampling Methods for Subgingival Plaque: Subgingival Paper Points or Mouthrinse Sample? | Nickles K. and Scharf S. and Rollke L. and Dannewitz B. and Eickholz P. | 5 |
| Detection of subgingival periodontal pathogens--comparison of two sampling strategies. | Nickles, Katrin and Scharf, Susanne and Rollke, Lasse and Mayer, Irina and Mayer, Matthias and Eickholz, Peter | 5 |
| Association between involuntary smoking and salivary markers related to periodontitis: a 2-year longitudinal study. | Nishida, N. and Yamamoto, Y. and Tanaka, M. and Kataoka, K. and Kuboniwa, M. and Nakayama, K. and Morimoto, K. and Shizukuishi, S. | 5 |
| New clinical diagnostic strategies based on pathogenesis of disease. | Offenbacher, S and Collins, J G and Arnold, R R | 5 |
| Relationship between saliva and sublingual immunotherapy | Oka A. and Okano M. | 5 |
| Adherence to experimental pellicle of rough-type lipopolysaccharides from subgingival plaque bacteria | Okuda K. and Kato T. and Ishihara K. and Naito Y. | 5 |
| Occurrence of selected bacteria in periodontal pockets of various depths in chronic and aggressive periodontitis | Orzechowska A. and Grabowska E. and Plakwicz P. and Gorska R. | 5 |
| Rapid detection of Actinobacillus actinomycetemcomitans using a loop-mediated isothermal amplification method. | Osawa, R and Yoshida, A and Masakiyo, Y and Nagashima, S and Ansai, T and Watari, H and Notomi, T and Takehara, T | 5 |
| Laboratory and clinical comparison of preservation media and transport conditions for survival of Actinobacillus actinomycetemcomitans | Piccolomini R. and Catamo G. and Di Bonaventura G. and Picciani C. and Paolantonio M. | 5 |
| Improved PCR for detection of the highly leukotoxic JP2 clone of Actinobacillus actinomycetemcomitans in subgingival plaque samples. | Poulsen, K. and Ennibi, O. K. and Haubek, D. | 5 |
| Analysis of neutrophil-derived antimicrobial peptides in gingival crevicular fluid suggests importance of cathelicidin LL-37 in the innate immune response against periodontogenic bacteria | Puklo M. and Guentsch A. and Hiemstra P.S. and Eick S. and Potempa J. | 5 |
| Phenotypic identification of periodontal Prevotella intermedia/nigrescens group isolates validated by MALDI-TOF mass spectrometry. | Rams, Thomas E and Sautter, Jacqueline D and Hsiao, Chinhua Y and van Winkelhoff, Arie J | 5 |
| Development of a PCR assay, specific for Peptostreptococcus anaerobius | Riggio M.P. and Lennon A. | 5 |
| Dental biofilms at healthy and inflamed gingival margins | Rudiger S.G. and Carlen A. and Meurman J.H. and Kari K. and Olsson J. | 5 |
| Endpoint quantitative PCR assays for Bacteroides forsythus, Porphyromonas gingivalis, and Actinobacillus actinomycetemcomitans. | Rudney, J. D. and Chen, R. and Pan, Y. | 5 |
| Evaluation of three selective media for isolation of Aggregatibacter actinomycetemcomitans | Rurenga P. and Raangs E. and Singadji Z. and Wekema-Mulder G. and Veloo A.C.M. and Van Winkelhoff A.J. | 5 |
| Structure of sub- and supragingival dental calculus in human periodontitis. An electron microscopic study | Ruzicka F. | 5 |
| Prevalence of subgingival staphylococcus at periodontally healthy and diseased sites. | Santos, B. R. M. dos and Demeda, C. F. and Silva, E. E. N. F. da and Britto, M. H. M. F. de and Lima, K. C. and Melo, M. C. N. de | 5 |
| Comparison of cultural methods and DNA probe analyses for the detection of Actinobacillus actinomycetemcomitans, Bacteroides gingivalis, and Bacteroides intermedius in subgingival plaque samples | Savitt E.D. and Strzempko M.N. and Vaccaro K.K. and Peros W.J. and French C.K. | 5 |
| Observation of fimbriae and flagella in dispersed subgingival dental plaque and fresh bacterial isolates from periodontal disease | Scannapieco F.A. and Kornman K.S. and Coykendall A.L. | 5 |
| The occurrence of Actinobacillus actinomycetemcomitans, Bacteroides gingivalis and Bacteroides intermedius in destructive periodontal disease in adults | Slots J. and Bragd L. and Wikstrom M. and Dahlen G. | 5 |
| Assessment of periodontal disease activity by gingival crevicular fluid assay. | Smith, Q T | 5 |
| Identification of the microorganisms responsible for periodontopathy by Multiplex RT-PCR | Squeri R. and La Fauci V. and Cannavo G. and Lo Giudice G. and Sindoni L. | 5 |
| Association of periodontitis with increased colonization by Prevotella nigrescens | Stingu C.S. and Schaumann R. and Jentsch H. and Eschrich K. and Brosteanu O. and Rodloff A.C. | 5 |
| Human cytomegalovirus and Epstein-Barr virus in apical and marginal periodontitis: a role in pathology?. | Sunde, Pia Titterud and Olsen, Ingar and Enersen, Morten and Beiske, Klaus and Grinde, Bjorn | 5 |
| Lysozyme and microbiota in relation to gingivitis and periodontitis. | Surna, Algimantas and Kubilius, Ricardas and Sakalauskiene, Jurgina and Vitkauskiene, Astra and Jonaitis, Juozas and Saferis, Viktoras and Gleiznys, Alvydas | 5 |
| Quantification of periodontopathic bacteria in saliva using the invader assay. | Tada, Akio and Takeuchi, Hiroaki and Shimizu, Hajime and Tadokoro, Kenichi and Tanaka, Kazuya and Kawamura, Katsumi and Yamaguchi, Toshikazu and Egashira, Toru and Nomura, Yoshiaki and Hanada, Nobuhiro | 5 |
| New rapid polymerase chain reaction-immunochromatographic assay for Porphyromonas gingivalis. | Takada, Kazuko and Sakaguchi, Yoshiaki and Oka, Chitoshi and Hirasawa, Masatomo | 5 |
| Evaluation of oral microbiota in undernourished and eutrophic children using checkerboard DNA-DNA hybridization | Testa M. and Erbiti S. and Delgado A. and Cardenas I.L. AO - Testa M.; ORCID: https://orcid.org/0000-0001-8945-3118 | 5 |
| A rapid DNA probe test compared to culture methods for identification of subgingival plaque bacteria | Tsai C.Y. and Wolff L.F. and Germaine G. and Hodges J. | 5 |
| Improved detection of oral spirochetes with an anaerobic culture method | Umeda M. and Ishikawa I. and Benno Y. and Mitsuoka T. | 5 |
| Selling biotechnology in the dental medicine marketplace: The OmniGene Diagnostics DNA probe tests for periodontal pathogens | Van Arsdell S.W. and DiFronzo F. and Backman K.C. and Mahler P.H. | 5 |
| Changes in oral microflora after full-mouth tooth extraction: a prospective cohort study. | Waal, Y. C. M. de and Winkel, E. G. and Raangs, G. C. and Vusse, M. L. van der and Rossen, J. W. A. and Winkelhoff, A. J. van | 5 |
| Porphyromonas gingivalis, Actinobacillus actinomycetemcomitans and Treponema denticola detection in oral plaque samples using the polymerase chain reaction | Watanabe K. and Frommel T.O. | 5 |
| Effects of subgingival irrigation on A. actinomycetemcomitans | Wikesjo U.M. and Reynolds H.S. and Christersson L.A. and Zambon J.J. and Genco R.J. | 5 |
| Detection rates of presumptive periodontal pathogens in subgingival plaque samples of untreated periodontitis using either four or six pooled samples. | Wohlfeil, Martin and Tabakci, Orhan and Arndt, Rita and Eickholz, Peter and Nickles, Katrin | 5 |
| The distribution of Porphyromonas gingivalis and Treponema denticola in different depth of periondontal pockets. | Wu ChunLan, Wu ChunLan and Jiang JianQun, Jiang JianQun and Lei JianQiang, Lei JianQiang | 5 |
| Comparison of the microbiota of supra- and subgingival plaque in health and periodontitis. | Ximenez-Fyvie, L A and Haffajee, A D and Socransky, S S | 5 |
| Comparative analysis of oral treponemes associated with periodontal health and disease | You M. and Mo S. and Leung W.K. and Watt R.M. | 5 |
| Oral treponeme major surface protein: sequence diversity and distributions within periodontal niches. | You, M. and Chan, Y. and Lacap-Bugler, D. C. and Huo, Y. B. and Gao, W. and Leung, W. K. and Watt, R. M. | 5 |
| Detection rate of Actinobacillus actinomycetemcomitans on the permanent 1st molars of primary school children in Taiwan by polymerase chain reaction. | Yuan Kuo, Yuan Kuo and Hsu PingChi, Hsu PingChi and Tseng ChuenChyi, Tseng ChuenChyi and Kiang, D. and Wang JenRen, Wang JenRen | 5 |
| Effectiveness of Diode (810 nm) Laser in Periodontal Parameters and Reduction of Subgingival Bacterial Load in Periodontitis Patients | Abdullah L.A. and Hashim N. and Rehman M.M. and Elhaj M.H. and Mukhtar M.M. and Gismalla B.G. | 6 |
| Correlation between the immunohistochemical expression of basic fibroblast growth factor in gingival tissue with the clinical periodontal parameters (plaque index, gingival index, bleeding on probing) and angiogenesis | Abed S.A. and Ali B.G. and Sarkis S.A. and AL-sayyid M.M. | 6 |
| Impact of photodynamic therapy versus ultrasonic scaler on gingival health during treatment with orthodontic fixed appliances. | Abellan, Rosa and Gomez, Clara and Iglesias-Linares, Alejandro and Palma, Juan Carlos | 6 |
| Effects of scaling and root planing and 0.2% chlorhexidine rinse on clinical and microbiological parameters in generalised chronic periodontitis - a clinico-microbiological study. | Abhima Kumar, Abhima Kumar and Khan, R. N. and Jan, S. M. and Roobal Behal, Roobal Behal | 6 |
| Frequency of Porphyromonas gingivalis fimA in smokers and nonsmokers after periodontal therapy | Abreu M.G.L. and Kawamoto D. and Mayer M.P.A. and Pascoal V.D.B. and Caiaffa K.S. and Zuza E.P. and Duque C. and Camargo G.A.D.C.G. AO - Camargo, Gabriela Alessandra da Cruz Galhardo; ORCID: https://orcid.... | 6 |
| Effect of three different motivational techniques on oral hygiene and gingival health of patients undergoing multibracketed orthodontics. | Acharya, Shivesh and Goyal, Ashima and Utreja, Ashok Kumar and Mohanty, Utkal | 6 |
| Comparison of Microbiome in Stimulated Saliva in Edentulous and Dentate Subjects | Adami, G. R. and Ang, M. J. and Kim, E. M. | 6 |
| Full-mouth disinfection effects on gingival fluid calprotectin, osteocalcin, and N-telopeptide of Type I collagen in severe periodontitis | Afacan B. and Cinarcik S. and Gurkan A. and Ozdemir G. and Ilhan H.A. and Vural C. and Kose T. and Emingil G. | 6 |
| Effect of Lactobacillus reuteri on gingival inflammation and composition of the oral microbiota in patients undergoing treatment with fixed orthodontic appliances: study protocol of a randomized control trial. | Agossa, Kevimy and Dubar, Marie and Lemaire, Gregoire and Blaizot, Alessandra and Catteau, Celine and Bocquet, Emmanuel and Nawrocki, Laurent and Boyer, Emile and Meuric, Vincent and Siepmann, Florence | 6 |
| The efficacy of chlorhexidine gel as an adjunctive treatment for patient with chronic periodontitis | Ahmad B.Z. | 6 |
| Identification of Tannerella forsythia and Treponema denticola in Down syndrome subjects and healthy subjects with periodontal disease - a PCR study. | Ahmed, A. N. and Victor, R. D. | 6 |
| Full-mouth disinfection and systemic antimicrobial therapy in generalized aggressive periodontitis: a randomized, placebo-controlled trial. | Aimetti, M. and Romano, F. and Guzzi, N. and Carnevale, G. | 6 |
| Serial doxycycline and metronidazole in prevention of recurrent periodontitis in high-risk patients. | Aitken, S and Birek, P and Kulkarni, G V and Lee, W L and McCulloch, C A | 6 |
| Application of photodynamic therapy against periodontal bacteria in established gingivitis lesions in adolescent patients undergoing fixed orthodontic treatment | Al Nazeh A. and Alshahrani A. and Almoammar S. and Kamran M.A. and Togoo R.A. and Alshahrani I. | 6 |
| Deoxyribonucleic Acid Probes Analyses for the Detection of Periodontal Pathogens | Al Yahfoufi Z. and Hadchiti W. and Berberi A. | 6 |
| Evaluation of the state of some oral obligate anaerobic and opportunistic microflora by periodontal inflammatory diseases | Al-Cafes M.A.M. and Usmanova I.N. and Gerasimova L.P. and Tuygunov M.M. and Usmanov I.R. and Gubaidullin A.G. | 6 |
| The role of soluble TLR-2 in the immunopathogenesis of gingivitis | Al-Ghurabi B.H. | 6 |
| Comparison between the Efficacy of Herbal and Conventional Dentifrices on Established Gingivitis. | Al-Kholani, Abdulwahab I | 6 |
| Aggressive and acute periodontal diseases | Albandar J.M. | 6 |
| The study effects of quercus infectoria on the oral environment in gingivitis patients | Alhamadani A.H. and Al-Muswie R.T. and Tarfa S.J. | 6 |
| Clinical study on the therapeutic effects of Quercus infectoria galls as oral powder in gingivitis and plaque patients | Alhamadani A.H. and Saeed H.A. and Khayoon H.A. AO - Alhamadani, Ali Hasanain; ORCID: https://orcid.org/0000-0002-9704-7862 | 6 |
| The effects of salvia officinalis gel as an adjunct to scaling and root planning in patients with periodontitis (Clinical and immunological study) | Aljuboori I.W. and Mahmood M.S. | 6 |
| Proportion of antibiotic resistance in subgingival plaque samples from Mexican subjects | Almaguer-Flores A. and Moreno-Borjas J.-Y. and Salgado-Martinez A. and Sanchez-Reyes M.-A. and Alcantara-Maruri E. and Ximenez-Fyvie L.-A. | 6 |
| Dental risk factors associated with oral Helicobacter pylori infection: a cross-sectional study based on saliva antigen test. | Almashhadany, Dhary Alewy and Zefenkey, Zean Fetehallah and Zaki, Ahmed Mohammed | 6 |
| Bacterial diversity and prevalence of antibiotic resistance genes in the oral microbiome. | Almeida, Viviane de Sousa Moreira and Azevedo, Jailton and Leal, Helena Ferreira and Queiroz, Artur Trancoso Lopo de and da Silva Filho, Hermes Pedreira and Reis, Joice Neves | 6 |
| Short-term improvement of clinical parameters and microbial diversity in periodontitis patients following Indocyanine green-based antimicrobial photodynamic therapy: A randomized single-blind split-mouth cohort | AlSarhan M.A. and Altammami M.A. and Alaqeely R.S. and AlEbdi A. and Jasser R.A. and Otaibi D.A. and Oraini S.A. and Habib S.R. and Alqahtani L. and Alduhaymi I.S. and Alrabiah D.K. and Alaradi M. and Alyamani E.J. | 6 |
| Long-term clinical effect of adjunctive antimicrobial photodynamic therapy in periodontal treatment: a randomized clinical trial | Alwaeli H.A. and Al-Khateeb S.N. and Al-Sadi A. | 6 |
| Evaluation of Gingegel Gel for the Treatment of Gingivitis: A randomized clinical trial | Alwan A.H. and Alghazali M.W. and Hussain A.A. | 6 |
| Clinical and microbiological evaluation of the effectiveness of the Nd:Yap laser for the initial treatment of adult periodontitis. A randomized controlled study. | Ambrosini, Pascal and Miller, Neal and Briancon, Serge and Gallina, Sebastien and Penaud, Jacques | 6 |
| Detection and quantification of Porphyromonas gingivalis and Aggregatibacter actinomycetemcomitans in bacteremia induced by interdental brushing in periodontally healthy and periodontitis patients. | Ambrosio, N. and Marin, M. J. and Laguna, E. and Herrera, D. and Sanz, M. and Figuero, E. | 6 |
| Detrimental effects of specific Periodontopathic bacterial infection on tachyarrhythmia compared to Bradyarrhythmia | Aoyama N. and Suzuki J.-I. and Kobayashi N. and Hanatani T. and Ashigaki N. and Yoshida A. and Shiheido Y. and Sato H. and Kumagai H. and Ikeda Y. and Akazawa H. and Komuro I. and Minabe M. and Izumi Y. and Isobe M. | 6 |
| Impact of smoking on the clinical, microbiological and immunological parameters of adult patients with periodontitis | Apatzidou D.A. and Riggio M.P. and Kinane D.F. | 6 |
| Express Diagnostics of Proteolytic Activity of Periodontopathogens-Methodological Approach. | Aronova, Ekaterina and Dmitrienko, Marina and Ivanova, Anastasija and Gaykova, Yulia and Kurochkina, Anna and Blinova, Alisa and Bazarnova, Julia and Paponova, Elizaveta | 6 |
| Resistance to beta-lactams and distribution of beta-lactam resistance genes in subgingival microbiota from Spanish patients with periodontitis | Arredondo A. and Blanc V. and Mor C. and Nart J. and Leon R. AO - Leon, Ruben; ORCID: https://orcid.org/0000-0001-6400-4906 | 6 |
| Azithromycin and erythromycin susceptibility and macrolide resistance genes in Prevotella from patients with periodontal disease. | Arredondo, A. and Blanc, V. and Mor, C. and Nart, J. and Leon, R. | 6 |
| Likelihood of transmitting Actinobacillus actinomycetemcomitans and Porphyromonas gingivalis in families with periodontitis | Asikainen S. and Chen C. and Slots J. | 6 |
| Effect of the diode laser on bacteremia associated with dental ultrasonic scaling: a clinical and microbiological study | Assaf M. and Yilmaz S. and Kuru B. and Ipci S.D. and Noyun U. and Kadir T. | 6 |
| Do periodontopathogens disappear after full-mouth tooth extraction? | Assche, N. van and Essche, M. van and Pauwels, M. and Teughels, W. and Quirynen, M. | 6 |
| Clinical and laboratory characterization of early onset periodontitis | Astemborski J.A. and Boughman J.A. and Myrick P.O. and Goodman S.B. and Wooten R.K. and Agarwal S. and Vincent J.W. and Suzuki J.B. | 6 |
| Adjunctive use of essential oils following scaling and root planing -a randomized clinical trial | Azad M.F. and Schwiertz A. and Jentsch H.F.R. | 6 |
| A comparison of intraoral antimicrobial effects of stabilized stannous fluoride dentifrice, baking soda/peroxide dentifrice, conventional NaF dentifrice and essential oil mouthrinse | Bacca L.A. and Leusch M. and Lanzalaco A.C. and Macksood D. and Bouwsma O.J. and Shaffer J.B. and Howard-Nordan K.S. and Knippenberg S.H. and Kreutzjans M.K. and Miller J.M. and Poore C.L. and Sunberg R.J. and Vastola K.A. and Becus M. and Bartizek R.D. and Block R.P. and Briner W.W. and White D.J. | 6 |
| The diagnosis and monitoring of aggressive periodontitis by salivary 8-OHDG biomarker | Badea V. and Nuca C. and Amariei C. and Voineagu L. and Dobrota A. and Zaharia A. and Arendt C. and Badea C.F. | 6 |
| Researches regarding the monitoring complementary treatment in periodontitis by using salivary 8-OHdG biomarker | Badea V. and Nuca C. and Amariei C. and Zaharia A. and Adriana Bucur L. and Grigorian M. | 6 |
| Cytokine alteration in multiple myeloma (MM) patients and bisphosphonate (BP)-related osteonecrosis of the jaw (BRONJ) | Badros A. and Philip S. and Lesho P. and Sadowska M. and Weikel D. and Meiller T. and Lapidus R. and Hester L. and Milliron T. and Goloubeva O. | 6 |
| Potential of diagnostic microbiology for treatment and prognosis of dental caries and periodontal diseases | Baehni P.C. and Guggenheim B. | 6 |
| Role of pathogenic oral flora in postoperative pneumonia following brain surgery | Bagyi K. and Haczku A. and Marton I. and Szabo J. and Gaspar A. and Andrasi M. and Varga I. and Toth J. and Klekner A. | 6 |
| Detection of enterococcus faecalis in subgingival biofilm of patients with chronic refractory periodontitis | Balaei-Gajan E. and Shirmohammadi A. and Abashov R. and Agazadeh M. and Faramarzie M. | 6 |
| Metabolomics reveals elevated macromolecular degradation in periodontal disease | Barnes V.M. and Ciancio S.G. and Shibly O. and Xu T. and Devizio W. and Trivedi H.M. and Guo L. and Jonsson T.J. | 6 |
| The proteins of Fusobacterium spp. involved in hydrogen sulfide production from L-cysteine | Basic, Amina and Blomqvist, Madeleine and Dahlen, Gunnar and Svensater, Gunnel | 6 |
| Reverse hybridization assay for rapid identification of periodontitis-associated interleukin-1 alleles | Becker M. and Weizenegger M. and Bartel J. | 6 |
| Sampling strategy for intraoral detection of periodontal pathogens before and following periodontal therapy | Beikler T. and Schnitzer S. and Abdeen G. and Ehmke B. and Eisenacher M. and Flemmig T.F. | 6 |
| Microbiological shifts in intra- and extraoral habitats following mechanical periodontal therapy. | Beikler, Thomas and Abdeen, Ghiath and Schnitzer, Stefan and Salzer, Sonja and Ehmke, Benjamin and Heinecke, Achim and Flemmig, Thomas F | 6 |
| Molecular microbiological evaluation of subgingival biofilm sampling by paper point and curette. | Belibasakis, G. N. and Schmidlin, P. R. and Sahrmann, P. | 6 |
| Influence of periodontal treatment on subgingival and salivary microbiotas | Belstrom D. and Grande M.A. and Sembler-Moller M.L. and Kirkby N. and Cotton S.L. and Paster B.J. and Holmstrup P. | 6 |
| Differences in bacterial saliva profile between periodontitis patients and a control cohort. | Belstrom, Daniel and Fiehn, Nils-Erik and Nielsen, Claus H and Kirkby, Nikolai and Twetman, Svante and Klepac-Ceraj, Vanja and Paster, Bruce J and Holmstrup, Palle | 6 |
| Microbial flora in chronic periodontitis: study at a tertiary health care center from north karnataka. | Benachinmardi, Kirtilaxmi K and Nagamoti, Jyoti and Kothiwale, Shaila and Metgud, Sharada C | 6 |
| Additional benefit of systemic antibiotics in subgingival instrumentation of stage III and IV periodontitis with Aggregatibacter actinomycetemcomitans: a retrospective analysis. | Benz, Leander and Winkler, Patrizia and Dannewitz, Bettina and Nickles, Katrin and Petsos, Hari and Aldiri, Talal and Eickholz, Peter | 6 |
| Self-ligating brackets exhibit accumulation of high levels of periodontopathogens in gingival crevicular fluid | Bergamo A.Z.N. and Casarin R.C.V. and do Nascimento C. and Matsumoto M.A.N. and de Carvalho F.K. and da Silva R.A.B. and da Silva L.A.B. and Nelson-Filho P. AO - Bergamo, Ana Zilda Nazar; ORCID: https://orcid.org/0000-0002-3313-2152 | 6 |
| Molecular detection and corelation of Helicobacter pylori in dental plaque and gastric biopsies of dyspeptic patients. | Bharath, T. S. and Reddy, M. S. and Raghu Dhanapal, Raghu Dhanapal and Kumar, N. G. R. and Raju, P. V. N. and Saraswathi, T. R. | 6 |
| Antimicrobial susceptibility pattern of oral isolates of Aggregatibacter actinomycetemcomitans. | Bhat, Kishore G and Khot, Preeti and Patil, Suvarna and Pattar, Geetha and Majukar, Sanjeevini | 6 |
| Novel therapeutic approach for the treatment of periodontitis by curcumin | Bhatia M. and Urolagin S.S. and Pentyala K.B. and Urolagin S.B. and Menaka K.B. and Bhoi S. | 6 |
| Probiotic milk drink as adjuvant therapy for the treatment of periodontitis: a randomized clinical trial with 180 days follow-up. | Bilouro, Fabio Cascardo and Rocha, Ramon Silva and Guimaraes, Jonas Toledo and Pimentel, Tatiana Colombo and Magnani, Marciane and Esmerino, Erick Almeida and de Freitas, Monica Queiroz and Silva, Marcia Cristina and da Cruz, Adriano Gomes and Canabarro, Antonio | 6 |
| Polymerase chain reaction as a prospect for the early diagnosis and prediction of periodontal diseases in adolescents | Birsan I. | 6 |
| Detection of nine microorganisms from the initial carious root lesions using a TaqMan-based real-time PCR | Bizhang M. and Ellerbrock B.I. and Preza D. and Raab W.H.M. and Singh P. and Beikler T. and Henrich B. and Zimmer S. | 6 |
| Clinical, microbiological and oxidative stress evaluation of periodontitis patients treated with two regimens of systemic antibiotics, adjunctive to non-surgical therapy: A placebo-controlled randomized clinical trial | Boia S. and Boariu M. and Baderca F. and Rusu D. and Muntean D. and Horhat F. and Boia E.-R. and Borza C. and Anghel A. and Stratul S.-I. | 6 |
| The effect of a one-stage full-mouth disinfection on different intra-oral niches. Clinical and microbiological observations. | Bollen, C M and Mongardini, C and Papaioannou, W and Van Steenberghe, D and Quirynen, M | 6 |
| Full- versus partial-mouth disinfection in the treatment of periodontal infections. A pilot study: long-term microbiological observations. | Bollen, C M and Vandekerckhove, B N and Papaioannou, W and Van Eldere, J and Quirynen, M | 6 |
| Prevalence of periodontopathogens in a black Brazilian secluded community matched with a black urban population | Bonifacio J.D. and Aquino D.R. and Franco G.C.N. and Cortelli S.C. and Cogo K. and Guimaraes dos Santos J. and Costa F.O. and Cortelli J.R. | 6 |
| Association between periodontal condition and subgingival microbiota in women during pregnancy: a longitudinal study | Borgo P.V. and Rodrigues V.A. and Feitosa A.C. and Xavier K.C. and Avila-Campos M.J. | 6 |
| Local Drug Delivery Systems as Novel Approach for Controlling NETosis in Periodontitis | Bosca, Adina Bianca and Dinte, Elena and Mihu, Carmen Mihaela and Parvu, Alina Elena and Melincovici, Carmen Stanca and Sovrea, Alina Simona and Marginean, Mariana and Constantin, Anne-Marie and Babtan, Anida-Maria and Muntean, Alexandrina and Ilea, Aranka | 6 |
| Local application of tetracycline solution with a microbrush: an alternative treatment for persistent periodontitis | Bosco J.M. and Lopes B.M. and Bosco A.F. and Spolidorio D.M. and Marcantonio R.A. | 6 |
| Comparison of subgingival bacterial sampling with oral lavage for detection and quantification of periodontal pathogens by real-time polymerase chain reaction | Boutaga K. and Savelkoul P.H.M. and Winkel E.G. and van Winkelhoff A.J. | 6 |
| Comparison of Real-Time PCR and Culture for Detection of Porphyromonas gingivalis in Subgingival Plaque Samples | Boutaga K. and Van Winkelhoff A.J. and Vandenbroucke-Grauls C.M.J.E. and Savelkoul P.H.M. | 6 |
| Guided tissue regeneration in the treatment of human infrabony defects. Clinical, radiographical and microbiological results: a pilot study | Bratthall G. and Soderholm G. and Neiderud A.M. and Kullendorff B. and Edwardsson S. and Attstrom R. | 6 |
| Metabolic changes during periodontitis therapy assessed by real-time ambient mass spectrometry | Bregy L. and Hirsiger C. and Gartenmann S. and Bruderer T. and Zenobi R. and Schmidlin P.R. AO - Gartenmann, Stefanie; ORCID: https://orcid.org/0000-0002-8569-451X | 6 |
| Periodontal conditions and distribution of Prevotella intermedia, Porphyromonas gingivalis and Aggregatibacter actinomycetemcomitans in HIV-infected patients undergoing anti-retroviral therapy and in an HIV-seronegative group of the Venezuelan population | Brito A. and Escalona L.A. and Correnti M. and Perrone M. and Bravo I.M. and Tovar V. | 6 |
| Predictive value of clinical and microbiological parameters for the treatment outcome of scaling and root planing. | Brochut, P. F. and Marin, I. and Baehni, P. and Mombelli, A. | 6 |
| Change of antibiotic susceptibility following periodontal therapy. A pilot study in aggressive periodontal disease. | Buchmann, Rainer and Muller, Rudiger F and Van Dyke, Thomas E and Lange, Dieter E | 6 |
| Antimicrobial activity of Desplac oral gel in the subgingival multispecies biofilm formation | Bueno-Silva B. and Kiausinus K.R. and Goncalves F.J.D.S. and Moreira M.V.C. and Oliveira E.G.D. and Brugnera Junior A. and Feres M. and Figueiredo L.C. | 6 |
| Non-Surgical Therapy Reduces Presence of JP2 Clone in Localized Aggressive Periodontitis | Burgess D.K. and Huang H. and Harrison P. and Kompotiati T. and Aukhil I. and Shaddox L.M. | 6 |
| Oral health status in adult patients with newly diagnosed acute leukemia | Busjan R. and Hasenkamp J. and Schmalz G. and Haak R. and Trumper L. and Ziebolz D. AO - Ziebolz, Dirk; ORCID: https://orcid.org/0000-0002-9810-2368 | 6 |
| Progression of chronic periodontitis can be predicted by the levels of Porphyromonas gingivalis and treponema denticola in subgingival plaque | Byrne S.J. and Dashper S.G. and Darby I.B. and Adams G.G. and Hoffmann B. and Reynolds E.C. | 6 |
| Oxygen high level laser therapy is efficient in treatment of chronic periodontitis: A clinical and microbiological study using PCR analysis | Caccianiga G. and Rey G. and Paiusco A. and Lauritano D. and Cura F. and Ormianer Z. and Carinci F. | 6 |
| Multiple sessions of antimicrobial photodynamic therapy associated with surgical periodontal treatment in patients with chronic periodontitis. | Cadore, Uislen B and Reis, Marilia B L and Martins, Sergio H L and Invernici, Marcos de M and Novaes, Arthur B Jr and Taba, Mario Jr and Palioto, Daniela B and Messora, Michel R and Souza, Sergio L S | 6 |
| Predictive, preventive, personalised and participatory periodontology: 'the 5Ps age' has already started. | Cafiero, C. and Matarasso, S. | 6 |
| Structure and Function of Oral Microbial Community in Periodontitis Based on Integrated Data | Cai Z. and Lin S. and Hu S. and Zhao L. | 6 |
| Adjunctive effect of chlorhexidine antiseptics in mechanical periodontal treatment: First results of a preliminary case series | Calderini A. and Pantaleo G. and Rossi A. and Gazzolo D. and Polizzi E. | 6 |
| Rapid antimicrobial resistance screening method for Bacteroides intermedius | Calsina G. and Lee Y.S. and Newman M.G. and Kornman K.S. and Nachnani S. and Flemmig T.F. | 6 |
| Microbiological analysis of gingivitis in pediatric patients under orthodontic treatment | Cardoso-Silva C. and Barberia E. and Ramos Atance J.A. and Maroto M. and Hernandez A. and Garcia-Godoy F. | 6 |
| Use of diode laser 980 nm as adjunctive therapy in the treatment of chronic periodontitis. A randomized controlled clinical trial | Caruso U. and Nastri L. and Piccolomini R. and D'Ercole S. and Mazza C. and Guida L. | 6 |
| [Neutrophil function and microbial associations in rapidly progressing periodontitis and chronic periodontitis in the adult]. | Caruso, F and Guida, L and Sanges, M R and Iuorio, G and Ianniello, R and Galdiero, M P and Tufano, M A | 6 |
| Scaling and root planing, systemic metronidazole and professional plaque removal in the treatment of chronic periodontitis in a Brazilian population. I. Clinical results. | Carvalho, L. H. and D'Avila, G. B. and Leao, A. and Haffajee, A. D. and Socransky, S. S. and Feres, M. | 6 |
| Antimicrobial photodynamic effect to treat residual pockets in periodontal patients: a randomized controlled clinical trial. | Carvalho, V. F. and Andrade, P. V. C. and Rodrigues, M. F. and Hirata, M. H. and Hirata, R. D. C. and Pannuti, C. M. and Micheli, G. de and Conde, M. C. | 6 |
| The combination of amoxicillin and metronidazole improves clinical and microbiologic results of one-stage, full-Mouth, ultrasonic debridement in aggressive periodontitis treatment. | Casarin, R. C. V. and Ribeiro, E. del P. and Sallum, E. A. and Nociti Junior, F. H. and Goncalves, R. B. and Casati, M. Z. | 6 |
| Influence of sampling strategy on microbiologic results before and after periodontal treatment. | Casas, Agustin and Herrera, David and Martin-Carnes, Javier and Gonzalez, Itziar and O'Connor, Ana and Sanz, Mariano | 6 |
| Detection of specific periodontal microorganisms from bacteraemia samples after periodontal therapy using molecular-based diagnostics | Castillo D.M. and Sanchez-Beltran M.C. and Castellanos J.E. and Sanz I. and Mayorga-Fayad I. and Sanz M. and Lafaurie G.I. | 6 |
| Developing an individualized treatment plan for dental patients with accompanying sensitization to bacteria, food, pollen, and more | Cekova-Yaneva M. and Stoeva-Ivanova I. and Nikolov G.S. | 6 |
| Use of fluorescence microscopy for monitoring periodontal disease state | Chan E.C.S. and De Vries J. and Harvey R.F. and Tam Y.C. | 6 |
| Adjunctive effects of a dietary supplement comprising dried whole fruit, vegetable and berry juice concentrates on clinical outcomes of treatment of periodontitis | Chapple I.L.C. and Milward M.M. and Ling-Mountford N. and Weston P. and Dallal G.E. and Matthews J.B. | 6 |
| Bacterial markers vs. clinical markers to predict progression of chronic periodontitis: a 2-yr prospective observational study. | Charalampakis, Georgios and Dahlen, Gunnar and Carlen, Anette and Leonhardt, Asa | 6 |
| Oral microbial community assembly under the influence of periodontitis | Chen H. and Peng S. and Dai L. and Zou Q. and Yi B. and Yang X. and Ma Z.S. | 6 |
| More Than Just a Periodontal Pathogen -the Research Progress on Fusobacterium nucleatum | Chen Y. and Huang Z. and Tang Z. and Huang Y. and Huang M. and Liu H. and Ziebolz D. and Schmalz G. and Jia B. and Zhao J. | 6 |
| Associations between salivary cytokines and periodontal and microbiological parameters in orthodontic patients | Chen Y. and Wong W.K. and Seneviratne J.C. and Huang S. and McGrath C. and Hagg U. AO - Chen, Yong; ORCID: https://orcid.org/0000-0001-8337-4873 | 6 |
| Effects of <ovid:i>Ginkgo biloba</ovid:i> extract on periodontal pathogens and its clinical efficacy as adjuvant treatment | Cheng Qian, Cheng Qian and Gao WeiMin, Gao WeiMin and Cao Bin, Cao Bin and Liu YingMei, Liu YingMei and Lin Mei, Lin Mei and Zhang LiangQiong, Zhang LiangQiong and Wang YiSong, Wang YiSong and Wang ZuoMin, Wang ZuoMin | 6 |
| Effects of Ginkgo biloba extract on periodontal pathogens and its clinical efficacy as adjuvant treatment. | Cheng, Qian and Gao, Wei-min and Cao, Bin and Liu, Ying-mei and Lin, Mei and Zhang, Liang-qiong and Wang, Yi-song and Wang, Zuo-min | 6 |
| The effect of spiramycin on Porphyromonas gingivalis and other "classic" periopathogens | Chiappe V. and Gomez M. and Fernandez-Canigia L. and Romanelli H. | 6 |
| Microbiologic changes in subgingival plaque after removal of fixed orthodontic appliances | Choi D.-S. and Cha B.-K. and Brinkmann P.-G.J. and Lee S.-Y. and Change B.-S. and Jang I. and Song J.-S. | 6 |
| Photodynamic therapy as adjunct to non-surgical periodontal treatment in patients on periodontal maintenance: a randomized controlled clinical trial. | Chondros, Panos and Nikolidakis, Dimitris and Christodoulides, Nicos and Rossler, Ralf and Gutknecht, Norbert and Sculean, Anton | 6 |
| White blood cell count in generalized aggressive periodontitis after non-surgical therapy | Christan C. and Dietrich T. and Hagewald S. and Kage A. and Bernimoulin J.P. | 6 |
| Subgingival distribution of periodontal pathogenic microorganisms in adult periodontitis | Christersson L.A. and Fransson C.L. and Dunford R.G. and Zambon J.J. | 6 |
| Dental bacterial plaques. Nature and role in periodontal disease | Christersson L.A. and Zambon J.J. and Genco R.J. | 6 |
| Periodontal healing after non-surgical therapy with a new ultrasonic device: A randomized controlled clinical trial | Christgau M. and Manner T. and Beuer S. and Hiller K.-A. and Schmalz G. | 6 |
| Photodynamic therapy as an adjunct to non-surgical periodontal treatment: a randomized, controlled clinical trial. | Christodoulides, N. and Nikolidakis, D. and Chondros, P. and Becker, J. and Schwarz, F. and Rossler, R. and Sculean, A. | 6 |
| Surface modification of orthodontic wires with photocatalytic titanium oxide for its antiadherent and antibacterial properties | Chun M.-J. and Shim E. and Kho E.-H. and Park K.-J. and Jung J. and Kim J.-M. and Kim B. and Lee K.-H. and Cho D.-L. and Bai D.-H. and Lee S.-I. and Hwang H.-S. and Ohk S.-H. | 6 |
| Toothbrushing and transient bacteremia in patients undergoing orthodontic treatment | Chung A. and Kudlick E.M. and Gregory J.E. and Royal G.C. and Reindorf C.A. | 6 |
| Adjunctive use of InGaAsP and Er,Cr:YSGG lasers in nonsurgical periodontal therapy: A randomized controlled clinical study | Ciurescu C.E. and Cosgarea R. and Ciurescu D. and Gheorghiu A. and Popa D. and Franzen R. and Arweiler N.B. and Sculean A. and Gutknecht N. | 6 |
| Aggressive periodontitis: The unsolved mystery | Clark D. and Febbraio M. and Levin L. | 6 |
| Oral microbiome changes associated with oral squamous cell carcinoma and salivary gland tumors | Cochrane K. and Podar M. and Carlson E. and Yang Z. and Nodit L. | 6 |
| The effect of delmopinol rinsing on dental plaque formation and gingivitis healing. | Collaert, B and Attstrom, R and De Bruyn, H and Movert, R | 6 |
| Clinical and microbiological features of refractory periodontitis subjects. | Colombo, A P and Haffajee, A D and Dewhirst, F E and Paster, B J and Smith, C M and Cugini, M A and Socransky, S S | 6 |
| Effects of non-surgical mechanical therapy on the subgingival microbiota of Brazilians with untreated chronic periodontitis: 9-month results. | Colombo, A. P. V. and Teles, R. P. and Torres, M. C. and Rosalem Junior, W. and Mendes, M. C. S. and Souto, R. M. and Uzeda, M. de | 6 |
| Impact of periodontal therapy on the subgingival microbiota of severe periodontitis: comparison between good responders and individuals with refractory periodontitis using the human oral microbe identification microarray. | Colombo, Ana Paula V and Bennet, Susan and Cotton, Sean L and Goodson, J Max and Kent, Ralph and Haffajee, Anne D and Socransky, Sigmund S and Hasturk, Hatice and Van Dyke, Thomas E and Dewhirst, Floyd E and Paster, Bruce J | 6 |
| Impact of gingivitis treatment for diabetic patients on quality of life related to periodontal objective parameters: A randomized controlled clinical trial | Cortelli S.C. and Costa F.O. and Gargioni-Filho A. and Aquino D.R. and Cota L.O.M. and Scherma A.P. and Miranda T.B. and Cortelli J.R. | 6 |
| A double-blind randomized clinical trial of subgingival minocycline for chronic periodontitis. | Cortelli, Jose R and Aquino, Davi R and Cortelli, Sheila C and Carvalho-Filho, Jonas and Roman-Torres, Caio V G and Costa, Fernando O | 6 |
| Essential oils in one-stage full-mouth disinfection: double-blind, randomized clinical trial of long-term clinical, microbial and salivary effects. | Cortelli, Sheila Cavalca and Cortelli, Jose Roberto and Holzhausen, Marinella and Franco, Gilson Cesar Nobre and Rebelo, Renato Zanotta and Sonagere, Alan Salinas and Queiroz, Celso da Silva and Costa, Fernando Oliveira | 6 |
| Microbiological and host-derived biomarker evaluation following non-surgical periodontal therapy with short-term administration of systemic antimicrobials: secondary outcomes of an RCT | Cosgarea R. and Eick S. and Jepsen S. and Arweiler N.B. and Juncar R. and Tristiu R. and Salvi G.E. and Heumann C. and Sculean A. | 6 |
| Subgingival chlorhexidine varnish administration as an adjunct to same-day full-mouth root planing. I. Clinical observations. | Cosyn, J. and Wyn, I. and Rouck, T. de and Sabzevar, M. M. | 6 |
| Influence of adjunctive azithromycin on microbiological and clinical outcomes in periodontitis patients: 6-month results of randomized controlled clinical trial. | Cuk, Katarina and Povsic, Katja and Milavec, Suzana and Seme, Katja and Gaspersic, Rok | 6 |
| No evidence of triclosan-resistant bacteria following long-term use of triclosan-containing toothpaste | Cullinan M.P. and Bird P.S. and Heng N.C. and West M.J. and Seymour G.J. | 6 |
| The rag locus of Porphyromonas gingivalis: a novel pathogenicity island | Curtis M.A. and Hanley S.A. and Aduse-Opoku J. | 6 |
| Candida species and selected behavioral factors co-associated with severe early childhood caries: Case-control study | Cvanova M. and Ruzicka F. and Kukletova M. and Lipovy B. and Gachova D. and Izakovicova Holla L. and Danek Z. and Hola V. and Bartosova M. and Jarkovsky J. and Dusek L. and Borilova Linhartova P. | 6 |
| Implications of oral Helicobacter pylori for the outcome of its gastric eradication therapy | Czesnikiewicz-Guzik M. and Loster B. and Bielanski W. and Guzik T.J. and Konturek P.C. and Zapala J. and Konturek S.J. | 6 |
| Low antibiotic resistance among anaerobic Gram-negative bacteria in periodontitis 5 years following metronidazole therapy | Dahlen G. and Preus H.R. | 6 |
| Methodological issues in the quantification of subgingival microorganisms using the checkerboard technique | Dahlen G. and Preus H.R. and Baelum V. | 6 |
| Treatment of periodontal disease based on microbiological diagnosis. A 5-year follow-up on individual patterns. | Dahlen, G and Wikstrom, M and Renvert, S | 6 |
| The effect of periodontal treatment on periodontal bacteria on the oral mucous membranes | Danser M.M. and Timmerman M.F. and van Winkelhoff A.J. and van der Velden U. | 6 |
| Effects of preprocedural mouth rinse on microbial load in aerosols produced during the ultrasonic scaling: A randomized controlled trial | Das S.J. and Kharbuli D. and Alam S. | 6 |
| Effect of adjunctive systemic azithromycin with periodontal surgery in the treatment of chronic periodontitis in smokers: a pilot study. | Dastoor, Sarosh F and Travan, Suncica and Neiva, Rodrigo F and Rayburn, Lindsay A and Giannobile, William V and Wang, Hom-Lay | 6 |
| [The use of antibiotics]. | De Mars, G | 6 |
| Evaluation of bacterial flora composition on teeth and periodontal tissues in patients in treatment with rapid palatal expander | De Santis D. and Pancera P. and Luciano U. and Gelpi F. and Causarano G. and Formentini D. and Marchiori M. and Lanaro L. and Puddu G. and Sinigaglia S. and Bertossi D. and Faccioni P. and Nocini P.F. | 6 |
| One-stage full-mouth disinfection. Long-term microbiological results analyzed by checkerboard DNA-DNA hybridization. | De Soete, M and Mongardini, C and Peuwels, M and Haffajee, A and Socransky, S and van Steenberghe, D and Quirynen, M | 6 |
| Short-term influence of lingual orthodontic therapy on microbial parameters and periodontal status a preliminary study | Demling A. and Demling C. and Schwestka-Polly R. and Stiesch M. and Heuer W. | 6 |
| Principles of periodontology | Dentino A. and Lee S. and Mailhot J. and Hefti A.F. | 6 |
| Effect of ultrasonic scalers on dental plaque and microbial count | Devishree R.A. | 6 |
| The detection of BANA micro-organisms in adult periodontitis before and after scaling and root planing by BANA-Enzymatic TM test kit: An in vivo study. | Dhalla, Nipun and Patil, Sudhir and Chaubey, Krishna Kumar and Narula, Inderpreet Singh | 6 |
| Clinical and microbiological effects of adjunctive, locally delivered chlorhexidine on patients with chronic periodontitis | Dimitra S. and Loannis L. and Malama A. and Theodora S. and Antonis K. | 6 |
| Occurrence and serotype distribution of Aggregatibacter actinomycetemcomitans in subjects without periodontitis in Turkey. | Dogan, B. and Chen, J. and CIftlIklI, S. Y. and Huang, J. and KadIr, T. and Alniak, A. K. and Chen, C. | 6 |
| Fluorescent antibody and flagella stains for rapid detection of bacteria at periodontally healthy and diseased sites | Drisko C.L. and Brandsberg J.W. and Walters P.L. and Killoy W.J. and Tira D.E. | 6 |
| Comparison of sTREM-1 and associated periodontal and bacterial factors before/after periodontal therapy, and impact of psychosocial factors | Dubar M. and Frippiat J.-P. and Remen T. and Boufenzer A. and Alauzet C. and Baumann C. and Gibot S. and Bisson C. AO - Dubar, Marie; ORCID: https://orcid.org/0000-0001-7157-8297 AO - Bisson, ... | 6 |
| Protozoans in subgingival biofilm: clinical and bacterial associated factors and impact of scaling and root planing treatment | Dubar M. and Zaffino M.-L. and Remen T. and Thilly N. and Cunat L. and Machouart M.-C. and Bisson C. AO - Dubar, Marie; ORCID: https://orcid.org/0000-0001-7157-8297 | 6 |
| A randomized, controlled clinical trial on the clinical, microbiological, and staining effects of a novel 0.05% chlorhexidine/herbal extract and a 0.1% chlorhexidine mouthrinse adjunct to periodontal surgery | Duss C. and Lang N.P. and Cosyn J. and Persson G.R. | 6 |
| Characteristics and utilization of antibody measurements in clinical studies of periodontal disease. | Ebersole, J L and Cappelli, D and Steffen, M J | 6 |
| Effects of smoking and treatment status on periodontal bacteria: evidence that smoking influences control of periodontal bacteria at the mucosal surface of the gingival crevice | Eggert F.M. and McLeod M.H. and Flowerdew G. | 6 |
| Microbial changes in patients with acute periodontal abscess after treatment detected by PadoTest | Eguchi T. and Koshy G. and Umeda M. and Iwanami T. and Suga J. and Nomura Y. and Kawanami M. and Ishikawa I. | 6 |
| Adjunctive antimicrobial therapy of periodontitis: long-term effects on disease progression and oral colonization. | Ehmke, B. and Moter, A. and Beikler, T. and Milian, E. and Flemmig, T. F. | 6 |
| Hyaluronic acid as an adjunct after scaling and root planing: A prospective randomized clinical trial | Eick S. and Renatus A. and Heinicke M. and Pfister W. and Stratul S.-I. and Jentsch H. | 6 |
| Comparison of microbial cultivation and a commercial PCR based method for detection of periodontopathogenic species in subgingival plaque samples. | Eick, Sigrun and Pfister, Wolfgang | 6 |
| Comparison of real-time polymerase chain reaction and DNA-strip technology in microbiological evaluation of periodontitis treatment. | Eick, Sigrun and Straube, Anna and Guentsch, Arndt and Pfister, Wolfgang and Jentsch, Holger | 6 |
| Growth kinetics, antigen profiling, and proteinase activity of Egyptian Trichomonas tenax isolates derived from patients having oral infections. | El Sibaei, Mahmoud M and Abdel-Fattah, Nashwa S and Ahmed, Sabah A and Abou-Seri, Hanan M | 6 |
| Subgingival microbial profiles of Sudanese patients with aggressive periodontitis. | Elabdeen, H. R. Z. and Mustafa, M. and Hasturk, H. and Klepac-Ceraj, V. and Ali, R. W. and Paster, B. J. and Dyke, T. van and Bolstad, A. I. | 6 |
| Clinical and Microbiological Outcomes of Topical Aloe Vera Gel vs Photochemotherapy as an Adjunct to Non-surgical Periodontal Treatment in Periodontitis | Elsadek M.F. and Ahmed B.M. and Eskandrani R.M. and Fahmy T.S. | 6 |
| D-PLEX500: a local biodegradable prolonged release doxycycline-formulated bone graft for the treatment for peri-implantitis. A randomized controlled clinical study | Emanuel N. and Machtei E.E. and Reichart M. and Shapira L. | 6 |
| Relationship between herpesviruses and periodontal disease progression | Emecen-Huja P. and Danaher R.J. and Dawson D.R. and Wang C. and Kryscio R.J. and Ebersole J.L. and Miller C.S. AO - Miller, Craig S.; ORCID: https://orcid.org/0000-0002-7657-4604 AO - Eb... | 6 |
| Clinical and microbiological effects of systemic ciprofloxacin and metronidazole in Aggregatibacter actinomycetemcomitans-associated periodontitis. | Esfahanizade, N. and Khalilinejad, S. and Zonubi, L. | 6 |
| Enamel matrix derivative (Emdogain(R)) for periodontal tissue regeneration in intrabony defects | Esposito M. and Grusovin M.G. and Papanikolaou N. and Coulthard P. and Worthington H.V. | 6 |
| Clinical and microbiological evaluation of high intensity diode laser adjutant to non-surgical periodontal treatment: A 6-month clinical trial | Euzebio Alves V.T. and de Andrade A.K.P. and Toaliar J.M. and Conde M.C. and Zezell D.M. and Cai S. and Pannuti C.M. and De Micheli G. | 6 |
| Effectiveness of Pisang Raja Peel Extract (Musa Paradisiaca L) on Bacterial Growth of Porphyromonas Gingivalis as the Cause of Periodontitis | Eva A.F.Z. and Masriadi and Hasanuddin N.R. and Mallombasang A.T.B. and Ajis M. | 6 |
| Prevalence of Porphyromonas gingivalis and Bacteroides forsythus in chronic periodontitis by multiplex PCR. | Faghri, J. and Moghim, S. and Abed, A. M. and Rezaei, F. and Chalabi, M. | 6 |
| Alterations in Oral-Nasal-Pharyngeal Microbiota and Salivary Proteins in Mouth-Breathing Children | Fan C. and Guo L. and Gu H. and Huo Y. and Lin H. | 6 |
| Scaling and root planing and chlorhexidine mouthrinses in the treatment of chronic periodontitis: a randomized, placebo-controlled clinical trial. | Faveri, Marcelo and Gursky, Lauren Christine and Feres, Magda and Shibli, Jamil Awad and Salvador, Sergio Luiz and de Figueiredo, Luciene Cristina | 6 |
| Microbiome changes in young periodontitis patients treated with adjunctive metronidazole and amoxicillin | Feres M. and Retamal-Valdes B. and Fermiano D. and Faveri M. and Figueiredo L.C. and Mayer M.P.A. and Lee J.-J. and Bittinger K. and Teles F. | 6 |
| Antibiotic resistance of subgingival species during and after antibiotic therapy. | Feres, M and Haffajee, A D and Allard, K and Som, S and Goodson, J M and Socransky, S S | 6 |
| Change in subgingival microbial profiles in adult periodontitis subjects receiving either systemically-administered amoxicillin or metronidazole. | Feres, M and Haffajee, A D and Allard, K and Som, S and Socransky, S S | 6 |
| Systemic doxycycline administration in the treatment of periodontal infections (II). Effect on antibiotic resistance of subgingival species. | Feres, M and Haffajee, A D and Goncalves, C and Allard, K A and Som, S and Smith, C and Goodson, J M and Socransky, S S | 6 |
| Clinical and microbiological benefits of strict supragingival plaque control as part of the active phase of periodontal therapy. | Feres, Magda and Gursky, Lauren Christine and Faveri, Marcelo and Tsuzuki, Claudia Ota and Figueiredo, Luciene Cristina | 6 |
| Efficacy of a triclosan/NaF dentifrice in the control of plaque and gingivitis and concurrent oral microflora monitoring | Fine D.H. and Furgang D. and Bonta Y. and DeVizio W. and Volpe A.R. and Reynolds H. and Zambon J.J. and Dunford R.G. | 6 |
| Indicators of periodontal disease activity: an evaluation | Fine D.H. and Mandel I.D. | 6 |
| Microbiological findings after periodontal therapy using Er:YAG laser and currets (preliminary results) | Firkova, E. and Yaneva, B. L. | 6 |
| Differential effects of systemic metronidazole and amoxicillin on Actinobacillus actinomycetemcomitans and Porphyromonas gingivalis in intraoral habitats | Flemmig T.F. and Milian E. and Kopp C. and Karch H. and Klaiber B. | 6 |
| Microbiological markers for prediction and assessment of treatment outcome following non-surgical periodontal therapy | Fujise O. and Hamachi T. and Inoue K. and Miura M. and Maeda K. | 6 |
| Involvement of Porphyromonas gingivalis fimA genotype in treatment outcome following non-surgical periodontal therapy | Fujise O. and Miura M. and Hamachi T. and Maeda K. | 6 |
| Short-term effects of triclosan on healing following subgingival scaling. | Furuichi, Y and Ramberg, P and Krok, L and Lindhe, J | 6 |
| Detection of antibodies against Aggregatibacter actinomycetemcomitans in serum and saliva through ELISA in periodontally healthy individuals and individuals with chronic periodontitis. | Gadekar, N. B. and Hosmani, J. V. and Bhat, K. G. and Kotrashetti, V. S. and Nayak, R. S. and Babji, D. V. and Pattanshetty, S. M. and Joshi, V. M. and Bansode, R. A. | 6 |
| The presence of Enterococcus faecalis in saliva as a risk factor for endodontic infection | Gaeta C. and Marruganti C. and Ali I.A.A. and Fabbro A. and Pinzauti D. and Santoro F. and Neelakantan P. and Pozzi G. and Grandini S. | 6 |
| Occurrence of periodontal pathogens in ethnic groups from a native Brazilian reservation. | Gaetti-Jardim, Elerson Jr and Pereira, Mauricio Fabiano and Vieira, Evanice Menezes Marcal and Schweitzer, Christiane Marie and Okamoto, Ana Claudia and Avila-Campos, Mario J | 6 |
| Periodontal effects of 0.25% sodium hypochlorite twice-weekly oral rinse. A pilot study. | Galvan, M and Gonzalez, S and Cohen, C L and Alonaizan, F A and Chen, C T-L and Rich, S K and Slots, J | 6 |
| Presence and antimicrobial profile of gram-negative facultative anaerobe rods in patients with chronic periodontitis and gingivitis | Gamboa F. and Garcia D.-A. and Acosta A. and Mizrahi D. and Paz A. and Martinez D. and Arevalo A. and Aristizabal F. and Abba M. | 6 |
| Comparison of Laser-assisted and Conventional Flap Surgery with Hydroxyapatite Crystals in the Treatment of Intrabony Defects under Magnification - A Randomised Clinical Trial | Gangolu M. and Pasupuleti M.K. and Penmetsa G.S. and Gottumukkala N.S. and Konathala S.V.R. and Balivada B.P. and Karuturi L. | 6 |
| Effect of systemic matrix metalloproteinase inhibition on periodontal wound repair: A proof of concept trial | Gapski R. and Barr J.L. and Sarment D.P. and Layher M.G. and Socransky S.S. and Giannobile W.V. | 6 |
| Plaque inhibitory effect of a 0.05% cetyl-pyridinium chloride mouth-rinse in a 4-day non-brushing model. | Garcia, V and Rioboo, M and Serrano, J and O'Connor, A and Herrera, D and Sanz, M | 6 |
| The effect of diode laser (980 nm) as an adjunct to non-surgical periodontal treatment | Gargallo-Albiol J. and Biscarri-Rodriguez L. and Arnabat-Dominguez J. and Mendieta C. | 6 |
| Prevalence of six periodontal pathogens in subgingival samples of Italian patients with chronic periodontitis. | Gatto, Maria Rosaria and Montevecchi, Marco and Paolucci, Michela and Landini, Maria Paola and Checchi, Luigi | 6 |
| Effect of 1% ornidazole and 0.25% chlorhexidine gluconate (OrnigreatTM gel) in the treatment of chronic periodontitis: a clinical evaluation. | Gautami, S. P. and Anudeep, M. and Arunbhupathi, P. and Ramyateja, G. | 6 |
| Prevalence of Helicobacter pylori detected by polymerase chain reaction in the oral cavity of periodontitis patients | Gebara E.C.E. and Pannuti C. and Faria C.M. and Chehter L. and Mayer M.P.A. and Lima L.A.P.A. | 6 |
| Effects of Baseplates of Orthodontic Appliances with in situ generated Silver Nanoparticles on Cariogenic Bacteria: A Randomized, Double-blind Cross-over Clinical Trial | Ghorbanzadeh R. and Pourakbari B. and Bahador A. | 6 |
| Effect of periodontal therapy on sulcular sulphide level a longitudinal study | Gleissner C. and Springborn I. and Willershausen B. | 6 |
| Expression of interleukin-17, tumor necrosis factor-alpha, and matrix metalloproteinase-8 in patients with chronic peri-implant mucositis | Gleiznys D. and Kriauciunas A. and Maminskas J. and Stumbras A. and Giedrimiene D. and Niekrash C. and Gleiznys A. and Sakalauskiene J. and Vitkauskiene A. | 6 |
| Microbiological study of the subgingival biofilm in HIV+/HAART patients at a specialized dental service. | Gliosca, Laura A and D Eramo, Luciana R and Bozza, Florencia L and Soken, Luciana and Abusamra, Lorena and Salgado, Pablo A and Squassi, Aldo F and Molgatini, Susana L | 6 |
| Comparison of Oral Microbe Quantities from Tongue Samples and Subgingival Pockets. | Gohler, Andre and Samietz, Stefanie and Schmidt, Carsten Oliver and Kocher, Thomas and Steinmetz, Ivo and Holtfreter, Birte | 6 |
| Effects of full-mouth scaling and root planning in conjunction with systematically administered azithromycin | Gomi K. and Yashima A. and Nagano T. and Kanazashi M. and Maeda N. and Arai T. | 6 |
| A novel approach to the use of subgingival controlled-release chlorhexidine delivery in chronic periodontitis: a randomized clinical trial. | Gonzales, J. R. and Harnack, L. and Schmitt-Corsitto, G. and Boedeker, R. H. and Chakraborty, T. and Domann, E. and Meyle, J. | 6 |
| Minocycline HCl microspheres reduce red-complex bacteria in periodontal disease therapy | Goodson J.M. and Gunsolley J.C. and Grossi S.G. and Bland P.S. and Otomo-Corgel J. and Doherty F. and Comiskey J. | 6 |
| Reduced dental plaque accumulation on composite gold alloy margins | Goodson J.M. and Shoher I. and Imber S. and Som S. and Nathanson D. | 6 |
| Salivary concentrations of macrophage activation-related chemokines are influenced by non-surgical periodontal treatment: a 12-week follow-up study | Grande M.A. and Belstrom D. and Damgaard C. and Holmstrup P. and Kononen E. and Gursoy M. and Gursoy U.K. AO - Damgaard, Christian; ORCID: https://orcid.org/0000-0003-2394-746X | 6 |
| Detection of herpetic viruses in gingival crevicular fluid of patients suffering from periodontal diseases: Prevalence and effect of treatment | Grenier G. and Gagnon G. and Grenier D. | 6 |
| Relationship between the presence or absence of gingival bleeding and the enzymatic BANA test | Grisi M.F. and Correa Filho T.A. and Fanganiello C.L. and Martins Junior W. and Silva-Neto C.R. and Salvador S.L. | 6 |
| Prediction of clinical response to anti-TNF treatment by oral parameters in Crohn's disease | Groselj D. and Grabec I. and Seme K. and Ihan A. and Ferkolj I. | 6 |
| Clinical and microbiological effects of multiple applications of antibacterial photodynamic therapy in periodontal maintenance patients. A randomized controlled clinical study | Grzech-Lesniak K. and Gaspirc B. and Sculean A. | 6 |
| Impact of baseline microbiological status on clinical outcomes in generalized aggressive periodontitis patients treated with or without adjunctive amoxicillin and metronidazole: an exploratory analysis from a randomized controlled clinical trial | Guerrero A. and Nibali L. and Lambertenghi R. and Ready D. and Suvan J. and Griffiths G.S. and Wilson M. and Tonetti M.S. | 6 |
| Investigation of a Novel Predictive Biomarker Profile for the Outcome of Periodontal Treatment. | Gul, Sarhang S and Griffiths, Gareth S and Stafford, Graham P and Al-Zubidi, Mohammed I and Rawlinson, Andrew and Douglas, Charles W I | 6 |
| Proteomics - The research frontier in periodontics. | Gupta, Abhaya and Govila, Vivek and Saini, Ashish | 6 |
| Alveolar bone loss in relation to toll-like receptor 4 and 9 genotypes and <ovid:i>Porphyromonas gingivalis</ovid:i> carriage | Gursoy, U. K. and He, Q. and Pussinen, P. and Huumonen, S. and Kononen, E. | 6 |
| Elevated baseline salivary protease activity may predict the steadiness of gingival inflammation during periodontal healing: a 12-week follow-up study on adults. | Gursoy, Ulvi Kahraman and Fteita, Dareen and Bikker, Floris J. and Grande, Maria Anastasia and Nazmi, Kamran and Gursoy, Mervi and Kononen, Eija and Belstrom, Daniel | 6 |
| Effect of modified Widman flap surgery and systemic tetracycline on the subgingival microbiota of periodontal lesions. | Haffajee, A D and Dzink, J L and Socransky, S S | 6 |
| Clinical, microbiological and immunological features associated with the treatment of active periodontosis lesions. | Haffajee, A D and Socransky, S S and Ebersole, J L and Smith, D J | 6 |
| Patterns of antibody response in subjects with periodontitis. | Haffajee, A D and Socransky, S S and Taubman, M A and Sioson, J and Smith, D J | 6 |
| Clinical and microbiological changes associated with the use of combined antimicrobial therapies to treat "refractory" periodontitis. | Haffajee, A. D. and Uzel, N. G. and Arguello, E. I. and Torresyap, G. and Guerrero, D. M. and Socransky, S. S. | 6 |
| No differences in microbiome changes between anti-adhesive and antibacterial ingredients in toothpastes during periodontal therapy. | Hagenfeld, Daniel and Prior, Karola and Harks, Inga and Jockel-Schneider, Yvonne and May, Theodor W and Harmsen, Dag and Schlagenhauf, Ulrich and Ehmke, Benjamin | 6 |
| Effect of probiotic lozenges on inflammatory reactions and oral biofilm during experimental gingivitis. | Hallstrom, Hadar and Lindgren, Susann and Yucel-Lindberg, Tulay and Dahlen, Gunnar and Renvert, Stefan and Twetman, Svante | 6 |
| Azithromycin as an adjunctive treatment of generalized severe chronic periodontitis: Clinical, microbiologic, and biochemical parameters | Han B. and Emingil G. and Ozdemir G. and Tervahartiala T. and Vural C. and Atilla G. and Baylas H. and Sorsa T. | 6 |
| The microbial community shifts of subgingival plaque in patients with generalized aggressive periodontitis following non-surgical periodontal therapy: a pilot study. | Han, Jing and Wang, Peng and Ge, Shaohua | 6 |
| Antimicrobial effects of a stannous fluoride toothpaste in distinct oral microenvironments | Haraszthy V.I. and Raylae C.C. and Sreenivasan P.K. | 6 |
| Microbiological and clinical effects of an oral hygiene regimen | Haraszthy V.I. and Sreenivasan P.K. | 6 |
| Microbial analysis of subgingival plaque samples compared to that of whole saliva in patients with periodontitis | Haririan H. and Andrukhov O. and Bertl K. and Lettner S. and Kierstein S. and Moritz A. and Rausch-Fan X. | 6 |
| Correlation of histometric, microbial, and clinical indicators of periodontal disease status before and after root planing | Harper D.S. and Robinson P.J. | 6 |
| Effect of 6 months use of a dentifrice and oral rinse containing sanguinaria extract and zinc chloride upon the microflora of the dental plaque and oral soft tissues. | Harper, D S and Mueller, L J and Fine, J B and Gordon, J and Laster, L L | 6 |
| 6-month use of 0.2% delmopinol hydrochloride in comparison with 0.2% chlorhexidine digluconate and placebo (II). Effect on plaque and salivary microflora | Hase J.C. and Edwardsson S. and Rundegren J. and Attstrom R. and Kelty E. | 6 |
| Effects of subgingival chlorhexidine irrigation in chronic moderate periodontitis. | Haskel, E and Esquenasi, J and Yussim, L | 6 |
| Prevention of Recurrent Childhood Caries with Probiotic Supplements: A Randomized Controlled Trial with a 12-Month Follow-Up | Hasslof P. and Granqvist L. and Stecksen-Blicks C. and Twetman S. AO - Hasslof P.; ORCID: https://orcid.org/0000-0002-7670-3103 | 6 |
| Gingival crevicular fluid from pregnant women impairs trophoblast cell function and trophoblast-neutrophil interaction | Hauk V. and D'Eramo L. and Calo G. and Merech F. and Doga L. and Lara B. and Gliosca L. and Massone C. and Molgatini S. and Ramhorst R. and Squassi A. and Perez Leiros C. | 6 |
| Stromal-derived factor-1 alpha (CXCL12) levels increase in periodontal disease. | Havens, A. M. and Chiu, E. and Taba Junior, M. and Wang, J. C. and Shiozawa, Y. and Jung, Y. H. and Taichman, L. S. and D'Silva, N. J. and Gopalakrishnan, R. and Wang, C. Y. and Giannobile, W. V. and Taichman, R. S. | 6 |
| Quantitative analysis of microbiota in saliva, supragingival, and subgingival plaque of Chinese adults with chronic periodontitis | He J. and Huang W. and Pan Z. and Cui H. and Qi G. and Zhou X. and Chen H. | 6 |
| Short-term microbiological effects of scaling and root planing and essential-oils mouthwash in Chinese adults | He J.-Y. and Qi G.-G. and Huang W.-J. and Sun X.-D. and Tong Y. and Peng C.-M. and Zhou X.-P. and Chen H. | 6 |
| Microbial colonization patterns predict the outcomes of surgical treatment of intrabony defects. | Heitz-Mayfield, Lisa and Tonetti, Maurizio S and Cortellini, Pierpaolo and Lang, Niklaus P and European Research Group on Periodontology (ERGOPERIO) | 6 |
| Assessment of Krillase chewing gum for the reduction of gingivitis and dental plaque | Hellgren K. | 6 |
| Microbiological effects of periodontal therapy plus azithromycin in patients with diabetes: results from a randomized clinical trial. | Hincapie, Juan P and Castrillon, Cesar A and Yepes, Fanny L and Roldan, Natalia and Becerra, Maria A and Moreno, Sandra M and Consuegra, Jessika and Contreras, Adolfo and Botero, Javier E | 6 |
| Effects of combined topical metronidazole and mechanical treatment on the subgingival flora in deep periodontal pockets in cuspids and biscuspids | Hitzig C. and Fosse T. and Charbit Y. and Bitton C. and Hannoun L. | 6 |
| Povidone-iodine as a periodontal pocket disinfectant | Hoang T. and Jorgensen M.G. and Keim R.G. and Pattison A.M. and Slots J. | 6 |
| A prospective randomized clinical trial into the capacity of a toothpaste containing NovaMin to prevent white spot lesions and gingivitis during orthodontic treatment | Hoffman D.A. and Clark A.E. and Rody W.J. and McGorray S.P. and Wheeler T.T. | 6 |
| Effect of initial therapy on dynamics of immunogloblin G levels to some periodontopathic bacteria in serum and gingival crevicular fluid | Hosaka Y. and Saito A. and Nakagawa T. and Seida K. and Yamada S. and Okuda K. | 6 |
| Second Generation of Zafirlukast Derivatives with Improved Activity against the Oral Pathogen Porphyromonas gingivalis | Howard K.C. and Gonzalez O.A. and Garneau-Tsodikova S. AO - Garneau-Tsodikova, Sylvie; ORCID: https://orcid.org/0000-0002-7961... | 6 |
| Microbial signatures of oral dysbiosis, periodontitis and edentulism revealed by Gene Meter methodology. | Hunter, M Colby and Pozhitkov, Alex E and Noble, Peter A | 6 |
| Microbiologic Findings in Relation to Risk Assessment for Periodontal Disease: A Cross-Sectional Study | Hur Y. and Choi S.K. and Ogata Y. and Stark P.C. and Levi P.A. | 6 |
| Piperacillin-tazobactam as an adjuvant in the mechanical treatment of patients with periodontitis: a randomized clinical study. | Hurtado-Celotti, Dolores and Martinez-Rodriguez, Natalia and Ruiz-Saenz, Pedro Luis and Barona-Dorado, Cristina and Santos-Marino, Juan and Martinez-Gonzalez, Jose Maria | 6 |
| Effects of orthodontic bands on microbiologic and clinical parameters. | Huser, M C and Baehni, P C and Lang, R | 6 |
| Metronidazole Potentiation by Panax Ginseng and Symphytum officinale: A New Strategy for P. gingivalis Infection Control | Ibrahim S.M. and Al-Mizraqchi A.S. and Haider J. AO - Ibrahim, Salah M.; ORCID: https://orcid.org/0009-0003-5842-5079 AO - Hai... | 6 |
| Chronic gingivitis: The prevalence of periodontopathogens and therapy efficiency | Igic M. and Kesic L. and Lekovic V. and Apostolovic M. and Mihailovic D. and Kostadinovic L. and Milasin J. | 6 |
| First trimester gingival crevicular fluid placental alkaline phosphatase and the prediction of preeclampsia | Illanes S. and Chaparro A. and Ornella R. and Ramirez V. and Kusanovic J.P. and Rice G. and Romero R. | 6 |
| Clinical and biochemical evaluation of lozenges containing Lactobacillus reuteri as an adjunct to non-surgical periodontal therapy in chronic periodontitis. | Ince, G. and Gursoy, H. and IpcI, S. D. and Cakar, G. and EmeklI-Alturfan, E. and Yilmaz, S. | 6 |
| Probiotic effects of orally administered Lactobacillus reuteri-containing tablets on the subgingival and salivary microbiota in patients with gingivitis. A randomized clinical trial. | Iniesta, Margarita and Herrera, David and Montero, Eduardo and Zurbriggen, Milena and Matos, Ana R and Marin, Maria J and Sanchez-Beltran, Mari C and Llama-Palacio, Arancha and Sanz, Mariano | 6 |
| Effects of Bifidobacterium probiotic on the treatment of chronic periodontitis: a randomized clinical trial. | Invernici, M. M. and Salvador, S. L. and Silva, P. H. F. and Soares, M. S. M. and Casarin, R. and Palioto, D. B. and Souza, S. L. S. and Taba Junior, M. and Novaes Junior, A. B. and Furlaneto, F. A. C. and Messora, M. R. | 6 |
| Hand instrumentation versus ultrasonic debridement in the treatment of chronic periodontitis: a randomized clinical and microbiological trial. | Ioannou, I. and Dimitriadis, N. and Papadimitriou, K. and Sakellari, D. and Vouros, I. and Konstantinidis, A. | 6 |
| Inhibitory Effect of Nepeta deflersiana on Climax Bacterial Community Isolated from the Oral Plaque of Patients with Periodontal Disease | Irfan S. and Abohashrh M. and Wahab S. and Abullais S.S. and Javali M.A. and Nisar N. and Alam M.M. and Srivastava S. and Saleem M. and Zaman G.S. and Ahmad I. and Mansuri N. AO - Ahmad, Irfan; ORCID: https://orcid.org/0000-0002-9500-4623 AO - Zaman, ... | 6 |
| Point-of-care detection of Tannerella forsythia using an antigen-antibody assisted dielectrophoretic impedance measurement method | Ishii Y. and Imamura K. and Kikuchi Y. and Miyagawa S. and Hamada R. and Sekino J. and Sugito H. and Ishihara K. and Saito A. | 6 |
| The effects of a desiccant agent in the treatment of chronic periodontitis: a randomized, controlled clinical trial | Isola G. and Matarese G. and Williams R.C. and Siciliano V.I. and Alibrandi A. and Cordasco G. and Ramaglia L. | 6 |
| Clinical evaluation of salivary periodontal pathogen levels by real-time polymerase chain reaction in patients before dental implant treatment | Ito T. and Yasuda M. and Kaneko H. and Sasaki H. and Kato T. and Yajima Y. | 6 |
| The effect of antimicrobial periodontal treatment on circulating tumor necrosis factor-alpha and glycated hemoglobin level in patients with type 2 diabetes | Iwamoto Y. and Nishimura F. and Nakagawa M. and Sugimoto H. and Shikata K. and Makino H. and Fukuda T. and Tsuji T. and Iwamoto M. and Murayama Y. | 6 |
| Non-Surgical Periodontal Treatment Impact on Subgingival Microbiome and Intra-Oral Halitosis. | Izidoro, Catarina and Botelho, Joao and Machado, Vanessa and Reis, Ana Mafalda and Proenca, Luis and Barroso, Helena and Alves, Ricardo and Mendes, Jose Joao | 6 |
| Effect of periodontal therapy on circulating levels of endotoxin in women with periodontitis: a pilot clinical trial. | Jacob, Shaju P and Nath, Sonia and Zade, R M | 6 |
| Clinical and microbiological characterization of periodontal abscesses. | Jaramillo, Adriana and Arce, Roger Mauricio and Herrera, David and Betancourth, Marisol and Botero, Javier Enrique and Contreras, Adolfo | 6 |
| Comparing the effectiveness of probiotic, green tea, and chlorhexidine- and fluoride-containing dentifrices on oral microbial flora: a double-blind, randomized clinical trial. | Jayashri Prabakar, Jayashri Prabakar and Joseph John, Joseph John and Arumugham, I. M. and Kumar, R. P. and Sakthi, D. S. | 6 |
| Oral microbial shift following 1-month supplementation of probiotic chewable tablets containing Lactobacillus reuteri UBLRu-87 as an adjunct to phase 1 periodontal therapy in chronic periodontitis patients: a randomized controlled clinical trial. | Jebin, A. Aysha and Nisha, Kj and Padmanabhan, Shyam | 6 |
| Nd:YAG (1064 nm) laser for the treatment of chronic periodontitis: a pilot study. | Jensen, Jorgen and Lulic, Martina and Heitz-Mayfield, Lisa J A and Joss, Andreas and Lang, Niklaus P | 6 |
| Nonsurgical therapy of chronic periodontitis with adjunctive systemic azithromycin or amoxicillin/metronidazole | Jentsch H.F. and Buchmann A. and Friedrich A. and Eick S. | 6 |
| Role of cathepsin D induced by Porphyromonas gingivalis lipopolysaccharide in periodontitis | Jeong H.W. and Chang D.S. and Kim J.S. and Hwang Y.S. AO - Jeong, Hyun Woong; ORCID: https://orcid.org/0000-0002-2772-1487 AO - Hw... | 6 |
| Exploring oral bacterial compositional network in two oral disease groups using a convergent approach of NGS-molecular diagnostics | Jeong, Jinuk and Ahn, Kung and Yun, Kyeongeui and Kim, Minseo and Choi, Yeseul and Han, Miyang and Mun, Seyoung and Kim, Yeon-Tae and Lee, Kyung Eun and Kim, Moon-Young and Ahn, Yongju and Han, Kyudong | 6 |
| Prevalence and Antibiotic Susceptibility Trends of Selected Enterobacteriaceae, Enterococci, and Candida albicans in the Subgingival Microbiota of German Periodontitis Patients: A Retrospective Surveillance Study | Jepsen K. and Falk W. and Brune F. and Cosgarea R. and Fimmers R. and Bekeredjian-Ding I. and Jepsen S. | 6 |
| Prevalence and antibiotic susceptibility trends of periodontal pathogens in the subgingival microbiota of German periodontitis patients: A retrospective surveillance study | Jepsen K. and Falk W. and Brune F. and Fimmers R. and Jepsen S. and Bekeredjian-Ding I. AO - Jepsen, Karin; ORCID: https://orcid.org/0000-0002-1015-3145 AO ... | 6 |
| Quantification of periodontal pathogens by paper point sampling from the coronal and apical aspect of periodontal lesions by real-time PCR | Jervoe-Storm P.-M. and AlAhdab H. and Koltzscher M. and Fimmers R. and Jepsen S. | 6 |
| Microbiological outcomes of quadrant versus full-mouth root planing as monitored by real-time PCR | Jervoe-Storm P.-M. and Alahdab H. and Semaan E. and Fimmers R. and Jepsen S. | 6 |
| Point-of-care diagnosis of periodontitis using saliva: Technically feasible but still a challenge | Ji S. and Choi Y. | 6 |
| An update on periodontal aetiopathogenesis and clinical implications | Jin L. | 6 |
| Persistence of extracrevicular bacterial reservoirs after treatment of aggressive periodontitis. | Johnson, Jason D and Chen, Ruoqiong and Lenton, Patricia A and Zhang, Guizhen and Hinrichs, James E and Rudney, Joel D | 6 |
| Mechanical biofilm disruption causes microbial and immunological shifts in periodontitis patients. | Johnston, W and Rosier, B T and Artacho, A and Paterson, M and Piela, K and Delaney, C and Brown, J L and Ramage, G and Mira, A and Culshaw, S | 6 |
| Microbiological and clinical effects of a dentifrice containing zinc citrate and Triclosan in the human experimental gingivitis model | Jones C.L. and Saxton C.A. and Ritchie J.A. | 6 |
| Periodontal antimicrobials - Finding the right solutions | Jorgensen M.G. and Aalam A. and Slots J. | 6 |
| Prevalence and abundance of 9 periodontal pathogens in the saliva of periodontally healthy adults and patients undergoing supportive periodontal therapy. | Jung, Woo-Ri and Joo, Ji-Young and Lee, Ju-Youn and Kim, Hyun-Joo | 6 |
| Association between self-efficacy and loss to follow-up in long-term periodontal treatment. | Kakudate, N. and Morita, M. and Yamazaki, S. and Fukuhara, S. and Sugai, M. and Nagayama, M. and Kawanami, M. and Chiba, I. | 6 |
| Clinical and microbiological effects of subgingival application of a chlorhexidine gel in chronic periodontitis. A pilot study. | Kalaitzakis, C J and Tynelius-Bratthall, G and Attstrom, R | 6 |
| Herpes viruses and periodontopathic bacteria in early-onset periodontitis | Kamma J.J. and Contreras A. and Slots J. | 6 |
| Chronic periodontitis is associated with spinal dysmobility in patients with ankylosing spondylitis. | Kang EunHa, Kang EunHa and Lee JungTae, Lee JungTae and Lee HyoJung, Lee HyoJung and Lee JooYoun, Lee JooYoun and Chang SungHae, Chang SungHae and Cho HyonJoung, Cho HyonJoung and Choi ByoongYong, Choi ByoongYong and Ha YouJung, Ha YouJung and Park KyoungUn, Park KyoungUn and Song YeongWook, Song YeongWook and Dyke, T. E. van and Lee YunJong, Lee YunJong | 6 |
| Effectiveness of chlorhexidine on patients with periodontitis | Kannan B. and Ramamurthy J. and Mani G. and Ganapathy D. | 6 |
| Assessment of clinical efficacy of locally delivered 0.2% Thymoquinone gel in the treatment of periodontitis | Kapil H. and Suresh D.K. and Bathla S.C. and Arora K.S. AO - Arora, Karandeep Singh; ORCID: https://orcid.org/0000-0003-1753-2305 | 6 |
| Diode Laser as an Adjunct to Kirkland Flap Surgery-A Randomized Split-Mouth Clinical and Microbiological Study | Karthikeyan J. and Vijayalakshmi R. and Mahendra J. and Kanakamedala A.K. and Chellathurai B.N.K. and Selvarajan S. and Namachivayam A. | 6 |
| Evaluation of curcumin gel as adjunct to scaling & root planing in management of periodontitis- randomized clinical & biochemical investigation | Kaur H. and Grover V. and Malhotra R. and Gupta M. | 6 |
| Exploration of correlation of oral hygiene and condition with influenza infection | Kawamoto M. and Tanaka H. and Sakurai A. and Otagiri H. and Karasawa I. and Yamada S.-I. and Kurita H. | 6 |
| Estimation of unstimulated salivary creatine phosphokinase in the patients with periodontitis | Keerthana R. and Vishnupriya V. and Gayathri R. | 6 |
| Effect of tablets containing probiotic candidate strains on gingival inflammation and composition of the salivary microbiome: a randomised controlled trial. | Keller, M. K. and Brandsborg, E. and Holmstrom, K. and Twetman, S. | 6 |
| A rationale for management of periodontal diseases: rapid identification of microbial 'therapeutic targets' with phase-contrast microscopy. | Keyes, P H and Rams, T E | 6 |
| Antibacterial activity of Salvadora persica against oral pathogenic bacterial isolates | Khalil M.A. and El-Sabbagh M.S. and El Naggar E.B. and El-Erian R.H. | 6 |
| Effect of Propolis mouthwash on clinical periodontal parameters in patients with gingivitis: A double-blinded randomized clinical trial | Kiani S. and Birang R. and Jamshidian N. AO - Kiani, Sima; ORCID: https://orcid.org/0000-0003-2035-5290 | 6 |
| Spotlight on therapeutic efficiency of green synthesis metals and their oxide nanoparticles in periodontitis | Kiarashi, M. and Mahamed, P. and Ghotbi, N. and Tadayonfard, A. and Nasiri, K. and Kazemi, P. and Badkoobeh, A. and Yasamineh, S. and Joudaki, A. | 6 |
| Properties of alkaline phosphatase in the gingival crevicular fluid | Kina J.R. and Yoshida N. and Goseki M. and Sasaki S. and Ishikawa I. | 6 |
| Oral fluid-based biomarkers of alveolar bone loss in periodontitis. | Kinney, Janet S and Ramseier, Christoph A and Giannobile, William V | 6 |
| Antibiotic susceptibility of putative periodontal pathogens in advanced periodontitis patients. | Kleinfelder, J W and Muller, R F and Lange, D E | 6 |
| Bacterial susceptibility to amoxicillin and potassium clavulanate in advanced periodontitis patients not responding to mechanical therapy. | Kleinfelder, J W and Muller, R F and Lange, D E | 6 |
| Clinical Effects of Stabilized Stannous Fluoride Dentifrice in Reducing Plaque Microbial Virulence I: Microbiological and Receptor Cell Findings | Klukowska M. and Haught J.C. and Xie S. and Circello B. and Tansky C.S. and Khambe D. and Huggins T. and White D.J. | 6 |
| Susceptibility of periodontopathogenic and cariogenic bacteria to defensins and potential therapeutic use of defensins in oral diseases | Komatsuzawa H. and Ouhara K. and Kawai T. and Yamada S. and Fujiwara T. and Shiba H. and Kurihara H. and Taubman M.A. and Sugai M. | 6 |
| A rapid DNA probe method for detection of Porphyromonas gingivalis and Actinobacillus actinomycetemcomitans | Komiya A. and Kato T. and Nakagawa T. and Saito A. and Takahashi J. and Yamada S. and Okuda K. | 6 |
| Detection of Tannerella forsythia and/or Prevotella intermedia might be useful for microbial predictive markers for the outcome of initial periodontal treatment in Koreans | Kook J.-K. and Sakamoto T. and Nishi K. and Kim M.-K. and Seong J.-H. and Son Y.N. and Kim D.-K. | 6 |
| Human oral, gut, and plaque microbiota in patients with atherosclerosis | Koren O. and Spor A. and Felin J. and Fak F. and Stombaugh J. and Tremaroli V. and Behre C.J. and Knight R. and Fagerberg B. and Ley R.E. and Backhed F. | 6 |
| Refractory periodontitis: critical questions in clinical management | Kornman K.S. | 6 |
| The influence of supragingival plaque control on clinical and microbial outcomes following the use of antibiotics for the treatment of periodontitis | Kornman K.S. and Newman M.G. and Moore D.J. and Singer R.E. | 6 |
| Clinical and microbiological patterns of adults with periodontitis. | Kornman, K S and Newman, M G and Alvarado, R and Flemmig, T F and Nachnani, S and Tumbusch, J | 6 |
| Clinical and microbiological evaluation of therapy for juvenile periodontitis. | Kornman, K S and Robertson, P B | 6 |
| Two subgingival plaque-sampling strategies used with RNA probes | Krigar D.-M. and Kaltschmitt J. and Krieger J.K. and Eickholz P. | 6 |
| Er:YAG laser in the treatment of periodontal sites with recurring chronic inflammation: a 12-month randomized, controlled clinical trial. | Krohn-Dale, Ivar and Boe, Olav E and Enersen, Morten and Leknes, Knut N | 6 |
| Real-time polymerase chain reaction quantification of human cytomegalovirus and Epstein-Barr virus in periodontal pockets and the adjacent gingiva of periodontitis lesions. | Kubar, Ayhan and Saygun, Isil and Ozdemir, Atilla and Yapar, Mehmet and Slots, Jorgen | 6 |
| Evaluation of efficacy of non-thermal atmospheric pressure plasma in treatment of periodontitis: a randomized controlled clinical trial | Kucuk D. and Savran L. and Ercan U.K. and Yarali Z.B. and Karaman O. and Kantarci A. and Saglam M. and Koseoglu S. AO - Koseoglu, Serhat; ORCID: https://orcid.org/0000-0002-4121-2494 | 6 |
| Detection And Quantification Of Treponema Denticola In Subgingival Plaque Of Humans By Polymerase Chain Reaction | Kukreja P. and Kukreja B.J. and Bhat K. | 6 |
| Antibiotic susceptibility patterns of Aggregatibacter actinomycetemcomitans and Porphyromonas gingivalis strains from different decades. | Kulik, E. M. and Thurnheer, T. and Karygianni, L. and Walter, C. and Sculean, A. and Eick, S. | 6 |
| A randomized, placebo-controlled trial of doxycycline: effect on the microflora of recurrent periodontitis lesions in high risk patients. | Kulkarni, G V and Lee, W K and Aitken, S and Birek, P and McCulloch, C A | 6 |
| Detection of Streptococcus anginosus from saliva by real-time polymerase chain reaction. | Kumagai, K. and Sugano, N. and Takane, M. and Iwasaki, H. and Tanaka, H. and Yoshinuma, N. and Suzuki, K. and Ito, K. | 6 |
| Target region selection is a critical determinant of community fingerprints generated by 16S pyrosequencing | Kumar, Purnima S. and Brooker, Michael R. and Dowd, Scot E. and Camerlengo, Terry | 6 |
| The relation of microbiologic data to aspartate aminotransferase enzyme activity in gingival crevicular fluid. | Kuru, B and Noyan, U and Yilmaz, S and Kadir, T and Acar, O and Buget, E | 6 |
| Reliability of findings around healthy implants in association with oral hygiene measures: A clinical, microbiological, and immunological follow-up in edentulous patients | Lachmann S. and Kimmerle-Muller E. and Axmann D. and Gomez-Roman G. and Weber H. and Haas R. | 6 |
| Detection and prevalence of the tetracycline resistance determinant Tet Q in the microbiota associated with adult periodontitis. | Lacroix, J M and Walker, C B | 6 |
| Sulfate-reducing bacteria in relation with other potential periodontal pathogens | Langendijk-Genevaux P.S. and Grimm W.D. and van der Hoeven J.S. | 6 |
| Decrease of sulfate-reducing bacteria after initial periodontal treatment | Langendijk-Genevaux P.S. and Hanssen J.T.J. and Van der Hoeven J.S. | 6 |
| Occurrence of doxycycline resistant bacteria in the oral cavity after local administration of doxycyline in patients with periodontal disease | Larsen T. | 6 |
| Clinical and microbiological results following nonsurgical periodontal therapy with or without local administration of piperacillin/tazobactam | Lauenstein M. and Kaufmann M. and Persson G.R. | 6 |
| Influence of anti-infective periodontal therapy on subgingival microbiota evaluated by chair-side test compared to qPCR - a clinical follow-up study. | Laugisch, Oliver and Auschill, Thorsten M. and Tumbrink, Anne and Sculean, Anton and Arweiler, Nicole B. | 6 |
| A clinical study: Melaleuca, Manuka, Calendula and green tea mouth rinse. | Lauten, J. D. and Boyd, L. and Hanson, M. B. and Lillie, D. and Gullion, C. and Madden, T. E. | 6 |
| Biofilm related to dental implants | Lee A. and Wang H.-L. | 6 |
| Detection of dental plaque and its potential pathogenicity using quantitative light-induced fluorescence | Lee E.-S. and de Josselin de Jong E. and Kim B.-I. AO - Kim, Baek-Il; ORCID: https://orcid.org/0000-0001-8234-2327 | 6 |
| Clinical Efficacy of 1% CHX Gluconate Gel and 0.12% CHX Solution: A Randomized Controlled Trial | Lee S.-Y. and Nam E.-J. AO - Lee, Su-Young; ORCID: https://orcid.org/0000-0002-5502-3037 | 6 |
| Subgingival Microbiome and Specialized Pro-Resolving Lipid Mediator Pathway Profiles Are Correlated in Periodontal Inflammation. | Lee, Chun-Teh and Li, Ruoxing and Zhu, Lisha and Tribble, Gena D and Zheng, W Jim and Ferguson, Brittney and Maddipati, Krishna Rao and Angelov, Nikola and Van Dyke, Thomas E | 6 |
| Fluorescence Spectroscopy Shows Porphyrins Produced by Cultured Oral Bacteria Differ Depending on Composition of Growth Media | Lennon AM. and Brune L. and Techert S. and Buchalla W. | 6 |
| Molecular characterization of oral bacteria isolated from human saliva | Leong S. and Marimuthu S. | 6 |
| Healing following ultrasonic debridement and PVP-iodine in individuals with severe chronic periodontal disease: A randomized, controlled clinical study | Leonhardt A. and Bergstrom C. and Krok L. and Cardaropoli G. | 6 |
| Five-year clinical, microbiological, and radiological outcome following treatment of peri-implantitis in man | Leonhardt A. and Dahlen G. and Renvert S. | 6 |
| Microbiological effect of the use of an ultrasonic device and iodine irrigation in patients with severe chronic periodontal disease: a randomized controlled clinical study. | Leonhardt, Asa and Bergstrom, Christina and Krok, Lena and Cardaropoli, Giuseppe | 6 |
| The short-term effect of apically repositioned flap surgery on the composition of the subgingival microbiota | Levy R.M. and Giannobile W.V. and Feres M. and Haffajee A.D. and Smith C. and Socransky S.S. | 6 |
| Analysis of the salivary microbiome in the periodontal disease patients with hypertension and non-hypertension | Li S. and Zaker R. and Chu X. and Asihati R. and Li C. and Guo X. and Jila P. and Sang X. | 6 |
| Quantification of periodontal pathogens cell counts by capillary electrophoresis. | Li ZhenQing, Li ZhenQing and Chen ShaoXiong, Chen ShaoXiong and Liu ChenChen, Liu ChenChen and Zhang DaWei, Zhang DaWei and Dou XiaoMing, Dou XiaoMing and Yamaguchi, Y. | 6 |
| Association of expression of SLIT3 and HMGB1 in lower gingival crevicular fluid of periodontitis patients with gastric Hp infection. | Li, Guang-Wu and Han, Jing-Ding and Lin, Qi-Yan and Zheng, Xu | 6 |
| The efficacy of proanthocyanidins and secnidazole in the treatment of chronic periodontitis after scaling and root planing therapy. | Li, M and Li, R and Jin, Q and Pang, J and Xu, Z | 6 |
| The Bio-Aging of Biofilms on Behalf of Various Oral Status on Different Titanium Implant Materials | Liao M. and Shi Y. and Chen E. and Shou Y. and Dai D. and Xian W. and Ren B. and Xiao S. and Cheng L. AO - Ren, Biao; ORCID: https://orcid.org/0000-0003-4215-2873 AO - Cheng, Lei; ... | 6 |
| Cementum hypoplasia in teeth affected by juvenile periodontitis | Lindskog S. and Blomlof L. | 6 |
| Skin-prick test for severe marginal periodontitis. | Lindskog, S and Zetterstrom, O and Kamkar, A and Bergman, E and Forsgardh, A and Blomlof, L | 6 |
| Characterization of the antioxidant profile of human saliva in peri-implant health and disease | Liskmann S. and Vihalemm T. and Salum O. and Zilmer K. and Fischer K. and Zilmer M. | 6 |
| Effect of subgingival irrigation with tetrapotassium peroxydiphosphate on scaled and untreated periodontal pockets | Listgarten M.A. and Grossberg D. and Schwimer C. and Vito A. and Gaffar A. | 6 |
| Positive correlation between the proportions of subgingival spirochetes and motile bacteria and susceptibility of human subjects to periodontal deterioration | Listgarten M.A. and Levin S. | 6 |
| Incidence of periodontitis recurrence in treated patients with and without cultivable Actinobacillus actinomycetemcomitans, Prevotella intermedia, and Porphyromonas gingivalis: a prospective study | Listgarten M.A. and Slots J. and Nowotny A.H. and Oler J. and Rosenberg J. and Gregor B. and Sullivan P. | 6 |
| Rationale for using microbiological diagnosis as an adjunct to periodontal treatment. | Listgarten, M A | 6 |
| Microbial composition and pattern of antibiotic resistance in subgingival microbial samples from patients with refractory periodontitis. | Listgarten, M A and Lai, C H and Young, V | 6 |
| Detection of fusobacterium nucleatum and fada adhesin gene in patients with orthodontic gingivitis and non-orthodontic periodontal inflammation. | Liu Ping, Liu Ping and Liu Yi, Liu Yi and Wang JianNing, Wang JianNing and Guo Yang, Guo Yang and Zhang YuJie, Zhang YuJie and Xiao ShuiQing, Xiao ShuiQing | 6 |
| Periodontal disease as a specific, albeit chronic, infection: diagnosis and treatment. | Loesche, W. J. and Grossman, N. S. | 6 |
| Porphyromonas endodontalis in chronic periodontitis: a clinical and microbiological cross-sectional study. | Lombardo Bedran, Telma Blanca and Marcantonio, Rosemary Adriana C and Spin Neto, Rubens and Alves Mayer, Marcia Pinto and Grenier, Daniel and Spolidorio, Luis Carlos and Spolidorio, Denise Palomari | 6 |
| A topical desiccant agent in association with manual debridement in the initial treatment of peri-implant mucositis: A clinical and microbiological pilot study | Lombardo G. and Signoriello A. and Corrocher G. and Signoretto C. and Burlacchini G. and Pardo A. and Nocini P.F. AO - Lombardo, Giorgio; ORCID: https://orcid.org/0000-0003-3677-6831 | 6 |
| A topical desiccant agent in association with ultrasonic debridement in the initial treatment of chronic periodontitis: a clinical and microbiological study. | Lombardo, Giorgio and Signoretto, Caterina and Corrocher, Giovanni and Pardo, Alessia and Pighi, Jacopo and Rovera, Angela and Caccuri, Francesca and Nocini, Pier Francesco | 6 |
| Photodynamic therapy (PDT) in non-surgical treatment of periodontitis | Lopez M.A. and Passarelli P.C. and Marra M. and Lopez A. and D'angelo A. and Moffa A. and Martinez S. and Casale M. and D'addona A. | 6 |
| Clinical, laboratory, and immunological studies of a family with a high prevalence of generalized prepubertal and juvenile periodontitis | Lopez N.J. | 6 |
| Periodontal therapy reduces the rate of preterm low birth weight in women with pregnancy-associated gingivitis | Lopez N.J. and Da Silva I. and Ipinza J. and Gutierrez J. | 6 |
| Early Response and Clinical Efficacy of a Mouthwash Containing Chlorhexidine, Anti Discoloration System, Polyvinylpyrrolidone/Vinyl Acetate and Sodium DNA in Periodontitis Model: A Triple-Blind Randomized Controlled Clinical Trial. | Lorusso, Felice and Tartaglia, Gianluca and Inchingolo, Francesco and Scarano, Antonio | 6 |
| Long-term evaluation of the antimicrobial susceptibility and microbial profile of subgingival biofilms in individuals with aggressive periodontitis. | Lourenco, Talita Gomes Baeta and Heller, Debora and do Souto, Renata Martins and Silva-Senem, Mayra Xavier E and Varela, Victor Macedo and Torres, Maria Cynesia Barros and Feres-Filho, Eduardo Jorge and Colombo, Ana Paula Vieira | 6 |
| Well-maintained patients with a history of periodontitis still harbor a more dysbiotic microbiome than health | Lu H. and He L. and Xu J. and Song W. and Feng X. and Zhao Y. and Meng H. AO - Lu, Hongye; ORCID: https://orcid.org/0000-0003-4079-9681 AO - He, Lu; ORCI... | 6 |
| Effect of adjunctive systemic antibiotics on microbial populations compared with scaling and root planing alone for the treatment of periodontitis: a pilot randomized clinical trial. | Lu, Hong-ye and He, Lu and Jin, Dongsiqi and Zhu, Yun-xuan and Meng, Huan-xin | 6 |
| Bactericidal Effects of Dental and Medical X-Ray Radiation on Oral Micro-Organisms | Lu, W. I. and Lu, D. P. | 6 |
| The relationship between odontogenic bacteraemia and orthodontic treatment procedures | Lucas V.S. and Omar J. and Vieira A. and Roberts G.J. | 6 |
| Identification of salivary microbiota and its association with host inflammatory mediators in periodontitis | Lundmark A. and Hu Y.O.O. and Huss M. and Johannsen G. and Andersson A.F. and Yucel-Lindberg T. | 6 |
| Treatment of subjects with refractory periodontal disease. | Magnusson, I and Low, S B and McArthur, W P and Marks, R G and Walker, C B and Maruniak, J and Taylor, M and Padgett, P and Jung, J and Clark, W B | 6 |
| Antimicrobial efficacy of 3% gingko biloba gel as a local drug delivery agent in the treatment of chronic periodontitis: a cross sectional comparative clinical study. | Mahendra, Jaideep and Kumari, Burnice Nalina and Mahendra, Little and Yagnik, Krutika | 6 |
| The value of systemically administered metronidazole in the modified Widman flap procedure. | Mahmood, M M and Dolby, A E | 6 |
| PReS-FINAL-2037: Association between clinical and microbiological periodontal condition in Colombian patients with juvenile idiophatic arthritis | Malagon C. and Romero C. and Vargas C. and De Avila J. and Lafaurie G.I. and Castillo D.M. and Mosquera A.C. | 6 |
| Clinical and microbiological effects of adjunctive photodynamic diode laser therapy in the treatment of chronic periodontitis: A randomized clinical trial. | Mallineni, Sahana and Nagarakanti, Sreenivas and Gunupati, Sumanth and Bv, Ramesh Reddy and Shaik, Mahaboob V and Chava, Vijay K | 6 |
| Chemotherapeutic agents for controlling plaque and gingivitis. | Mandel, I D | 6 |
| Comparison of the detection of periodontal pathogens in bacteraemia after tooth brushing by culture and molecular techniques | Marin M.-J. and Figuero E. and Gonzalez I. and O'Connor A. and Diz P. and Alvarez M. and Herrera D. and Sanz M. | 6 |
| Validation of a multiplex qPCR assay for detection and quantification of Aggregatibacter actinomycetemcomitans, Porphyromonas gingivalis and Tannerella forsythia in subgingival plaque samples. A comparison with anaerobic culture | Marin M.J. and Ambrosio N. and O'Connor A. and Herrera D. and Sanz M. and Figuero E. | 6 |
| Characterizing peri-implant and sub-gingival microbiota through culturomics. First isolation of some species in the oral cavity. A pilot study | Martellacci L. and Quaranta G. and Fancello G. and D'addona A. and Sanguinetti M. and Patini R. and Masucci L. | 6 |
| A randomized clinical trial on the clinical and microbiological efficacy of a xanthan gel with chlorhexidine for subgingival use. | Matesanz, Paula and Herrera, David and Echeverria, Ana and O'Connor, Ana and Gonzalez, Itziar and Sanz, Mariano | 6 |
| PECULIARITIES OF PERIODONTAL POCKET MICROBIOME IN PATIENTS WITH GENERALIZED PERIODONTITIS IN THE POST-COVID PERIOD | Matviykiv T.I. and Rozhko M.M. and Kutsyk R.V. and Gerelyuk V.I. | 6 |
| Nitrate and a nitrate-reducing Rothia aeria strain as potential prebiotic or synbiotic treatments for periodontitis | Mazurel D. and Carda-Dieguez M. and Langenburg T. and Ziemyte M. and Johnston W. and Martinez C.P. and Albalat F. and Llena C. and Al-Hebshi N. and Culshaw S. and Mira A. and Rosier B.T. AO - Mazurel, Danuta; ORCID: https://orcid.org/0000-0002-9611-1391 AO - Car... | 6 |
| Comparison of DNA probe and ELISA microbial analysis methods and their association with adult periodontitis. | Melvin, W L and Assad, D A and Miller, G A and Gher, M E and Simonson, L and York, A K | 6 |
| Molecular & Immunological approaches in oral inflammatory diseases: A bridge to precision medicine | Mendes K. and Gomes A.T.P.C. and Pinto M. and Marques T. and Correia M. and Rosa N. | 6 |
| Efficacy of the additional use of subgingival air-polishing with erythritol powder in the treatment of periodontitis patients: a randomized controlled clinical trial. Part II: effect on sub-gingival microbiome | Mensi M. and Caselli E. and D'Accolti M. and Soffritti I. and Farina R. and Scotti E. and Guarnelli M.E. and Fabbri C. and Garzetti G. and Marchetti S. and Sordillo A. and Trombelli L. | 6 |
| Oxygen tension (pO2) in untreated human periodontal pockets. | Mettraux, G R and Gusberti, F A and Graf, H | 6 |
| Identification of Bacteroides forsythus in subgingival dental plaque with the aid of a rapid PCR method | Meurman J.H. and Wahlfors J. and Korhonen A. and Alakuijala P. and Vaisanen P. and Torkko H. and Janne J. | 6 |
| Periodontal pathogens and associated factors in aggressive periodontitis: results 5-17 years after active periodontal therapy | Meyer-Baumer, Amelie and Eick, Sigrun and Mertens, Christian and Uhlmann, Lorenz and Hagenfeld, Daniel and Eickholz, Peter and Kim, Ti-Sun and Cosgarea, Raluca | 6 |
| Grade C Molar-Incisor Pattern Periodontitis in Young Adults: What Have We Learned So Far? | Miguel, M. M. V. and Shaddox, L. M. | 6 |
| Clinical and microbiological evaluation of local application efficiency of m-chip in complex treatment of periodontitis | Mikhaylova E.S. and Nikolaeva M.O. and Koroleva I.V. | 6 |
| Impact of Local Drug Delivery of Minocycline on the Subgingival Microbiota during Supportive Periodontal Therapy: A Randomized Controlled Pilot Study. | Miyazawa, Haruna and Nakajima, Takako and Horimizu, Makoto and Okuda, Kazuhiro and Sugita, Noriko and Yamazaki, Kyoko and Li, Lu and Hayashi-Okada, Yoshiko and Arita, Takuya and Nishimoto, Misa and Nishida, Mieko and Genco, Robert J and Yamazaki, Kazuhisa | 6 |
| Effect of a controlled-release chlorhexidine chip on clinical and microbiological parameters and prostaglandin E2 levels in gingival crevicular fluid. | Mizrak, Tansel and Guncu, Guliz N and Caglayan, Feriha and Balci, Tansel Ansal and Aktar, Gulseren Samanci and Ipek, Fikret | 6 |
| Effectiveness of non-surgical periodontal therapy by gingival expression of IL-1beta and IL-6 | Mlachkova A. and Dosseva-Panova V. and Popova C. and Kicheva M. | 6 |
| A tangible prospect for the treatment of gingivitis using a potentially probiotic strain Lactobacillus plantarum MK06 isolated from traditional dairy products: a triple blind randomized clinical trial. | Modiri, Sima and Heidari, Mohadeseh and Shahmohammadi, Rojin and Jabbareh, Leila and Maboudi, Avideh and Moosazadeh, Mahmood and Vali, Hojatollah and Noghabi, Kambiz Akbari | 6 |
| Comparative efficacy of depotphoresis and diode laser for reduction of microbial load and postoperative pain, and healing of periapical lesions: a randomized clinical trial | Moghadam M.D. and Saberi E.A. and Molashahi N.F. and Ebrahimi H.S. | 6 |
| Microbiological monitoring | Mombelli A. | 6 |
| Local antibiotic therapy guided by microbiological diagnosis | Mombelli A. and Schmid B. and Rutar A. and Lang N.P. | 6 |
| Clinical and microbiological changes associated with an altered subgingival environment induced by periodontal pocket reduction. | Mombelli, A and Nyman, S and Bragger, U and Wennstrom, J and Lang, N P | 6 |
| Light-activated disinfection using a light-emitting diode lamp in the red spectrum: Clinical and microbiological short-term findings on periodontitis patients in maintenance. A randomized controlled split-mouth clinical trial | Mongardini C. and Di Tanna G.L. and Pilloni A. | 6 |
| One stage full- versus partial-mouth disinfection in the treatment of chronic adult or generalized early-onset periodontitis. I. Long-term clinical observations | Mongardini C. and Van Steenberghe D. and Dekeyser C. and Quirynen M. | 6 |
| Clinical and microbiological effects of the adjunctive use of probiotics in the treatment of gingivitis: A randomized controlled clinical trial | Montero E. and Iniesta M. and Rodrigo M. and Marin M.J. and Figuero E. and Herrera D. and Sanz M. AO - Montero, Eduardo; ORCID: https://orcid.org/0000-0003-2525-8529 | 6 |
| Clinical effects of Lactobacillus rhamnosus in non-surgical treatment of chronic periodontitis: a randomized placebo-controlled trial with 1-year follow-up. | Morales, A. and Carvajal, P. and Silva, N. and Hernandez, M. and Godoy, C. and Rodriguez, G. and Cabello, R. and Garcia-Sesnich, J. and Hoare, A. and Diaz, P. I. and Gamonal, J. | 6 |
| Antimicrobial photodynamic therapy as an adjunct to non-surgical treatment of aggressive periodontitis: a split-mouth randomized controlled trial | Moreira A.L. and Novaes A.B. and Grisi M.F. and Taba M. and Souza S.L. and Palioto D.B. and de Oliveira P.G. and Casati M.Z. and Casarin R.C. and Messora M.R. | 6 |
| Comparative analysis of putative periodontopathic bacteria by multiplex polymerase chain reaction | Morikawa M. and Chiba T. and Tomii N. and Sato S. and Takahashi Y. and Konishi K. and Numabe Y. and Iwata K. and Imai K. | 6 |
| Periodontopathogenic bacteria in subglottic samples from patients undergoing elective intubation for general anesthesia: A pilot study | Morillo C.M.R. and Saraiva L. and Romito G.A. and Pannuti C.M. and Oliveira H.P. and Peres M.P.S.M. and Carmona M.J.C. and Villar C.C. | 6 |
| Protection effect of gray mangrove extract to oxidative stress after mixed periodontopathogen bacteria infection-animal models | Mulawarmanti D. and Revianti S. | 6 |
| In vivo investigation of diode laser application on red complex bacteria in non-surgical periodontal therapy: a split-mouth randomised control trial | Mulder-van Staden S. and Holmes H. and Hille J. AO - Mulder-van Staden, Sune; ORCID: https://orcid.org/0000-0003-3847-9451 AO ... | 6 |
| Evaluation of the efficacy of a dentifrice containing amine fluoride on gingival status-a clinical investigation | Mulla M. and Kashyap R. and Hegde S. and Maiya A. and Sarpangala M. and Sayed F.R. | 6 |
| The composition of the subgingival microflora of young adults suffering from juvenile periodontitis | Muller H.P. and Flores-de-Jacoby L. | 6 |
| Clinical alterations in relation to the morphological composition of the subgingival microflora following scaling and root planing | Muller H.P. and Hartmann J. and Flores-de-Jacoby L. | 6 |
| Subgingival microbiologic effects of one-time irradiation by CO2 laser: A pilot study | Mullins S.L. and MacNeill S.R. and Rapley J.W. and Williams K.B. and Eick J.D. and Cobb C.M. | 6 |
| Oral antiseptic and periodontitis: a clinical and microbiological study. | Mummolo, Stefano and D'Ercole, Simonetta and Marchetti, Enrico and Campanella, Vincenzo and Martinelli, Diego and Marzo, Giuseppe and Tripodi, Domenico | 6 |
| Clinically considerations about induced experimental periodontitis in rats treated by photodynamic therapy. | Muresan, Stefana and Dreanca, Alexandra and Repciuc, Calin and Tabaran, Flaviu and Pop, Romelia and Dejescu, Cosmina and Sevastre, Bogdan and David, Raluca and Pop, Alexandru Raul and Pantea, Stelian and Oana, Liviu | 6 |
| DNA probe detection of periodontal pathogens in HIV-associated periodontal lesions | Murray P.A. and Winkler J.R. and Peros W.J. and French C.K. and Lippke J.A. | 6 |
| Development of experimentally-induced periodontitis in a Sprague Dawley rat model. | Mustafa, Hana H. and Ali, Ahmed Kh. and Cheng, Chen and Radzi, Rozanaliza and Fong, Lau and Mustapha, Noordin and Dyary, Hewa O. | 6 |
| Community periodontal index of treatment needs index: an indicator of anaerobic periodontal infection. | Muthukumar, S and Suresh, R | 6 |
| The effect of oolong tea as an adjunct to nonsurgical management of chronic periodontitis: A randomized controlled clinical trial | Nafade S. and Agnihotri R. and Kamath S.U. and Shenoy P.A. and Khadher N.A. and Nayak D.D. | 6 |
| Integrated biomarker profiling of smokers with periodontitis | Nagarajan R. and Al-Sabbagh M. and Dawson D. and Ebersole J.L. | 6 |
| Controlled study of lactoperoxidase gel on oral flora and saliva in irradiated patients with oral cancer | Nagy K. and Urban E. and Fazekas O. and Thurzo L. and Nagy E. | 6 |
| Effects of systemic sitafloxacin on periodontal infection control in elderly patients | Nakajima T. and Okui T. and Miyauchi S. and Honda T. and Shimada Y. and Ito H. and Akazawa K. and Yamazaki K. | 6 |
| Changes in the subgingival microbiota and periodontal parameters before and 3 months after bracket placement | Naranjo A.A. and Trivino M.L. and Jaramillo A. and Betancourth M. and Botero J.E. | 6 |
| Analysis of the oral microbiome in a patient with cardiofaciocutaneous syndrome and severe periodontal disease: impact of systemic antibiotic therapy. | Navarro, Carolina Munoz and del Carmen Sanchez Beltran, Maria and Vargas, Carolina Arriagada and Vazquez, Pilar Batalla and Freitas, Marcio Diniz and Posse, Jacobo Limeres and Dios, Pedro Diz and Mato, Eliane Garcia | 6 |
| Assessment of antibacterial effect of hydrogen water on plaque from patients with chronic periodontitis | Nayak A. and Bhatt A. and Bhat K. and Nayak R. and Hooli A. and Naik S. | 6 |
| Assessment of non-surgical periodontal therapy on IL-22 and S100A12 concentration in gingival crevicular fluid | Nejadi R. and Sattari M. and Taleghani F. | 6 |
| Gram-negative periodontal pathogens and bacterial endotoxin in metallic orthodontic brackets with or without an antimicrobial agent: an in-vivo study. | Nelson-Filho, Paulo and Valdez, Remberto Marcelo Argandona and Andrucioli, Marcela Cristina Damiao and Saraiva, Maria Conceicao Pereira and Feres, Magda and Sorgi, Carlos Arterio and Faccioli, Lucia Helena | 6 |
| Salivary MRP-8/14 and the presence of periodontitis-associated bacteria in children with bonded maxillary expansion treatment | Nemec M. and Mittinger N. and Bertl M. and Liu E. and Jonke E. and Andrukhov O. and Rausch-Fan X. AO - Andrukhov, Oleh; ORCID: https://orcid.org/0000-0002-0485-2142 | 6 |
| <ovid:i>A. actinomycetemcomitans</ovid:i> profile and red complex bacterial species of an Afro-Brazilian community: a comparative study | Neris, M. de A. and Cortelli, S. C. and Aquino, D. R. and Miranda, T. B. de and Costa, F. de O. and Cortelli, J. R. | 6 |
| Microbiological assays as predictors of the response to periodontal therapy. | Newcomb, G M and Nixon, K C | 6 |
| Anaerobic oral and dental infection | Newman M.G. | 6 |
| Common dental infections in the primary care setting. | Nguyen, Duc Huu and Martin, James T | 6 |
| Feline periodontal disease and therapy. | Niemiec, B. A. | 6 |
| Clinical and radiographic evaluation of periodontitis in down's syndrome children in South Indian Population | Nizar Ahmed A. and Ramakrishnan T. and Victor D.J. | 6 |
| Assessment of Porphyromonas gingivalis and Aggregatibacter actinomycetemcomitans in Down's syndrome subjects and systemically healthy subjects: a comparative clinical trial. | Nizar Ahmed, Nizar Ahmed and Harinath Parthasarathy, Harinath Parthasarathy and Mohamed Arshad, Mohamed Arshad and Victor, D. J. and Mathew, D. and Siva Sankari, Siva Sankari | 6 |
| Combined effects of Nd:YAG laser irradiation with local antibiotic application into periodontal pockets | Noguchi T. and Sanaoka A. and Fukuda M. and Suzuki S. and Aoki T. | 6 |
| Prospective longitudinal changes in the periodontal inflamed surface area following active periodontal treatment for chronic periodontitis | Nomura Y. and Morozumi T. and Saito A. and Yoshimura A. and Kakuta E. and Suzuki F. and Nishimura F. and Takai H. and Kobayashi H. and Noguchi K. and Takahashi K. and Tabeta K. and Umeda M. and Minabe M. and Fukuda M. and Sugano N. and Hanada N. and Yoshinari N. and Sekino S. and Takashiba S. and Sato S. and Nakamura T. and Sugaya T. and Nakayama Y. and Ogata Y. and Numabe Y. and Nakagawa T. | 6 |
| Salivary biomarkers for predicting the progression of chronic periodontitis | Nomura Y. and Shimada Y. and Hanada N. and Numabe Y. and Kamoi K. and Sato T. and Gomi K. and Arai T. and Inagaki K. and Fukuda M. and Noguchi T. and Yoshie H. | 6 |
| Antimicrobial photodynamic therapy in the non-surgical treatment of aggressive periodontitis: Microbiological profile | Novaes Jr. A.B. and Schwartz-Filho H.O. and De Oliveira R.R. and Feres M. and Sato S. and Figueiredo L.C. | 6 |
| Control of gingival inflammation in a teenager population using ultrasonic prophylaxis. | Novaes Junior, A. B. and Souza, S. L. S. de and Taba Junior, M. and Grisi, M. F. de M. and Suzigan, L. C. and Tunes, R. S. | 6 |
| Pathogenicity of periodontal marker bacteria related to Community Periodontal Index (CPI) and smoking in elderly people from Constanta, Romania | Nuca C. and Badea V. and Zaharia A. and Nuca A.-C. and Badea C.F. | 6 |
| Clinical and microbiological characterization of localized aggressive periodontitis: A cohort study | Oettinger-Barak O. and Sela M.N. and Sprecher H. and Machtei E.E. | 6 |
| Effects of periodontal therapy during pregnancy on periodontal status, biologic parameters, and pregnancy outcomes: A pilot study | Offenbacher S. and Lin D. and Strauss R. and McKaig R. and Irving J. and Barros S.P. and Moss K. and Barrow D.A. and Hefti A. and Beck J.D. | 6 |
| Involvement of periodontopathic anaerobes in aspiration pneumonia | Okuda K. and Kimizuka R. and Abe S. and Kato T. and Ishihara K. | 6 |
| Benefits of Bifidobacterium animalis subsp. lactis Probiotic in Experimental Periodontitis | Oliveira L.F. and Salvador S.L. and Silva P.H. and Furlaneto F.A. and Figueiredo L. and Casarin R. and Ervolino E. and Palioto D.B. and Souza S.L. and Taba M. and Novaes A.B. and Messora M.R. | 6 |
| Azithromycin and full-mouth scaling for the treatment of generalized stage III and IV periodontitis: a 6-month randomized comparative clinical trial. | Oliveira, Alcione M. S. D. and Costa, Fernando O. and Nogueira, Leticia Mara R. and Cortelli, Sheila C. and Oliveira, Peterson A. D and Aquino, Davi R. and Miranda, Tais B. and Cortelli, Jose R. | 6 |
| Antimicrobial photodynamic therapy in the non-surgical treatment of aggressive periodontitis: cytokine profile in gingival crevicular fluid, preliminary results. | Oliveira, R. R. de and Schwartz Filho, H. O. and Novaes Junior, A. B. and Garlet, G. P. and Souza, R. F. de and Taba Junior, M. and Souza, S. L. S. de and Ribeiro, F. J. | 6 |
| Epstein-Barr virus-infected plasma cells in periodontitis lesions | Olivieri C.V. and Raybaud H. and Tonoyan L. and Abid S. and Marsault R. and Chevalier M. and Doglio A. and Vincent-Bugnas S. AO - Olivieri, Charles V.; ORCID: https://orcid.org/0000-0002-5237-7358 | 6 |
| Comparison of three anaerobic culture techniques and media for viable recovery of subgingival plaque bacteria | Olsen I. and Socransky S.S. | 6 |
| Acute focal infections of dental origin | Olsen I. and van Winkelhoff A.J. | 6 |
| Probiotics support resilience of the oral microbiota during resolution after experimental gingivitis - a randomized, double-blinded, placebo-controlled trial. | Olsen, Christine Lundtorp and Massarenti, Laura and Vendius, Vincent Frederik Dahl and Gursoy, Ulvi Kahraman and van Splunter, Annina and Bikker, Floris J. and Gursoy, Mervi and Damgaard, Christian and Markvart, Merete and Belstrom, Daniel | 6 |
| False results associated with darkground microscopy of subgingival plaque. | Omar, A A and Newman, H N | 6 |
| Quasi-randomized trial of effects of perioperative oral hygiene instruction on inpatients with heart diseases using a behavioral six-step method | Omori C. and Ekuni D. and Morita M. and Ohbayashi Y. and Miyake M. | 6 |
| Assessment and management of periodontal infections: a medical-surgical approach | Oringer R.J. and Williams R.C. | 6 |
| Increased antibacterial activity of zinc polycarboxylate cement by the addition of chlorhexidine gluconate in fixed prosthodontics. | Orug, Berrin Ors and Baysallar, Mehmet and Cetiner, Deniz and Kucukkaraaslan, Ayten and Dogan, Berna and Doganci, Levent and Akca, Eralp and Bal, Belgin | 6 |
| Azithromycin as an adjunct to scaling and root planing in the treatment of Porphyromonas gingivalis-associated periodontitis: a pilot study. | Oteo, Alfonso and Herrera, David and Figuero, Elena and O'Connor, Ana and Gonzalez, Itziar and Sanz, Mariano | 6 |
| The probiotics in correction of microbiocenosis and cytokine balance of the oral cavity of patient with chronic inflammatory disease of periodont | Ovcharenko Y. and Maryanenko L. and Bondarenko N. and Nizhnik V. and Erichev V. and Aksenova T. and Bagdasaryan N. | 6 |
| Periodontal treatment in severe aplastic anemia | Oyaizu K. and Mineshiba F. and Mineshiba J. and Takaya H. and Nishimura F. and Tanimoto I. and Arai H. and Takashiba S. | 6 |
| The correlation of gingival crevicular fluid interleukin-8 levels and periodontal status in localized juvenile periodontitis | Ozmeric N. and Bal B. and Balos K. and Berker E. and Bulut S. | 6 |
| Treatment of Papillon-Lefevre syndrome periodontitis | Pacheco J.J. and Coelho C. and Salazar F. and Contreras A. and Slots J. and Velazco C.H. | 6 |
| Identification of a Novel Bacterial Outer Membrane Interleukin-1B-Binding Protein from Aggregatibacter actinomycetemcomitans | Paino A. and Ahlstrand T. and Nuutila J. and Navickaite I. and Lahti M. and Tuominen H. and Valimaa H. and Lamminmaki U. and Pollanen M.T. and Ihalin R. | 6 |
| Effects of triclosan on host response and microbial biomarkers during experimental gingivitis | Pancer B.A. and Kott D. and Sugai J.V. and Panagakos F.S. and Braun T.M. and Teles R.P. and Giannobile W.V. and Kinney J.S. AO - Giannobile, William V.; ORCID: https://orcid.org/0000-0002-7102-9746 | 6 |
| Comparative evaluation of two subgingival irrigating solutions in the management of periodontal disease: a clinico-microbial study. | Pandya, D. J. and Balaji Manohar, Balaji Manohar and Mathur, L. K. and Rajesh Shankarapillai, Rajesh Shankarapillai | 6 |
| Clinical and microbiologic effects of subgingival controlled-release delivery of chlorhexidine chip in the treatment of periodontitis: A multicenter study | Paolantonio M. and D'Angelo M. and Grassi R.F. and Perinetti G. and Piccolomini R. and Pizzo G. and Annunziata M. and D'Archivio D. and D'Ercole S. and Nardi G. and Guida L. | 6 |
| Clinical significance of Actinobacillus actinomycetemcomitans in young individuals during orthodontic treatment. A 3-year longitudinal study. | Paolantonio, M and Pedrazzoli, V and di Murro, C and di Placido, G and Picciani, C and Catamo, G and De Luca, M and Piaccolomini, R | 6 |
| Pyrosequencing Analysis of Subgingival Microbiota in Distinct Periodontal Conditions | Park O.-J. and Yi H. and Jeon J.H. and Kang S.-S. and Koo K.-T. and Kum K.-Y. and Chun J. and Yun C.-H. and Han S.H. | 6 |
| Clinical, microbiological and inflammatory evidence of the efficacy of combination therapy including serratiopeptidase in the treatment of periimplantitis | Passariello C. and Lucchese A. and Pera F. and Gigola P. | 6 |
| A HOSPITAL BASED OBSERVATIONAL ASSESSMENT OF THE MICROFLORA IN THE POST-CHEMOTHERAPY PATIENTS OF ORAL CANCER | Patel D. and Khan Z. and Parekh P. and Shah D. and Malik P. | 6 |
| Bactericidal effect of extracts and metabolites of Robinia pseudoacacia L. on Streptococcus mutans and Porphyromonas gingivalis causing dental plaque and periodontal inflammatory diseases | Patra J.K. and Kim E.S. and Oh K. and Kim H.-J. and Dhakal R. and Kim Y. and Baek K.-H. | 6 |
| Clinical and antimicrobial efficacy of a controlled-release device containing chlorhexidine in the treatment of chronic periodontitis. | Pattnaik, S. and Anand, N. and Chandrasekaran, S. C. and Chandrashekar, L. and Mahalakshmi, K. and Satpathy, A. | 6 |
| Effect of surgical and non-surgical periodontal treatment on periodontal status and subgingival microbiota. | Pedrazzoli, V and Kilian, M and Karring, T and Kirkegaard, E | 6 |
| Prospects application of polypyrrole-based immunosensor to Porphyromonas Gingivalis quantification in subgingival plaque samples | Pei Z. and Quan B. and Niu Z. and Shi S. and Shi L. and Tang C. and Liu Y. | 6 |
| A double-blind, paralleled-arm, placebo-controlled and randomized clinical trial of the effectiveness of probiotics as an adjunct in periodontal care. | Pelekos, George and Ho, Sze Nga and Acharya, Aneesha and Leung, Wai Keung and McGrath, Colman | 6 |
| Efficacy of local use of probiotics as an adjunct to scaling and root planing in chronic periodontitis and halitosis: A randomized controlled trial | Penala S. and Kalakonda B. and Pathakota K.R. and Jayakumar A. and Koppolu P. and Lakshmi B.V. and Pandey R. and Mishra A. | 6 |
| The genomics and metagenomics of asthma severity (GEMAS) study: Rationale and design | Perez-Garcia J. and Hernandez-Perez J.M. and Gonzalez-Perez R. and Sardon O. and Martin-Gonzalez E. and Espuela-Ortiz A. and Mederos-Luis E. and Callero A. and Herrera-Luis E. and Corcuera P. and Sanchez-Machin I. and Poza-Guedes P. and Garcia L.M.G. and Ramirez-Martin P. and Perez-Negrin L. and Izaguirre-Flores H. and Barrios-Recio J. and Perez-Rodriguez E. and Alcoba-Florez J. and Canas J.A. and Munoz J.M.R. and Del Pozo V. and Korta-Murua J. and Mendez L.I.P. and Hernandez-Ferrer M. and Villar J. and Lorenzo-Diaz F. and Pino-Yanes M. | 6 |
| Longitudinal monitoring of subgingival colonization by Actinobacillus actinomycetemcomitans, and crevicular alkaline phosphatase and aspartate aminotransferase activities around orthodontically treated teeth. | Perinetti, G. and Paolantonio, M. and Serra, E. and D'Archivio, D. and D'Ercole, S. and Festa, F. and Spoto, G. | 6 |
| The impact of a low-frequency chlorhexidine rinsing schedule on the subgingival microbiota (the TEETH clinical trial) | Persson G.R. and Yeates J. and Persson R.E. and Hirschi-Imfeld R. and Weibel M. and Kiyak H.A. | 6 |
| Subgingival plaque removal in buccal and lingual sites using a novel low abrasive air-polishing powder. | Petersilka, G J and Steinmann, D and Haberlein, I and Heinecke, A and Flemmig, T F | 6 |
| Effect of orthodontic therapy with fixed and removable appliances on oral microbiota: a six-month longitudinal study. | Petti, S and Barbato, E and Simonetti D'Arca, A | 6 |
| Characterization of Antimicrobial Agent Loaded Eudragit RS Solvent Exchange-Induced In Situ Forming Gels for Periodontitis Treatment. | Phaechamud, Thawatchai and Jantadee, Takron and Mahadlek, Jongjan and Charoensuksai, Purin and Pichayakorn, Wiwat | 6 |
| The impact of Crohn's disease on periodontal status-preliminary results from polibd study | Piatek D. and Korona-Glowniak I. and Sztembis J. and Jarmakiewicz S. and Malm A. and Kiela P.R. and Filip R. | 6 |
| Saliva and serum immune responses in apical periodontitis | Pietiainen M. and Liljestrand J.M. and Akhi R. and Buhlin K. and Johansson A. and Paju S. and Salminen A. and Mantyla P. and Sinisalo J. and Tjaderhane L. and Horkko S. and Pussinen P.J. | 6 |
| Periodontal risk assessment, diagnosis and treatment planning | Pihlstrom B.L. | 6 |
| Treatment of residual periodontal pockets using a hyaluronic acid-based gel: a 12 month multicenter randomized triple-blinded clinical trial. | Pilloni, Andrea and Zeza, Blerina and Kuis, Davor and Vrazic, Domagoj and Domic, Tomislav and Olszewska-Czyz, Iwona and Popova, Christina and Kotsilkov, Kamen and Firkova, Elena and Dermendzieva, Yana and Tasheva, Angelina and Orru, Germano and Sculean, Anton and Prpic, Jelena | 6 |
| Hypochlorous Acid as a Potential Postsurgical Antimicrobial Agent in Periodontitis: A Randomized, Controlled, Non-Inferiority Trial | Plata J.C. and Diaz-Baez D. and Delgadillo N.A. and Castillo D.M. and Castillo Y. and Hurtado C.P. and Neuta Y. and Calderon J.L. and Lafaurie G.I. AO - Diaz-Baez, David; ORCID: https://orcid.org/0000-0001-8890-6250 AO - ... | 6 |
| Clinical effectiveness of photodynamic therapy in the treatment of periodontitis | Polansky R. and Haas M. and Heschl A. and Wimmer G. | 6 |
| Influence of different ceramic materials and surface treatments on the adhesion of Prevotella intermedia | Poole S.F. and Pitondo-Silva A. and Oliveira-Silva M. and Moris I.C.M. and Gomes E.A. | 6 |
| Acyl-substituted dermaseptin S4 derivatives with improved bactericidal properties, including on oral microflora | Porat Y. and Marynka K. and Tam A. and Steinberg D. and Mor A. | 6 |
| Clarithromycin, as an adjunct to non surgical periodontal therapy for chronic periodontitis: A double blinded, placebo controlled, randomized clinical trial | Pradeep A.R. and Kathariya R. | 6 |
| Clinical and microbiological effects of levofloxacin in the treatment of chronic periodontitis: a randomized, placebo-controlled clinical trial | Pradeep A.R. and Singh S.P. and Martande S.S. and Naik S.B. and N P. and Kalra N. and Suke D.K. | 6 |
| Microbiological outcomes of systemic ornidazole use in chronic periodontitis. Part II. | Pradeep, A R and Kalra, Nitish and Priyanka, N and Naik, Savitha B | 6 |
| Clinical and microbiologic effects of subgingivally delivered 0.5% azithromycin in the treatment of chronic periodontitis. | Pradeep, A. R. and Sagar, S. V. and Happy Daisy, Happy Daisy | 6 |
| Modified-release subantimicrobial dose doxycycline enhances scaling and root planing in subjects with periodontal disease. | Preshaw, Philip M and Novak, M John and Mellonig, James and Magnusson, Ingvar and Polson, Alan and Giannobile, William V and Rowland, Randal W and Thomas, John and Walker, Clay and Dawson, Dolphus R and Sharkey, Dennis and Bradshaw, Mark H | 6 |
| Reproducibility of a commercially available subgingival plaque sampling strategy and analysis strategy with oligonucleotide probes. | Pretzl, Bernadette and Paul, Jule and Krigar, Diana M and Uhlmann, Lorenz and Eickholz, Peter and Dannewitz, Bettina | 6 |
| The effect of metronidazole on the presence of P. gingivalis and T. forsythia at 3 and 12 months after different periodontal treatment strategies evaluated in a randomized, clinical trial | Preus H.R. and Gjermo P. and Scheie A.A. and Baelum V. | 6 |
| Effectiveness of crushed bay leaves in removing dental plaque among tobacco chewer's | Priyadarsini A. and Sugantha R. | 6 |
| Effect of photodynamic therapy adjunct to scaling and root planing in periodontitis patients: a randomized clinical trial. | Pulikkotil, S. J. and Toh, C. G. and Mohandas, K. and Leong, K. V. G. | 6 |
| Effect of controlled-release Periochip TM on clinical and microbiological parameters in patients of chronic periodontitis. | Puri, Komal and Dodwad, Vidya and Bhat, Kishore and Puri, Nikhil | 6 |
| A built-in adjuvant-engineered mucosal vaccine against dysbiotic periodontal diseases | Puth S. and Hong S.H. and Na H.S. and Lee H.H. and Lee Y.S. and Kim S.Y. and Tan W. and Hwang H.S. and Sivasamy S. and Jeong K. and Kook J.-K. and Ahn S.-J. and Kang I.-C. and Ryu J.-H. and Koh J.T. and Rhee J.H. and Lee S.E. | 6 |
| The short-term effects of low-level lasers as adjunct therapy in the treatment of periodontal inflammation | Qadri T. and Miranda L. and Tuner J. and Gustafsson A. | 6 |
| Clinical and microbial evaluation of dental scaling associated with subgingival minocycline in chronic periodontitis subjects. | Querido, Silvia Maria Rodrigues and Cortelli, Sheila Cavalca and Araujo, Marcelo Werneck Barata de and Cortelli, Jose Roberto | 6 |
| A split-mouth study on periodontal and microbial parameters in children with complete unilateral cleft lip and palate | Quirynen M. and Dewinter G. and Avontroodt P. and Heidbuchel K. and Verdonck A. and Carels C. | 6 |
| One stage full- versus partial-mouth disinfection in the treatment of chronic adult or generalized early-onset periodontitis. II. Long-term impact on microbial load | Quirynen M. and Mongardini C. and Pauwels M. and Bollen C.M. and Van Eldere J. and van Steenberghe D. | 6 |
| The effect of a 1-stage full-mouth disinfection on oral malodor and microbial colonization of the tongue in periodontitis patients. A pilot study | Quirynen M. and Mongardini C. and Van Steenberghe D. | 6 |
| Initial subgingival colonization of 'pristine' pockets | Quirynen M. and Vogels R. and Pauwels M. and Haffajee A.D. and Socransky S.S. and Uzel N.G. and van Steenberghe D. | 6 |
| Dynamics of initial subgingival colonization of 'pristine' peri-implant pockets | Quirynen M. and Vogels R. and Peeters W. and Van Steenberghe D. and Naert I. and Haffajee A. | 6 |
| Microbiological and Periodontal Status of Patients Enduring Orthodontic Treatment | Quraeshi S.S. and Hussain M. and Iqbal S.S. and Haider S. and Soomro S.P. and Khan I. | 6 |
| Modulation of pathogenic oral biofilms towards health with nisin probiotic | Radaic A. and Ye C. and Parks B. and Gao L. and Kuraji R. and Malone E. and Kamarajan P. and Zhan L. and Kapila Y.L. AO - Radaic, Allan; ORCID: https://orcid.org/0000-0002-0899-9061 | 6 |
| Erratum: Comparative estimation of salivary total antioxidant capacity in periodontal health and chronic periodontitis - A pilot study (Asian Journal of Pharmaceutical and Clinical Research DOI: 10.22159/ajpcr.2018.v11i10.28409) | Raghavendra U. and Rao A. and Desoza J. and Pai V.R. and Nair S. and Kumar V. and Kalal B.S. | 6 |
| Nigella sativa oil as a treatment for gingivitis: A randomized active-control trial | Rahman I. and Mohammed A. and AlSheddi M.A. and Algazlan A. and Alwably A. and Hebbal M. and Omar M.G. | 6 |
| Knowledge and awareness on the effects of smoking on oral health among general population | Rajkumar K.V. and Santhanam A. and Sangeetha S. | 6 |
| Microbiological Effects of Sodium Hypochlorite/-Amino Acids and Cross-linked Hyaluronic Acid Adjunctive to Non-surgical Periodontal Treatment | Ramanauskaite, E. and Machiulskiene Visockiene, V. and Shirakata, Y. and Friedmann, A. and Pereckaite, L. and Balciunaite, A. and Dvyliene, U. M. and Vitkauskiene, A. and Baseviciene, N. and Sculean, A. | 6 |
| Estimation of red-complex bacteria in diode laser treated chronic periodontitis patients: A clinical and microbiological study | Rangaiah Mahalakshmi M. and Leela R.P. and Yadalam P.K. and Rajula P.B. and Vadivelu S.A. and Maharshi Malakar V. | 6 |
| Machine learning enabled multiplex detection of periodontal pathogens by surface-enhanced Raman spectroscopy | Rathnayake, R. A. C. and Zhao, Z. and McLaughlin, N. and Li, W. and Yan, Y. and Chen, L. L. and Xie, Q. and Wu, C. D. and Mathew, M. T. and Wang, R. R. | 6 |
| Er:YAG laser treatment in supportive periodontal therapy. | Ratka-Kruger, Petra and Mahl, Dominik and Deimling, Daniela and Monting, Jurgen Schulte and Jachmann, Ingeborg and Al-Machot, Elyan and Sculean, Anton and Berakdar, Mohammad and Jervoe-Storm, Pia-Merete and Braun, Andreas | 6 |
| Disease severity associated with presence in subgingival plaque of Porphyromonas gingivalis, Aggregatibacter actinomycetemcomitans, and Tannerella forsythia, singly or in combination, as detected by nested multiplex PCR | Ready D. and D'Aiuto F. and Spratt D.A. and Suvan J. and Tonetti M.S. and Wilson M. | 6 |
| qPCR Detection and Quantification of Aggregatibacter actinomycetemcomitans and Other Periodontal Pathogens in Saliva and Gingival Crevicular Fluid among Periodontitis Patients | Reddahi S. and Bouziane A. and Dib K. and Tligui H. and Ennibi O.K. AO - Ennibi, Oum keltoum; ORCID: https://orcid.org/0000-0002-1224-9124 | 6 |
| Clinical and microbiological effects of subgingival antimicrobial irrigation with citric acid as evaluated by an enzyme immunoassay and culture analysis | Renvert S. and Dahlen G. and Snyder B. | 6 |
| Treatment of periodontal disease based on microbiological diagnosis. Relation between microbiological and clinical parameters during 5 years | Renvert S. and Dahlen G. and Wikstrom M. | 6 |
| Immediate effect of instrumentation on the subgingival microflora in deep inflamed pockets under strict plaque control | Rhemrev G.E. and Timmerman M.F. and Veldkamp I. and Van Winkelhoff A.J. and Van Der Velden U. | 6 |
| Periodontal debridement as a therapeutic approach for severe chronic periodontitis: a clinical, microbiological and immunological study. | Ribeiro, E. del P. and Bittencourt, S. and Sallum, E. A. and Nociti Junior, F. H. and Goncalves, R. B. and Casati, M. Z. | 6 |
| Full-mouth ultrasonic debridement associated with amoxicillin and metronidazole in the treatment of severe chronic periodontitis. | Ribeiro, E. del P. and Bittencourt, S. and Zanin, I. C. J. and Ambrosano, G. M. B. and Sallum, E. A. and Nociti Junior, F. H. and Goncalves, R. B. and Casati, M. Z. | 6 |
| Are putative periodontal pathogens reliable diagnostic markers?. | Riep, Birgit and Edesi-Neuss, Lilian and Claessen, Friderike and Skarabis, Horst and Ehmke, Benjamin and Flemmig, Thomas F and Bernimoulin, Jean-Pierre and Gobel, Ulf B and Moter, Annette | 6 |
| Identification of periodontitis bacteria and resistance test on amoxicillin antibiotics in patients of RSGM Kandea Makassar | Rieuwpassa I.E. and Achmad H. and Susanti A. | 6 |
| Development of a PCR assay specific for <ovid:i>Peptostreptococcus anaerobius</ovid:i> | Riggio, M. P. and Lennon, A. | 6 |
| Clinical and microbiological effects of fixed orthodontic appliances on periodontal tissues in adolescents | Ristic M. and Vlahovic Svabic M. and Sasic M. and Zelic O. | 6 |
| Evaluation of clinical and microbiological measures to predict treatment response in severe periodontitis. | Robertson, P B and Buchanan, S A and Armitage, G C and Newbrun, E and Taggart, E J and Hoover, C I | 6 |
| Clinical and microbiologic evaluation, by real-time polymerase chain reaction, of non-surgical treatment of aggressive periodontitis associated with amoxicillin and metronidazole. | Rodrigues, A. S. and Lourencao, D. S. and Lima Neto, L. G. and Pannuti, C. M. and Hirata, R. D. C. and Hirata, M. H. and Lotufo, R. F. M. and Micheli, G. de | 6 |
| The effects of a new mouthrinse containing chlorhexidine, cetylpyridinium chloride and zinc lactate on the microflora of oral halitosis patients: a dual-centre, double-blind placebo-controlled study. | Roldan, S and Winkel, E G and Herrera, D and Sanz, M and Van Winkelhoff, A J | 6 |
| A short-term clinical and microbial evaluation of periodontal therapy associated with amalgam overhang removal | Roman-Torres C.V.G. and Cortelli S.C. and de Araujo M.W.B. and Aquino D.R. and Cortelli J.R. | 6 |
| Whole Genome Sequencing and Phenotypic Analysis of Antibiotic Resistance in Filifactor alocis Isolates | Romero-Martinez R. and Maher A. and Alvarez G. and Figueiredo R. and Leon R. and Arredondo A. AO - Arredondo, Alexandre; ORCID: https://orcid.org/0000-0003-4459-1300 AO ... | 6 |
| Efficacy of photodynamic therapy and periodontal treatment in patients with gingivitis and fixed orthodontic appliances: Protocol of randomized, controlled, double-blind study | Rosa E.P. and Murakami-Malaquias-Silva F. and Schalch T.O. and Teixeira D.B. and Horliana R.F. and Tortamano A. and Tortamano I.P. and Buscariolo I.A. and Longo P.L. and Negreiros R.M. and Bussadori S.K. and Motta L.J. and Horliana A.C.R.T. | 6 |
| Effect of triclosan on the subgingival microbiota of periodontitis-susceptible subjects | Rosling B. and Dahlen G. and Volpe A. and Furuichi Y. and Ramberg P. and Lindhe J. | 6 |
| Topical antimicrobial therapy and diagnosis of subgingival bacteria in the management of inflammatory periodontal disease | Rosling B.G. and Slots J. and Christersson L.A. | 6 |
| Microbiological and clinical effects of topical subgingival antimicrobial treatment on human periodontal disease. | Rosling, B G and Slots, J and Webber, R L and Christersson, L A and Genco, R J | 6 |
| Impact of the Diet on the Formation of Oxidative Stress and Inflammation Induced by Bacterial Biofilm in the Oral Cavity. | Rowinska, Ilona and Szyperska-Slaska, Adrianna and Zariczny, Piotr and Paslawski, Robert and Kramkowski, Karol and Kowalczyk, Pawel | 6 |
| Indocyanine green based antimicrobial photodynamic therapy as an adjunct to non-surgical periodontal treatment in periodontal maintenance patients: a clinico-microbiological study. | Roy Chowdhury, Urbashi and Kamath, Deepa and Rao, Pooja and Shenoy M, Suchitra and Shenoy, Ramya | 6 |
| Understanding the pathophysiology behind chairside diagnostics and genetic testing for IL-1 and IL-6 | Rudick C.P. and Lang M.S. and Miyamoto T. AO - Rudick, Courtney P.; ORCID: https://orcid.org/0000-0002-8652-1290 | 6 |
| Combined application of Er:YAG and Nd:YAG lasers in treatment of chronic periodontitis. A split-mouth, single-blind, randomized controlled trial. | Saglam, M. and Koseoglu, S. and TasdemIr, I. and Yilmaz, H. E. and Savran, L. and Sutcu, R. | 6 |
| Comparative Evaluation of Conventional Therapy With and Without Use of Diode Laser (DL) in the Treatment of Chronic Generalized Periodontitis: A Clinico-Microbiological Study. | Saha, Ankita and Kamble, Pallavi and Mangalekar, Sachin B | 6 |
| Povidone-iodine gel and solution as adjunct to ultrasonic debridement in nonsurgical periodontitis treatment: an RCT pilot study | Sahrmann P. and Imfeld T. and Ronay V. and Attin T. and Schmidlin P.R. | 6 |
| Effect of application of a PVP-iodine solution before and during subgingival ultrasonic instrumentation on post-treatment bacteraemia: a randomized single-centre placebo-controlled clinical trial | Sahrmann P. and Manz A. and Attin T. and Zbinden R. and Schmidlin P.R. | 6 |
| Lactobacillus salivarius NK02: a Potent Probiotic for Clinical Application in Mouthwash. | Sajedinejad, Neda and Paknejad, Mojgan and Houshmand, Behzad and Sharafi, Hakimeh and Jelodar, Reza and Shahbani Zahiri, Hossein and Noghabi, Kambiz Akbari | 6 |
| Efficacy of Xanthan-Based Chlorhexidine Gel on the Levels of Interleukin-1beta in Chronic Periodontitis: An Interventional Study. | Sajna, H R and Ramesh, Amitha and Kedlaya, Madhurya N and Thomas, Biju | 6 |
| Supragingival and subgingival microbiota of adult patients with Down's syndrome. Changes after periodontal treatment. | Sakellari, D and Belibasakis, G and Chadjipadelis, T and Arapostathis, K and Konstantinidis, A | 6 |
| Prevalence and distribution of Aggregatibacter actinomycetemcomitans serotypes and the JP2 clone in a Greek population. | Sakellari, D. and Katsikari, A. and Slini, T. and Ioannidis, I. and Konstantinidis, A. and Arsenakis, M. | 6 |
| Probiotic Monotherapy with Lactobacillus reuteri (Prodentis) as a Coadjutant to Reduce Subgingival Dysbiosis in a Patient with Periodontitis | Salinas-Azuceno C. and Martinez-Hernandez M. and Maldonado-Noriega J.-I. and Rodriguez-Hernandez A.-P. and Ximenez-Fyvie L.-A. | 6 |
| Nonsurgical periodontal therapy with/without 980 nm diode laser in patients after myocardial infarction: a randomized clinical trial | Samulak R. and Suwala M. and Dembowska E. AO - Samulak, Renata; ORCID: https://orcid.org/0000-0003-0145-9131 AO - Su... | 6 |
| Presence and antibiotic resistance of Porphyromonas gingivalis, Prevotella intermedia, and Prevotella nigrescens in children | Sanai Y. and Persson G.R. and Starr J.R. and Luis H.S. and Bernardo M. and Leitao J. and Roberts M.C. | 6 |
[truncated: 148,142 more chars]
