# Supplementary material for: Diagnostic Accuracy of Microbiome‐Derived Biomarkers in Periodontitis: Systematic Review and Meta‐Analysis
Source: J Periodontal Res. 2025 Jan 13;60(8):748–61. doi: 10.1111/jre.13377 (PMC12476084; doi:10.1111/jre.13377)
Supplement: Supplementary file 6 — Table S5a. [file JRE-60-748-s002.docx]

***S5a. Diagnostic performance assessment of salivary biomarkers***

| Target pathogen | Included studies | Biomarkers | Detection Threshold (Unit) | Total sample (*N*) | Case, Control | Sensitivity, specificity (%) | PPV, NPV(%) | "-LR, +LR" | Youden's Index(a) | Odd ratio (OR) | 95% CI for OR | Significance level p=.05 |
| --- | --- | --- | --- | --- | --- | --- | --- | --- | --- | --- | --- | --- |
|  |  | (Technique type) |  |  |  |  |  |  |  |  |  |  |
| *Porphyromonas gingivalis* | O'Brien-Simpson et al. (2017) | The Monoclonal antibodies to P.g (immunochromatographical device) | 1 × 10⁵ (cells/mL) | 100 | 50,50 | 81.3, 94.0 | 93.1, 83.4 | 0.199. 13.55 | 0.77 | 71.37 | 18.10 - 281.47 | <.0001 |
|  |  |  |  |  |  |  |  |  |  |  |  |  |
|  |  |  |  |  |  |  |  |  |  |  |  |  |
|  | Hyva ̈rinen et al. (2009) | Target Bacterial Load (qPCR) | 8–16 (genome copies/ mL) | 165 | 84, 81 | 80.0, 44.0 | 59.7, 68.0 | 0.45, 1.43 | 0.24 | 3.15 | 1.58 - 6.28 | 0.001 |
|  | Saygun et al. (2011) | Target Bacterial Load (qPCR) | 40000 (copies/mL) | 150 | 82, 68 | 89.2, 94.6 | 93.1, 91.3 | 0.11, 16.49 | 0.84 | 169.9 | 41.56 - 607.12 | <.0001 |
| *Prevotella intermedia* | Hyva ̈rinen et al. (2009) | Target Bacterial Load (qPCR) | 8–16(genome copies/ mL) | 165 | 84, 81 | 51.2, 77.8 | 70.5, 60.6 | 0.63 - 2.30 | 0.29 | 3.67 | 1.87 - 7.22 | <.0001 |
|  | Saygun et al. (2011) | Target Bacterial Load (qPCR) | 910000 copies/mL | 150 | 82, 68 | 86.5, 83.8 | 81.6, 88.2 | 0.16 - 5.33 | 0.7 | 34.79 | 13.89 - 87.16 | <.0001 |
| *Tannerella forsythia* | Hyva ̈rinen et al. (2009) | Target Bacterial Load (qPCR) | 8–16(genome copies/ mL) | 165 | 84, 81 | 94.1, 32.1 | 59.0, 83.9 | 0.19 - 1.39 | 0.26 | 7.47 | 2.70 - 20.65 | <.0001 |
|  | Saygun et al. (2011) | Target Bacterial Load (qPCR) | 700000 (copies/mL) | 150 | 82, 68 | 89.2, 86.5 | 84.6, 90.6 | 0.13 - 6.6018 | 0.76 | 56.25 | 20.54 - 154.05 | <.0001 |
| *Aggregatibacter actinomycetemcomitans* | Hyva ̈rinen et al. (2009) | Target Bacterial Load (qPCR) | 8–16(genome copies/ mL) | 165 | 84, 81 | 42.9, 74.1 | 63.2, 55.6 | 0.77 - 1.65 | 0.17 | 2.14 | 1.11 - 4.14 | 0.023 |
|  | Saygun et al. (2011) | Target Bacterial Load (qPCR) | 2100 (copies/mL) | 150 | 82, 68 | 48.8, 80.9 | 75.5, 56.7 | 0.63 - 2.55 | 0.3 | 4.03 | 1.92 - 8.48 | <.0001 |
| *Campylobacter rectus* | Saygun et al. (2011) | Target Bacterial Load (qPCR) | 700000 (copies/mL) | 150 | 82, 68 | 59.5, 91.9 | 89.1, 65.3 | 0.44 - 6.77 | 0.51 | 15.34 | 5.95 - 39.56 | <.0001 |
| *Fusobacterium nucleatum* | Saygun et al. (2011) | Target Bacterial Load (qPCR) | 5000000 (copies/mL) | 150 | 82, 68 | 70.3, 74.3 | 0.77, 0.67 | 0.40 - 2.73 | 0.45 | 7.25 | 3.51 - 14.99 | <.0001 |
| *Treponema denticola* | Hyva ̈rinen et al. (2009) | Target Bacterial Load (qPCR) | 8–16(genome copies/ mL) | 165 | 84, 81 | 50.0, 66.7 | 60.9, 56.3 | 0.75 - 1.50 | 0.17 | 2 | 1.07 - 3.75 | 0.031 |
| SUBP bacteria | Ma et al. (2021) | The sum of these bacteria relative abundances (16s rRNA Sequencing) | NA (Relative Abundance %) | 67 | 42, 25 | 90.5, 72.0 | 84.4, 81.8 | 0.13 - 3.23 | 0.63 | 24.43 | 6.33 - 94.27 | <.0001 |
| Salivary Endotoxin activity | Zaric et al. (2022) | Salivary endotoxin activity (rFC assays) | 0.01(EU/mL) | 65 | 32, 33 | 69.0, 61.0 | 63.2, 67.0 | 0.51 - 1.77 | 0.3 | 3.38 | 1.22 - 9.41 | 0.02 |

*Abbreviations in Table: PPV: Positive predictive value; NPV: Negative predictive value; -LR: Negative likelihood ratio; +LR: Positive likelihood ratio; 95% CI: 95% confidence interval. NA: Not applicable. Note: Youden's Index(^a^): the optimal diagnostic performance threshold, identifying the point where the balance between sensitivity (true positive rate) and specificity (true negative rate) is maximised: Youden’s Index=Sensitivity+Specificity−1.*
