# Supplementary material for: Diagnostic Accuracy of Microbiome‐Derived Biomarkers in Periodontitis: Systematic Review and Meta‐Analysis
Source: J Periodontal Res. 2025 Jan 13;60(8):748–61. doi: 10.1111/jre.13377 (PMC12476084; doi:10.1111/jre.13377)
Supplement: Supplementary file 7 — Table S5b. [file JRE-60-748-s003.docx]

***S5b. Diagnostic Performance Assessment of Subgingival Biomarkers.***

| Target pathogen | Included studies | Biomarkers | Detection Threhold (Unit) | Total sample (*N*) | Case, Control | Sensitivity, specificity (%) | PPV, NPV(%) | "-LR, +LR" | Youden's index(a) | Odd ratio(OR) | 95% CI for OR | Significance level p=.05 |
| --- | --- | --- | --- | --- | --- | --- | --- | --- | --- | --- | --- | --- |
|  |  | (Technique type) |  |  |  |  |  |  |  |  |  |  |
| *Porphyromonas gingivalis* | Ramseier et al. (2009) | The relative abundance of bacteria (qPCR) | 0.1 (%) | 100 | 39,40 | 80.0, 78.0 | 78.0, 80.0 | 0.26 -3.53 | 0.58 | 13.3 | 4.6 -39.1 | < 0.001 |
| *Prevotella intermedia* | Ramseier et al. (2009) | The relative abundance of bacteria (qPCR) | 0.4 (%) | 100 | 39,40 | 72.0, 73.0 | 72.0, 73.0 | 0.39 – 2.61 | 0.45 | 6.7 | 2.5 - 18 | <0.001 |
| *Treponema denticola* | Ramseier et al. (2009) | The relative abundance of bacteria (qPCR) | 0.2 (%) | 100 | 39,40 | 82.0, 83.0 | 82.0, 83.0 | 0.22 – 4.68 | 0.65 | 21.6 | 6.8 – 68.4 | <.001 |
| *Tannerella forsythia* | Ramseier et al. (2009) | The relative abundance of bacteria (qPCR) | 0.1 (%) | 100 | 39,40 | 80.0, 80.0 | 80.0, 80.0 | 0.26 – 3.97 | 0.6 | 15.5 | 5.2 – 46.4 | <.001 |
| *Campylobacter rectus* | Ramseier et al. (2009) | The relative abundance of bacteria (qPCR) | 0.1 (%) | 100 | 39,40 | 62.0, 63.0 | 62.0, 63.0 | 0.62 – 1.64 | 0.25 | 2.7 | 1.1- 6.6 | <.001 |
| *Fusobacterium nucleatum* | Ramseier et al. (2009) | The relative abundance of bacteria (qPCR) | 2.8 (%) | 100 | 39,40 | 59.0, 60.0 | 59.0, 60.0 | 0.68 – 1.47 | 0.19 | 2.7 | 0.9 – 5.3 | 0.251 |
| *Eikenella corrodens* | Ramseier et al. (2009) | The relative abundance of bacteria (qPCR) | 0.0 (%) | 100 | 39,40 | 21.0, 88.0 | 62.0, 53.0 | 0.91 – 1.64 | 0.15 | 1.8 | 0.5 -6.1 | 0.691 |
| The combination of *Tannerella forsythia, Treponema denticola, Porphyromonas gingivalis, Prevotella intermedia, and Aggregatibacter actinomycetemcomitans* | Arweiler et al. (2020) | The detection of these bacteria (the CST device) | 7.5 × 10⁴ (bacteria/mL) | 125 | 397, 100 | 85.1, 100 | 100.0, 62.9 | 0.15 - unlimited | 0.85 | 1144 | 70.07 - 18661.6 | <.0001 |
| *Porphyromonas gingivalis* and *Tannerella forsythia* | Grundner et al. (2022) | Ceramide phosphoethanolamine (Dot blot) | NA (NA) | 40 | 20, 20 | 95.0, 45.0 | 63.3, 90.0 | 0.11 - 1.73 | 0.4 | 15.6 | 1.73 -139.66 | 0.014 |
| Subgingival endotoxin activity | Zaric et al. (2022) | Subgingival endotoxin activity (rFC assays) | 0.01(EU/mL) | 65 | 32, 33 | 90.6, 87.9 | 87.9, 90.6 | 0.11, 7.48 | 0.79 | 70.1 | 14.39 - 341.27 | <.0001 |
| *Treponema denticola* and *Porphyromonas gingivalis* | Loesche et al. (1990a) | The trypsin-like proteolytic activity (BANA assays) | 1.5 × 10⁶(CFU/mL) | 702 | 452, 250 | 83.9, 42.0 | 72.3, 59.0 | 0.38 - 1.45 | 0.26 | 3.76 | 2.64 - 5.36 | <.0001 |
|  | Hemmings et al. (1997) | The trypsin-like proteolytic activity (BANA assays) | 1 ×10⁵ (CFU/mL) | 126 | 90, 36 | 98.9, 55.6 | 84.8, 95.2 | 0.02, 2.23 | 0.55 | 111 | 13.93 - 888.47 | <.0001 |

*Abbreviations in Table: PPV: Positive predictive value; NPV: Negative predictive value; -LR: Negative likelihood ratio; +LR: Positive likelihood ratio; 95% CI: 95% confidence interval. NA: Not applicable .Note: Youden's Index(^a^): the optimal diagnostic performance threshold, identifying the point where the balance between sensitivity (true positive rate) and specificity (true negative rate) is maximised: Youden’s Index=Sensitivity+Specificity−1.*
