# Supplementary material for: Diagnostic Accuracy of Microbiome‐Derived Biomarkers in Periodontitis: Systematic Review and Meta‐Analysis
Source: J Periodontal Res. 2025 Jan 13;60(8):748–61. doi: 10.1111/jre.13377 (PMC12476084; doi:10.1111/jre.13377)
Supplement: Supplementary file 8 — Table S6a. [file JRE-60-748-s007.docx]

***S6a. Homogeneity and Heterogeneity analysis of*** ***salivary biomarkers***

| No | Index test | Number of evaluation/studies | Control Condition | | Target condition | | Technique | BREM(Y/N) | |  |
| --- | --- | --- | --- | --- | --- | --- | --- | --- | --- | --- |
|  | Name |  | Range of number of samples | Types | Range of number of samples | Types | Name |  |  |  |
| Salivary | | | | | | | | | | |
| 1 | *Porphyromonas gingivalis* | 3 | ≤ 30 (2)  71-120(1) | H (2)  H and G (1) | 31-70 (1)  71-120 (2) | P (2)  CP and AP (1) | Immunochromatographic  Device (1)  qPCR (2) | | Y |  |
| 2 | *Prevotella intermedia* | 2 | ≤ 30(1)  71-120(1) | H (1)  H and G (1) | 31-70(1)  71-120(1) | P (1)  CP and AP (1) | qPCR (2) | | N |  |
| 3 | *Tannerella forsythia* | 2 | ≤ 30(1)  71-120(1) | H (1)  H and G (1) | 31-70(1)  71-120(1) | P (1)  CP and AP (1) | qPCR (2) | | N |  |
| 4 | *Aggregatibacter actinomycetemcomitans* | 2 | 31-70 (1)  71-120 (1) | H (1)  H and G (1) | 71-120(2) | P (1)  CP and AP (1) | qPCR (3) | | N |  |
| 5 | *Campylobacter rectus* | 1 | 31-70(1) | H and G | 71-120(1) | CP and AP (1) | qPCR (1) | | N |  |
| 6 | *Fusobacterium nucleatum* | 1 | 31-70(1) | H and G | 71-120(1) | CP and AP (1) | qPCR (1) | | N |  |
| 7 | *Treponema denticola* | 1 | 71-120(1) | H | 71-120(1) | P (1) | qPCR (1) | | N |  |
| 8 | SUBP bacteria | 1 | ≤ 30(1) | H | 31-70(1) | P (1) | 16s rRNA Sequencing (1) | | N |  |
| 9 | Endotoxin activity | 1 | 31-70(1) | H | 31-70(1) | P (1) | rFC assays (1) | | N |  |

*Abbreviations in Table: BREM: Bivariate Random-Effects Model; SUBP:* the subgingival plaque-specific*; H: Healthy control subjects or periodontal sites; G: Patients or sites with gingivitis; P: Patients or sites with periodontitis; CP: Chronic periodontitis; AP: Aggressive periodontitis; PCP: Progressive Chronic Periodontitis; SCP: Stable Chronic Periodontitis; Y: Yes; N: No.*
