# Supplementary material for: Diagnostic Accuracy of Microbiome‐Derived Biomarkers in Periodontitis: Systematic Review and Meta‐Analysis
Source: J Periodontal Res. 2025 Jan 13;60(8):748–61. doi: 10.1111/jre.13377 (PMC12476084; doi:10.1111/jre.13377)
Supplement: Supplementary file 9 — Table S6b. [file JRE-60-748-s008.docx]

***S6b. Homogeneity and Heterogeneity analysis of*** ***subgingival biomarkers.***

| No | Index test | Number of evaluation/studies | Control Condition | | Target condition | | Technique | BREM(Y/N) |
| --- | --- | --- | --- | --- | --- | --- | --- | --- |
|  | Name |  | Range of number of samples | Types | Range of number of samples | Types | Name |  |
| **1** | The trypsin-like proteolytic activity | 1 | 31-70 (1) | H and G (1) | 71-120 (1) | P (1) | BANA assays | N |
| 2 | *Porphyromonas gingivalis* | 1 | 31-70 (1) | H and G (1) | 71-120 (1) | P (1) | qPCR | N |
| 3 | *Prevotella intermedia* | 1 | 31-70 (1) | H and G (1) | 71-120 (1) | P (1) | qPCR | N |
| 4 | *Aggregatibacter actinomycetemcomitans* | 1 | 31-70 (1) | SCP (1) | 31-70 (1) | PCP (1) | qPCR | N |
|  |  |  |  |  |  |  |  |  |
| 5 | *Treponema denticola* | 1 | 31-70(1) | H and G(1) | 71-120 (1) | P (1) | qPCR | N |
|  |  |  |  |  |  |  |  |  |
| 6 | *Tannerella forsythia* | 1 | 31-70(1) | H and G (1) | 71-120 (1) | P (1) | qPCR | N |
|  |  |  |  |  |  |  |  |  |
| 7 | *Campylobacter rectus* | 1 | 31-70(1) | H and G (1) | 71-120 (1) | P (1) | qPCR | N |
|  |  |  |  |  |  |  |  |  |
| 8 | *Fusobacterium nucleatum* | 1 | 31-70(1) | H and G (1) | 71-120 (1) | P (1) | qPCR | N |
|  |  |  |  |  |  |  |  |  |
| 9 | *Eikenella corrodens* | 1 | 31-70(1) | H and G (1) | 71-120 (1) | P (1) | qPCR | N |
|  |  |  |  |  |  |  |  |  |
| 10 | The combination of *Tannerella forsythia, Treponema denticola, Porphyromonas gingivalis, Prevotella intermedia, and Aggregatibacter actinomycetemcomitans* | 1 | 121-200(1) | H (1) | 71-120(1) | P (1) | The CST device | N |
|  |  |  |  |  |  |  |  |  |
| 11 | *Porphyromonas gingivalis* **and** *Tannerella forsythia* | 1 | ≤ 30 (1) | H (1) | ≤ 30 (1) | P (1) | Dot plot | N |
|  |  |  |  |  |  |  |  |  |
| 12 | Endotoxin activity | 1 | 31-70(1) | H(1) | 31-70(1) | P(1) | rFC assays (1) | N |

*Abbreviations in Table: H: Healthy control subjects or periodontal sites; G: Patients or sites with gingivitis; P: Patients or sites with periodontitis; CP: Chronic periodontitis; AP: Aggressive periodontitis; PCP: Progressive Chronic Periodontitis; SCP: Stable Chronic Periodontitis; BANA test: benzoyl-DL-arginine-naphthylamide (BANA) test; CST: Chair side test.*
